# Preparation and Preclinical Evaluation of ^18^F-Labeled Olutasidenib Derivatives for Non-Invasive Detection of Mutated Isocitrate Dehydrogenase 1 (mIDH1)

**DOI:** 10.3390/molecules29163939

**Published:** 2024-08-21

**Authors:** Roberta Cologni, Marcus Holschbach, Daniela Schneider, Dirk Bier, Annette Schulze, Carina Stegmayr, Heike Endepols, Johannes Ermert, Felix Neumaier, Bernd Neumaier

**Affiliations:** 1 Institute of Neuroscience and Medicine, Nuclear Chemistry (INM-5), Forschungszentrum Jülich GmbH, Wilhelm-Johnen-Straße, 52428 Jülich, Germany; cologniroberta@gmail.com (R.C.); m.holschbach@fz-juelich.de (M.H.); d.schneider@fz-juelich.de (D.S.); d.bier@fz-juelich.de (D.B.); a.schulze@fz-juelich.de (A.S.); heike.endepols@uk-koeln.de (H.E.); j.ermert@fz-juelich.de (J.E.); f.neumaier@fz-juelich.de (F.N.); 2 Institute of Radiochemistry and Experimental Molecular Imaging, Faculty of Medicine and University Hospital Cologne, University of Cologne, Kerpener Str. 62, 50937 Cologne, Germany; 3 Institute of Neuroscience and Medicine, Medical Imaging Physics (INM-4), Forschungszentrum Jülich GmbH, Wilhelm-Johnen-Straße, 52428 Jülich, Germany; c.stegmayr@fz-juelich.de; 4 Department of Nuclear Medicine, Faculty of Medicine and University Hospital Cologne, University of Cologne, Kerpener Str. 62, 50937 Cologne, Germany

**Keywords:** radiofluorination, positron emission tomography (PET), mIDH-selective PET tracer, binding studies

## Abstract

Mutations of isocitrate dehydrogenase 1 (IDH1) are key biomarkers for glioma classification, but current methods for detection of mutated IDH1 (mIDH1) require invasive tissue sampling and cannot be used for longitudinal studies. Positron emission tomography (PET) imaging with mIDH1-selective radioligands is a promising alternative approach that could enable non-invasive assessment of the IDH status. In the present work, we developed efficient protocols for the preparation of four ^18^F-labeled derivatives of the mIDH1-selective inhibitor olutasidenib. All four probes were characterized by cellular uptake studies with U87 glioma cells harboring a heterozygous IDH1 mutation (U87-mIDH) and the corresponding wildtype cells (U87-WT). In addition, the most promising probe was evaluated by PET imaging in healthy mice and mice bearing subcutaneous U87-mIDH and U87-WT tumors. Although all four probes inhibited mIDH1 with variable potencies, only one of them ([^18^F]mIDH-138) showed significantly higher in vitro uptake into U87-mIDH compared to U87-WT cells. In addition, PET imaging with [^18^F]mIDH-138 in mice demonstrated good in vivo stability and low non-specific uptake of the probe, but also revealed significantly higher uptake into U87-WT compared to U87-mIDH tumors. Finally, application of a two-tissue compartment model (2TCM) to the PET data indicated that preferential tracer uptake into U87-WT tumors results from higher specific binding rather than from differences in tracer perfusion. In conclusion, these results corroborate recent findings that mIDH1-selective inhibition may not directly correlate with mIDH1-selective target engagement and indicate that in vivo engagement of wildtype and mutated IDH1 may be governed by factors that are not faithfully reproduced by in vitro assays, both of which could complicate development of PET probes.

## 1. Introduction

**Figure 1:**
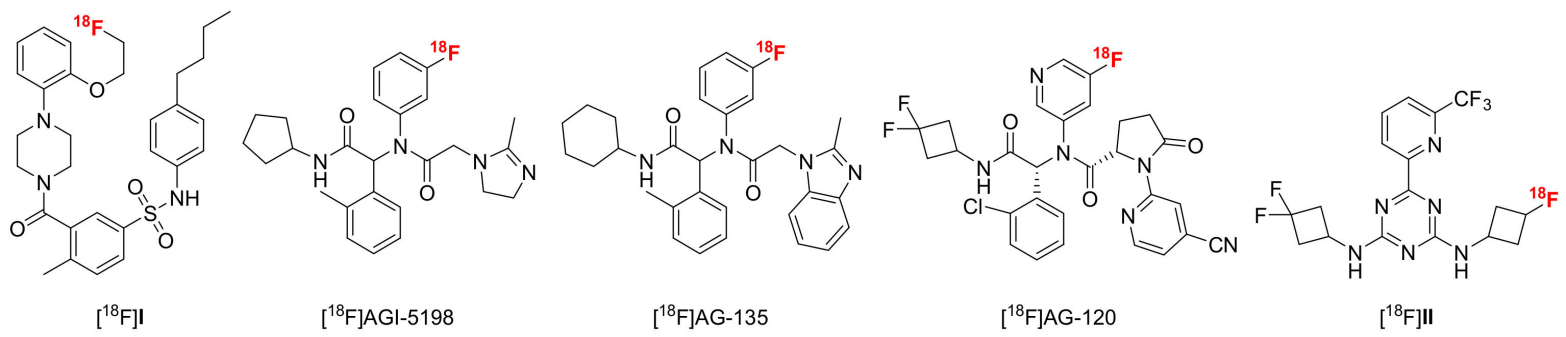
Overview of ^18^F-labeled compounds previously evaluated as candidate PET tracers for non-invasive detection of mutated isocitrate dehydrogenase (mIDH).

Members of the isocitrate dehydrogenase (IDH) family are key metabolic enzymes that catalyze the oxidative decarboxylation of isocitrate to α-ketoglutarate (α-KG) in the cytosol and peroxisomes (IDH1) or mitochondria (IDH2 and IDH3) of all eukaryotic cells [1]. Heterozygous missense mutations in the genes encoding IDH1 or, less commonly, IDH2 are frequent in diffuse gliomas and may drive malignant transformation during early stages of the disease. In the vast majority of IDH mutated gliomas (>90%), these mutations are located at codon 132 of IDH1 and result in an arginine-to-histidine (R132H) substitution at the active site, although other mutations affecting the corresponding arginine residue in IDH1 or IDH2 have been described as well [1,2,3]. All of these mutations equip the enzyme with a neomorphic activity that results in increased conversion of α-KG into the potential oncometabolite 2-hydroxyglutarate (2-HG) [4,5]. High concentrations of 2-HG competitively suppress a number of α-KG-dependent enzymes involved in epigenetic regulation, which could in turn predispose affected cells to more aggressive mutations [4,6]. Although the therapeutic benefit of targeting mutated IDH (mIDH) in gliomas remains controversial [7,8], numerous mIDH-selective inhibitors have been developed to date and in many cases shown to effectively suppress 2-HG production in patients or preclinical tumor models [3,9,10]. Interestingly, IDH-mutated gliomas have also consistently been demonstrated to be associated with a better prognosis than comparable IDH wildtype gliomas [5,11,12,13], which resulted in inclusion of the IDH status into the WHO classification of adult-type diffuse gliomas [14,15,16,17]. In particular, IDH mutations are now considered as a disease-defining feature of oligodendrogliomas and astrocytomas, while IDH wildtype gliomas (designated as glioblastomas) are recognized as a separate, more aggressive disease with distinct molecular genetics [15,16,17]. Accordingly, assessment of the IDH status by immunohistochemistry and/or genomic sequencing has become an integral part of the diagnostic algorithm for glioma classification. However, while these approaches allow for reliable detection of the most frequent IDH mutations, they require invasive tissue sampling and cannot be used for follow-up examinations or longitudinal studies. In contrast, positron emission tomography (PET) imaging enables the non-invasive visualization of molecular targets or biochemical processes with radiolabeled probes, making it a promising approach for routine assessment of the IDH status. For example, a number of established PET tracers like *O*-(2-[^18^F]fluoroethyl)tyrosine ([^18^F]FET) or 6-[^18^F]fluoro-3,4-dihydroxyphenylalanine ([^18^F]FDOPA) have been successfully used to infer the IDH mutation status in gliomas [18,19,20,21,22]. Nevertheless, these probes can only provide indirect evidence for the presence of IDH mutations, and their diagnostic accuracy for detection of mIDH is limited. As such, recent efforts have focused on the development of mIDH-selective PET tracers to facilitate glioma classification and possibly delineation by direct detection of mIDH expression (which is expected to be homogenous among tumor cells). Unfortunately, most candidate PET tracers evaluated in previous studies have proven to be unsuitable for glioma imaging (reviewed in [23]). Thus, radiolabeled analogs of the butyl-phenyl sulfonamide class of mIDH1 inhibitors (like [^18^F]**I** in Figure 1) showed limited mIDH selectivity in cellular uptake studies and very low brain uptake in rodents [24]. Likewise, phenylglycine-based radioligands derived from the FDA-approved mIDH1 inhibitor ivosidenib or its preclinical analogs either showed limited in vivo tumor uptake and retention ([^18^F]AGI-5198 & [^18^F]AG-135, Figure 1) or insufficient brain uptake to be useful for glioma imaging ([^18^F]AG-120, Figure 1) [25,26,27]. Conversely, while an ^18^F-labeled aminotriazine-based tracer candidate derived from the pan-mIDH1/2 inhibitor vorasidenib ([^18^F]**II** in Figure 1) showed promising in vitro and in vivo mIDH specificity, this radioligand suffered from extensive in vivo defluorination [28].

**Figure 2:**
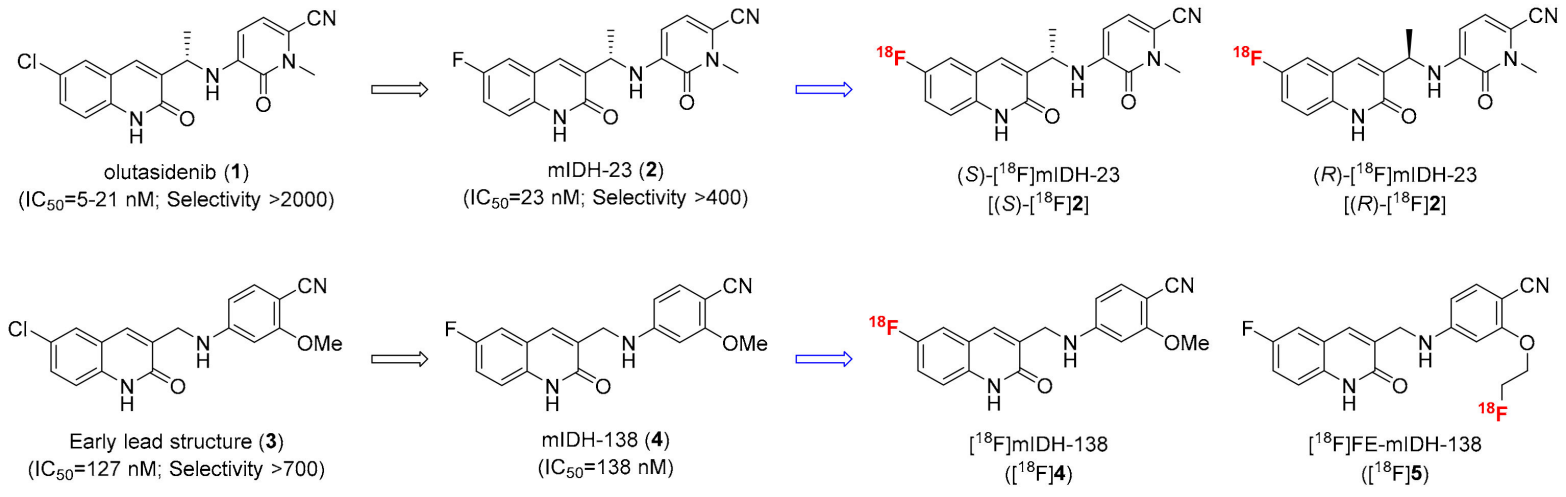
mIDH1-selective quinolinone-based inhibitors selected as lead structures (left) and radiolabeled analogs prepared in the present study (right). IC_50_ values indicated below the lead structures correspond to the reported potency for inhibition of the most frequent mIDH1_R132H_ mutation in biochemical assays, while the selectivity factors correspond to the ratio of the biochemical IC_50_ values for mIDH1_R132H_ and the wildtype enzyme.

Another class of potential lead structures for the development of mIDH-selective PET tracers are quinolinone-based inhibitors like the clinical candidate olutasidenib and its preclinical analogs, which have been shown to selectively inhibit several IDH1 mutations with low nanomolar IC_50_ values and exhibit favorable pharmacokinetic properties (e.g., high in vivo stability and effective brain penetration) [29,30]. In addition, replacement of the 6-chloro substituent in olutasidenib (**1**) by fluorine had no major effects on mIDH1-selective inhibition of the resulting analog (mIDH-23, **2**) in functional assays (Figure 2), and its ^18^F-labeled isotopolog was reported to be stable during incubation in EtOH or mouse serum [31]. However, neither the cellular uptake nor the in vivo properties of this radioligand have been evaluated to date, so that the suitability of quinolinone-based tracers for mIDH-selective PET imaging remains to be established. As such, the aim of the present work was to prepare several ^18^F-labeled olutasidenib derivatives and to evaluate their mIDH selectivity by cellular uptake studies and (for the best candidate) in vivo PET imaging. Apart from the known ^18^F-labeled olutasidenib derivative (*S*)-[^18^F]mIDH-23 [(*S*)-[^18^F]**2**], we prepared and evaluated its (*R*)-methyl enantiomer (*R*)-[^18^F]mIDH-23 [(*R*)-[^18^F]**2**]. In addition, an early quinolinone lead structure (**3**) with good inhibitory potency and excellent selectivity for the most frequent mIDH1_R132H_ mutation [29] was modified by replacement of the 6-chloro substituent with fluorine (Figure 2). The resulting analog (mIDH-138, **4**), which has been shown to inhibit mIDH1_R132H_ with similar potency as **3** [29], was either radiofluorinated without further structural modification to obtain [^18^F]mIDH-138 ([^18^F]**4**) or by replacement of the methoxy group on the cyanobenzene moiety with a [^18^F]fluoroethoxy group to obtain [^18^F]FE-mIDH-138 ([^18^]**5**) (Figure 2).

## 2. Results and Discussion

### 2.1. Chemistry and Radiochemistry

#### 2.1.1. Synthesis of Reference Compound (**4**) and Radiolabeling Precursors (11, 18, 19) for the Preparation of [^18^F]mIDH-138 ([^18^F]**4**)

**Scheme 1:**
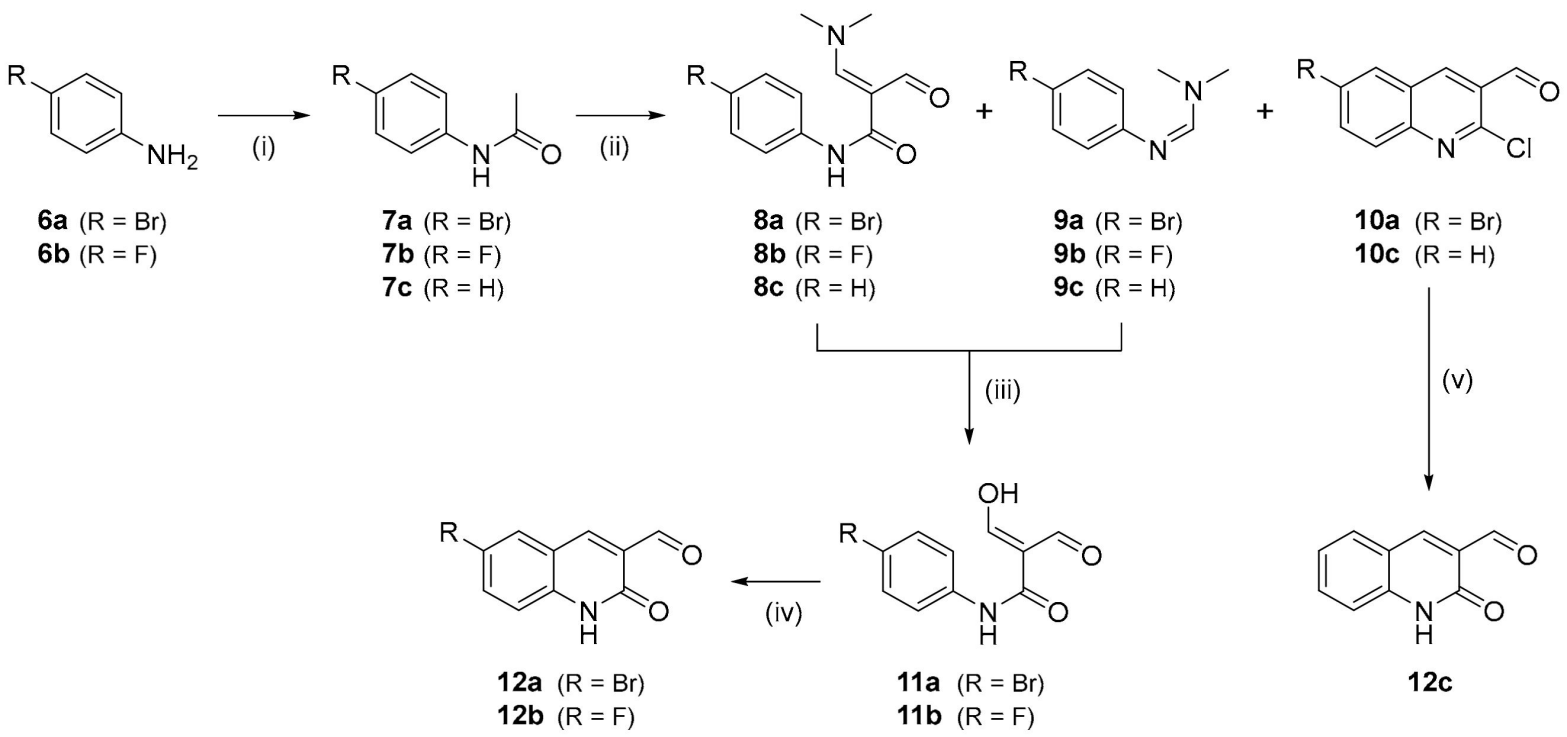
Preparation of 3-formylquinolone intermediates **12a**–**c**. Conditions: (i) DIPEA, Ac_2_O, AcOEt, rt, 16 h; (ii) DMF, POCl_3_, 75 °C, 1.2 h; (iii) a: EtOH:20% NaOH_aq_ (1.1:1.0), b: 0.1 m HCl, 3 h; (iv) PPA, 150 °C, 15 min; (v) 2 m HCl, reflux, 16 h.

To prepare the reference compound and radiolabeling precursors for [^18^F]mIDH-138 ([^18^F]**4**), the 3-formyl-2-quinolone scaffold was first constructed according to the methodology reported by Meth-Cohn et al. [32,33]. To this end, the *para*-halogenated anilines **6a**, and **6b** were acetylated to obtain the acetanilides **7a** and **7b** (Scheme 1). Subsequent formylation of **7a**, **7b** or commercial acetamide **7c** afforded mixtures of compounds **8a**–**c**, **9a**–**c** and **10a**,**c** in a ratio that depended on the degree of activation of the respective starting material. The mixtures of **8a**,**b** and **9a**,**b** were then converted into the corresponding malondialdehydes **11a**,**b**, which were treated with polyphosphoric acid to furnish the analytically pure 6-halogenated 3-formyl-2-quinolones **12a**,**b**. The unsubstituted 3-formyl-2-quinolone **12c** was prepared by boiling **10c** with hydrochloric acid. Overall, 6-bromo-3-formyl-2-quinolone **12a** and 6-fluoro-3-formyl-2-quinolone **12b** were obtained in total yields of 30% and 39% over four steps, while unsubstituted 3-formyl-2-quinolone **12c** was synthesized in a total yield of 40% over three steps.

**Scheme 2:**
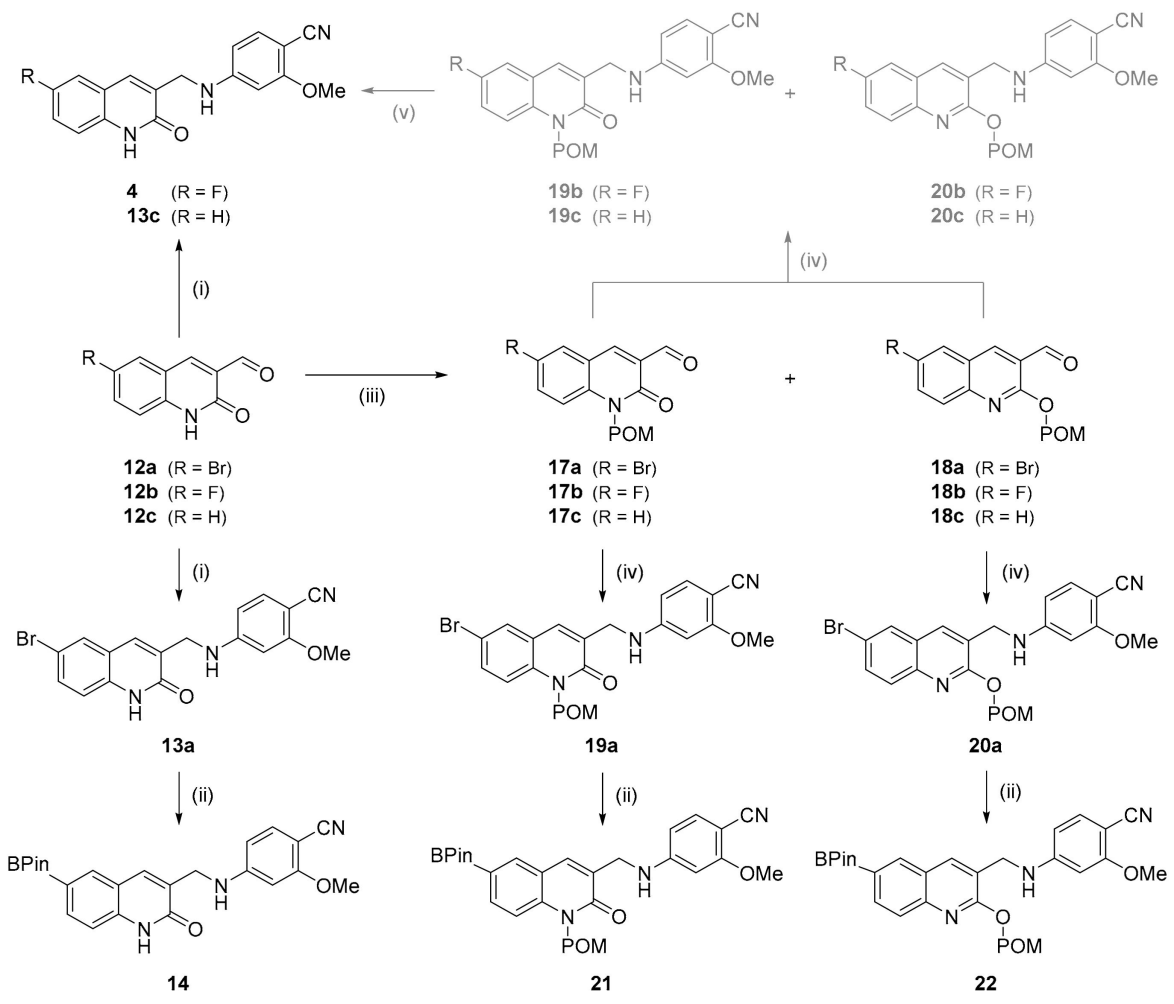
Preparation of reference compound (**4**) and radiolabeling precursors (**14**, **21**, **22**) for [^18^F]mIDH-138 ([^18^F]**4**). Conditions: (i) **16**, DCE, AcOH, NaBH(OAc)_3_, rt, 16 h; (ii) (Bpin)_2_, KOAc, Pd(dppf)Cl_2_, dioxane, 80 °C, 22 h; (iii) POM-Cl, K_2_CO_3_, DMF, 60 °C, 2 h; (iv) **16**, TMS-Cl or TMS-OTf, NaBH_4_, DMF, 0 °C, 75–120 min; (v) 2 N NaOH, MeOH.

Next, the aldehyde moiety in **12a**,**b** was reductively aminated with 4-amino-2-methoxybenzonitrile **16** (prepared by reduction of commercial 4-nitro-2-methoxybenzonitrile **15**, see Section S2.1 in Supplementary Materials) to derive bromo-substituted quinolone **13a** and the fluoro-substituted reference compound **4** in 45% and 33% yield, respectively (Scheme 2) [34]. Subsequent Miyaura borylation of **13a** [35] afforded the non-protected radiolabeling precursor **14** in 17% yield. 

Since the amide moiety of **14** interfered with Cu-mediated radiofluorination (see below), we also prepared the *N*- and *O*-protected radiolabeling precursors. To this end, a mixture of the *N*- and *O*-protected intermediates **17a** and **18a** was prepared by introducing a pivaloyloxymethyl (POM) protecting group into **12a** (Scheme 2) [36]. After separation and isolation by column chromatography, **17a** and **18a** were conjugated with aniline **16**, which furnished the protected quinolones **19a** and **20a** [37]. Finally, conversion of **19a** or **20a** into the corresponding boronic acid pinacol esters (as described above for preparation of **14**) afforded the *N*- or *O*-POM protected radiolabeling precursors **21** or **22** in 87% or 80% yield, respectively. 

Due to the higher solubility and reactivity of the POM-protected compared to the non-protected intermediates, reference compound **4** and its unsubstituted analog **13c** (for identification of protodeboronated side products formed during radiofluorination) could also be prepared by conjugation of **17a** or **17b** and **18a** or **18b** with aniline **16** and subsequent de-protection of the resulting mixtures of **19b**,**c** and **20b**,**c** (Scheme 2).

#### 2.1.2. Radiosynthesis of [^18^F]mIDH-138 ([^18^F]**4**)

**Scheme 3:**
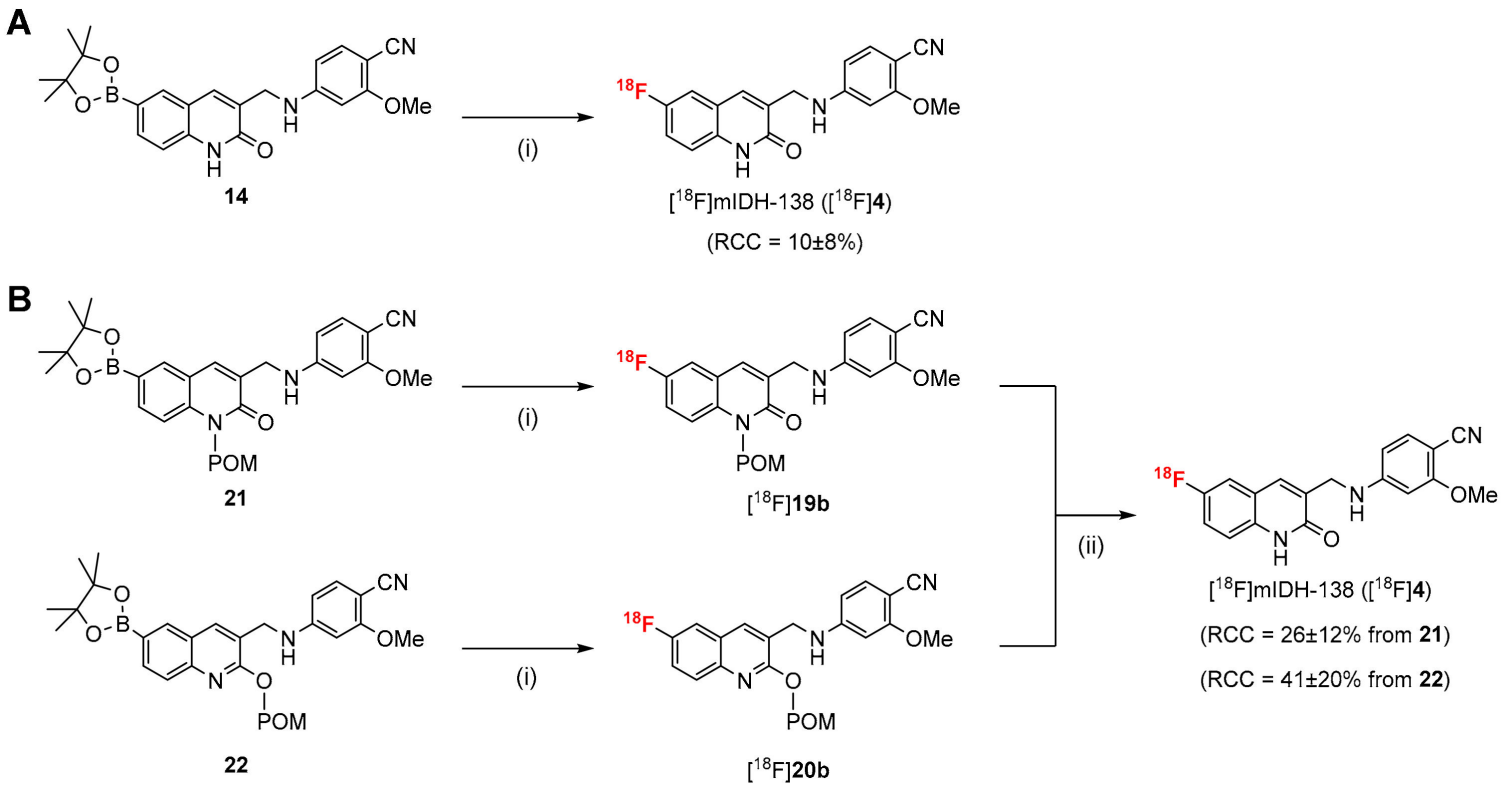
Radiosynthesis of [^18^F]mIDH-138 ([^18^F]**4**) by Cu-mediated radiofluorination of non-protected (**A**) or protected (**B**) precursors. Conditions: (i) elution of [^18^F]F^−^ with Et_4_NHCO_3_ in MeOH followed by evaporation of MeOH, addition of precursor (**14**, **21** or **22**) and Cu(4-PhePy)_4_(ClO_4_)_2_ (10 µmol of each) in DMA, 110 °C, 10 min; (ii) 0.25 m NaOH_aq_ in MeOH, 80 °C, 3 min. Abbreviations: RCC, radiochemical conversion.

The radiosynthesis of [^18^F]mIDH-138 ([^18^F]**4**) was initially attempted by copper-mediated radiofluorination of the non-protected boronic acid pinacol ester **14** with no carrier added [^18^F]fluoride ([^18^F]F^−^) (Scheme 3A). To this end, aqueous [^18^F]F^−^ was trapped on a QMA cartridge and eluted with a solution of Et_4_NHCO_3_ in MeOH. After evaporation of the solvent and addition of precursor **14** and Cu(4-PhPy)_4_(ClO_4_)_2_ [38] (10 µmol of each) in DMA, the reaction mixture was stirred for 10 min at 110 °C. This approach afforded [^18^F]**4**, but only in low radiochemical conversions (RCCs) of 10 ± 8% (n = 3), as determined by radio-HPLC analysis. Since partial chelation of the copper mediator by the amide moiety in precursor **14** could potentially reduce the ^18^F-labeling efficacy, we carried out a series of spiking experiments with the bromo-substituted analog **13a** to evaluate its effects on the radiofluorination of 4-acetylbenzeneboronic acid as a benchmark reaction (see Section S3.4.2 in Supplementary Materials) [39]. As suspected, addition of **13a** completely prevented the formation of 4-[^18^F]fluoroacetophenone, indicating a need for appropriate protection groups to minimize the detrimental effects of the amide moiety in **14** on ^18^F-fluorination. Accordingly, the radiosynthesis of [^18^F]**4** was instead performed with the *N*- or *O*-protected precursors **21** or **22** (Scheme 3B). After copper-mediated radiofluorination according to the same protocol as described above, the reaction mixture was cooled to 80 °C and 0.25 m NaOH_aq_ in MeOH was added to hydrolyze the POM-protecting groups. Stirring was continued for three minutes and the mixture was acidified with 5% TFA in MeCN. Most of the solvents and the copper mediator were then removed by solid phase extraction (SPE), the product was purified by semi-preparative HPLC and the resulting eluate was loaded onto an HLB cartridge. Subsequent elution of the product with 1 mL EtOH followed by evaporation of the alcohol and formulation with PBS buffer and 1% Tween 80 afforded [^18^F]**4** as a ready-to-inject solution with residual solvent and copper levels below the limit of detection.

Using precursor **21**, [^18^F]**4** was obtained in RCCs of 26 ± 12% (n = 15), isolated radiochemical yields (RCYs) of 22 ± 11% (n = 11), a molar activity (A_m_) of 13–180 GBq/µmol (from 1–4 GBq starting activities) (n = 7) and radiochemical purities (RCPs) of >99%. Radiofluorination of precursor **22** afforded [^18^F]**4** in RCCs of 41 ± 20% (n = 38), RCYs of 29 ± 11% (n = 30), an A_m_ of 13–540 GBq/µmol (from 1–4 GBq starting activities) (n = 18) and RCPs of >99%.

#### 2.1.3. Synthesis of Reference Compounds [(*S*)- and (*R*)-2] and Radiolabeling Precursors [(*S*)- and (*R*)-29, (*S*)- and (*R*)-30] for (*S*)- and (*R*)-[^18^F]mIDH-23 [(*S*)- and (*R*)-[^18^F]**2**]

**Scheme 4:**
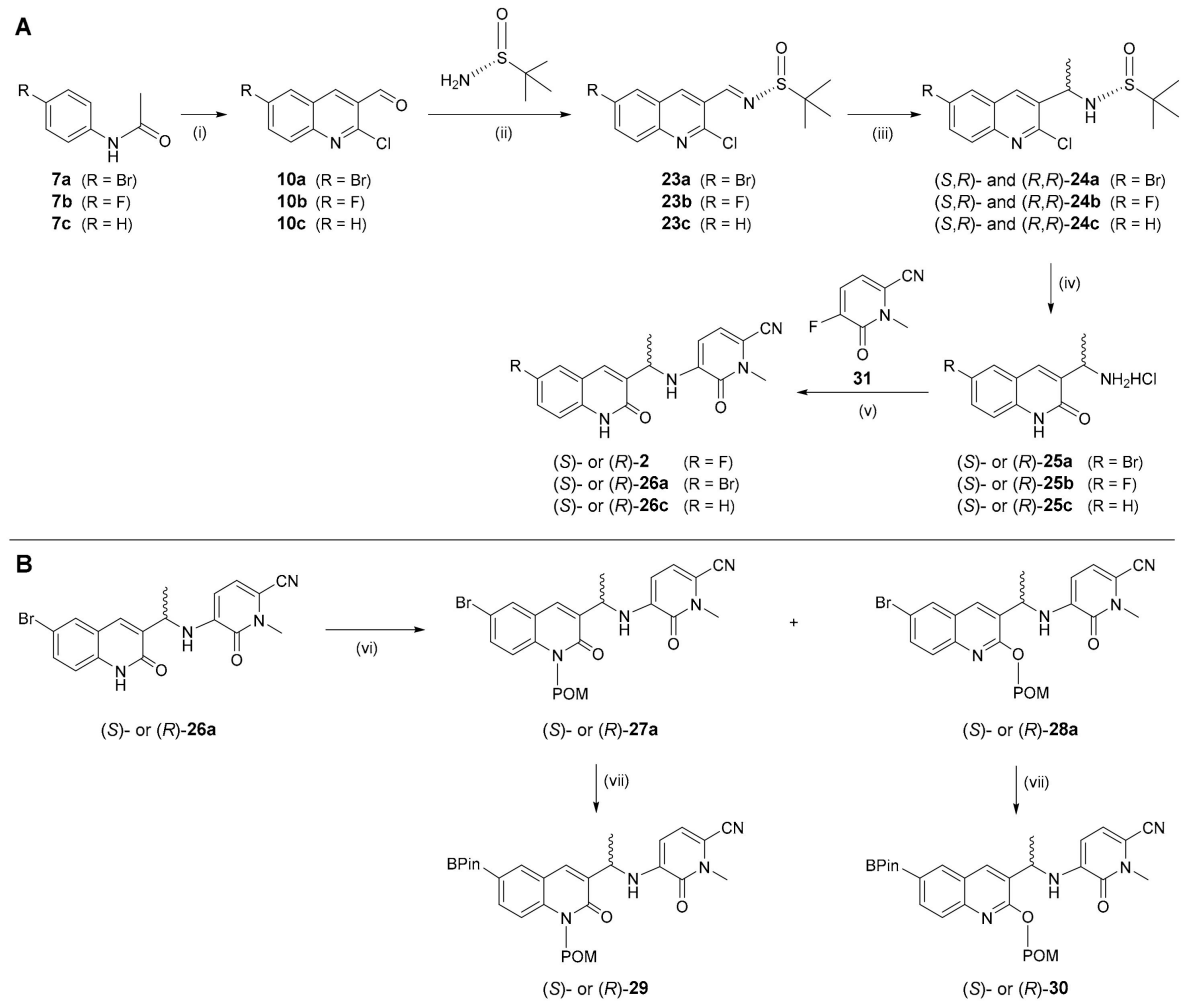
Preparation of reference compounds [(*S*)- and (*R*)-**2**] (**A**) and radiolabeling precursors [(*S*)- and (*R*)-**29**, (*S*)- and (*R*)-**30**] (**B**) for (*S*)- and (*R*)-[^18^F]mIDH-23 [(*S*)- and (*R*)-[^18^F]**2**]. Conditions: (i) triphosgene, DMF, 75 °C, 4 h; (ii) CuSO_4_, (*R*)-2-methylpropane-2-sulfinamide, DCE, 55 °C, 16 h; (iii) MeMgBr in Et_2_O, CH_2_Cl_2_, -60 °C to rt, 16 h; (iv) 1 N HCl:dioxane (1:1), reflux, 2 h; (v) **31**, DIPEA, DMSO, 110 °C, 16 h; (vi) K_2_CO_3_, POM-Cl, DMF, rt, 16 h; (vii) (Bpin)_2_, KOAc, Pd(dppf)Cl_2_, dioxane, 80 °C, 3 h.

Preparation of the reference compounds and radiolabeling precursors for (*S*)- and (*R*)-[^18^F]mIDH-23 [(*S*)- and (*R*)-[^18^F]**2**] started with the synthesis of formylquinolines **10a**–**c**. Due to the poor yields of **10a**,**c** obtained with the POCl_3_-based approach shown in Scheme 1, formylation of acetanilides **7a**–**c** was instead carried out using triphosgene [40], which afforded **10a**–**c** in about 40% yields (Scheme 4A). Subsequent condensation with the commercial chiral auxiliary (*R*)-(+)-2-methyl-2-propanesulfinamide (Ellman’s Sulfinamide) [41,42] followed by methylation of the resulting imines **23a**–**c** with methyl magnesium bromide afforded (*S*,*R*)- and (*R*,*R*)-**24a**–**c** in moderate yields [43]. The chiral auxiliary was removed under acidic conditions and the resulting amine salts (*S*)- and (*R*)-**25a**–**c** were directly reacted with *N*-methylpyridone **31** (for preparation see Section S2.2 in Supplementary Materials) to afford bromo-substituted intermediates (*S*)- and (*R*)-**26a**, fluoro-substituted reference compounds (*S*)- and (*R*)-**2** and their unsubstituted analogs (*S*)- and (*R*)-**26c**. Finally, (*S*)- or (*R*)-**26a** were POM-protected as described above and the resulting *N*- or *O*-protected isomers [(*S*)*-* or (*R*)-**27** and (*S*)- or (*R*)-**28**] converted into the corresponding boronic acid pinacol esters to afford radiolabeling precursors (*S*)- or (*R*)-**29** and (*S*)- or (*R*)-**30**, respectively (Scheme 4B). 

The relative stereochemistry of the methyl group in the prepared compounds was determined by polarimetry, while the absolute stereochemistry was assigned by comparing the NMR spectra of intermediates (*S*,*R*)- and (*R*,*R*)-**24b** with the ones reported by Weber et al., who also measured circular dichroism spectra of the fluorinated products [31]. This allowed for configurational assignment of the radiolabeling precursors by chiral HPLC (see Section S3.3.3 in Supplementary Materials).

#### 2.1.4. Radiosynthesis of (*S*)- and (*R*)-[^18^F]mIDH-23 [(*S*)- and (*R*)-[^18^F]**2**]

**Scheme 5:**
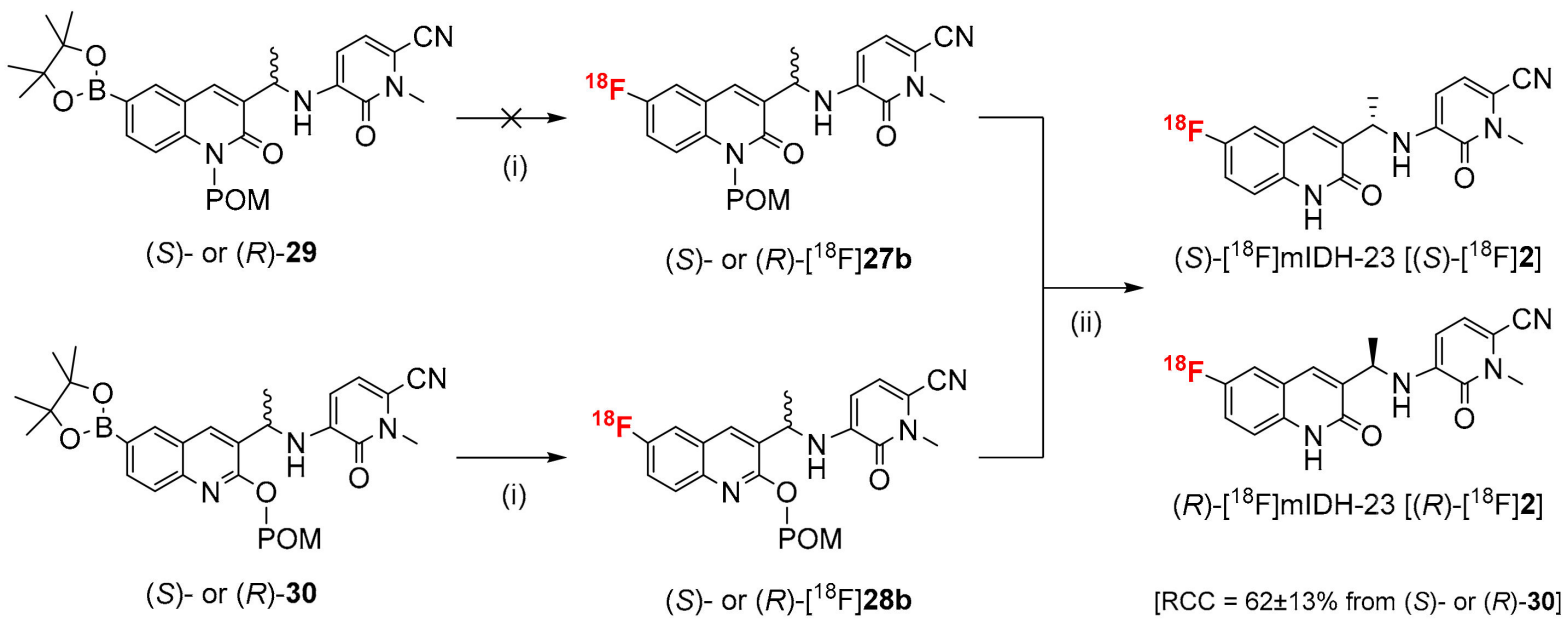
Radiosynthesis of (*S*)- and (*R*)-[^18^F]mIDH-23 [(*S*)- and (*R*)-[^18^F]**2**] by Cu-mediated radiofluorination. Conditions: (i) elution of [^18^F]F^−^ with Et_4_NHCO_3_ in MeOH followed by evaporation of MeOH, addition of precursor [(*S*)- or (*R*)-**29** or (*S*)- or (*R*)-**30**] and Cu(4-PhePy)_4_(ClO_4_)_2_ (10 µmol of each) in DMI, 100 °C, 15 min; (ii) 0.25 m NaOH_aq_ in MeOH, 80 °C, 3 min followed by quench with 5% TFA in MeCN.

The radiosynthesis of (*S*)- and (*R*)-[^18^F]mIDH-23 [(*S*)- and (*R*)-[^18^F]**2**] was initially performed under the same conditions as described above for [^18^F]**4**, which resulted in no ^18^F-incorporation in the case of the *N*-protected precursors [(*S*)- or (*R*)-**29**] and afforded the desired tracers in rather low RCCs when the *O*-protected precursors [(*S*)- or (*R*)-**30**] were used. In subsequent optimization studies with different precursor amounts and reaction solvents, temperatures or times (see Section S3.3.1 in Supplementary Materials), the highest RCCs were achieved when (*S*)- or (*R*)-**30** (10 µmol) were radiofluorinated for 15 min at 100 °C in DMI, followed by de-protection of the radiolabeled intermediates with 0.25 m NaOH at 80 °C for 3 min and acidification with 5% TFA in MeCN (Scheme 5). After purification by SPE and semi-preparative HPLC (as described above for [^18^F]**4**), this protocol afforded (*S*)- and (*R*)-[^18^F]**2** in RCCs of 61 ± 13% (n = 6), RCYs of 50 ± 10% (n = 4), an A_m_ of 102-275 GBq/µmol (from approximately 1 GBq starting activity) (n = 2) and RCPs of >99%. The residual solvent and copper levels were well below the acceptable limit, and HPLC analyses with co-injection of the unsubstituted analogs (*S*)- or (*R*)-**26c** demonstrated that the protodeboronated side products formed during radiofluorination were efficiently removed by semi-preparative HPLC (see Section S3.3.1 in Supplementary Materials). 

#### 2.1.5. Synthesis of Reference Compound (**5**) and Radiolabeling Precursor (**39**) for [^18^F]FE-mIDH-138 ([^18^F]**5**) 

**Scheme 6:**
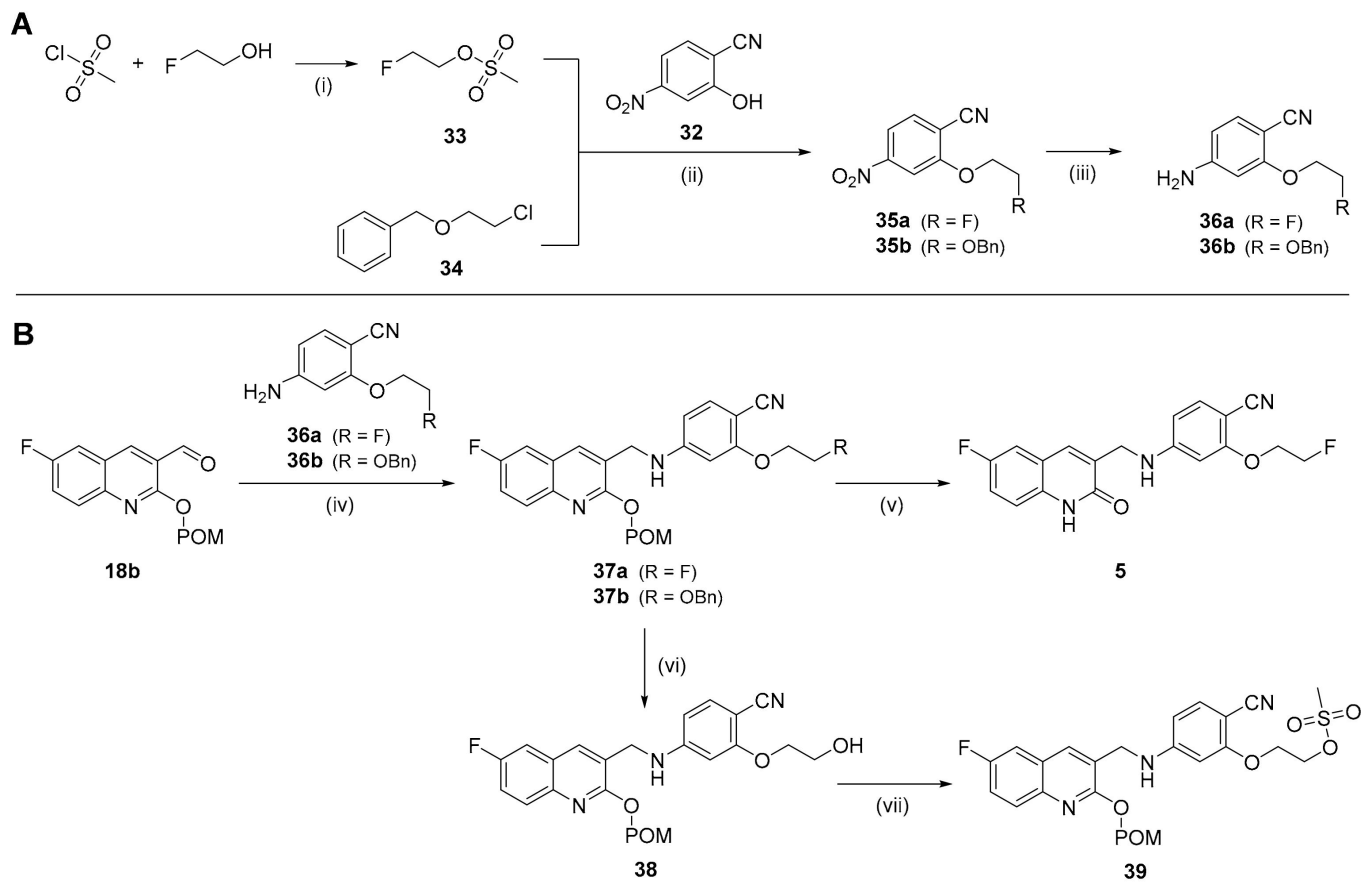
Preparation of intermediates **36a**,**b** (**A**) and the reference compound (**5**) and radiolabeling precursor (**39**) (**B**) for [^18^F]FE-mIDH-138 ([^18^F]**5**). Conditions: (i) Ms-Cl, NEt_3_, CH_2_Cl_2_, rt, 3 h; (ii) **33** or **34**, Cs_2_CO_3_, DMF, 60 °C, 2.5 h; (iii) Zn powder, NH_4_Cl, EtOH, rt; (iv) **36a**,**b**, TMS-OTf, NaBH_4_, DMF, 0 °C, 5 h; (v) 40% NaOH_aq_ in MeOH, 50 °C, 1 h; (vi) Pd/C, EtOH, rt, 5 h; (vii) Ms-Cl, NEt_3_, CH_2_Cl_2_, 0 °C to rt, 2.5 h.

The reference compound and the radiolabeling precursor for [^18^F]FE-mIDH-138 ([^18^F]**5**) were prepared analogous to the methodology described in Section 2.1.1 by conjugating the POM-protected 3-formylquinolone **18b** with appropriate anilines. The necessary anilines **36a** and **36b** were obtained by *O*-alkylation of 2-hydroxy-4-nitrobenzonitrile **32** with 2-fluoroethyl methanesulfonate **33** (prepared by esterification of fluoroethyl alcohol with methanesulfonyl chloride [44]) or commercially available [(2-chloroethoxy)methyl]benzene **34 [36]**, and subsequent reduction in the resulting nitroarenes **35a**,**b** [45] (Scheme 6A).

Compounds **36a**,**b** were then conjugated with **18b** to afford the protected quinolones **37a**,**b** (Scheme 6B) [37]. Subsequent de-protection of **37a** with NaOH_aq_ in MeOH furnished the reference compound **5** in 10% yield, while selective hydrogenation of the benzylic protecting group in **37b** afforded POM-protected alcohol **38** in almost quantitative yield [46]. Finally, **38** was treated with methanesulfonyl chloride to obtain the *O*-protected radiolabeling precursor **39** [44]. Attempts to also prepare the corresponding *N*-protected radiolabeling precursor were unsuccessful, since Pd/C-catalyzed hydrogenation resulted in cleavage of the benzylic amine linker in the *N*-protected intermediate.

#### 2.1.6. Radiosynthesis of [^18^F]FE-mIDH-138 ([^18^F]**5**)

**Scheme 7:**
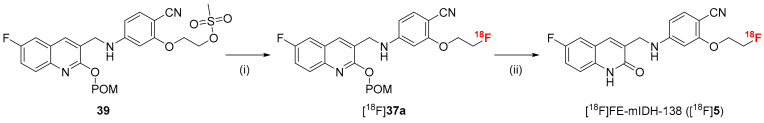
Radiosynthesis of [^18^F]FE-mIDH-138 ([^18^F]**5**) by ‘minimalist’ aliphatic radiofluorination. Conditions: (i) elution of [^18^F]F^−^ with Me_4_NOTf in MeOH followed by evaporation of MeOH, addition of **39** (2 mg) in MeCN, 85 °C, 15 min; (ii) 0.1 m NaOH_aq_ in EtOH, 35 °C, 10 min.

Due to the base liability of the ethoxy side chain, the reaction conditions for radiofluorination of **39** and subsequent de-protection of the radiolabeled intermediate [^18^F]**37a** had to be separately optimized to identify suitably mild basic conditions (see Section S3.4.1 in Supplementary Materials). The best results were obtained when [^18^F]F^−^ pre-processing was performed under ‘minimalist’ conditions by trapping aqueous [^18^F]F^−^ on a QMA cartridge, removing remaining water with MeOH and air, and eluting the [^18^F]F^−^ with a solution of Me_4_NOTf in MeOH (Scheme 7). MeOH was evaporated at 85 °C under reduced pressure for 5 min before a solution of **39** (3.5 µmol) in MeCN was added. The reaction mixture was then stirred for 15 min at 85 °C and cooled to room temperature. For de-protection of the radiolabeled intermediate, 0.1 m NaOH_aq._ in EtOH was added and the resulting mixture was stirred at 35 °C for 10 min (Scheme 7). Finally, the reaction was quenched by addition of H_2_O and the crude product was purified via semi-preparative HPLC, which afforded [^18^F]**5** in RCYs of 26 ± 8% (n = 3), an A_m_ of 14-47 GBq/µmol (n = 4) (from approximately 1 GBq starting activity) and RCPs of >99%.

### 2.2. In Vitro Evaluation

#### 2.2.1. Lipophilicity

As a measure for the lipophilicity of the four radiolabeled probes, their partition coefficients (logP) and distribution coefficients at pH 7.4 (logD_7.4_) were determined by the shake-flask method. Table 1 summarizes the results of these experiments and compares them with the predicted logD_7.4_ values calculated using the ADMETlab platform [47]. Overall, the experimentally determined logD_7.4_ values showed good agreement with the predicted values and ranged from 2.5 to 2.7, indicating a moderate lipophilicity of the compounds.

#### 2.2.2. In Vitro Stability 

The in vitro stability of the radiolabeled probes was evaluated by incubation studies with phosphate-buffered saline (PBS) and rat serum. The results of these experiments showed no evidence for decomposition after incubation of the four compounds in PBS at room temperature for up to three hours (see Figure S18 in Section S4.1.2 of Supplementary Materials). In addition, all four radiotracers proved to be stable for at least one hour when incubated in rat serum at 37 °C (see Figure S19 in Section S4.1.3 of Supplementary Materials). Finally, the stability of the non-labeled reference compounds in organic solution (DMSO) was investigated, which revealed no decomposition of (*S*)-**2** and less than 10% decomposition of **4** and **5** for at least 28 h (see Figure S17 in Section S4.1.1 of Supplementary Materials).

#### 2.2.3. Inhibitory Potency

**Figure 3:**
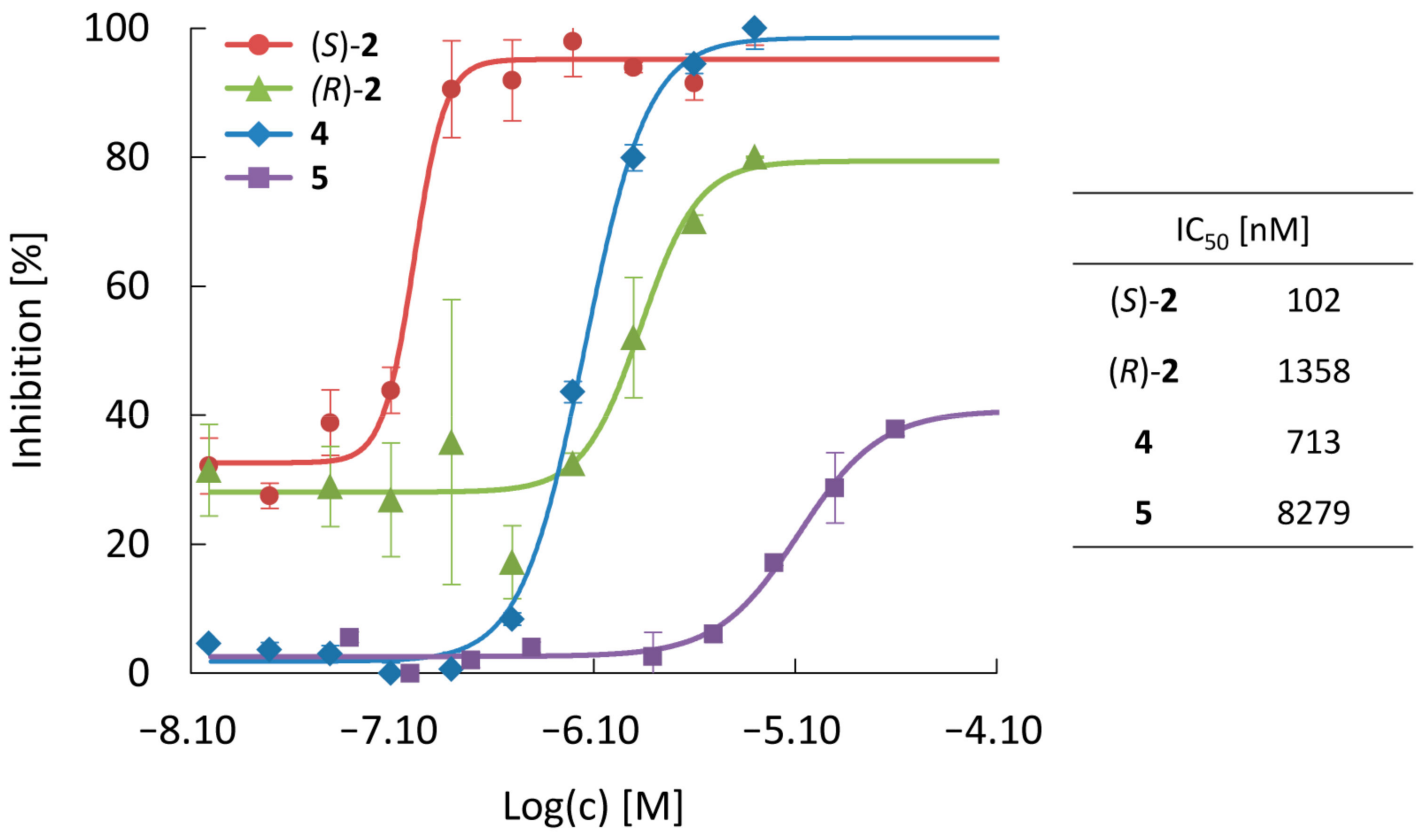
Dose-response curves and half-maximal inhibitory concentrations (IC_50_) for suppression of mIDH1_R132H_ by the fluorinated olutasidenib derivatives. Data are shown as mean ± standard deviation (n = 3). For additional fit parameters and 95% confidence intervals, see Table S12 in Section S4.1.4 of Supplementary Materials.

The inhibitory activity of the fluorinated olutasidenib derivatives against the most frequent IDH1 mutation in gliomas (mIDH1_R132H_) was evaluated by enzymatic assays with serially diluted solutions on the non-labeled compounds. Figure 3 shows dose-response curves derived from these experiments and the corresponding half-maximal inhibitory concentrations (IC_50_) for the different compounds. While all four compounds inhibited the activity of the mutant enzyme, their inhibitory potency differed markedly, with IC_50_ values ranging from nanomolar to micromolar concentrations. As expected based on the results of previous studies [30,31], replacement of the 6-chloro substituent in olutasidenib by a fluorine atom resulted in good retention of inhibitory potency (IC_50_ = 102 nM for (*S*)-**2**), while the corresponding (*R*)-methyl enantiomer was substantially less potent (IC_50_ = 1.4 µM for (*R*)-**2**). Replacement of the 6-chloro substituent in the early lead structure by fluorine resulted in a compound with intermediate inhibitory potency (IC_50_ = 713 nM for **4**), while additional replacement of the methoxy group at the cyanobenzene moiety by a fluoroethoxy group strongly increased the IC_50_ value (IC_50_ = 8.3 µM for **5**).

#### 2.2.4. Cellular Uptake and Localization

**Figure 4:**
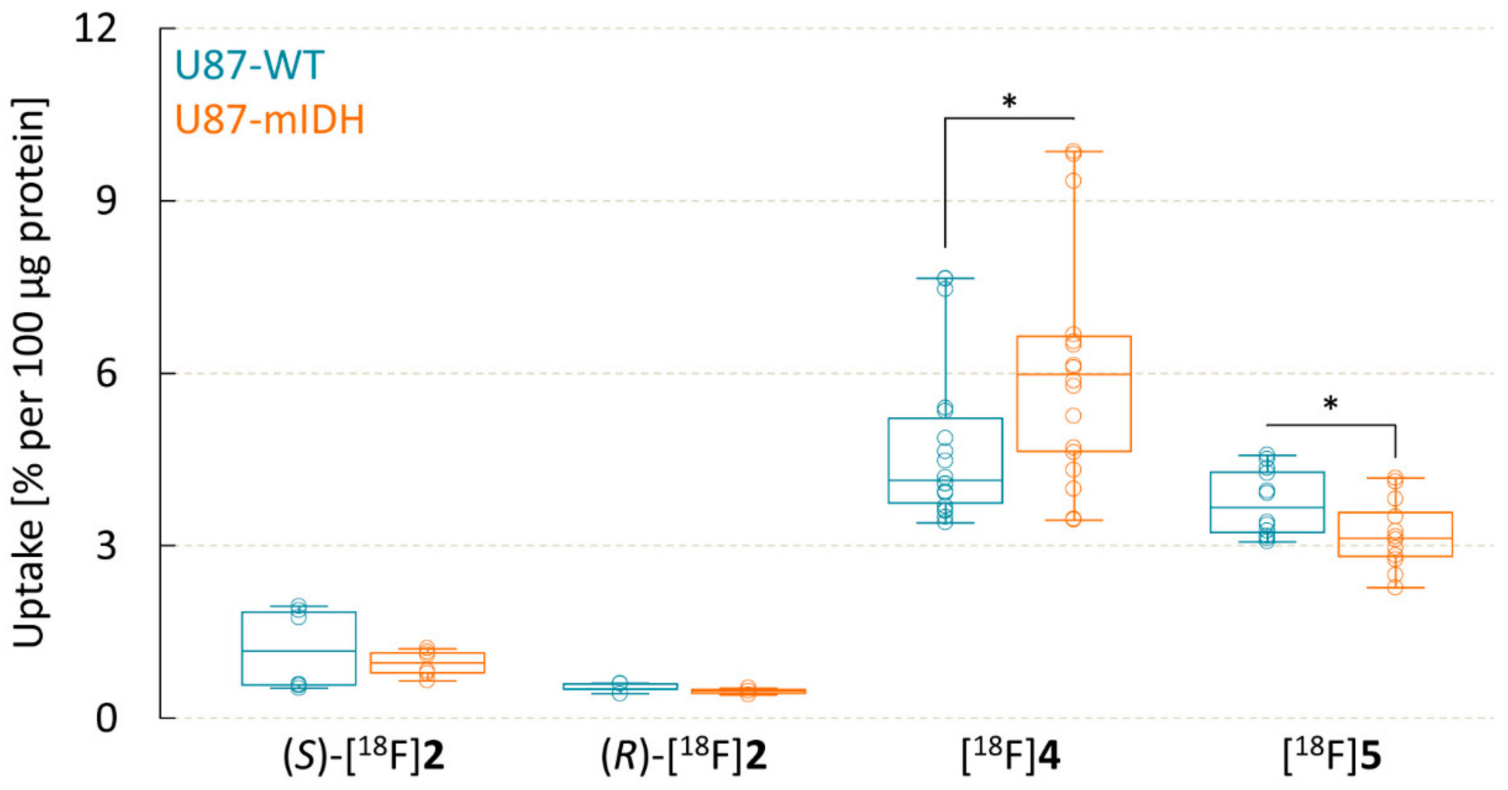
Uptake of ^18^F-labeled olutasidenib derivatives into wildtype (U87-WT) and IDH-mutated (U87-mIDH) U87 glioma cells after 60 min. Boxplots indicate median, 25th and 75th percentile (box), minimum and maximum values (whiskers) and individual data points (circles). Statistically significant differences (*p* < 0.05) in cellular uptake of the compounds between U87-WT and U87-mIDH cells were identified by a two-tailed *t*-test and are indicated by asterisks.

Next, cellular uptake of the ^18^F-labeled olutasidenib derivatives by U87 glioma cells with a heterozygous IDH1_R132H_ mutation (U87-mIDH) and the corresponding wildtype cells (U87-WT) was evaluated. Unexpectedly, the results revealed a very low cellular uptake of (*S*)-[^18^F]**2** after incubation for 1 h, without clear differences between IDH-mutated (0.9 ± 0.2% per 100 µg protein) and wildtype (1.2 ± 0.7% per 100 µg protein) cells (Figure 4). Moreover, despite the >10-fold higher IC_50_ value of the (*R*)-methyl enantiomer, cellular uptake of (*S*)-[^18^F]**2** by U87-mIDH cells was only approx. 2-fold higher than that of (*R*)-[^18^F]**2** (0.5 ± 0.1% per 100 µg protein), suggesting that inhibitor potency and cellular uptake were not directly related. Consistent with this assumption, uptake of [^18^F]**4** and, to a lesser extent, the ^18^F-fluoroethoxylated analog [^18^F]**5** by both cell lines was considerably higher than that of (*S*)-[^18^F]**2**. In addition, [^18^F]**4** showed a moderately but significantly higher uptake into U87-mIDH (6.0 ± 2.0% per 100 µg protein) compared to U87-WT (4.7 ± 1.4% per 100 µg protein, *p* = 0.03) cells. Conversely, ^18^F-fluoroethoxylated [^18^F]**5** preferentially accumulated in the wildtype cell line (3.7 ± 0.6% per 100 µg protein) and showed significantly lower uptake into U87-mIDH cells (3.2 ± 0.6% per 100 µg protein, *p* = 0.03). 

To assess whether the apparent contrast between inhibitory potency and cellular uptake could be related to differential membrane partitioning or subcellular distribution of the probes, their cellular localization was also analyzed by cell fractionation experiments after incubation with the different compounds. However, the results showed that all four compounds mainly accumulated in the cytoplasm, while the fraction of radioactivity associated with the cellular membranes and organelles was always below 5%.

Taken together, these results are in line with recent findings that mIDH-selective inhibitors may not necessarily exhibit mIDH-selective binding [48] and that their cellular uptake may be governed by factors not related to inhibitor affinity or potency [27]. Thus, while (*S*)-**2** demonstrated promising potency and selectivity in functional assays [31], cellular uptake of (*S*)-[^18^F]**2** was low and showed no difference between U87-mIDH and U87-WT cells. Conversely, **4** exhibited roughly 7-fold lower potency than (*S*)-**2** in functional assays (Figure 3), but cellular uptake of [^18^F]**4** was 5−8-fold higher than that of (*S*)-[^18^F]**2** (Figure 4). In addition, [^18^F]**4** was the only probe that showed preferential uptake into U87-mIDH cells, so that it was selected for further in vivo evaluation.

### 2.3. In Vivo Evaluation of [^18^F]mIDH-138 ([^18^F]**4**)

#### 2.3.1. Biodistribution Studies in Healthy C57BL/6 Mice

**Figure 5:**
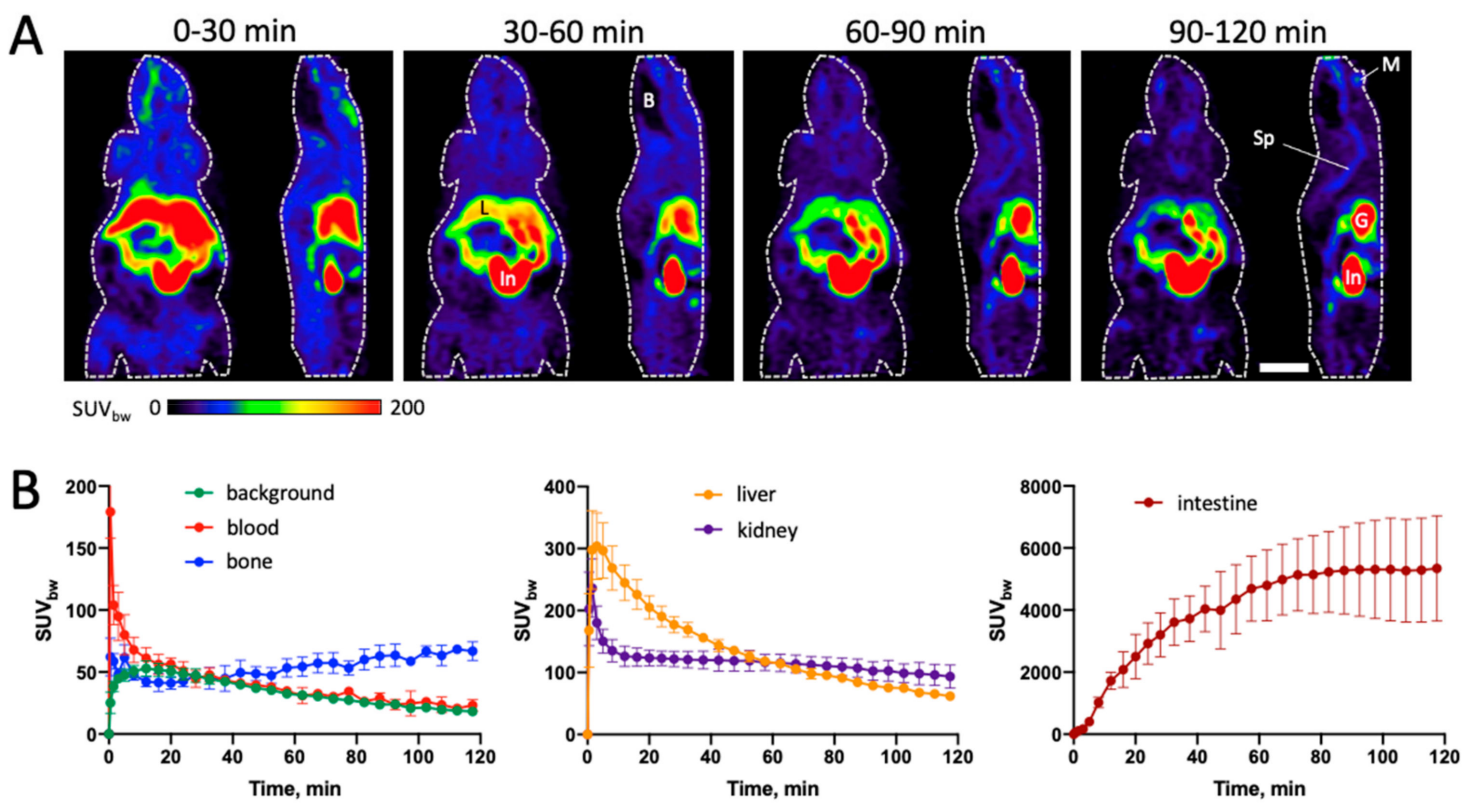
Biodistribution of [^18^F]**4** in healthy C57BL/6 mice. (**A**) Representative horizontal (left) and sagittal (right) summed PET images for the indicated 30 min timeframes after tracer injection. The body contour is indicated by a white dashed line. Scale bar: 10 mm. (**B**) Time activity curves for volumes of interest (VOIs) placed in the indicated organs, expressed as mean SUV_bw_ values ± standard deviation (n = 4). Abbreviations: B—brain, G—gall bladder, In—intestines, L—liver, M—mandible (lower jaw), Sp—spine.

The in vivo stability, whole-body biodistribution and excretory pathways of [^18^F]**4** were investigated by small animal PET imaging in healthy C57BL/6 mice (Figure 5). The results demonstrated almost exclusive hepatobiliary excretion of the radiotracer, with high uptake in liver, gall bladder and intestine. Liver uptake showed an early peak (max. SUV_bw_ 304 ± 54 after 3 min) followed by a fast washout, while intestinal radioactivity increased steadily and reached maximum values at the end of the measurements (max. SUV_bw_ 5344 ± 1693 after 120 min). In contrast, renal excretion was negligible, with very low radioactivity accumulation in the urinary bladder. Background uptake into muscles and other tissues was also low, with maximum SUV_bw_ values of 52 ± 6 after 16 min followed by a decline to 18 ± 2 after 120 min. Bone uptake of radioactivity was relatively low as well but increased slightly during the measurements and reached SUV_bw_ values of 67 ± 8 after 120 min (Figure 5), indicating that the radiotracer underwent moderate defluorination. Finally, brain uptake of radioactivity throughout all measurements remained very low, suggesting limited transfer of [^18^F]**4** across the intact blood–brain barrier (BBB). Nevertheless, while limited BBB penetrance might be an issue in patients with an intact BBB, brain tumors like gliomas are often associated with partial BBB disruption, which could facilitate tumor delivery of the probe. In addition, mutations of IDH1 have also been detected in certain solid tumors outside of the brain [49,50,51,52,53], suggesting that mIDH1-selective PET tracers could also be used for peripheral tumor imaging. With this in mind and based on the reasonable in vivo stability and low background uptake, the tumor accumulation and mIDH-selectivity of [^18^F]**4** were further investigated in a subcutaneous tumor model.

#### 2.3.2. Biodistribution Studies in CB17-SCID Mice with Subcutaneous U87-WT and U87-mIDH Tumors

**Figure 6:**
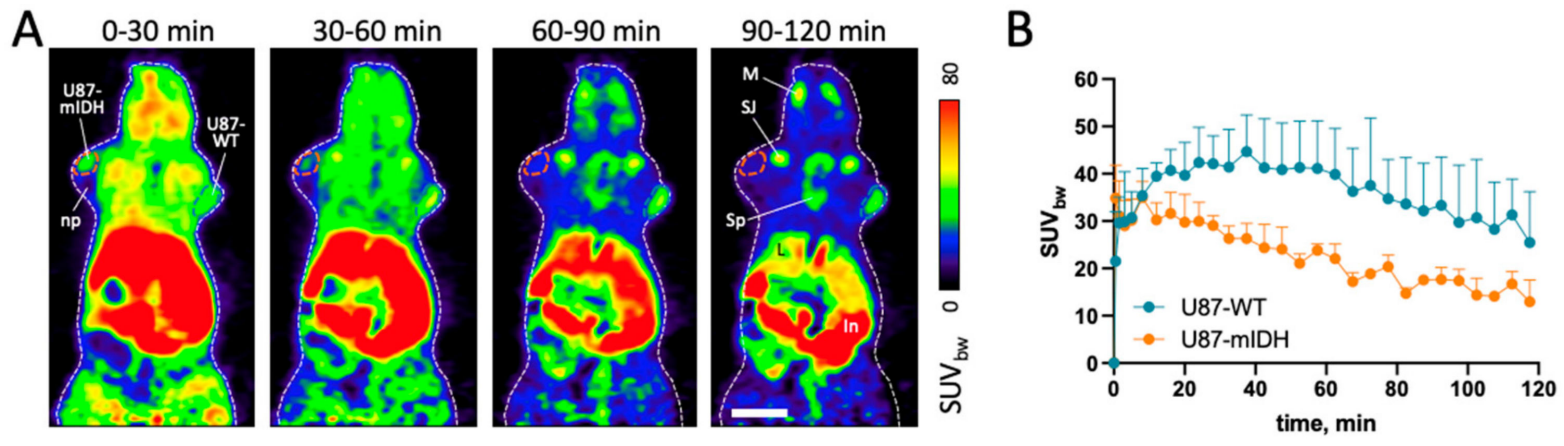
Biodistribution of [^18^F]**4** in CB17-SCID mice with subcutaneous U87-mIDH and U87-WT tumors. (**A**) Representative horizontal summed PET images for the indicated 30 min timeframes after tracer injection. The body contour is indicated by a white dashed line. The U87-mIDH tumor was located in the left shoulder region (indicated by the orange dashed line), while the U87-WT tumor was located in the right shoulder region (indicated by the blue dashed line). Scale bar: 10 mm. (**B**) Time activity curves for volumes of interest (VOIs) placed in the non-necrotic parts of the tumors, expressed as mean SUV_bw_ values ± standard deviation (n = 3). Abbreviations: In—intestines, L—liver, M—mandible (lower jaw), np—necrotic part of the tumor, SJ—shoulder joint, Sp—spine.

The in vivo tumor uptake and mIDH selectivity of [^18^F]**4** were investigated by small animal PET imaging in CB17-SCID mice bearing subcutaneous U87-WT and U87-mIDH tumors, which were implanted in the right and left shoulder region, respectively (Figure 6). Due to premature death of three mice during the 120 min measurements (after 65, 80 and 95 min), full time activity curves (TACs) could only be calculated for the remaining three animals, while data from a total of five mice could be used for kinetic modelling (see below). Unexpectedly, analysis of the PET measurements revealed more pronounced uptake of [^18^F]**4** into U87-WT compared to U87-mIDH tumors. Thus, while initial tracer uptake by both tumors was similar, accumulation of radioactivity in the U87-mIDH tumors already peaked after 8 mins (max. SUV_bw_ 35 ± 8), which was followed by a quick washout (Figure 6B). In contrast, the U87-WT tumors showed a sustained accumulation phase (max. SUV_bw_ 45 ± 8 after 38 min) and better retention of radioactivity (Figure 6B), which resulted in almost two-fold higher SUV_bw_ values during the second half of the measurements (Table 2). Interestingly, very similar differences in the time course of tracer uptake (i.e., early peak followed by fast washout in IDH1 mutated tumors versus sustained tracer accumulation in IDH1 wildtype tumors) have previously been observed in a preclinical study with the TSPO-radioligand [^18^F]DPA-714 [22], suggesting that they could be related to factors like, e.g., differences in tracer perfusion between the tumors. Indeed, considering that expression of mIDH is well known to have profound effects on numerous aspects of tumor biology and growth [54,55,56,57,58], it seemed conceivable that differences in the composition and/or structure of the tumors (percentage and distribution of hypoxic, necrotic, etc., cells) could result in genotype-dependent differences in the wash-in/wash-out phases, although visual inspection of the dissected tumors did not substantiate this hypothesis.

To obtain further insight into the potential mechanisms underlying these unexpected findings, tumoral tracer uptake was analyzed with a two-tissue compartment model (2TCM), using radioactivity in the heart as image-derived input function. Although the use of an image-derived input function instead of an invasive arterial input function could introduce some degree of systematic bias (due to, e.g., a lack of correction for spill-over and metabolism), this should affect the parameter estimation for both tumors in a given animal in the same manner and therefore have little impact on inter-tumor comparisons. The results of the kinetic modelling showed that both specific distribution volume (V_S_) and non-displaceable binding potential (BP_ND_) were significantly higher for U87-WT compared to U87-mIDH tumors, whereas the influx and efflux rate constants (K_1_, k_2_) for the two tumor types were comparable (Table 3). These results indicate that the higher tracer uptake in U87-WT tumors resulted from higher specific binding rather than from differences in tracer perfusion or wash-in/wash-out phases. In particular, V_S_ and BP_ND_ depend on the density of receptors available for radioligand binding (B_avail_), the affinity of the radioligand (K_D_) and its free fraction (f_ND_) [59], suggesting that the differences in tracer uptake were related to differences in one or more of these parameters between the two tumor types. Assuming that nonspecific binding of the radioligand in both tumors was similar, differences in f_ND_ can most likely be excluded. Likewise, although recent findings indicate that most mIDH1-selective inhibitors can indeed bind to both wildtype and mutant enzymes [27,48], a significantly lower K_D_ of the radiotracer for the wildtype enzyme seems unlikely and should have been evident in the in vitro studies. Thus, while our observation that inhibitor potency and cellular uptake are not directly related clearly supports the assumption that mIDH1-selective inhibition may not correlate with mIDH1-selective binding, the preferential in vitro uptake of [^18^F]**4** into mIDH1-expressing cells argues against a higher affinity of this radioligand for the wildtype enzyme (but see below). On the other hand, the expression and function of mIDH1 have been shown to be affected by various intracellular and extracellular factors [58], which means that differences in B_avail_ between the tumors could potentially explain the apparent contrast between our in vitro and in vivo findings. For example, while expression of the mutated enzyme in the isogenic U87-mIDH cell line under well-defined in vitro conditions should be comparable to that of the wildtype enzyme in the parental U87-WT cell line, it seems conceivable that environmental stimuli in the tumor microenvironment could differentially affect the expression levels of the two enzymes in vivo. In addition, olutasidenib and most other mIDH1-selective inhibitors are thought to preferentially or exclusively bind to resting enzyme conformations [23] so that differences in the equilibrium between active and inactive states could affect the number of enzymes available for binding. Indeed, a recent study with the mIDH1-selective radioligand [^18^F]AG-120 reported significant differences in the apparent number of binding sites between cells expressing mutated and wildtype IDH1, despite similar expression levels of both enzymes [27]. In particular, while [^18^F]AG-120 showed comparable affinities for both enzymes, the number of binding sites and cellular tracer accumulation in mIDH1-expressing cells were much higher, suggesting that a larger fraction of enzymes in these cells was in the binding-permissive resting conformation. Based on these findings and considering that AG-120 and olutasidenib have been shown to target the same induced-fit pocket [23], the preferential in vitro uptake of [^18^F]**4** by U87-mIDH cells observed in the present study could be explained not only by a higher affinity of the radioligand for the mutated enzyme, but also by a higher fraction of resting enzymes in the U87-mIDH cells. Since the equilibrium between active and inactive states is well known to be highly dynamic and sensitive to many factors like the concentration of substrates, co-factors and catalytic metal ions [60,61,62], the latter could in turn provide an attractive explanation for the opposite results observed in vivo. Thus, preferential uptake into the wildtype tumors could result if the exact conformational ensemble in vivo is different from that in the in vitro assays and, e.g., a larger fraction of the enzymes in these tumors resides in the inactive conformation. In any case, while further studies are required to firmly establish the underlying mechanisms, our findings demonstrate that in vivo engagement of wildtype and mutated IDH1 is governed by factors that are not faithfully reproduced by in vitro assay.

## 3. Materials and Methods

### 3.1. Chemistry and Radiochemistry

A detailed description of all chemical and radiochemical procedures is provided in Supplementary Materials.

### 3.2. Cell Culture and In Vitro Studies

#### 3.2.1. Cell Culture and Reagents

Human glioblastoma cells with a c.395G>A knock-in mutation encoding mIDH1_R132H_ (HTB-14IG™) and the corresponding wildtype cells (HTB-14™) were purchased from the American Type Culture Collection (ATCC). The cells were grown under standard culture conditions (5% CO_2_ and 95% air at 37 °C) in Eagle’s Minimum Essential Medium (MEM) supplemented with 10% fetal bovine serum (FBS) and antibiotics (penicillin–streptomycin mixed solution, Gibco, Thermo Fisher Scientific, Oberhausen, Germany). The identity of the cell lines and functional expression of the mutated enzyme were confirmed by measurement of 2-HG production (for details see below), which demonstrated 3.6-fold higher levels in the HTB-14IG™ (7.56 ± 1.25 nmol per 10^6^ cells) compared to the parental HTB-14™ (2.08 ± 0.83 nmol per 10^6^ cells) cell line.

#### 3.2.2. 2-Hydroxyglutarate (2-HG) Assays

Cellular 2-HG production was measured with a commercially available assay kit (Abcam, Cambridge, UK, ab211070). To this end, the cells were cultured in 9 cm dishes as described above until they had reached about 90% confluency. The cells were then detached with trypsin, suspended in 1.6 mL MEM, counted, and centrifuged for 5 min (1000× *g*) at ambient temperature. The resulting cell pellet was resuspended in 100 µL assay buffer and the cell membranes were disrupted by ultrasonication for 1 min on ice. After centrifugation (50,000× *g*) for 5 min at 4 °C, the supernatant was collected and used for determination of the 2-HG concentration according to the manufacturer specifications.

#### 3.2.3. Determination of Lipophilicity

A solution of the corresponding radiotracer in DMSO (1 µL) was added to a mixture of *n*-octanol and PBS (pH = 7.4) or water (750 µL of each) in a 2 mL Eppendorf vial and the vial was vortexed for 2 min and centrifuged for another 2 min to separate the two phases. A total of 300 µL of the upper *n*-octanol phase was then carefully removed from the top, while 300 µL of the aqueous phase was removed by piercing the bottom of the vial with a syringe and cannula to avoid contact with the organic phase. Both fractions were transferred into fresh vials and counted in a gamma counter to determine the ratio between the activity in the organic and the aqueous phase. All measurements were performed in triplicate and the results are expressed as mean ± standard deviation of the logarithm of the partition coefficient between *n*-octanol and water (logP) or the logarithm of the distribution coefficient between *n*-octanol and PBS (logD_7.4_), respectively.

#### 3.2.4. Determination of Stability in DMSO

A solution of the corresponding reference compound in DMSO (1 mg/mL) was incubated at room temperature for 28–30 h, during which aliquots were removed every few hours and analyzed by analytical HPLC. The fraction of intact reference compound at each time point was calculated by dividing the peak area of the reference compound by the total area of all peaks in the chromatogram and plotted as a function of time.

#### 3.2.5. Determination of Stability in PBS

A solution of the corresponding radiotracer (approximately 0.5–1.0 MBq) in 0.5 µL DMSO was added to 0.5 mL PBS and the mixture was incubated at room temperature for three hours. After 0, 1, 2 and 3 h, 2.5 µL aliquots of the solution were removed and spotted on a radio TLC plate, which was air-dried and developed with an appropriate eluent for the respective tracer. For determination of the amount of intact tracer, the TLC plate was scanned with a phosphor imager and all peaks were integrated and baseline subtracted. The radiotracers were identified by comparison of the migration distances with the non-radioactive reference compounds. If the TLC plates were developed after collection of all samples, partial or complete defluorination was detected for the samples collected during early time-points but not for the last sample (see Figure S18A,B in Section S4.1.2 of Supplementary Materials), indicating silica-induced degradation of the tracers. If the aliquots for each time-point were spotted on separate TLC plates that were immediately developed to avoid silica-induced tracer degradation, no evidence for defluorination was observed for any of the time-points examined (see Figure S18C,D in Section S4.1.2 of Supplementary Materials). 

#### 3.2.6. Determination of Stability in Rat Serum

A vial with 0.5 mL of rat serum was prewarmed in a thermoshaker at 37 °C for 5 min before a solution of the corresponding radiotracer (approximately 0.5–1.0 MBq) in 0.5 µL DMSO was added. The mixture was shaken for 1 h at 37 °C, during which 60 µL aliquots were removed after 5, 15, 30 and 60 min (n = 3 per time-point). Each aliquot was added to a vial with 120 µL MeCN and the vials were vortexed for 2 min and centrifuged for another 2 min. Then, 2.5 µL of the supernatants and the respective reference compound were spotted on separate lanes of a radio TLC plate and the plate was developed with a suitable eluent for the respective tracer. Baseline, frontline and the reference compound peak were then detected with a UV lamp and spotted with activity to be visualized with a phosphor imager. The TLC plate was scanned at the phosphor imager, all peaks were integrated and the background was subtracted. The radiotracers were identified by comparison of the migration distance of the radioactive spots with the non-radioactive reference compounds. 

#### 3.2.7. Determination of Inhibitory Potency

The inhibitory activity of the candidate probes against mIDH1 was evaluated by enzymatic assays with serially diluted solutions of the non-labeled compounds using a commercially available assay kit (IDH1 (R132H) from BPS Bioscience, San Diego, CA, USA). IC_50_ values were determined with GraphPad Prism 4.00 by fitting the resulting dose-response curves with the Hill function.

#### 3.2.8. Cellular Uptake Studies

For the cellular uptake studies, 1.5 × 10^5^ cells were seeded into gelatin-coated 12-well transwell plates and incubated in 1 mL MEM (without FBS and antibiotics) containing 18.5 kBq (0.5 µCi) of the corresponding radiotracer. After incubation at 21 °C for 1 h, the supernatant was collected, the cells were lysed with 40 mM NaOH and the radioactivity of both fractions was measured with a gamma counter. The total protein concentration of the cell lysate was measured by a bicinchoninic acid (BCA) assay. The radiotracer uptake was calculated as the ratio of radioactivity in the cell fraction and the supernatant and normalized by protein content (% uptake/100 µg protein). Statistical analysis of the results was performed with Originlab Pro 2023b using two-tailed *t*-tests with a significance level of 0.05 to identify significant differences between groups.

To assess the ability of the radiotracers to cross the cell membrane and enter the intracellular compartment, separate cell fractionation experiments were conducted after incubation with the corresponding radiotracer as described above. To this end, the cells were detached with trypsin and dispersed with an ultra-turrax (1 min at maximum speed on ice). After centrifugation (50,000× *g*) for 5 min at 4 °C, the supernatant containing the cell plasma was separated from the pellet containing the cell membranes, organelles, and nuclei. The radioactivity of both fractions was measured with a gamma counter and the percentage of radiotracer in the cell plasma was calculated from the activity ratio.

### 3.3. In Vivo Studies

#### 3.3.1. Animals

All experiments were carried out in accordance with the EU directive 2010/63/EU for animal experiments and the German Animal Welfare Act (TierSchG, 2006) and were approved by the regional authorities (LANUV NRW; 81-02.04.2022.A442). Six male CB17-SCID mice and four male C57BL/6 mice (Janvier Labs, Le Genest-Saint-Isle, France) were housed in groups in individually ventilated cages (Allentown LLC, Allentown, NJ, USA) in a temperature- and humidity-controlled room (20 ± 2 °C, 50–60% humidity) on a 12:12 h light–dark cycle. Throughout the experiments, the animals had ad libitum access to water and food.

#### 3.3.2. Subcutaneous Tumor Model

Six male CB17-SCID mice (24–26 g) were used for the subcutaneous tumor model. Twenty-four hours before tumor implantation, the animals were injected (i.p.) with 20 µL anti-asialo GM1 rabbit (Fujifilm Wako Chemicals Europe GmbH, Neuss, Germany) in 0.9% NaCl (total volume: 100 µL) to promote tumor cell survival and growth through a temporary reduction in natural killer cell activity. For tumor inoculation, suspensions of 5 × 10^6^ tumor cells in 75 µL culture medium and 75 µL Corning Matrigel (Merck, Darmstadt, Germany) were injected subcutaneously into the right (U87-WT cells) and left (U87-mIDH cells) shoulder region. Due to faster growth of the U87-mIDH tumors, U87-WT cells were injected five days before the U87-mIDH cells.

#### 3.3.3. PET Measurements

The mice were anesthetized with isoflurane (5% for induction, 2% for maintenance) in O_2_/air (3:7), and a catheter for tracer injection was inserted into the lateral tail vein. They were placed on an animal holder (Medres, Cologne, Germany) and fixed with a tooth bar in a respiratory mask. Body temperature was maintained at 37 °C by warming the animal bed. Eyes were protected from drying with Bepanthen eye and nose ointment (Bayer, Leverkusen, Germany). Respiratory rate was monitored and maintained at around 40–60 breaths per minute by adjusting the isoflurane concentration. A PET scan in list mode was conducted using a Focus 220 micro PET scanner (CTI-Siemens, Erlangen, Germany) with a resolution at the center of the field of view of 1.4 mm. Data acquisition started with intravenous tracer injection and ended after 120 min. This was followed by a 10 min transmission scan using a ^57^Co point source for attenuation correction. After the scan was completed, the catheter was removed and the animals were allowed to wake up in their home cage. Data were histogrammed in three ways: (1) 4 × 30 min frames for visual display, (2) 28 frames (2 × 1 min, 2 × 2 min, 6 × 4 min, 18 × 5 min) for determination of time–activity curves, (3) 53 frames (12 × 10 s, 6 × 30 s, 15 × 1 min, 20 × 5 min) for kinetic modelling. Full 3D rebinning was followed by an iterative OSEM3D/MAP reconstruction algorithm with attenuation and decay correction. The resulting voxel sizes were 0.47 mm × 0.47 mm × 0.80 mm. All further analysis was performed with the software VINCI 5.21 (Max Planck Institute for Metabolism Research, Cologne, Germany). Standardized uptake values based on body weight (SUV_bw_) were determined according to the following equation: SUV_bw_ = radioactivity [Bq/g] × body weight [g] × 100/injected dose [Bq]. Statistical analysis of the results was performed with a 2-way analysis of variance (ANOVA) followed by Sidak’s multiple comparison test to identify significant differences between the two tumor types.

#### 3.3.4. Kinetic Modelling

Kinetic modeling of the PET data was performed with PMOD V.4.403 (PMOD Technologies GmbH, Zürich, Switzerland). Time–activity curves (TACs) were generated from volumes of interest (VOIs) drawn around the tumors and the heart. The heart TAC served as image-derived input function for kinetic modeling. Using a two-tissue compartment model (2TCM) with the blood volume fraction (v_B_) constrained to 0.05, the kinetic rate constants K_1_ to k_4_ as well as V_T_, V_S_ and BP_ND_ (k_3_/k_4_) were estimated for both tumors. The choice of the specific model was based on both goodness of fit (AIC) and fitting robustness (standard deviation, parameter stability). Statistical analysis of the results was performed with Originlab Pro 2023b, using two-tailed *t*-tests with a significance level of 0.05 to identify significant differences between the two tumor types.

## 4. Conclusions

In conclusion, our in vitro studies with four ^18^F-labeled derivatives of olutasidenib corroborate recent findings that mIDH1-selective inhibition may not directly correlate with mIDH1-selective target engagement. In addition, biodistribution studies with [^18^F]**4** demonstrated that in vivo engagement of wildtype and mutated IDH1 by this radioligand is governed by factors that are not faithfully reproduced by in vitro assays. Taken together, these findings highlight several obstacles that could complicate preclinical development of mIDH1-selective PET tracers. For example, given the discrepancies between mIDH1-selective inhibition, in vitro uptake and in vivo engagement observed in the present and previous studies, it remains to be firmly established whether structural modification of existing mIDH1-selective ligands through medicinal chemistry approaches can be used to achieve selective target engagement in vivo. Furthermore, as target engagement could be influenced by environmental factors, more sophisticated animal models, such as patient-derived xenografts that closely mimic human glioma pathology, may be required to reliably assess a tracer’s in vivo performance. Future research utilizing such models will be crucial to determine the suitability of existing mIDH1-selective inhibitors as leads for development of PET probes as well as to identify potential structural modifications that could be used to improve in vivo target engagement.

## Figures and Tables

**Table 1:** Summary of experimental and calculated lipophilicity metrics.

Tracer	logP	logD_7.4_	
		Exp. Determined	ADMETlab
(*S*)-[^18^F]mIDH-23 [(*S*)-[^18^F]**2**]	negative	2.51 ± 0.11	2.33
(*R*)-[^18^F]mIDH-23 [(*R*)-[^18^F]**2**]	negative	2.51 ± 0.11	2.33
[^18^F]mIDH-138 ([^18^F]**4**)	2.50 ± 0.03	2.67 ± 0.04	2.75
[^18^F]FE-mIDH-138 ([^18^F]**5**)	2.77 ± 0.03	2.72 ± 0.06	2.87

**Table 2:** Comparison of [^18^F]**4** uptake between U87-WT and U87-mIDH tumors.

Timeframe	SUV_bw_		Ratio (WT/mIDH)	Significance ^1^
	U87-WT	U87-mIDH		
0–30 min	35 ± 4	31 ± 3	1.1	*p* = 0.5810
30–60 min	42 ± 9	24 ± 2	1.7	***p* = 0.0031**
60–90 min	36 ± 10	18 ± 1	1.9	***p* = 0.0033**
90–120 min	30 ± 10	16 ± 1	1.9	***p* = 0.0087**

^1^ Two-way ANOVA main effects: Factor timeframe: F(3,6) = 19.98, *p* = 0.0016; factor tumor type: F(1,2) = 8.04, *p* = 0.1051; factor interaction: F(3,6) = 5.11, *p* = 0.0433. Shown are results of Sidak’s multiple comparison test, with significant differences indicated in bold.

**Table 3:** Micro- and macro-parameters obtained via kinetic modeling of the PET data (2TCM) ^1^.

Parameter	U87-WT Tumor	U87-mIDH Tumor	Significance
K_1_ (mL/ccm/min)	0.174 ± 0.068	0.141 ± 0.047	*p* = 0.401
k_2_ (1/min)	0.553 ± 0.238	0.578 ± 0.173	*p* = 0.858
k_3_ (1/min)	0.089 ± 0.059	0.057 ± 0.040	*p* = 0.343
k_4_ (1/min)	0.044 ± 0.022	0.053 ± 0.023	*p* = 0.525
V_S_ (mL/ccm)	0.576 ± 0.143	0.225 ± 0.070	***p* = 0.001**
V_T_ (mL/ccm)	0.921 ± 0.250	0.471 ± 0.084	***p* = 0.005**
K_1_/k_2_ (mL/ccm)	0.345 ± 0.172	0.246 ± 0.063	*p* = 0.261
k_3_/k_4_, BP_ND_ (1/1)	1.874 ± 0.653	0.962 ± 0.399	***p* = 0.029**

^1^ Results are given as mean ± standard deviation (n = 5). Statistically significant differences (*p* < 0.05) between U87-WT and U87-mIDH tumors were identified by a two-tailed *t*-test and are indicated in bold. Abbreviations: K_1_–k_4_: rate constants of the 2TCM; V_S_: specific distribution volume; V_T_: total distribution volume; BP_ND_ (=k_3_/k_4_): non-displaceable binding potential.

## Data Availability

The raw data supporting the conclusions of this article will be made available by the authors on request.

## Supplementary Materials

The following supporting information can be downloaded at: https://www.mdpi.com/article/10.3390/molecules29163939/s1, Detailed description of all chemical and radiochemical procedures including HPLC chromatograms of the radiolabeled compounds (Sections S1–S3); additional results of the preclinical in vitro evaluation (Section S4); ^1^H-, ^13^C and ^19^F-NMR spectra of all prepared compounds (Section S5).
