# Supplementary material for: Preparation and Preclinical Evaluation of 18F-Labeled Olutasidenib Derivatives for Non-Invasive Detection of Mutated Isocitrate Dehydrogenase 1 (mIDH1)
Source: Molecules. 2024 Aug 21;29(16):3939. doi: 10.3390/molecules29163939 (PMC11356819; doi:10.3390/molecules29163939)

Supporting Information for

Preparation and Preclinical Evaluation of ^{18}F-Labeled Olutasidenib Derivatives for Non-Invasive Detection of Mutated Isocitrate Dehydrogenase 1 (mIDH1)

Roberta Cologni [1,2], Marcus Holschbach [1], Daniela Schneider[1], Dirk Bier [1], Annette Schulze [1], Carina Stegmayr [3], Heike Endepols [1,2,4], Johannes Ermert [1], Felix Neumaier [1,2] and Bernd Neumaier [1,2*]

[1] Institute of Neuroscience and Medicine, Nuclear Chemistry (INM-5), Forschungszentrum Jülich GmbH, Wilhelm-Johnen-Straße, 52428 Jülich, Germany

[2] Institute of Radiochemistry and Experimental Molecular Imaging, Faculty of Medicine and University Hospital Cologne, University of Cologne, Kerpener Straße 62, 50937 Cologne, Germany

[3] Institute of Neuroscience and Medicine, Medical Imaging Physics (INM-4), Forschungszentrum Jülich GmbH, Wilhelm-Johnen-Straße, 52428 Jülich, Germany

[4] Department of Nuclear Medicine, Faculty of Medicine and University Hospital Cologne, University of Cologne, Kerpener Straße 62, 50937 Cologne, Germany

* corresponding author: b.neumaier@fz-juelich.de

Table of contents

1. General information ***4***

1.1. Solvents and reagents **4**

1.2. Spectroscopy **4**

1.3. Chromatography **4**

2. Organic preparative syntheses ***6***

2.1 Preparation of precursors 14, 21 and 22, reference compound 4 and protodeboronated impurity 13c **6**

2.2 Preparation of *N*-protected precursors (*S*)- or (*R*)-29, *O*-protected precursors (*S*)- or (*R*)-30, reference compounds (*S*)- or (*R*)-2 and protodeboronated impurities (*S*)- or (*R*)-26c **20**

2.2.3 Preparation of *O*-protected precursor 39 and reference compound 5. ..... **37**

3. Radiochemistry ***44***

3.1 General Conditions **44**

3.2. Analytical high-performance liquid chromatography (HPLC) **44**

3.3. Semipreparative HPLC **45**

3.4. Radiosynthesis of [^{18}F]4 ..... **46**

3.4.1. Copper-mediated radiofluorination of non-protected precursor **14** 46

3.4.2. Spiking experiment 46

3.4.3. Copper mediated radiofluorination of POM-protected precursors **21** and **22** 49

3.4.4. Determination of carrier amount and molar activity 52

3.5. Radiosynthesis of (*S*)-[^{18}F]2 and (*R*)-[^{18}F]2 ..... **54**

3.5.1. Copper-mediated radiofluorination of (*S*)- and (*R*)-**29** and (*S*)- and (*R*)-**30** 54
3.5.2. Determination of carrier amount and molar activity.. 58
3.5.3. Determination of absolute configuration 58

3.6. Radiosynthesis of [^{18}F]5 61
3.6.1. Aliphatic radiofluorination of precursor **39** 61
3.6.2 Determination of carrier amount and molar activity.. 67

4. Preclinical evaluation.. 68

4.1 *In vitro* stability and inhibitory potency 68
4.1.1. *In vitro* stability in DMSO 68
4.1.2. *In vitro* stability in phosphate-buffered saline (PBS).. 69
4.1.3. *In vitro* stability in rat serum 70
4.1.4. Inhibitory potency (IC_{50}) 71

5. NMR Spectra 72
5.1. 4-Bromoacetanilide (**7a**)... 72
5.2. 4-Fluoroacetanilide (**7b**) 73
5.3. 6-Bromo-2-chloroquinoline-3-carbaldehyde (**10a**).. 74
5.4. 2-Chloroquinoline-3-carbaldehyde (10c).. 75
5.5. *N*-(4-Bromophenyl)-2-formyl-3-hydroxyacrylamide (**11a**)... 76
5.6. *N*-(4-Fluorophenyl)-2-formyl-3-hydroxyacrylamide (**11b**) 77
5.7. 6-Bromo-2-oxo-1,2-dihydroquinoline-3-carbaldehyde (**12a**)... 79
5.8. 6-Fluoro-2-oxo-1,2-dihydroquinoline-3-carbaldehyde (**12b**) 80
5.9. 2-Oxo-1,2-dihydroquinoline-3-carbaldehyde (**12c**)... 81
5.10. 4-{[(6-Bromo-2-oxo-1,2-dihydroquinolin-3-yl)methyl]amino}-2-methoxybenzonitrile (**13a**).......... 82
5.11. 4-{[(6-Fluoro-2-oxo-1,2-dihydroquinolin-3-yl)methyl]amino}-2-methoxybenzonitrile (**4**) 84
5.12. 2-Methoxy-4-(((2-oxo-1,2-dihydroquinolin-3-yl)methyl)amino)benzonitrile (**13c**) 85
5.13. 2-Methoxy-4-{[(2-oxo-6-(4,4,5,5-tetramethyl-1,3,2-dioxaborolan-2-yl)-1,2-dihydroquinolin-3-yl)methyl]amino}benzonitrile (**14**)... 86
5.14. 4-Amino-2-methoxybenzonitrile (**16**).. 88
5.15. (6-Bromo-3-formyl-2-oxoquinolin-1(2*H*)-yl)methyl pivalate (**17a**) 89
5.16. (6-Fluoro-3-formyl-2-oxoquinolin-1(2*H*)-yl)methyl pivalate (**17b**)... 90
5.17. [(6-Bromo-3-formylquinolin-2-yl)oxy]methyl pivalate (**18a**)... 91
5.18. [(6-Fluoro-3-formylquinolin-2-yl)oxy]methyl pivalate (**18b**) 92
5.19. (3-Formyl-2-oxoquinolin-1(2*H*)-yl)methyl pivalate (**17c**) and [(3-formylquinolin-2-yl)oxy]methyl pivalate (**18c**).. 94
5.20. 6-Bromo-1-ethyl-2-oxo-1,2-dihydroquinoline-3-carbaldehyde (**S1a**)... 95
5.21. 6-Bromo-2-ethoxyquinoline-3-carbaldehyde (**S1b**) 96
5.22. (6-Bromo-3-{[(4-cyano-3-methoxyphenyl)amino]methyl}-2-oxoquinolin-1(2*H*)-yl)methyl pivalate (**19a**) 97
5.23. [(6-Bromo-3-[{(4-cyano-3-methoxyphenyl)amino}methyl]quinolin-2-yl)oxy]methyl pivalate (**20a**)98
5.24. (3-[{(4-Cyano-3-methoxyphenyl)amino}methyl]-2-oxoquinolin-1(2*H*)-yl)methyl pivalate (**19c**).. 100
5.25. [(3-[{(4-Cyano-3-methoxyphenyl)amino}methyl]quinolin-2-yl)oxy]methyl pivalate (**20c**)............ 101
5.26. (3-[{(4-Cyano-3-methoxyphenyl)amino}methyl]-2-oxo-6-(4,4,5,5-tetramethyl-1,3,2-dioxaborolan-2-yl)quinoline-1(2*H*)-yl)methyl pivalate (**21**)... 102
5.27. [(3-[{(4-Cyano-3-methoxyphenyl)amino}methyl]-6-(4,4,5,5-tetramethyl-1,3,2-dioxaborolan-2-yl)quinolin-2-yl)oxy]methyl pivalate (**22**).. 103
5.28. (*R*)-*N*-[(6-bromo-2-chloroquinolin-3-yl)methylene]-2-methylpropane-2-sulfinamide (**23a**)........... 104
5.29. (*R*)-*N*-[(6-Fluoro-2-chloroquinolin-3-yl)methylene]-2-methylpropane-2-sulfinamide (**23b**)........... 105
5.30. (*R*)-*N*-[(2-chloroquinolin-3-yl)methylene]-2-methylpropane-2-sulfinamide (**23c**) 106
5.31. (*R*)-*N*-[(*S*)-1-(6-Bromo-2-chloroquinolin-3-yl)ethyl]-2-methylpropane-2-sulfinamide [(*S*,*R*)-**24a**] 107
5.32. (*R*)-*N*-[(*R*)-1-(6-Bromo-2-chloroquinolin-3-yl)ethyl]-2-methylpropane-2-sulfinamide [(*R*,*R*)-**24a**] 108
5.33. (*R*)-*N*-[(*S*)-1-(6-Fluoro-2-chloroquinolin-3-yl)ethyl]-2-methylpropane-2-sulfinamide [(*S*,*R*)-**24b**]. 109
5.34. (*R*)-*N*-[(*R*)-1-(6-Fluoro-2-chloroquinolin-3-yl)ethyl]-2-methylpropane-2-sulfinamide [(*R*,*R*)-**24b**] 111
5.35. (*R*)-*N*-[(*S*)-1-(2-Chloroquinolin-3-yl)ethyl]-2-methylpropane-2-sulfinamide [(*S*,*R*)-**24c**] 112
5.36. (*R*)-*N*-[(*R*)-1-(2-Chloroquinolin-3-yl)ethyl]-2-methylpropane-2-sulfinamide [(*R*,*R*)-**24c**] 113
5.37. (*S*)-5-[{1-(6-Fluoro-2-oxo-1,2-dihydroquinoline-3-yl)ethyl}amino]-1-methyl-6-oxo-1,6-dihydropyridine-2-carbonitrile [(*S*)-**2**].. 114
5.38. (*R*)-5-[{1-(6-Fluoro-2-oxo-1,2-dihydroquinoline-3-yl)ethyl}amino]-1-methyl-6-oxo-1,6-dihydropyridine-2-carbonitrile [(*R*)-**2**] 116

5.39. (*S*)-5-[{1-(6-Bromo-2-oxo-1,2-dihydroquinoline-3-yl)ethyl}amino]-1-methyl-6-oxo-1,6-dihydropyridine-2-carbonitrile [(*S*)-**26a**] 117
5.40. (*R*)-5-[{1-(6-Bromo-2-oxo-1,2-dihydroquinoline-3-yl)ethyl}amino]-1-methyl-6-oxo-1,6-dihydropyridine-2-carbonitrile [(*R*)-**26a**] 118
5.41. (*S*)-5-[{1-(2-Oxo-1,2-dihydroquinoline-3-yl)ethyl}amino]-1-methyl-6-oxo-1,6-dihydropyridine-2-carbonitrile [(*S*)-**26c**] 119
5.42. (*R*)-5-[{1-(2-Oxo-1,2-dihydroquinoline-3-yl)ethyl}amino]-1-methyl-6-oxo-1,6-dihydropyridine-2-carbonitrile [(*R*)-**26c**] 120
5.43. (*S*)-((6-Bromo-3-(1-((6-cyano-1-methyl-2-oxo-1,2-dihydropyridin-3-yl)amino)ethyl)quinolin-2-yl)oxy)methyl pivalate [(*S*)-**27a**] and (*S*)-(6-bromo-3-(1-((6-cyano-1-methyl-2-oxo-1,2-dihydropyridin-3-yl)amino)ethyl)-2-oxoquinolin-1(2*H*)-yl)methyl pivalate [(*S*)-**28a**] 121
5.44. (*R*)-((6-Bromo-3-(1-((6-cyano-1-methyl-2-oxo-1,2-dihydropyridin-3-yl)amino)ethyl)quinolin-2-yl)oxy)methyl pivalate [(*R*)-**27a**] and (*R*)-(6-bromo-3-(1-((6-cyano-1-methyl-2-oxo-1,2-dihydropyridin-3-yl)amino)ethyl)-2-oxoquinolin-1(2*H*)-yl)methyl pivalate [(*R*)-**28a**] 122
5.45. (*S*)-(3-(1-((6-Cyano-1-methyl-2-oxo-1,2-dihydropyridin-3-yl)amino)ethyl)-2-oxo-6-(4,4,5,5-tetramethyl-1,3,2-dioxaborolan-2-yl)quinolin-1(2*H*)-yl)methyl pivalate [(*S*)-**29**] 123
5.46. (*S*)-((3-(1-((6-Cyano-1-methyl-2-oxo-1,2-dihydropyridin-3-yl)amino)ethyl)-6-(4,4,5,5-tetramethyl-1,3,2-dioxaborolan-2-yl)quinolin-2-yl)oxy)methyl pivalate [(*S*)-**30**] 124
5.47. (*R*)-(3-(1-((6-Cyano-1-methyl-2-oxo-1,2-dihydropyridin-3-yl)amino)ethyl)-2-oxo-6-(4,4,5,5-tetramethyl-1,3,2-dioxaborolan-2-yl)quinolin-1(2*H*)-yl)methyl pivalate [(*R*)-**29**] 125
5.48. (*R*)-((3-(1-((6-Cyano-1-methyl-2-oxo-1,2-dihydropyridin-3-yl)amino)ethyl)-6-(4,4,5,5-tetramethyl-1,3,2-dioxaborolan-2-yl)quinolin-2-yl)oxy)methyl pivalate [(*R*)-**30**] 126
5.49. 2-Cyano-5-fluoropyridine 1-oxide (**S3**) 127
5.50. 6-Cyano-3-fluoropyridin-2-yl acetate (**S4**) 129
5.51. 6-Cyanopyridine-2,3-diyl diacetate (**S4b**) 130
5.52. 5-Fluoro-6-oxo-1,6-dihydropyridine-2-carbonitrile (**S5**) 131
5.53. 5-Fluoro-1-methyl-6-oxo-1,6-dihydropyridine-2-carbonitrile (**31**) 132
5.54. 5-Fluoro-6-methoxypicolinonitrile (**31b**) 134
5.55. 2-Fluoroethyl methanesulfonate (**33**) 135
5.56. 2-(2-Fluoroethoxy)-4-nitrobenzonitrile (**35a**) 136
5.57. 2-(2-(Benzyloxy)ethoxy)-4-nitrobenzonitrile (**35b**) 138
5.58. 4-Amino-2-(2-fluoroethoxy)benzonitrile (**36a**) 139
5.59. 4-Amino-2-(2-(benzyloxy)ethoxy)benzonitrile (**36b**) 141
5.60. ((3-(((4-Cyano-3-(2-fluoroethoxy)phenyl)amino)methyl)-6-fluoroquinolin-2-yl)oxy)methyl pivalate (**37a**) 142
5.61. 2-(2-Cyano-5-(((6-fluoro-2-((pivaloyloxy)methoxy)quinolin-3-yl)methyl)amino)phenoxy)ethyl benzoate (**37b**) 143
5.62. 2-(2-Cyano-5-(((6-fluoro-2-oxo-1-((pivaloyloxy)methyl)-1,2-dihydroquinolin-3-yl)methyl)amino)phenoxy)ethyl benzoate (**S6**) 145
5.63. 4-(((6-Fluoro-2-oxo-1,2-dihydroquinolin-3-yl)methyl)amino)-2-(2-fluoroethoxy)benzonitrile (**5**). 147
5.64. ((3-(((4-Cyano-3-(2-hydroxyethoxy)phenyl)amino)methyl)-6-fluoroquinolin-2-yl)oxy)methyl pivalate (**38**) 149
5.65. ((3-(((4-Cyano-3-(2-((methylsulfonyl)oxy)ethoxy)phenyl)amino)methyl)-6-fluoroquinolin-2-yl)oxy)methyl pivalate (**39**) 151

1. General information

1.1. Solvents and reagents

Unless noted otherwise, all solvents and reagents were purchased from MerckKGaA (Darmstadt, Germany), Merck (Taufkirchen, Germany), Activate Scientific (Prien, Germany) or ABCR GmbH (Karlsruhe, Germany) and used without further purification.

1.2. Spectroscopy

Nuclear magnetic resonance (NMR) spectra were recorded in 5% solution at 299 K (^{1}H: 400.13 MHz; ^{13}C: 100.61 MHz; ^{19}F: 376.49 MHz) using a Bruker Avance Neo 400 (Bruker Bio Spin GmbH, Rheinstetten, Germany). The measured chemical shifts (δ) are reported in parts per million (ppm) relative to the residual solvent signals (for $CDCl_3$, δ_H = 7.26, δ_C = 77.16; for $(CD_3)_2SO$, δ_H = 2.50, δ_C = 50.32). The ^{1}H-NMR spectra are reported as follows: δ (in ppm) (number of protons, multiplicity, coupling constant J (in Hertz) where appropriate, assignment). The following abbreviations and their combinations are used when reporting NMR data: s = singlet, d = doublet, t = triplet, q = quartet, p = quintet and m = multiplet. NMR signals were assigned based on information from additional two-dimensional experiments (COSY, gHSQC). Coupling constants for protons are given in the form $^{n}J(^{1}H, X)$, those for carbons as $^{n}J(^{13}C,^{19}F)$. All ^{13}C-, and ^{19}F-NMR spectra were recorded under ^{1}H-broadband decoupling (CPD). Compound names were generated by ChemDraw Professional 21.0.0 (PerkinElmer Informatics, Inc.) following IUPAC nomenclature. Numbering of compounds is illustrated on the structures themselves (vide infra).
Low-resolution mass spectra were obtained in electrospray ionization (ESI positive) mode with a Thermo Finnigan Surveyor mass spectrometer (Thermo Fisher Scientific GmbH, Dreieich, Germany). The analytes were dissolved in methanol (about 1 mg/ mL) and injected directly through a valve on the ionization interface. The flow rate of the eluent (methanol/water/acetic acid, 50/50/0.2, v/v/v) was 200 mL/min. Reported are the m/z-values of the pseudo-ion $[M+H]^+$.
High resolution mass spectrometry and elemental analyses were performed by the University of Cologne. Analyses indicated by the symbols of the elements are within ±0.4% of the theoretical values.

1.3. Chromatography

Thin layer chromatography (TLC) on silica coated TLC aluminum sheets with fluorescent indicator (SIL ALUGRAM G/UV254 Macherey-Nagel GmbH, Düren, Germany) was

performed to monitor the progress of all reactions. Chromatograms were visualized under UV light.

Flash chromatography was performed with a Grace Reveleris® iES flash chromatography system equipped with RevealXTM multisignal (UV/ELSD) detection and Reveleris® flash silica cartridges (size 12 to 40 mm) as stationary phase.

2. Organic preparative syntheses

2.1 Preparation of precursors **14, 21** and **22,** reference compound **4** and protodeboronated impurity **13c**

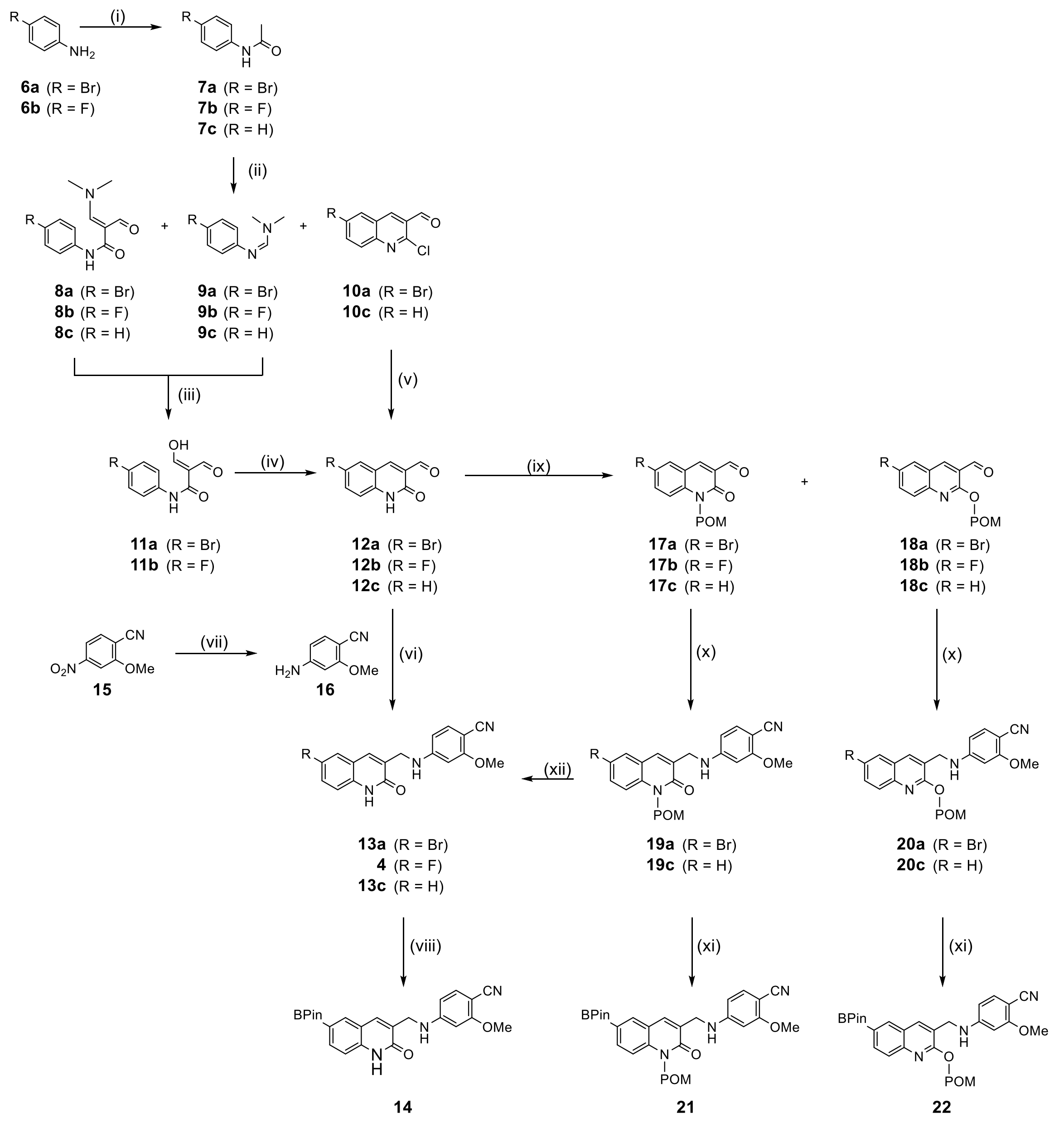

Scheme S1. *Preparation of reference compound* ***4****, unprotected radiofluorination precursor* ***14*** *and POM-protected radiofluorination precursors* ***21*** *and* ***22****.*
i) DIPEA, Ac_2O, AcOEt, rt, 16 h; ii) DMF, $POCl_3$, 75 °C, 1.2 h; iii) EtOH : 20% NaOH (1.1:1), HCl, 3 h; iv) PPA, 150 °C, 15 min; v) HCl, reflux, 16 h; vi) ***16*** *(1 eq.), DCE, AcOH, $NaBH(OAc)_3$, rt, 16 h; vii) NH_4Cl, zinc powder, EtOH : AcOEt (1:1), 40 °C, 2 h; viii) $Bpin)_2$, KOAc, $Pd(Cl)_2dppf$, dioxane, 80 °C, 22 h; ix) POM-Cl, Na_2CO_3, DMF, 60 °C, 2 h; x)* ***16*** *TMS-OTf, $NaBH_4$, DMF, 0 °C, 45 min, xi) $(Bpin)_2$, KOAc, $Pd(dppf)Cl_2$, dioxane, 80 °C, overnight; xii) MeOH:$NaOH_{aq.}$, rt, 72 h.*

Preparation of 4-bromoacetanilide (7a)

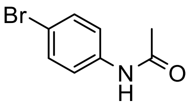

Acetic anhydride (1.39 mL, 14.70 mmol, 1.1 eq.) and *N*,*N*-diisopropylethylamine (2.6 mL, 14.70 mmol, 1.1 eq.) were added to a solution of 4-bromoaniline (**6a**, 2.30 g, 13.37 mmol) in AcOEt (68 mL, [0.1]) and the mixture was stirred at room temperature until TLC (Hex:AcOEt 4:1) indicated complete consumption of the starting material (~ 16 h). The organic phase was washed with H_2O (2 × 15 mL) and brine, dried over Na_2SO_4 and concentrated under reduced pressure to afford **7a** (2.80 g, 13.08 mmol, 97%) as a white solid.

R_f : 0.2

^{1}H-NMR [400 MHz, $(CD_3)_2SO$]: δ 10.06 (s, 1H), 7.56 (d, *J* = 8.8 Hz, 2H), 7.46 (d, *J* = 8.8 Hz, 2H), 2.05 (s, 3H). ^{13}C-NMR [101 MHz, $(CD_3)_2SO$]: δ 168.92 (s), 139.15 (s), 131.91 (s), 121.32 (s), 114.95 (s), 24.48 (s).

HRMS (ESI) calcd for C_8H_8BrNO $[M+H]^+$: 214.06, found: 213.9863; M.p. = 167 °C

Preparation of 4-fluoroacetanilide (7b)

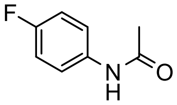

7b (2.06 g, 13.45 mmol, 100%, white solid) was prepared from 4-fluoroaniline (6b, 1.50 g, 13.48 mmol) using the same procedure as described for **7a**.

R_f : 0.2

^{1}H-NMR [400 MHz, $(CD_3)_2SO$]: δ 9.97 (s, 1H), 7.74 – 7.45 (m, 2H), 7.28 – 6.99 (m, 2H), 2.03 (s, 3H). ^{13}C-NMR [101 MHz, $(CD_3)_2SO$]: δ 168.61 (s), 158.28 (d, *J* = 239.5 Hz), 136.19 (d, *J* = 2.4 Hz), 121.13 (d, *J* = 7.7 Hz), 115.63 (d, *J* = 22.3 Hz), 24.30 (s). ^{19}F-NMR [376 MHz, $(CD_3)_2SO$]: δ -119.82.

HRMS (ESI) calcd for C_8H_8FNO $[M+H]^+$: 153.06, found: 154.0663; M.p. = 153 °C

Preparation of (E)-*N*-(4-bromophenyl)-3-(dimethylamino)-2-formylacrylamide (8a), (Z)-*N*'-(4-bromophenyl)-*N*,*N*-dimethylformimidamide (9a) and 6-bromo-2-chloroquinoline-3-carbaldehyde (10a)

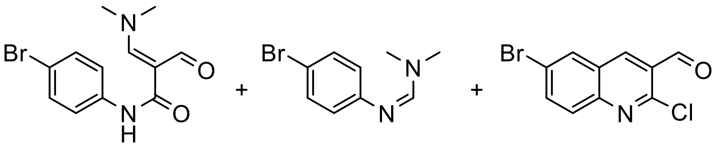

8a **9a** **10a**

$POCl_3$ (6.00 mL, 65.8 mmol, 7 eq.) was added dropwise under argon to pre-cooled (0 °C) anhydrous DMF (1.82 mL, 23.5 mmol, 2.5 eq.). The mixture was allowed to warm to room temperature and **7a** (2.01 g, 9.40 mmol) was added. After vigorous stirring at room temperature for 5 minutes, the reaction mixture was heated at 75 °C until TLC (Hex:AcOEt 3:2) indicated complete consumption of the starting material. The

reaction was then quenched by addition of ice and the resulting precipitate was recovered by filtration to afford **10a** (76 mg, 0.28 mmol, 3%) as an orange solid. The filtrate was treated with 40% aqueous NaOH and extracted with $CHCl_3$. The combined organic fractions were dried over Na_2SO_4 and concentrated under reduced pressure to afford a mixture of **8a** and **9a** as an orange oil that was used without further purification.

6-Bromo-2-chloroquinoline-3-carbaldehyde (**10a**)

R_f : 0.89

^{1}H-NMR [400 MHz, $(CD_3)_2SO$]: δ 10.33 (s, 1H), 8,97 (s, 1H), 8.61 (d, J=2.3 Hz, 1H), 8.12 (dd, J=9.0, 2.3 Hz, 1H), 8.00 (d, J=9.0 Hz, 1H). ^{13}C-NMR [101 MHz, $(CD_3)_2SO$]: δ 189.67 (s), 150.10 (s), 147.65 (s), 140.78 (s), 137.10 (s), 132.46 (s), 130.37 (s), 128.19 (s), 127.56 (s), 121.55 (s).

MS (ESI) calcd for $C_{10}H_5BrClNO$ $[M+H]^+$: 270.51, found: 227.12; M.p. = 164 °C

Preparation of (E)-*N*-(4-fluorophenyl)-3-(dimethylamino)-2-formylacrylamide (8b) and (Z)-*N*'-(4-fluorophenyl)-*N*,*N*-dimethylformimidamide (9b)

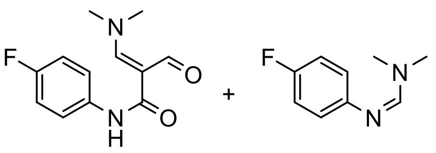

8b **9b**

A mixture of **8b** and **9b** (orange oil) was prepared from **7b** (1.50 g, 13.48 mmol) using the same procedure as described for **8a** and **9a**.

Preparation of (E)-3-(dimethylamino)-2-formyl-*N*-phenylacrylamide (8c), (Z)-*N*,*N*-dimethyl-*N*'-phenylformimidamide (9c) and 2-chloroquinoline-3-carbaldehyde (10c)

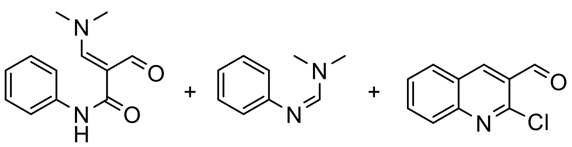

8c **9c** **10c**

10c (830.0 mg, 4.33 mmol, 29%, white solid) and a mixture of **8c** and **9c** were prepared from **7c** (2.0 g, 14.79 mmol) using the same procedure as described for **8a**, **9a** and **10a**.

2-Chloroquinoline-3-carbaldehyde (**10c**)

R_f : 0.78

^{1}H-NMR [400 MHz, $(CD_3)_2SO$]: δ 10.38 (s, 1H), 8.98 (s, 1H), 8.30 – 8.25 (m, 1H), 8.04 (ddt, *J* = 8.5, 1.4, 0.7 Hz, 1H), 7.98 (ddd, *J* = 10.1, 5.8, 2.4 Hz, 1H), 7.76 (ddd, *J* = 8.1, 6.7, 1.4 Hz, 1H). ^{13}C-NMR [101 MHz, $(CD_3)_2SO$]: δ 189.84 (s), 149.45 (s), 149.02 (s), 141.85 (s), 134.35 (s), 130.68 (s), 128.72 (s), 128.24 (s), 126.82 (s), 126.80 (s).

MS (ESI) calcd for $C_{10}H_6ClNO$ $[M+H]^+$: 191.61, found: 224.17 (adduct with MeOH); M.p. = 146 °C

Preparation of *N*-(4-bromophenyl)-2-formyl-3-hydroxyacrylamide (11a)

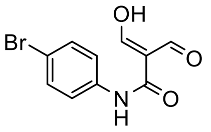

A solution of the mixture of **8a** and **9a** (1 eq. calculated based on the 4-bromoacetanilide) in EtOH and 20% aqueous NaOH (1.1:1.0) (135 mL, [0.1]) was boiled for 2 minutes and immediately poured onto ice. The resulting mixture was treated with concentrated HCl until the pH was 3 and vigorously stirred for 3 h. The resulting precipitate was collected by filtration to afford **11a** (1.67 mg, 6.17 mmol, 65% over two steps) as an orange solid.

R_f : 0.0

^{1}H-NMR [400 MHz, $(CD_3)_2SO$]: δ 10.70 (s, 1H), 9.02 (s, 2H), 7.62 – 7.57 (m, 2H), 7.57 – 7.52 (m, 2H). ^{13}C-NMR [101 MHz, $(CD_3)_2SO$]: δ 188.04 – 184.91 (m), 166.20 (s), 136.86 (s), 132.26 (s), 122.98 (s), 116.77 (s), 111.32 (s).

MS (ESI) calcd for $C_{10}H_8BrNO_3$ $[M+H]^+$: 270.08, found: 227.12 (Formamidine **9a**); M.p. = 242 °C

Preparation of *N*-(4-fluorophenyl)-2-formyl-3-hydroxyacrylamide (11b)

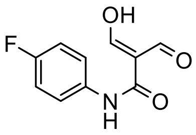

11b (1.13 g, 5.40 mmol, 41% over two steps) was prepared from the mixture of **8b** and **9b** using the same procedure as described for **11a**.

R_f : 0.0

^{1}H-NMR [400 MHz, $(CD_3)_2SO$]: δ 10.67 (s, 1H), 9.02 (s, 2H), 7.71 – 7.51 (m, 2H), 7.28 – 7.10 (m, 2H). ^{13}C NMR [101 MHz, $(CD_3)_2SO$]: δ 187.20 (s), 166.67 (s), 159.37 (d, J = 241.7 Hz), 133.52 (s), 123.25 (d, J = 8.1 Hz), 116.09 (d, J = 22.5 Hz), 110.79 (s). ^{19}F-NMR [376 MHz, $(CD_3)_2SO$]: δ -117.71 (s).

MS (ESI) calcd for $C_{10}H_8BrNO_3$ $[M+H]^+$: 209.18, found: no peak; M.p. > 300 °C

Preparation of 6-bromo-2-oxo-1,2-dihydroquinoline-3-carbaldehyde (12a)

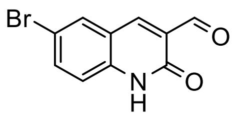

A mixture of **11a** (1.67 g, 6.17 mmol) and polyphosporic acid (10 grams for each gram of starting material, 16.7 g) was stirred at 150 °C (internal temperature) for 15 min, after which TLC (Hex:AcOEt 4:1) indicated complete consumption of the starting material. The mixture was poured onto ice and the resulting precipitate collected by filtration to afford **12a** (925 mg, 3.67 mmol, 60%) as a black solid.

R_f : 0.0

^{1}H-NMR [400 MHz, $(CD_3)_2SO$]: δ 12.36 (s, 1H), 10.23 (s, 1H), 8.48 (s, 1H), 8.21 (d, *J* = 2.3 Hz, 1H), 7.80 (dd, *J* = 8.8, 2.3 Hz, 1H). ^{13}C-NMR [101 MHz, $(CD_3)_2SO$]: δ 190.13 (s), 161.66 (s), 141.65 (s), 140.57 (s), 136.43 (s), 133.05 (s), 126.88 (s), 120.27 (s), 118.06 (s), 114.57 (s).
HRMS (ESI) calcd for $C_{10}H_6BrNO_2$ $[M+H]^+$: 252.07, found: 251.9655; M.p. > 300 °C

Preparation of 6-fluoro-2-oxo-1,2-dihydroquinoline-3-carbaldehyde (12b)

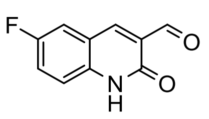

12b (1.00 g, 5.23 mmol, 96%, black solid) was prepared from **11b** (1.13 g, 5.40 mmol) and polyphosphoric acid (11.3 g) using the same procedure as described for **12a**.

R_f : 0.0

^{1}H-NMR [400 MHz, $(CD_3)_2SO$]: δ 12.31 (s, 1H), 10.24 (s, 1H), 8.49 (s, 1H), 7.81 (dd, *J* = 9.0, 2.9 Hz, 1H), 7.58 (td, *J* = 8.9, 2.9 Hz, 1H), 7.39 (dd, *J* = 9.1, 4.7 Hz, 1H). ^{13}C-NMR [101 MHz, $(CD_3)_2SO$]: δ 190.23 (s), 160.18 (d, *J* = 288.6 Hz), 156.37 (s), 141.93 (d, *J* = 3.6 Hz), 138.44 (s), 126.91 (s), 122.44 (d, *J* = 24.9 Hz), 119.17 (d, *J* = 9.5 Hz), 117.90 (d, *J* = 8.3 Hz), 115.50 (d, *J* = 22.8 Hz). ^{19}F-NMR [376 MHz, $(CD_3)_2SO$]: δ -120.11.
HRMS (ESI) calcd for $C_{10}H_6BrNO_2$ $[M+H]^+$: 191.16, found: 192.0456; M.p. > 300 °C

Preparation of 2-oxo-1,2-dihydroquinoline-3-carbaldehyde (12c)

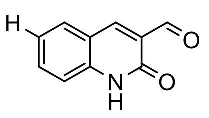

10c (830 mg, 4.33 mmol) was suspended in 2 M HCl (12 mL) and the reaction mixture was refluxed for 16 hours. The mixture was diluted with H_2O and the resulting precipitate was recovered by filtration to afford **12c** (619.0 mg, 3.57 mmol, 82%) as an orange solid.

^{1}H-NMR [400 MHz, $(CD_3)_2SO$]: δ 10.38 (s, 1H), 8.98 (s, 1H), 8.30 – 8.25 (m, 1H), 8.04 (ddt, J = 8.5, 1.4, 0.7 Hz, 1H), 7.98 (ddd, J = 10.1, 5.8, 2.4 Hz, 1H), 7.76 (ddd, J = 8.1, 6.7, 1.4 Hz, 1H). ^{13}C-NMR [101 MHz, $(CD_3)_2SO$]: δ 189.84 (s), 149.45 (s), 149.02 (s), 141.85 (s), 134.35 (s), 130.68 (s), 128.72 (s), 128.24 (s), 126.82 (s), 126.80 (s).
MS (ESI) calcd for $C_{10}H_7NO_2$ $[M+H]^+$: 173.17, found: 174.10; M.p. = 146 °C

Preparation of 4-{[(6-bromo-2-oxo-1,2-dihydroquinolin-3-yl)methyl]amino}-2-methoxybenzonitrile (13a)

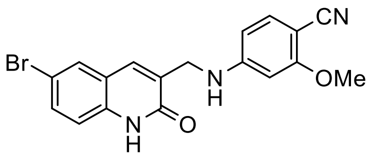

Acetic acid (300 µL, 3 eq.) was added under argon to a suspension of **12a** (435.50 mg, 1.73 mmol, 1 eq.) and 4-amino-2-methoxybenzonitrile (**16**, 259.0 mg, 1.73 mmol, 1 eq.) in anhydrous DCE (10.2 mL, [0.17]) until all solid were completely dissolved. After vigorous stirring under argon for 16 hours, $NaBH(OAc)_3$ (733.31 mg, 3.46 mmol, 2 eq.) was added and the mixture was stirred for another 6 hours, after which TLC ($CHCl_3$:AcOEt 3:7) indicated no further reaction. The mixture was diluted with chloroform and washed with H_2O and saturated $NaHCO_3$ (until the aqueous phase was free from UV active compounds), after which the organic phase was dried over Na_2SO_4 and concentrated under reduced pressure. The crude product was purified by column chromatography with a $CHCl_3$:AcOEt gradient to afford **13a** (296.80 mg, 0.77 mmol, 45%) as a white solid.

R_f : 0.6

^{1}H-NMR [400 MHz, $(CD_3)_2SO$]: δ 12.05 (s, 1H), 7.92 (d, J = 2.0 Hz, 1H), 7.73 (s, 1H), 7.61 (dd, J = 8.7, 2.2 Hz, 1H), 7.29 (d, J = 8.6 Hz, 1H), 7.26 (d, J = 8.8 Hz, 1H), 7.16 (t, J = 5.9 Hz, 1H), 6.33 (s, 1H), 6.23 (dd, J = 8.5, 1.9 Hz, 1H), 4.25 (d, J = 4.9 Hz, 2H), 3.80 (s, 2H). ^{13}C-NMR [101 MHz, $(CD_3)_2SO$]: δ 162.86 (s), 161.88 (s), 154.42 (s), 137.57 (s), 134.70 (s), 134.67 (s), 132.79 (s), 131.87 (s), 130.10 (s), 121.25 (s), 118.70 (s), 117.58 (s), 113.93 (s), 105.47 (s), 95.00 (s), 86.58 (s), 56.05 (s), 41.87 (s).

HRMS (ESI) calcd for $C_{18}H_{14}BrN_3O_2$ $[M+H]^+$: 384.23, found: 384.0342; M.p. > 300 °C

Preparation of 4-{[(6-fluoro-2-oxo-1,2-dihydroquinolin-3-yl)methyl]amino}-2-methoxybenzonitrile (4)

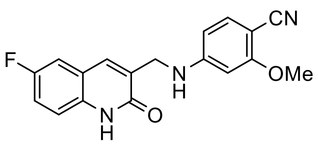

4 (106.70 mg, 0.33 mmol, 33%, white solid) was prepared from **12b** (193.00 mg, 1.01 mmol) and **16** (148.10 mg, 1.00 mmol) using the same procedure as described for 13a.

R_f : 0.6

^{1}H-NMR [400 MHz, $(CD_3)_2SO$]: δ 12.00 (s, 1H), 7.74 (s, 1H), 7.55 (dd, J = 9.2, 2.5 Hz, 1H), 7.41 – 7.32 (m, 2H), 7.30 (d, J = 8.6 Hz, 1H), 7.17 (t, J = 6.0 Hz, 1H), 6.34 (d, J = 1.7 Hz, 1H), 6.24 (dd, J = 8.6, 1.8 Hz, 1H), 4.26 (d, J = 5.7 Hz, 2H), 3.80 (s, 3H). ^{13}C-NMR [101 MHz, $(CD_3)_2SO$]: δ 162.29 (d, J = 115.1 Hz), 158.68 (s), 156.31 (s), 154.44 (s), 135.15 (s), 135.13 (s), 134.95 (d, J = 3.1 Hz), 134.74 (s), 131.95 (s), 120.19 (d, J = 8.8 Hz), 118.68 (s), 118.30 (d, J = 24.4 Hz), 117.14 (d, J = 8.5 Hz), 112.92 (d, J = 23.0 Hz), 105.45 (s), 95.00 (s), 86.60 (s), 60.23 (s), 56.05 (s). ^{19}F-NMR (376 MHz, $(CD_3)_2SO$]: δ -121.03 (s).

HRMS (ESI) calcd for $C_{18}H_{14}FN_3O_2$ $[M+H]^+$: 323.33, found: 324.1140; M.p. = 222 °C

Preparation of 2-methoxy-4-{[(2-oxo-6-(4,4,5,5-tetramethyl-1,3,2-dioxaborolan-2-yl)-1,2-dihydroquinolin-3-yl)methyl]amino}benzonitrile (14)

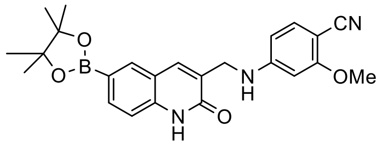

A solution of **13a** (296.80 mg, 0.77 mmol, 1 eq.), $(Bpin)_2$ (215.70 mg, 1.10 mmol, 1.5 eq.), KOAc (532.0 mg, 5.40 mmol, 7 eq.) and $[Pd(Cl)_2dppf]$ (28.20 mg, 0.04 mmol, 5%) in anhydrous dioxane (3.8 mL, [0.2]) was vigorously stirred under nitrogen at 80 °C for 17 hours until TLC ($CHCl_3$:AcOEt 1:1) indicated no further consumption of the starting materials over time. The reaction mixture was diluted with ethyl acetate and washed with brine, after which the organic phase was dried over $NaSO_4$ and concentrated under reduced pressure. The crude product was purified by column chromatography with a $CHCl_3$:AcOEt gradient to afford **14** (43.0 mg, 0.10 mmol, 17%) as a white solid.

R_f : 0.4

^{1}H-NMR [400 MHz, $(CD_3)_2SO$]: δ 12.06 (s, 1H), 7.95 (s, 1H), 7.85 (s, 1H), 7.72 (dd, *J* = 8.2, 1.3 Hz, 1H), 7.30 (dd, *J* = 8.4, 2.2 Hz, 2H), 7.14 (t, *J* = 6.0 Hz, 1H), 6.35 (d, *J* = 1.7 Hz, 1H), 6.25 (dd, *J* = 8.6, 1.8 Hz, 1H), 4.23 (d, *J* = 5.7 Hz, 2H), 3.80 (s, 3H), 1.29 (s, 11H). ^{13}C-NMR [101 MHz, $(CD_3)_2SO$]: δ 162.83 (s), 162.28 (s), 154.41 (s), 140.68 (s), 136.48 (s), 135.73 (s), 135.32 (s), 134.66 (s), 130.40 (s), 119.05 (s), 118.74 (s), 114.92 (s), 105.38 (s), 95.02 (s), 86.46 (s), 84.16 (s), 56.03 (s), 41.86 (s), 25.15 (s).

HRMS (ESI) calcd for $C_{24}H_{26}BN_3O_4$ $[M+H]^+$: 431.30, found: 432.2088; M.p. = 262 °C

Preparation of 4-amino-2-methoxybenzonitrile (16)

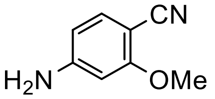

A mixture of 4-nitro-2-methoxybenzonitrile (**15**, 2.00 g, 11.23 mmol, 1 eq.), NH_4Cl (1.80 g, 33.68 mmol, 3 eq.) and zinc powder (2.20 g, 33.68 mmol, 3 eq.) in EtOH:AcOEt (1:1, 40 mL, [0.3]) was vigorously stirred at 40 °C until TLC (Hex:AcOEt 1:1) indicated complete consumption of the starting material (~ 2 h). The remaining zinc powder was removed by filtration over a celite pad and rinsed with additional AcOEt. The filtrate was washed with H_2O, dried over Na_2SO_4 and concentrated under reduced pressure to afford **16** (1.65 g, 11.13 mmol, 99%) as a white solid that could be used without further purification.

R_f : 0.38

^{1}H-NMR [400 MHz, $(CD_3)_2SO$]: δ 7.24 (d, *J* = 8.4 Hz, 1H), 6.27 (d, *J* = 1.9 Hz, 1H), 6.21 (dd, *J* = 8.4, 1.9 Hz, 1H), 6.18 (s, 2H), 3.78 (s, 3H). ^{13}C-NMR [101 MHz, $(CD_3)_2SO$]: δ 162.90 (s), 155.38 (s), 134.71 (s), 118.90 (s), 106.90 (s), 96.13 (s), 85.94 (s), 55.83 (s).
HRMS (ESI) calcd for $C_8H_8N_2O$ $[M+H]^+$: 148.17, found: 149.0709; M.p. = 100 °C

Preparation of (6-bromo-3-formyl-2-oxoquinolin-1(2*H*)-yl)methyl pivalate (17a) and [(6-bromo-3-formylquinolin-2-yl)oxy]methyl pivalate (18a)

A solution of **12a (**873.2 mg, 3.46 mmol, 1 eq.) and anhydrous K_2CO_3 (717.3 mg, 5.19 mmol, 1.5 eq.) in anhydrous DMF (17 mL, [0.2]) was stirred under argon at 60 °C for 30 min. The reaction mixture was allowed to cool to room temperature before POM-Cl (738 µL, 5.19 mmol, 1.5 eq.) was added and stirring continued for 2 h until TLC (Hex:AcOEt 4:1) indicated complete consumption of the starting material. The reaction mixture was then diluted with EtOAc and washed with H2O, after which the organic phase was dried over Na_2SO_4 and concentrated under reduced pressure. An aliquot of the residue was removed to determine the ratio of the two products, while the rest was purified by column chromatography (Hex: AcOEt 9:1) to afford **17a** (207.0 mg, 0.56 mmol, 16%) and **18a** (329.6 mg, 0.9 mmol, 26%) as pale yellow solids.

(6-Bromo-3-formyl-2-oxoquinolin-1(2*H*)-yl)methyl pivalate (**17a**)

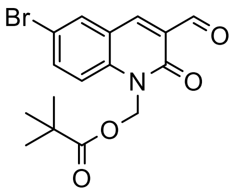

R_f : 0.4
^{1}H-NMR (400 MHz, $CDCl_3$): δ 10.46 (s, 1H), 8.32 (s, 1H), 7.90 (d, J = 2.3 Hz, 1H), 7.78 (dd, J = 9.1, 2.3 Hz, 1H), 7.30 (d, J = 9.1 Hz, 2H), 6.36 (s, J = 34.5 Hz, 2H), 1.22 (s, 9H). ^{13}C-NMR (101 MHz, $CDCl_3$): δ 189.09 (s), 177.71 (s), 161.11 (s), 144.18 (d, J = 103.4 Hz), 141.19 (s), 136.64 (s), 133.91 (s), 128.01 – 124.35 (m), 120.83 (s), 116.67 (s), 116.17 (s, J = 50.4 Hz), 65.51 (s), 39.11 (s), 26.98 (s).
HRMS (ESI) calcd for $C_{16}H_{16}BrNO_4$ $[M+H+Na]^+$: 366.22, found: 388.0154; M.p. = 166 °C

[(6-Bromo-3-formylquinolin-2-yl)oxy]methyl pivalate (**18a**)

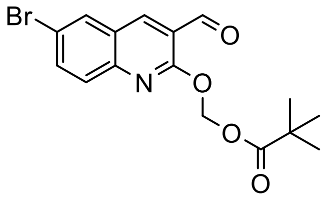

R_f : 0.8
^{1}H-NMR (400 MHz, $CDCl_3$): δ 10.44 (s, 1H), 8.53 (s, 1H), 7.99 (d, J = 2.1 Hz, 1H), 7.80 (dd, J = 8.9, 2.2 Hz, 1H), 7.73 (d, J = 9.0 Hz, 1H), 6.33 (s, 2H), 1.22 (s, J = 12.5 Hz, 10H). ^{13}C-NMR (101 MHz, $CDCl_3$): δ 188.15 (s), 177.32 (s), 159.06 (s), 146.94 (s), 139.02 (s), 135.95 (s), 131.43 (s), 129.22 (s), 126.07 (s), 120.19 (s), 119.09 (s), 82.29 (s), 38.84 (s), 26.90 (s).

MS (ESI) calcd for $C_{16}H_{16}BrNO_4$ $[M+H]^+$: 366.22, found: 399.76 (MeOH adduct); M.p. = 149 °C

Preparation of (6-fluoro-3-formyl-2-oxoquinolin-1(2*H*)-yl)methyl pivalate (17b) and [(6-fluoro-3-formylquinolin-2-yl)oxy]methyl pivalate (18b)

17b (85 mg, 0.28 mmol, 24%, pale yellow solid) and **18b** (178.75 mg, 0.58 mmol, 49%, pale yellow solid) were prepared from **12b** (226.90 mg, 1.18 mmol, 1 eq.) using the same procedure as described for **17a** and **18a**.

(6-Fluoro-3-formyl-2-oxoquinolin-1(2*H*)-yl)methyl pivalate (**17b**)

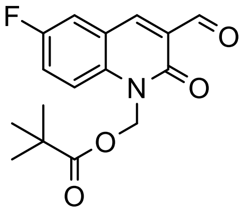

R_f : 0.3

^{1}H-NMR [400 MHz, $(CD_3)_2SO$]: δ 10.22 (s, 1H), 8.52 (s, 1H), 7.89 (dd, J = 8.5, 3.0 Hz, 1H), 7.74 – 7.65 (m, 1H), 7.61 (dd, J = 9.4, 4.4 Hz, 1H), 6.29 (s, 2H), 1.11 (s, J = 6.4 Hz, 10H). ^{13}C-NMR [101 MHz, $(CD_3)_2SO$]: δ 189.65 (s), 177.08 (s), 160.67 (s), 158.08 (d, J = 241.5 Hz), 142.38 (d, J = 3.0 Hz), 137.69 (s), 126.00 (s), 122.38 (d, J = 24.3 Hz), 120.30 (d, J = 9.2 Hz), 117.68 (d, J = 8.3 Hz), 117.00 (d, J = 22.9 Hz). ^{19}F-NMR [376 MHz, $(CD_3)_2SO$]: δ -119.47 (s). HRMS (ESI) calcd for $C_{16}H_{16}FNO_4$ $[M+H]^+$: 305.31, found: 306.1139; M.p. = 114 °C

[(6-Fluoro-3-formylquinolin-2-yl)oxy]methyl pivalate (**18b**)

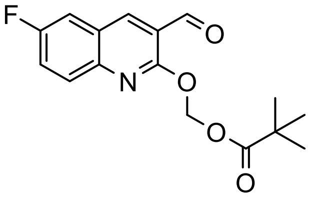

R_f : 0.7

^{1}H-NMR [400 MHz, $(CD_3)_2SO$]: δ 10.27 (s, 1H), 8.75 (s, 1H), 7.90 (dd, J = 9.0, 2.8 Hz, 1H), 7.80 (dt, J = 20.6, 10.3 Hz, 1H), 7.71 (td, J = 8.9, 2.9 Hz, 1H), 6.25 (s, 2H), 1.12 (s, 9H). ^{13}C-NMR [101 MHz, $(CD_3)_2SO$]: δ 188.57 (s), 176.86 (s), 160.63 (s), 158.96 – 156.87 (m), 144.84 (s), 140.83 (d, J = 5.1 Hz), 129.75 (d, J = 8.9 Hz), 125.64 (d, J = 10.6 Hz), 123.15 (d, J = 25.7 Hz), 120.39 (s), 113.66 (d, J = 22.3 Hz), 82.64 (s), 26.92 (s). ^{19}F-NMR [376 MHz, $(CD_3)_2SO$]: δ -114.96 (s). HRMS (ESI) calcd for $C_{16}H_{16}FNO_4$ $[M+H]^+$: 305.31, found: 338.15 (MeOH adduct); M.p. = 92 °C

Preparation of (3-formyl-2-oxoquinolin-1(2*H*)-yl)methyl pivalate (17c) and [(3-formylquinolin-2-yl)oxy]methyl pivalate (18c)

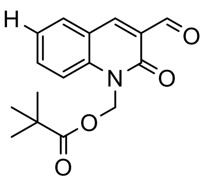

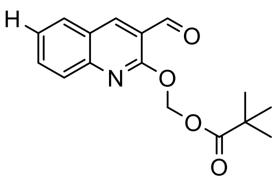

A mixture of **17c** and **18c** (213.22 mg, 0.74 mmol, 63%, pale yellow solid) was prepared from **12c** (200.0 mg, 1.15 mmol, 1 eq.) using the same procedure as described for **17a** and **18a** and used without separation or purification

R_f : 0.3 (**17c**) / 0.8 (**18c**)

^{1}H-NMR [400 MHz, $(CD_3)_2SO$]: δ 10.31 (s, 1H), 10.25 (s, 1H), 8.87 (s, 1H), 8.60 (s, 1H), 8.19 – 8.15 (m, 1H), 8.05 (dd, J = 7.8, 1.5 Hz, 1H), 7.62 – 7.56 (m, 2H), 7.44 – 7.38 (m, 1H), 6.31 (s,J = 3.3 Hz, 1H), 6.30 (s, 2H), 1.14 (s, 10H), 1.13 (s, J = 4.4 Hz, 7H). ^{13}C-NMR [101 MHz, $(CD_3)_2SO$]: δ 189.81 (s), 188.87 (s), 177.21 (s), 176.94 (s), 161.01 (s, J = 3.0 Hz), 158.78 (s), 147.89 (s), 143.64 (s), 141.83 (s), 140.98 (s), 134.79 (s), 133.79 (s), 132.73 (s), 130.69 (s), 127.30 (s), 126.37 (s), 125.18 (s), 125.14 (s), 124.18 (s), 120.00 (s), 119.35 (s), 115.40 (s), 82.68 (s), 66.36 (s), 38.91 (s), 38.75 (s), 27.11 (s), 27.00 (s).

MS (ESI) calcd for $C_{16}H_{17}NO_4$ $[M+H]^+$: 287.32, found: 288.41; M.p. = not determined

Preparation of 6-bromo-1-ethyl-2-oxo-1,2-dihydroquinoline-3-carbaldehyde (S1a) and 6-bromo-2-ethoxyquinoline-3-carbaldehyde (S1b)

S1a (25.2 mg, 0.1 mmol, 28%, yellow solid) and **S1b** (55.0 mg, 0.20 mmol, 56%, yellow solid) were prepared from **12a** (87.40 mg, 0.35 mmol) using the same procedure as described for **17a** and **18a** but with bromoethane (39 µL, 0.76 mmol, 2 eq.) instead of POM-Cl.

6-Bromo-1-ethyl-2-oxo-1,2-dihydroquinoline-3-carbaldehyde (**S1a**)

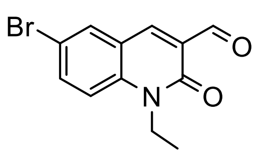

R_f : 0.2

^{1}H-NMR (400 MHz, DMSO) δ 10.27 (s, 1H), 8.47 (s, 1H), 8.28 (d, J = 2.4 Hz, 1H), 7.89 (dd, J = 9.1, 2.4 Hz, 1H), 7.63 (d, J = 9.2 Hz, 1H), 4.32 (q, J = 7.1 Hz, 2H), 1.24 (t, J = 7.1 Hz, 3H). ^{13}C-NMR (101 MHz, DMSO) δ 190.34 (s), 160.61 (s), 140.37 (d, J = 19.5 Hz), 140.27 (s), 136.65 (s), 134.18 (s), 125.99 (s), 121.24 (s), 117.70 (s), 114.90 (s), 37.46 (s), 13.01 (s).

HRMS (ESI) calcd for $C_{12}H_{10}BrNO_2$ $[M+H]^+$: 280.12, found: 279.9969; M.p. = 171 °C

6-Bromo-2-ethoxyquinoline-3-carbaldehyde (**S1b**)

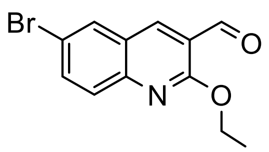

R_f : 0.8

^{1}H-NMR (400 MHz, DMSO) δ 10.33 (s, 1H), 8.69 (s, 1H), 8.35 (d, J = 2.3 Hz, 1H), 7.89 (dd, J = 8.9, 2.3 Hz, 1H), 7.71 (d, J = 8.9 Hz, 1H), 4.57

(q, J = 7.1 Hz, 2H), 1.44 (t, J = 7.1 Hz, 4H). ^{13}C-NMR (101 MHz, DMSO) δ 189.29 (s), 161.13 (s), 147.24 (s), 139.80 (s), 135.90 (s), 132.26 (s), 129.22 (s), 125.87 (s), 120.82 (s), 117.74 (s), 62.82 (s), 14.72 (s).

HRMS (ESI) calcd for $C_{12}H_{10}BrNO_2$ $[M+H]^+$: 280.12, found: 279.9970; M.p. = 112 °C

Preparation of (6-bromo-3-{[(4-cyano-3-methoxyphenyl)amino]methyl}-2-oxoquinolin-1(2*H*)-yl)methyl pivalate (19a)

A solution of **17a (**207.0 mg, 0.56 mmol) and **16** (74.4 mg, 0.51 mmol) in anhydrous DMF (1 mL, [0.5]) was cooled to 0 °C under argon before addition of TMS-Cl (161 µL, 1.27 mmol) and $NaBH_4$ (20 mg, 0.51 mmol). The reaction mixture was stirred for 2 h at 0 °C until TLC (Hex:AcOEt 6:4) indicated complete consumption of the limiting reagent **16**. After dilution with AcOEt and quenching of remaining TMS-Cl by addition of Na_2CO_3 (s.s.), the organic phase was dried over Na_2SO_4 and concentrated under reduced pressure. The crude product was purified by column chromatography (Hex: AcOEt 6:4) to afford **19a** (141.4 mg, 0.28 mmol, 55%) as a yellow solid.

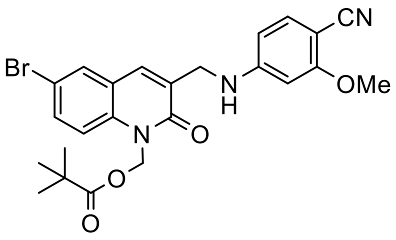

R_f : 0.4

^{1}H-NMR (400 MHz, $CDCl_3$): δ 7.70 (d, J = 2.2 Hz, 1H), 7.65 (dd, J = 9.0, 2.3 Hz, 1H), 7.60 (s, 1H), 7.33 (d, J = 8.5 Hz, 1H), 7.25 (d, J = 9.0 Hz, 1H), 6.35 (s, 2H), 6.24 (dd, J = 8.5, 2.1 Hz, 1H), 6.17 (d, J = 2.0 Hz, 1H), 4.44 (d, J = 0.9 Hz, 2H), 3.86 (s, 3H), 1.21 (s, J = 13.8 Hz, 11H). ^{13}C-NMR (101 MHz, $CDCl_3$): δ 177.65 (s), 162.99 (s), 161.28 (s), 152.35 (s), 136.89 (s), 135.54 (s), 134.96 (s), 133.50 (s), 131.16 (s), 130.08 (s), 121.65 (s), 117.81 (s), 116.24 (s), 115.79 (s), 105.15 (s), 95.56 (s), 90.23 (s), 66.02 (s), 55.79 (s), 43.66 (s), 40.97 – 37.24 (m), 26.99 (s).

HRMS (ESI) calcd for $C_{24}H_{24}BrN_3O_4$ $[M+H]^+$: 498.38, found: 498.1024; M.p. = 137 °C

Preparation of [(6-bromo-3-[{(4-cyano-3-methoxyphenyl)amino}methyl]quinolin-2-yl)oxy]methyl pivalate (20a)

20a (192.5 mg, 0.39 mmol, 48%, yellow solid) was prepared from **18a** (329.6 mg, 0.90 mmol) and **16** (121.2 mg, 0.81 mmol) using the same procedure as described for **19a** but with TMS-OTf (366 µL, 2.02 mmol) instead of TMS-Cl, a total reaction time of 75 minutes and a different solvent ratio (Hex: AcOEt 7:3) during purification by column chromatography.

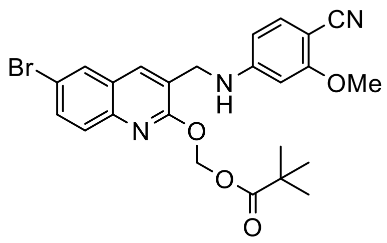

R_f : 0.6

^{1}H-NMR (400 MHz, $CDCl_3$): δ 7.86 (s, 1H), 7.83 (d, J = 1.6 Hz, 1H), 7.71 (d, J = 8.9 Hz, 1H), 7.68 (dd, J = 8.9, 1.9 Hz, 1H), 7.27 (d, J = 8.5 Hz, 1H), 6.27 (s, 2H), 6.19 (dd, J = 8.5, 1.8 Hz, 1H), 6.15 (d, J = 1.6 Hz, 1H), 5.13 (t, J = 5.9 Hz, 1H), 4.48 (d, J = 5.8 Hz, 2H), 3.81 (s, 3H), 1.20 (s, 9H). ^{13}C-NMR (101 MHz, $CDCl_3$): δ 177.83 (s), 162.92 (s), 157.95 (s), 152.77 (s), 143.99 (s), 135.57 (s), 134.82 (s), 132.91 (s), 129.38 (s), 128.97 (s), 126.81 (s), 122.79 (s), 118.37 (s), 118.13 (s), 104.92 (s), 95.20 (s), 89.43 (s), 82.26 (s), 55.67 (s), 42.49 (s), 26.91 (s).

HRMS (ESI) calcd for $C_{24}H_{24}BrN_3O_4$ $[M+H]^+$: 498.38, found: 498.1024; M.p. = 152 °C

Preparation of (3-[{(4-cyano-3-methoxyphenyl)amino}methyl]-2-oxoquinolin-1(2*H*)-yl)methyl pivalate (19c) and [(3-[{(4-cyano-3-methoxyphenyl)amino}methyl]quinolin-2-yl)oxy]methyl pivalate (20c)

19c (110 mg, 0.26 mmol, 35%, oil) and **20c** (85.0 mg, 0.20 mmol, 27%, oil) were prepared from a mixture of **17c** and **18c (**209.22 mg, 0.73 mmol) using the same procedure as described for **20a**.

(3-[{(4-Cyano-3-methoxyphenyl)amino}methyl]-2-oxoquinolin-1(2*H*)-yl)methyl pivalate (**19c**)

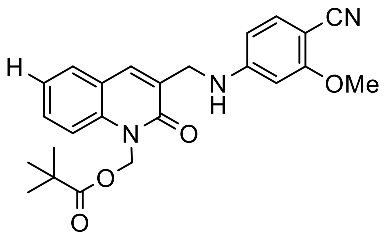

R_f: 0.3

^{1}H-NMR (400 MHz, $CDCl_3$): δ 7.70 (s, 1H), 7.58 – 7.51 (m, 2H), 7.32 (d, J = 8.9 Hz, 1H), 7.27 (m, 2H), 6.35 (s, 2H), 6.23 (dd, J = 8.5, 2.1 Hz, 1H), 6.17 (d, J = 2.0 Hz, 1H), 4.42 (s, 2H), 3.80 (s, 3H), 1.19 (s, 9H). ^{13}C-NMR (101 MHz, $CDCl_3$): δ 177.76 (s), 162.96 (s), 161.72 (s), 152.99 (s), 137.96 (s), 136.92 (s), 134.75 (s), 130.76 (s), 129.03 (s), 128.72 (s), 123.35 (s), 120.18 (s), 118.19 (s), 113.98 (s), 105.09 (s), 95.24 (s), 89.20 (s), 66.22 (s), 55.70 (s), 43.36 (s), 26.99 (s), 26.89 (s).

MS (ESI) calcd for $C_{24}H_{25}N_3O_4$ $[M+H]^+$: 419.48, found: 420.14; M.p. = oil

[(3-[{(4-Cyano-3-methoxyphenyl)amino}methyl]quinolin-2-yl)oxy]methyl pivalate (**20c**)

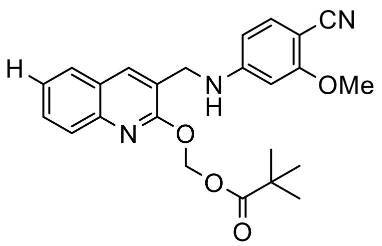

Rf : 0.66

^{1}H-NMR (400 MHz, $CDCl_3$): δ 7.96 (s, 1H), 7.86 (d, J = 8.4 Hz, 1H), 7.69 (dd, J = 8.1, 1.1 Hz, 1H), 7.63 (ddd, J = 8.4, 7.0, 1.5 Hz, 1H), 7.41 (ddd, J = 8.1, 7.0, 1.2 Hz, 1H), 7.25 (d, J = 8.5 Hz, 1H), 6.31 (s, 2H), 6.21 (dd, J = 8.5, 2.1 Hz, 1H), 6.17 (d, J = 2.0 Hz, 1H), 4.47 (s, 2H), 3.80 (s, 3H), 1.21 (s, 9H). ^{13}C-NMR (101 MHz, $CDCl_3$): δ 177.90 (s), 162.91 (s), 157.70 (s), 153.04 (s), 145.33 (s), 136.78 (s), 134.71 (s), 129.65 (s), 127.36 (s), 127.24 (s), 125.66 (s), 125.03 (s), 121.61 (s), 118.30 (s), 105.03 (s), 95.15 (s), 89.04 (s), 82.24 (s), 55.61 (s), 42.54 (s), 38.86 (s), 26.91 (s).

MS (ESI) calcd for $C_{24}H_{25}N_3O_4$ $[M+H]^+$: 419.48, found: 420.05; M.p. = oil

Preparation of (3-[{(4-cyano-3-methoxyphenyl)amino}methyl]-2-oxo-6-(4,4,5,5-tetramethyl-1,3,2-dioxaborolan-2-yl)18uinoline-1(2*H*)-yl)methyl pivalate (21)

21 (133.7 mg, 0.25 mmol, 87%, white solid) was prepared from **19a** (141.40 mg, 0.28 mmol) using the same procedure as described for **14**.

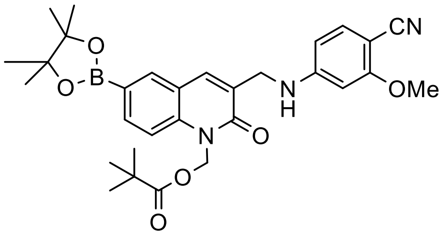

Rf (Hex:AcOEt 6:4): 0.35

^{1}H-NMR (400 MHz, $CDCl_3$): δ 7.99 (d, J = 1.2 Hz, 1H), 7.95 (dd, J = 8.5, 1.4 Hz, 1H), 7.68 (s, 1H), 7.30 (d, J = 8.5 Hz, 2H), 6.37 (s, 2H), 6.22 (dd, J = 8.5, 2.0 Hz, 1H), 6.14 (d, J = 2.0 Hz, 1H), 4.42 (s, 2H), 3.84 (s, 3H), 1.19 (s, J = 5.2 Hz, 10H). ^{13}C-NMR (101 MHz, $CDCl_3$): δ 177.72 (s), 162.99 (s), 161.79 (s), 152.76 (s), 139.95 (s), 137.05 (s), 136.73 (s), 136.20 (s), 134.87 (s), 128.47 (s), 119.55 (s), 118.01 (s), 113.32 (s), 104.98 (s), 95.19 (s), 89.58 (s), 84.23 (s), 66.12 (s), 55.74 (s), 43.36 (s), 24.88 (s).

HRMS (ESI) calcd for $C_{30}H_{36}BN_3O_6$ $[M+H]^+$: 545.44, found: 546.2769; M.p. > 300 °C

Preparation of [(3-[{(4-cyano-3-methoxyphenyl)amino}methyl]-6-(4,4,5,5-tetramethyl-1,3,2-dioxaborolan-2-yl)quinolin-2-yl)oxy]methyl pivalate (22)

22 (166.0 mg, 0.30 mmol, 80%, white solid) was prepared from **20a** (192.50 mg, 0.39 mmol) using the same procedure as described for **14**.

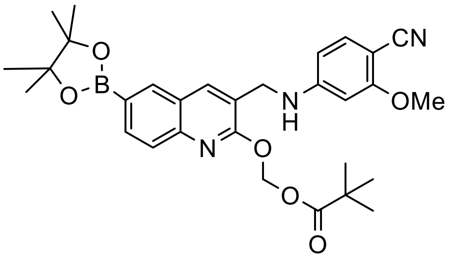

R_f (Hex:AcOEt 6:4): 0.63

^{1}H-NMR (400 MHz, $CDCl_3$): δ 8.22 (s, 1H), 8.03 (dd, J = 8.4, 1.4 Hz, 1H), 7.98 (s, 1H), 7.87 (d, J = 8.4 Hz, 1H), 7.30 (d, J = 8.5 Hz, 1H), 6.31 (s, J = 7.4 Hz, 2H), 6.22 (dd, J = 8.5, 2.1 Hz, 1H), 6.14 (d, J = 2.0 Hz, 1H), 4.49 (s, 2H), 3.85 (s, J = 5.7 Hz, 3H), 1.20 (s, J = 2.1 Hz, 9H). ^{13}C-NMR (101 MHz, $CDCl_3$): δ 177.91 (s), 162.96 (s), 158.43 (s), 152.59 (s), 147.02 (s), 137.48 (s), 135.41 (s), 134.90 (s), 134.86 (s), 126.31 (s), 124.96 (s), 121.42 (s), 117.96 (s), 105.04 (s), 95.16 (s), 89.73 (s), 84.13 (s), 82.36 (s), 55.68 (s), 42.73 (s), 38.87 (s), 24.92 (s).

HRMS (ESI) calcd for $C_{30}H_{36}BN_3O_6$ $[M+H]^+$: 545.44, found: 546.2769; M.p. > 300 °C

Preparation of 2-methoxy-4-[{(2-oxo-1,2-dihydroquinolin-3-yl)methyl}amino]benzonitrile (13c)

2 M NaOH was added to a mixture of **19c** and **20c** (30.0 mg, 71.5 µmol) in MeOH and the resulting suspension was stirred for 72 h (due to incomplete dissolution of the starting material, the reaction did not reach completion as indicated by TLC [Hex:AcOEt 3:2]). After quenching of remaining base with HCl, the mixture was directly purified by column chromatography (Hex: AcOEt 75:25) to afford **13c** (8 mg, 26.2 µmol, 37%) as a white solid.

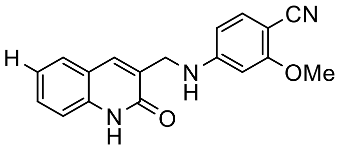

R_f : 0.1

^{1}H-NMR [400 MHz, $(CD_3)_2SO$]: δ 11.92 (s, 1H), 7.77 (s, 1H), 7.63 (dd, J = 7.8, 0.9 Hz, 1H), 7.50 – 7.44 (m, 1H), 7.32 (d, J = 8.1 Hz, 1H), 7.30 (d, J = 8.6 Hz, 1H), 7.19 – 7.13 (m, 2H), 6.36 (d, J = 1.8 Hz, 1H), 6.25 (dd, J = 8.6, 1.8 Hz, 1H), 4.25 (d, J = 5.8 Hz, 2H), 3.80 (s, 3H). ^{13}C-NMR [101 MHz, $(CD_3)_2SO$]: δ 162.86 (s), 162.05 (s), 154.47 (s), 138.44 (s), 136.00 (s), 134.71 (s), 130.45 (s), 130.32 (s), 128.11 (s), 122.38 (s), 119.47 (s), 118.71 (s), 115.36 (s), 105.45 (s), 94.99 (s), 86.50 (s), 56.04 (s), 41.79 (s).

MS (ESI) calcd for $C_{18}H_{15}N_3O_2$ $[M+H]^+$: 305.34, found: 306.15; M.p. = 152 °C

2.2 Preparation of *N*-protected precursors (*S*)- or (*R*)-**29**, *O*-protected precursors (*S*)- or (*R*)-**30**, reference compounds (*S*)- or (*R*)-**2** and protodeboronated impurities (*S*)- or (*R*)-**26c**

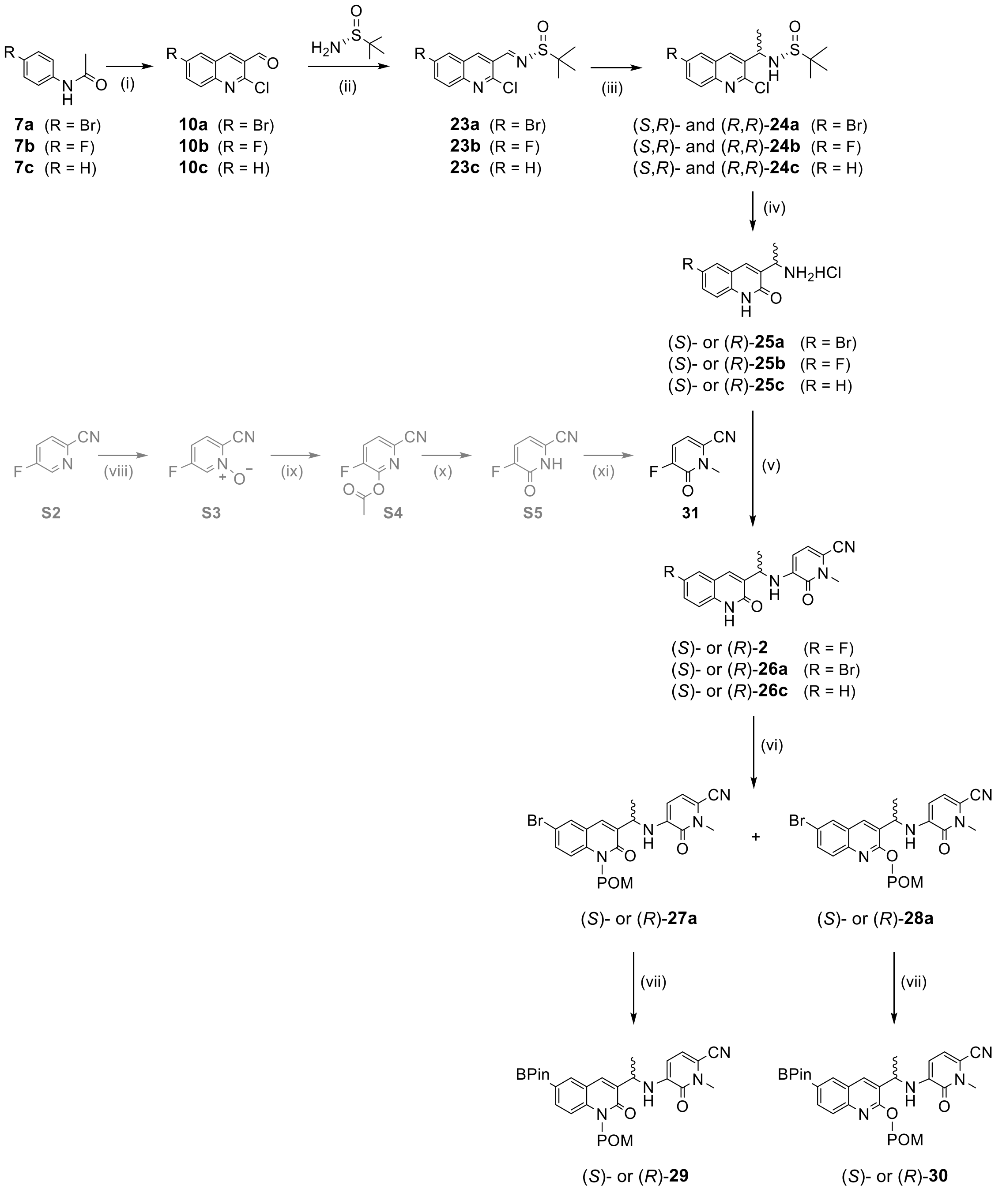

Scheme S2. *Preparation of protected precursors (S)- or (R)-**29** and (S)- or (R)-**30**, reference compounds (S)- and (R)-**2** and protodeboronated impurities (S)- and (R)-**26c***

*Procedures: **i**) triphosgene, DMF, 75 °C, 4 h; **ii**) $CuSO_4$, (R)-2-methylpropane-2-sulfinamide, DCE, 55 °C, 16 h; **iii**) MeMgBr in Et_2O, CH_2Cl_2, -60 °C to rt, 16 h; **iv**) 1 N HCl : dioxane (1:1), reflux, 2 h; **v**) **31**, DIPEA, DMSO, 110 °C, 16 h; **vi**) K_2CO_3, POM-Cl, DMF, rt, 16 h; **vii**) $(Bpin)_2$, KOAc, Pd(dppf)Cl_2, dioxane, 80 °C, 3 h.; viii) TFAA, UHP, CH_2Cl_2, 16 h; ix) Ac_2O, reflux, 16 h; x) K_2CO_3, MeOH, rt, 2 h; xi) K_2CO_3), MeI, DMF, rt, 1 h.*

Preparation of 6-bromo-2-chloroquinoline-3-carbaldehyde (10a)

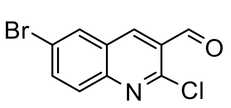

DMF (10.5 mL, 135.38 mmol, 7 eq.) was added dropwise under argon to pre-cooled triphosgene (40.2 g, 135.38 mmol, 7 eq.). After complete addition, **7a** (4.14 g, 19.34 mmol, 1 eq.) was added, the solid mixture was allowed to warm to room temperature and then stirred for 2 h at room temperature followed by 2 h at 75 °C. When TLC analysis (Hex:AcOEt 3:2) of a quenched aliquot indicated complete conversion, the stirred reaction mixture was diluted with H_2O, and the resulting precipitate was recovered by filtration and rinsed with H_2O to afford **10a** (2.1 g, 7.77 mmol, 40%) as an orange solid that could be used without further purification.

R_f : 0.89

^{1}H-NMR [400 MHz, $(CD_3)_2SO$]: δ 10.33 (s, 1H), 8,97 (s, 1H), 8.61 (d, J=2.3 Hz, 1H), 8.12 (dd, J=9.0, 2.3 Hz, 1H), 8.00 (d, J=9.0 Hz, 1H). ^{13}C-NMR [101 MHz, $(CD_3)_2SO$]: δ 189.67 (s), 150.10 (s), 147.65 (s), 140.78 (s), 137.10 (s), 132.46 (s), 130.37 (s), 128.19 (s), 127.56 (s), 121.55 (s).

HRMS (ESI) calcd for $C_{10}H_5BrClNO$ $[M+H]^+$: 270.51, found: 227.12; M.p. = 164 °C

Preparation of 6-fluoro-2-chloroquinoline-3-carbaldehyde (10b)

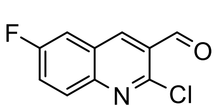

10b (834.0 mg, 3.98 mmol, 41%, white solid) was prepared from **7b** (1.5 g, 9.70 mmol) using the same procedure as described for **10a**.

R_f : 0.81

^{1}H-NMR (400 MHz, $CDCl_3$): δ 10.59 (s, 1H), 8.73 (s, 1H), 8.11 (dd, J = 9.2, 5.1 Hz, 1H), 7.67 (ddt, J = 10.7, 5.4, 2.7 Hz, 1H), 7.62 (dd, J = 8.1, 2.8 Hz, 1H). ^{13}C-NMR (101 MHz, $CDCl_3$): δ 188.94 (s), 161.06 (d, J = 252.0 Hz), 146.88 (s), 139.50 (d, J = 5.7 Hz), 131.20 (d, J = 9.1 Hz), 127.39 (s), 127.29 (s), 126.90 (s), 123.84 (d, J = 25.9 Hz), 112.66 (d, J = 22.3 Hz). ^{19}F-NMR (376 MHz, $CDCl_3$): δ -109.95 (s).

MS (ESI) calcd for $C_{10}H_5ClFNO$ $[M+H]^+$: 209.60, found: 209.0040; M.p. = 172 °C

Preparation of 2-chloroquinoline-3-carbaldehyde (10c)

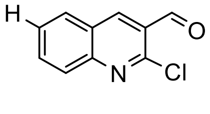

10c (886.3 mg, 4.62 mmol, 42%, white solid) was prepared from **7c**: (1.5 g, 11.09 mmol) using the same procedure as described for **10a**.

R_f : 0.78

^{1}H-NMR [400 MHz, $(CD_3)_2SO$]: δ 10.38 (s, 1H), 8.98 (s, 1H), 8.30 – 8.25 (m, 1H), 8.04 (ddt, *J* = 8.5, 1.4, 0.7 Hz, 1H), 7.98 (ddd, *J* = 10.1, 5.8, 2.4 Hz, 1H), 7.76 (ddd, *J* = 8.1, 6.7, 1.4 Hz,

1H). ^{13}C-NMR [101 MHz, $(CD_3)_2SO$]: δ 189.84 (s), 149.45 (s), 149.02 (s), 141.85 (s), 134.35 (s), 130.68 (s), 128.72 (s), 128.24 (s), 126.82 (s), 126.80 (s).

HRMS (ESI) calcd for $C_{10}H_6ClNO$ $[M+H]^+$: 191.61, found: 191.0132; M.p. = 146 °C

Preparation of (*R*)-*N*-[(6-bromo-2-chloroquinolin-3-yl)methylene]-2-methylpropane-2-sulfinamide (23a)

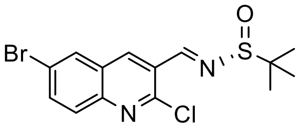

A solution of **10a** (1.00 g, 3.71 mmol, 1 eq.) and anhydrous $CuSO_4$ (888.9 mg, 5.56 mmol, 1.5 eq.) in DCE (7.4 mL, [0.5]) was stirred under argon at 55 °C for 30 min, followed by addition of (*R*)-2-methylpropane-2-sulfinamide (673.9 mg, 5.56 mmol, 1.5 eq.). The reaction mixture was vigorously stirred at 55 °C for 72 h until TLC (Hex:AcOEt 3:2) indicated complete conversion of the starting material, after which the $CuSO_4$ was removed by filtration through a pad of celite and rinsed with chloroform. The filtrate was dried over Na_2SO_4 and concentrated under reduced pressure and the crude product was purified by column chromatography (Hex:AcOEt 95%:5%) to afford **23a** (693 mg, 1.85 mmol, 50%) as an orange solid.

R_f : 0.80

^{1}H-NMR [400 MHz, $(CD_3)_2SO$]: δ 12.33 (s, 1H), 8.78 (d, *J* = 2.8 Hz, 1H), 8.63 (d, *J* = 8.9 Hz, 1H), 8.18 (dd, *J* = 10.7, 2.1 Hz, 1H), 7.75 (td, *J* = 8.9, 2.2 Hz, 1H), 7.30 (dd, *J* = 8.8, 5.6 Hz, 1H). ^{13}C-NMR [101 MHz, $(CD_3)_2SO$]: δ 161.14 (s), 158.34 (s), 139.70 (s), 139.42 (s), 135.49 (s), 132.29 (s), 125.81 (s), 120.74 (s), 117.95 (s), 114.52 (s), 58.19 (d, *J* = 2.1 Hz), 22.59 (s).

HRMS (ESI) calcd for $C_{14}H_{14}BrClN_2OS$ $[M+H]^+$: 373.69, found: 372.9773; M.p. = 220 °C

Preparation of (*R*)-*N*-[(6-fluoro-2-chloroquinolin-3-yl)methylene]-2-methylpropane-2-sulfinamide (23b)

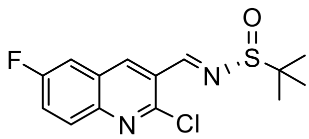

23b (799.4 mg, 2.56 mmol, 75%, white solid) was prepared from **10b** (712.9 mg, 3.40 mmol, 1 eq.) using the same procedure as described for **23a** (reaction time: 16h).

R_f : 0.73

^{1}H-NMR [400 MHz, $(CD_3)_2SO$]: δ 9.10 (s, 1H), 8.89 (s, *J* = 4.5 Hz, 1H), 8.13 – 8.06 (m, 2H), 7.90 – 7.84 (m, 1H), 1.26 (s, *J* = 20.9 Hz, 11H). ^{13}C-NMR [101 MHz, $(CD_3)_2SO$]: δ 160.74 (d, *J* = 247.8 Hz), 159.01 (s), 148.91 (s), 145.73 (s), 139.54 (d, *J* = 5.6 Hz), 131.18 (d, *J* = 9.5 Hz), 128.05 (d, *J* = 11.1 Hz), 126.24 (s), 123.54 (d, *J* = 26.2 Hz), 113.21 (d, *J* = 22.9 Hz), 58.58 (s), 22.63 (s). ^{19}F-NMR (376 MHz, $(CD_3)_2SO$): δ -111.08 (s).

HRMS (ESI) calcd for $C_{14}H_{14}ClFN_2OS$ $[M+H]^+$: 312.19, found: 313.0570; M.p. = 149 °C

Preparation of (*R*)-*N*-[(2-chloroquinolin-3-yl)methylene]-2-methylpropane-2-sulfinamide (23c)

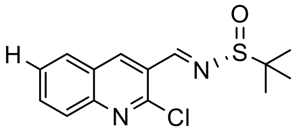

23c (545.2 mg, 1.85 mmol, 80%, white solid) was prepared from **10c**: (445.0 mg, 2.32 mmol, 1 eq.) using the same procedure as described for **23a** (reaction time: 16h).

R_f : 0.68

^{1}H-NMR (400 MHz, $CDCl_3$): δ 9.13 (s, 1H), 8.86 (s, 1H), 8.08 (dd, *J* = 8.5, 0.8 Hz, 1H), 8.00 – 7.94 (m, 1H), 7.85 (ddd, *J* = 8.5, 7.0, 1.4 Hz, 1H), 7.65 (ddd, *J* = 8.1, 5.3, 1.1 Hz, 1H), 1.34 (s, 10H). ^{13}C-NMR (101 MHz, $CDCl_3$): δ 159.25 (s), 150.08 (s), 148.88 (s), 138.84 (s), 132.64 (s), 128.94 (s), 128.60 (s), 127.89 (s), 126.80 (s), 125.76 (s), 58.45 (s), 22.78 (s).

HRMS (ESI) calcd for $C_{14}H_{15}ClN_2OS$ $[M+H]^+$: 294.80, found: 295.0668; M.p. = 151 °C

Preparation of (*R*)-*N*-[(*S*)- and (*R*)-*N*-[(*R*)-1-(6-bromo-2-chloroquinolin-3-yl)ethyl]-2-methylpropane-2-sulfinamide [(*S*,*R*)- and (*R*,*R*)-24a]

A solution of **23a** (693.0 mg, 1.85 mmol, 1 eq.) in CH_2Cl_2 (3.7 mL, [0.5]) was cooled to -60 °C for 15 min under argon before a solution of MeMgBr in Et_2O (925 µL, 3 M, 1.5 eq.) was added. After stirring for 3 h, the reaction mixture was placed in the freezer at -20 °C and left to stand for 72 h, after which TLC (Hex:AcOEt 3:2) indicated almost complete conversion of the starting material. The reaction was then quenched with NH_4Cl and the mixture was extracted with $CHCl_3$. The combined organic phases were dried over Na_2SO_4 and concentrated under reduced pressure and the crude product was purified by column chromatography (Hex:AcOEt 90%:10%) to afford (*S*,*R*)-**24a** (216.0 mg, 0.55 mmol, 30%) and (*R*,*R*)-**24a** (92.0 mg, 0.24 mmol, 13%) as white solids.

(*R*)-*N*-[(*S*)-1-(6-Bromo-2-chloroquinolin-3-yl)ethyl]-2-methylpropane-2-sulfinamide [(*R,S*)-**24a**]

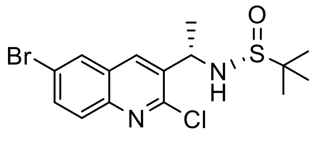

R_f : 0.19

^{1}H-NMR (400 MHz, $CDCl_3$): δ 8.17 (s, 1H), 7.99 (d, *J* = 2.1 Hz, 1H), 7.90 (d, *J* = 9.0 Hz, 1H), 7.81 (dd, *J* = 9.0, 2.2 Hz, 1H), 5.12 (qd, *J* = 6.7, 4.6 Hz, 1H), 3.49 (d, *J* = 4.2 Hz, 1H), 1.71 (d, *J* = 6.7 Hz, 3H), 1.28 (s, 9H). ^{13}C-NMR (101 MHz, $CDCl_3$): δ 150.30 (s), 145.41 (s), 136.53 (s), 135.17

(s), 133.95 (s), 129.97 (s), 129.58 (s), 128.36 (s), 121.20 (s), 56.12 (s), 51.92 (s), 23.45 (s), 22.62 (s).

HRMS (ESI) calcd for $C_{14}H_{14}BrClN_2OS$ [M+H]$^+$: 373.69, found: 389.0085; M.p. = 134 °C

(*R*)-*N*-[(*R*)-1-(6-Bromo-2-chloroquinolin-3-yl)ethyl]-2-methylpropane-2-sulfinamide [(*R*,*R*)-**24a**]

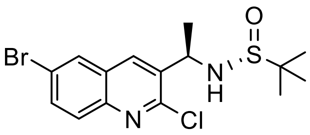

R_f : 0.25

^{1}H-NMR (400 MHz, $CDCl_3$): δ 8.17 (s, 1H), 8.00 (d, *J* = 2.1 Hz, 1H), 7.91 (d, *J* = 9.0 Hz, 1H), 7.81 (dd, *J* = 9.0, 2.2 Hz, 1H), 5.12 (qd, *J* = 6.7, 4.5 Hz, 1H), 3.51 (d, *J* = 4.3 Hz, 1H), 1.71 (d, *J* = 6.7 Hz, 4H), 1.28 (s, 11H). ^{13}C-NMR (101 MHz, $CDCl_3$): δ 150.30 (s), 145.41 (s), 136.52 (s), 135.17 (s), 133.95 (s), 129.96 (s), 129.58 (s), 128.36 (s), 121.20 (s), 56.14 (s), 51.93 (s), 23.44 (s), 22.62 (s).

HRMS (ESI) calcd for $C_{15}H_{18}BrClN_2OS$ [M+H]$^+$: 389.74, found: 389.0085; M.p. = 110.5 °C

Preparation of (*R*)-*N*-[(*S*)- and (*R*)-*N*-[(*R*)-1-(6-fluoro-2-chloroquinolin-3-yl)ethyl]-2-methylpropane-2-sulfinamide [(*S*,*R*)- and (*R*,*R*)-24b]

(*S*,*R*)-**24b** (175.0 mg, 0.53 mmol, 21%, white solid) and (*R*,*R*)-**24b** (29.0 mg, 0.09 mmol, 3%, white solid) were prepared from **23b** (790.0 mg, 2.53 mmol, 1 eq.) using the same procedure as described for (*S*,*R*)- and (*R*,*R*)-**24a** (reaction time: 72 h).

(*R*)-*N*-((*S*)-1-(6-Fluoro-2-chloroquinoline-3-yl)ethyl)-2-methylpropane-2-sulfinamide [(*S*,*R*)-**24b**]

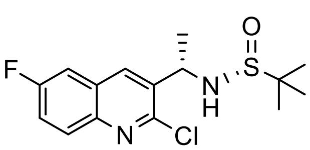

R_f : 0.16

^{1}H-NMR [400 MHz, $(CD_3)_2SO$]: δ 8.48 (s, 1H), 7.97 (dd, *J* = 9.2, 5.3 Hz, 1H), 7.82 (dd, *J* = 9.2, 2.8 Hz, 1H), 7.65 (td, *J* = 8.9, 2.9 Hz, 1H), 5.64 (d, *J* = 6.4 Hz, 1H), 4.84 (p, *J* = 6.6 Hz, 1H), 1.61 (d, *J* = 6.8 Hz, 3H), 1.11 (s, 9H). ^{13}C-NMR [101 MHz, $(CD_3)_2SO$]: δ 160.53 (d, *J* = 246.6 Hz), 149.20 (d, *J* = 2.8 Hz), 143.62 (s), 137.28 (s), 136.90 (d, *J* = 5.2 Hz), 130.81 (d, *J* = 9.5 Hz), 128.34 (d, *J* = 10.8 Hz), 120.96 (d, *J* = 26.1 Hz), 111.60 (d, *J* = 22.2 Hz), 55.85 (s), 52.62 (s), 23.29 (s), 23.08 (s). ^{19}F-NMR [376 MHz, $(CD_3)_2SO$]: δ -112.21 (s, *J* = 13.8 Hz).

HRMS (ESI) calcd for $C_{15}H_{18}ClFN_2OS$ [M+H]$^+$: 328.83, found: 329.0886; M.p. = 114 °C

(*R*)-*N*-((*R*)-1-(6-Fluoro-2-chloroquinoline-3-yl)ethyl)-2-methylpropane-2-sulfinamide [(*R*,*R*)-**24b**]

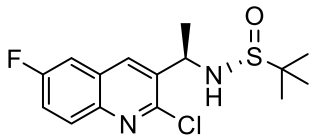

R_f : 0.20

^{1}H-NMR [400 MHz, $(CD_3)_2SO$]: δ 8.55 (s, 1H), 8.04 (dd, *J* = 8.9, 5.3 Hz, 1H), 7.88 (dd, *J* = 8.9, 1.9 Hz, 1H), 7.73 (t, *J* = 7.6 Hz, 1H), 6.08 (d, *J* = 6.8 Hz, 1H), 4.91 – 4.76 (m, 1H), 1.52 (d, *J* = 6.6 Hz, 3H), 1.12 (s, 9H). ^{13}C-NMR [101 MHz, $(CD_3)_2SO$]: δ 160.59 (d, *J* = 246.3 Hz), 148.90 (s), 143.71 (s), 137.90 (s), 137.08 (d, *J* = 5.2 Hz), 130.91 (d, *J* = 9.5 Hz), 128.37 (d, *J* = 10.7 Hz), 121.10 (d, *J* = 26.0 Hz), 111.69 (d, *J* = 22.4 Hz), 55.87 (s), 51.15 (s), 23.13 (s), 22.98 (s). ^{19}F-NMR [376 MHz, $(CD_3)_2SO$]: δ -112.17 (s).

MS (ESI) calcd for $C_{15}H_{18}ClFN_2OS$ $[M+H]^+$: 328.83, found: 329.18; M.p. = 138 °C

Preparation of (*R*)-*N*-[(*S*)- and (*R*)-*N*-[(*R*)-1-(2-chloroquinolin-3-yl)ethyl]-2-methylpropane-2-sulfinamide [(*S*,*R*)- and (*R*,*R*)-24c]

(*S*,*R*)-**24c** (39 mg, 0.13 mmol, 36%, white solid) and (*R*,*R*)-**24c** (22 mg, 0.07 mmol, 20%, white solid) were prepared from **23c (**104 mg, 0.35 mmol, 1 eq.) using the same procedure as described for (*S*,*R*)- and (*R*,*R*)-**24a** (reaction time: 16h).

(*R*)-*N*-((*S*)-1-(2-Chloroquinoline-3-yl)ethyl)-2-methylpropane-2-sulfinamide [(*S*,*R*)-**24c**]

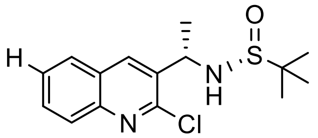

R_f : 0.12

^{1}H-NMR (400 MHz, $CDCl_3$): δ 8.24 (s, 1H), 8.02 (d, *J* = 8.5 Hz, 1H), 7.81 (d, *J* = 8.1 Hz, 1H), 7.73 (ddd, *J* = 8.4, 7.0, 1.3 Hz, 1H), 7.60 – 7.53 (m, 1H), 5.16 – 5.06 (m, 1H), 3.54 (d, *J* = 4.6 Hz, 1H), 1.71 (d, *J* = 6.7 Hz, 3H), 1.25 (s, 9H). ^{13}C-NMR (101 MHz, $CDCl_3$): δ 149.87 (s), 146.85 (s), 136.23 (d, *J* = 7.4 Hz), 135.30 (s), 130.52 (d, *J* = 5.9 Hz), 128.26 (s), 127.52 (s), 127.28 (s), 127.24 (s), 56.09 (s), 52.14 (s), 23.46 (s), 22.62 (s, *J* = 42.2 Hz).

HRMS (ESI) calcd for $C_{15}H_{19}ClN_2O_2S$ $[M+H]^+$: 310.84, found: 311.0977; M.p. = oil

(*R*)-*N*-((*R*)-1-(2-Chloroquinoline-3-yl)ethyl)-2-methylpropane-2-sulfinamide [(*R*,*R*)-**24c**]

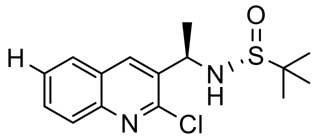

R_f : 0.20

^{1}H-NMR (400 MHz, $CDCl_3$): δ 8.25 (s, 1H), 8.04 (dd, *J* = 8.1, 0.5 Hz, 1H), 7.84 (dd, *J* = 8.2, 1.1 Hz, 1H), 7.75 (ddd, *J* = 8.5, 7.0, 1.4 Hz, 1H), 7.59 (ddd, *J* = 8.1, 7.0, 1.2 Hz, 1H), 5.16 – 5.07 (m, 1H), 3.49 (d, *J* = 4.4 Hz, 1H), 1.73 (d, *J* = 6.7 Hz, 4H), 1.27 (s, 9H). ^{13}C-NMR (101 MHz, $CDCl_3$): δ 149.90 (s), 146.88 (s), 136.24 (s), 135.30 (s), 130.50 (s), 128.30 (s), 127.52 (s), 127.29 (s, *J* = 4.0 Hz), 127.25 (s), 56.09 (s), 52.17 (s), 23.47 (s), 22.62 (s).

HRMS (ESI) calcd for $C_{15}H_{19}ClN_2O_2S$ $[M+H]^+$: 310.84, found: 311.0977; M.p. = oil

Preparation of (*S*)-3-(1-aminoethyl)-6-bromoquinolin-2(1*H*)-one hydrochloride [(*S*)-25a]

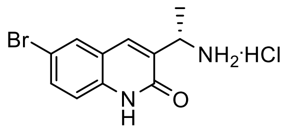

A solution of (*S*,*R*)-**24a** (245.0 mg, 0.63 mmol) in dioxane and 1 N HCl (1:1, 3.1 mL, [0.2]) was stirred at 100 °C for 4 h until TLC (Hex:AcOEt 3:2) indicated complete conversion of the starting material. After removal of the solvent and drying of the resulting residue under reduced pressure overnight, the crude product was characterized by mass spectrometry and used without further purification.

R_f : 0.0

MS (ESI) calcd for $C_{11}H_{12}BrClN_2O$ $[M+H]^+$: 303.58, found: 268.07.

Preparation of (*R*)-3-(1-aminoethyl)-6-bromoquinolin-2(1*H*)-one hydrochloride [(*R*)-25a]

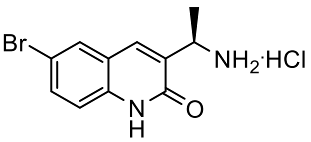

(*R*)-**25a** was prepared from (*R*,*R*)-**24a** (103.0 mg, 0.26 mmol) using the same procedure as described for (*S*)-**25a**.

R_f : 0.0

MS (ESI) calcd for $C_{11}H_{12}BrClN_2O$ $[M+H]^+$: 303.58, found: 268.07.

Preparation of (*S*)-3-(1-aminoethyl)-6-fluoroquinolin-2(1*H*)-one hydrochloride [(*S*)-25b]

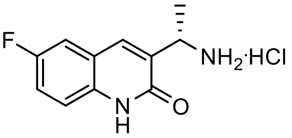

(*S*)-**25b** was prepared from (*S*,*R*)-**24b** (334.7 mg, 1.02 mmol) using the same procedure as described for (*S*)-**25a** (reaction time: 2 h).

R_f : 0.0

MS (ESI) calcd for $C_{11}H_{12}Cl_2FN_2O$ $[M+H]^+$: 242.68, found: 207.30.

Preparation of (*R*)-3-(1-aminoethyl)-6-fluoroquinolin-2(1*H*)-one hydrochloride [(*R*)-25b]

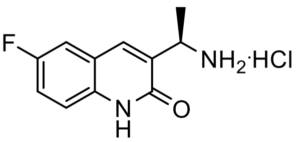

(*R*)-**25b** was prepared from (*R*,*R*)-**24b** (167.0 mg, 0.51 mmol) using the same procedure as described for (*S*)-**25a** (reaction time: 2 h).

R_f : 0.0

MS (ESI) calcd for $C_{11}H_{12}Cl_2FN_2O$ $[M+H]^+$: 224.69, found: 207.30.

Preparation of (*S*)-3-(1-aminoethyl)-quinolin-2(1*H*)-one hydrochloride [(*S*)-25c]

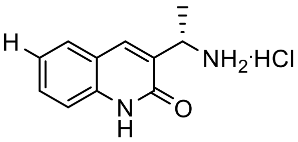

(*S*)-**25c** was prepared from (*S*,*R*)-**24c** (144 mg, 0.46 mmol) using the same procedure as described for (*S*)-**25a** (reaction time: 7 h).

R_f : 0.0

MS (ESI) calcd for $C_{11}H_{13}ClN_2O$ $[M+H]^+$: 224.69, found: 189.34.

Preparation of (*R*)-3-(1-aminoethyl)-quinolin-2(1*H*)-one hydrochloride [(*R*)-25c]

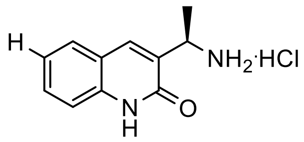

(*R*)-**25c** was prepared from (*R*,*R*)-**24c** (219 mg, 0.70 mmol) using the same procedure as described for (*S*)-**25a** (reaction time: 7 h).

R_f : 0.0

MS (ESI) calcd for $C_{11}H_{13}ClN_2O$ $[M+H]^+$: 224.69, found: 189.34.

Preparation of (*S*)-5-[{1-(6-bromo-2-oxo-1,2-dihydroquinoline-3-yl)ethyl}amino]-1-methyl-6-oxo-1,6-dihydropyridine-2-carbonitrile [(*S*)-26a]

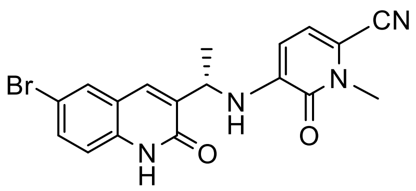

N,*N*-Diisopropylethylamine (224 µL, 1.29 mmol, 3 eq.) was added under argon to a solution of (*S*)-**25a** (116.0 mg, 0.43 mmol, 1 eq.) and 5-fluoro-1-methyl-6-oxo-1,6-dihydropyridine-2-carbonitrile (**31**, 73.0 mg, 0.48 mmol, 1.1 eq.) in DMSO (4.3 mL, [0.1]). The reaction mixture was stirred at 110 °C for 16 h until TLC (Hex:AcOEt 1:4) indicated complete conversion of the starting material. The reaction mixture was allowed to cool to room temperature, after which it was diluted with AcOEt and remaining *N*,*N*-diisopropylethylamine quenched by addition of H_2O. The organic phase was separated, washed with brine, dried over Na_2SO_4 and concentrated under reduced pressure. The crude product was purified by column chromatography to afford (*S*)-**26a** (96.0 mg, 0.24 mmol, 56%) as a white oil.

R_f : 0.58

^{1}H-NMR (400 MHz, $CDCl_3$): δ 12.53 (s, 1H), 7.61 (d, J = 2.0 Hz, 1H), 7.59 (s, 1H), 7.54 (dd, J = 8.7, 2.0 Hz, 1H), 7.32 (d, J = 8.7 Hz, 1H), 6.67 (d, J = 7.8 Hz, 1H), 6.36 (d, J = 6.6 Hz, 1H), 5.91 (d, J = 7.9 Hz, 1H), 4.84 (p, J = 6.5 Hz, 1H), 3.73 (s, J = 8.4 Hz, 3H), 1.64 (d, J = 6.6 Hz, 3H). ^{13}C-NMR (101 MHz, $CDCl_3$): δ 162.90 (s), 156.80 (s), 141.25 (s), 140.61 (s), 136.66 (s), 134.42 (s), 133.95 (s), 133.18 (s), 129.95 (s), 121.16 (s), 119.40 (s), 117.44 (s), 115.25 (s), 114.72 (s), 105.76 (s), 104.50 (s), 47.76 (s), 40.94 (s), 34.65 (s).

MS (ESI) calcd for $C_{15}H_{18}BrClN_2OS$ $[M+H]^+$: 399.25, found: 399.01; M.p. = oil

Preparation of (*R*)-5-[{1-(6-bromo-2-oxo-1,2-dihydroquinoline-3-yl)ethyl}amino]-1-methyl-6-oxo-1,6-dihydropyridine-2-carbonitrile [(*R*)-26a]

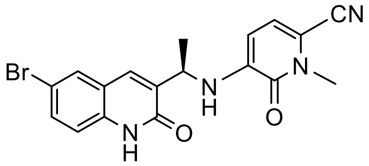

(*R*)-**26a** (64.6 mg, 0.16 mmol, 50%, white oil) was prepared from (*R*)-**25a** (85.0 mg, 0.32 mmol, 1 eq.) using the same procedure as described for (*S*)-**26a**.

R_f : 0.58

^{1}H-NMR (400 MHz, $CDCl_3$): δ 11.94 (s, 1H), 8.24 (d, J = 7.3 Hz, 1H), 7.68 (d, J = 2.1 Hz, 1H), 7.63 – 7.59 (m, 2H), 7.31 (d, J = 8.7 Hz, 1H), 6.93 (d, J = 7.3 Hz, 1H), 6.70 (d, J = 7.8 Hz, 1H), 6.29 (d, J = 6.6 Hz, 1H), 5.93 (d, J = 7.9 Hz, 1H), 4.88 (p, J = 6.6 Hz, 1H), 3.77 (d, J = 6.1 Hz, 3H), 1.68 (d, J = 6.7 Hz, 4H). ^{13}C-NMR (101 MHz, $CDCl_3$): δ 162.90 (s), 156.80 (s), 141.25 (s), 136.66 (s), 134.42 (s), 133.18 (s), 129.95 (s), 121.16 (s), 119.40 (s), 117.44 (s), 115.25 (s), 114.72 (s), 113.18 (s), 105.76 (s), 104.50 (s), 47.76 (s), 40.94 (s), 34.65 (s).

MS (ESI) calcd for $C_{15}H_{18}BrClN_2OS$ $[M+H]^+$: 399.25, found: 399.03; M.p. = oil

Preparation of (*S*)-5-[{1-(6-fluoro-2-oxo-1,2-dihydroquinoline-3-yl)ethyl}amino]-1-methyl-6-oxo-1,6-dihydropyridine-2-carbonitrile [(*S*)-2]

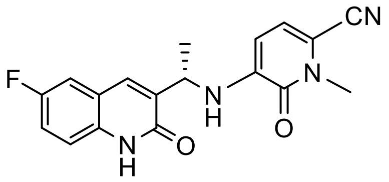

(*S*)-**2** (72.0 mg, 0.21 mmol, 20%, white solid) was prepared from (*S*)-**25b** (247.53 mg, 1.02 mmol, 1 eq.) using the same procedure as described for (*S*)-**26a**.

R_f : 0.39

α_D: +86.6 (0.187 g/100mL)

^{1}H-NMR (400 MHz, $CDCl_3$): δ 12.70 (s, 1H), 7.65 (s, 1H), 7.47 (dd, J = 9.0, 4.6 Hz, 1H), 7.27 (td, J = 8.5, 2.7 Hz, 1H), 7.20 (dd, J = 8.5, 2.7 Hz, 1H), 6.70 (d, J = 7.8 Hz, 1H), 6.37 (d, J = 6.7 Hz, 1H), 5.95 (d, J = 7.9 Hz, 1H), 4.89 (p, J = 6.6 Hz, 1H), 3.76 (s, 3H), 1.67 (d, J = 6.7 Hz, 3H). ^{13}C-NMR (101 MHz, $CDCl_3$): δ 161.20 (d, J = 365.0 Hz), 156.97 (s), 156.85 (s), 141.29 (s), 134.44 (d, J = 3.3 Hz), 134.36 (s, J = 7.3 Hz), 134.29 (s), 120.40 (d, J = 9.0 Hz), 119.39 (s), 118.82 (d, J = 24.6 Hz), 117.46 (d, J = 8.3 Hz), 114.73 (s), 112.54 (d, J = 22.7 Hz), 105.82 (s), 104.45 (s), 47.82 (s), 34.66 (s), 21.42 (s). ^{19}F-NMR (376 MHz, $CDCl_3$): δ -119.13 (s).

HRMS (ESI) calcd for $C_{18}H_{15}ClFN_4O_2$ $[M+H]^+$: 338.12, found: 339.1251; M.p. = 218°C

Preparation of (*R*)-5-[{1-(6-fluoro-2-oxo-1,2-dihydroquinoline-3-yl)ethyl}amino]-1-methyl-6-oxo-1,6-dihydropyridine-2-carbonitrile [(*R*)-2]

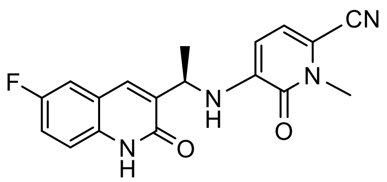

(*R*)-**2** (60.2 mg, 0.18 mmol, 35%, white solid) was prepared from (*R*)-**25b** (123.03 mg, 0.51 mmol, 1 eq.) using the same procedure as described for (*S*)-**26a**.

R_f : 0.39

α_D: -63.7 (0.124 g/100mL)

^{1}H-NMR [400 MHz, $(CD_3)_2SO$]: δ 12.00 (s, 1H), 7.77 (s, 1H), 7.50 (dd, J = 9.2, 2.6 Hz, 1H), 7.44 – 7.30 (m, 2H), 6.97 (d, J = 7.8 Hz, 1H), 6.94 (d, J = 7.7 Hz, 1H), 5.97 (d, J = 8.1 Hz, 1H), 4.70 (p, J = 6.7 Hz, 1H), 3.59 (s, 3H), 3.33 (s, J = 8.7 Hz, 3H). ^{13}C-NMR [101 MHz, $(CD_3)_2SO$]: δ 160.01 (d, J = 270.3 Hz), 156.38 (s), 156.30 (s), 141.90 (s), 135.54 (s), 135.14 (s), 134.13 (d, J = 3.1 Hz), 120.15 (d, J = 9.1 Hz), 119.90 (s), 118.52 (d, J = 24.5 Hz), 117.18 (d, J = 8.5 Hz), 115.63 (s), 113.00 (d, J = 22.8 Hz), 105.01 (s), 104.11 (s), 47.83 (s), 34.54 (s), 20.86 (s). ^{19}F-NMR [376 MHz, $(CD_3)_2SO$]: δ -120.87 (s).

HRMS (ESI) calcd for $C_{18}H_{15}ClFN_4O_2$ $[M+H]^+$: 338.12, found: 339.1251; M.p. = 218°C

Preparation of (*S*)-5-[{1-(2-oxo-1,2-dihydroquinoline-3-yl)ethyl}amino]-1-methyl-6-oxo-1,6-dihydropyridine-2-carbonitrile [(*S*)-26c]

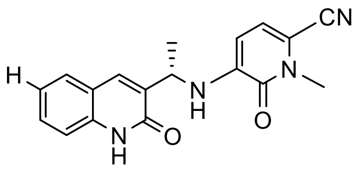

(*S*)-**26c** (40.0 mg, 0.12 mmol, 26%, white oil) was prepared from (*S*)-**25c** (105.8 mg, 0.47 mmol, 1 eq.) using the same procedure as described for (*S*)-**26a**.

R_f : 0.57

α_D: not determined

^{1}H-NMR (400 MHz, $CDCl_3$): δ 12.57 (s, 1H), 7.73 (s, 1H), 7.58 – 7.45 (m, 3H), 7.23 (ddd, *J* = 8.1, 7.0, 1.3 Hz, 1H), 6.70 (d, *J* = 7.8 Hz, 1H), 6.38 (d, *J* = 6.7 Hz, 1H), 5.99 (d, *J* = 8.1 Hz, 1H), 4.94 (p, *J* = 6.6 Hz, 1H), 3.77 (s, 3H), 1.70 (d, *J* = 6.7 Hz, 3H). ^{13}C-NMR (101 MHz, $CDCl_3$): δ 163.37 (s), 156.89 (s), 141.40 (s), 137.76 (s), 135.29 (s), 133.04 (s), 130.48 (s), 127.84 (s), 122.91 (s), 119.83 (s), 119.52 (s), 115.79 (s), 114.81 (s), 105.64 (s), 104.51 (s), 47.68 (s), 34.65 (s), 21.53 (s).

HRMS (ESI) calcd for $C_{18}H_{16}N_4O_2$ $[M+H]^+$: 320.25, found: 321.1353; M.p. = oil

Preparation of (*R*)-5-[{1-(2-oxo-1,2-dihydroquinoline-3-yl)ethyl}amino]-1-methyl-6-oxo-1,6-dihydropyridine-2-carbonitrile [(*R*)-26c]

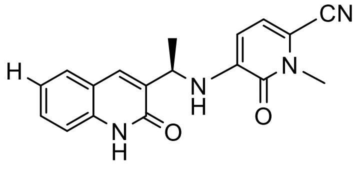

(*R*)-**26c** (60.2 mg, 0.18 mmol, 35%, white oil) was prepared from (*R*)-**25c** (123.03 mg, 0.51 mmol, 1 eq.) using the same procedure as described for (*S*)-**26a**.

R_f : 0.57

α_D: not determined

^{1}H-NMR (400 MHz, $CDCl_3$): δ 12.57 (s, 1H), 7.73 (s, 1H), 7.58 – 7.45 (m, 3H), 7.23 (ddd, *J* = 8.1, 7.0, 1.3 Hz, 1H), 6.70 (d, *J* = 7.8 Hz, 1H), 6.38 (d, *J* = 6.7 Hz, 1H), 5.99 (d, *J* = 8.1 Hz,

1H), 4.94 (p, J = 6.6 Hz, 1H), 3.77 (s, 3H), 1.70 (d, J = 6.7 Hz, 3H). ^{13}C-NMR (101 MHz, $CDCl_3$): δ 163.37 (s), 156.89 (s), 141.40 (s), 137.76 (s), 135.29 (s), 133.04 (s), 130.48 (s), 127.84 (s), 122.91 (s), 119.83 (s), 119.52 (s), 115.79 (s), 114.81 (s), 105.64 (s), 104.51 (s), 47.68 (s), 34.65 (s), 21.53 (s).

HRMS (ESI) calcd for $C_{18}H_{16}N_4O_2$ $[M+H]^+$: 320.25, found: 321.1353; M.p. = oil

Preparation of (*S*)-((6-bromo-3-(1-((6-cyano-1-methyl-2-oxo-1,2-dihydropyridin-3-yl)amino)ethyl)quinolin-2-yl)oxy)methyl pivalate and (*S*)-(6-bromo-3-(1-((6-cyano-1-methyl-2-oxo-1,2-dihydropyridin-3-yl)amino)ethyl)-2-oxoquinolin-1(2*H*)-yl)methyl pivalate [(*S*)-27a and (*S*)-28a]

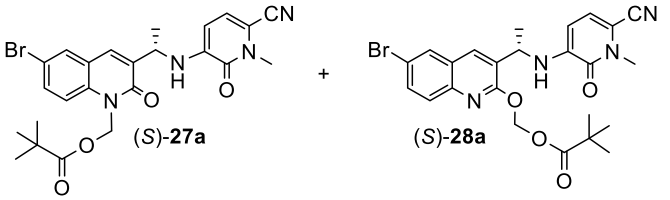

A solution of (*S*)-**26a** (203.0 mg, 0.51 mmol, 1 eq.) and K_2CO_3 (105.7 mg, 0.76 mmol, 1.5 eq.) in DMF (2.5 mL, [0.2]) was vigorously stirred under argon at 60 °C for 15 min. After cooling to room temperature, POM-Cl (109 µL, 0.76 mmol, 1.5 eq.) was added and the mixture was stirred for 16 h until TLC (Hex: AcOEt 3:2) indicated complete consumption of the starting material and presence of two products. The reaction mixture was diluted with saturated NH_4Cl and extracted with EtOAc, after which the combined organic fractions were dried over Na_2SO_4 and concentrated under reduced pressure. The crude product was purified by column chromatography to afford a mixture of (*S*)-**27a** and (*S*)-**28a** (221 mg, 0.43 mmol, 84%) as yellow solids.

R_f: 0.61 [(*S*)-**27a**] / 0.77 [(*S*)-**28a**]

^{1}H-NMR (400 MHz, $CDCl_3$): δ 7.83 (d, J = 2.0 Hz, 1H), 7.80 (s, 1H), 7.74 (d, J = 8.9 Hz, 1H), 7.70 (dd, J = 8.9, 2.1 Hz, 1H), 7.66 (d, J = 2.2 Hz, 1H), 7.63 (dd, J = 8.9, 2.3 Hz, 1H), 7.51 (s, 1H), 7.22 (d, J = 8.9 Hz, 1H), 6.70 (d, J = 7.8 Hz, 1H), 6.63 (d, J = 7.8 Hz, 1H), 6.34 (dd, J = 11.3, 5.3 Hz, 4H), 6.20 (d, J = 6.1 Hz, 1H), 6.13 (d, J = 6.4 Hz, 1H), 5.90 (d, J = 8.0 Hz, 1H), 5.75 (d, J = 7.9 Hz, 1H), 4.88 – 4.77 (m, 2H), 3.77 (d, J = 1.2 Hz, 6H), 1.63 (d, J = 6.6 Hz, 8H), 1.63 (d, J = 6.7 Hz, 7H), 1.23 (s, 2H), 1.22 (s, 11H).

HRMS (ESI) calcd for $C_{24}H_{25}BrN_4O_4$ $[M+H]^+$: 513.39, found: 513.1135; M.p.: not measured

Preparation of (*R*)-((6-bromo-3-(1-((6-cyano-1-methyl-2-oxo-1,2-dihydropyridin-3-yl)amino)ethyl)quinolin-2-yl)oxy)methyl pivalate and (*R*)-(6-bromo-3-(1-((6-cyano-1-methyl-2-oxo-1,2-dihydropyridin-3-yl)amino)ethyl)-2-oxoquinolin-1(2*H*)-yl)methyl pivalate [(*R*)-27a and (*R*)-28a]

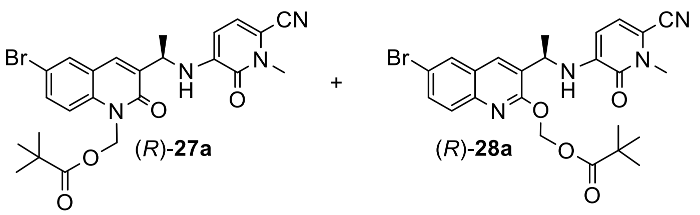

A mixture of (*R*)-**27a** and (*R*)-**28a** (74 mg, 0.13 mmol, 99%, yellow solid with traces of DMF) was prepared from (*R*)-**26a** (58 mg, 0.14 mmol) using the same procedure as described for (*S*)-**27a** and (*S*)-**28a**.

R_f: 0.61 [(*R*)-**27a**] / 0.77 [(*R*)-**28a**]

^{1}H-NMR (400 MHz, $CDCl_3$): δ 7.81 (d, J = 2.0 Hz, 1H), 7.80 (s, 1H), 7.72 (d, J = 8.9 Hz, 1H), 7.68 (dd, J = 8.9, 2.1 Hz, 1H), 7.64 (d, J = 2.2 Hz, 1H), 7.61 (dd, J = 8.9, 2.3 Hz, 1H), 7.51 (s, 1H), 7.21 (d, J = 8.9 Hz, 1H), 6.69 (d, J = 7.8 Hz, 1H), 6.62 (d, J = 7.8 Hz, 1H), 6.33 (dd, J = 11.2, 5.3 Hz, 3H), 6.22 (d, J = 6.2 Hz, 1H), 6.14 (d, J = 7.5 Hz, 1H), 5.90 (d, J = 7.9 Hz, 1H), 5.74 (d, J = 7.9 Hz, 1H), 4.87 – 4.76 (m, 2H), 3.75 (s, 2H), 3.75 (s, 2H), 1.62 (d, J = 6.7 Hz, 6H), 1.21 (s, 9H), 1.21 (s, 5H).

HRMS (ESI) calcd for $C_{24}H_{25}BrN_4O_4$ $[M+H]^+$: 513.39, found: 513.1135; M.p.: not measured

Preparation of (*S*)-(3-(1-((6-cyano-1-methyl-2-oxo-1,2-dihydropyridin-3-yl)amino)ethyl)-2-oxo-6-(4,4,5,5-tetramethyl-1,3,2-dioxaborolan-2-yl)quinolin-1(2*H*)-yl)methyl pivalate and (*S*)-((3-(1-((6-cyano-1-methyl-2-oxo-1,2-dihydropyridin-3-yl)amino)ethyl)-6-(4,4,5,5-tetramethyl-1,3,2-dioxaborolan-2-yl)quinolin-2-yl)oxy)methyl pivalate [(*S*)-29 and (*S*)-30]

A solution of the mixture of (*S*)-**27a** and (*S*)-**28a** (221 mg, 0.43 mmol, 1 eq.), bis(pinacolato)diboron (132.0 mg, 0.52 mmol, 1.2 eq.), KOAc (295.4 mg, 3.0 mmol, 7 eq.) and Pd(dppf)Cl_2 (17.6 mg, 0.15 mmol, 5%) in dioxane was stirred at 80 °C under argon for 4.5 h until mass spectrometry indicated complete consumption of the starting material. The reaction mixture was diluted with EtOAc, washed with H_2O and brine, and the organic phase was dried over Na_2SO_4 and concentrated under reduced pressure. The crude products were purified by column chromatography (Hexane: AcOEt 60%:40%) to afford (*S*)-**29** (60 mg, 0.10 mmol, 23%) and (*S*)-**30** (76 mg, 0.13 mmol, 30%) as white solids.

(*S*)-((3-(1-((6-cyano-1-methyl-2-oxo-1,2-dihydropyridin-3-yl)amino)ethyl)-6-(4,4,5,5-tetramethyl-1,3,2-dioxaborolan-2-yl)quinolin-2-yl)oxy)methyl pivalate **[**(*S*)-**29**]

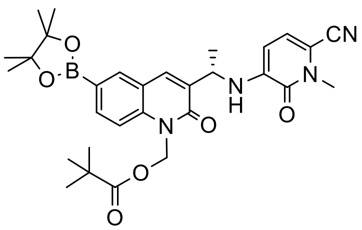

Yield: 23% (60 mg, 0.10 mmol)

α_D: -53.7 (0.095 g/100mL)

R_f: 0.44

^{1}H-NMR (400 MHz, $CDCl_3$): δ 7.97 (d, *J* = 1.2 Hz, 1H), 7.94 (dd, *J* = 8.5, 1.4 Hz, 1H), 7.59 (s, 1H), 7.29 (d, *J* = 8.4 Hz, 1H), 6.68 (d, *J* = 7.8 Hz, 1H), 6.43 – 6.33 (m, 2H), 6.13 (d, *J* = 6.5 Hz, 1H), 5.89 (d, *J* = 7.9 Hz, 1H), 4.88 – 4.80 (m, 1H), 3.78 (s, *J* = 2.5 Hz, 3H), 1.64 (d, *J* = 6.6 Hz, 5H), 1.36 (s, 9H), 1.22 (s, 9H). ^{13}C-NMR (101 MHz, $CDCl_3$): δ 177.78 (s), 161.31 (s), 156.75 (s), 141.15 (s), 140.01 (s), 136.66 (s), 136.38 (s), 135.22 (s), 132.42 (s), 119.58 (s), 119.30 (s), 114.74 (s), 113.24 (s), 105.85 (s), 104.61 (s), 84.19 (s), 66.38 (s), 47.88 (s), 34.60 (s), 29.70 (s), 27.02 (s), 24.90 (s), 24.83 (s), 21.28 (s).

HRMS (ESI) calcd for $C_{30}H_{37}BN_4O_6$ $[M+H]^+$: 560.46, found: 561.2878 ; M.p. = amorphic solid

(*S*)-(3-(1-((6-cyano-1-methyl-2-oxo-1,2-dihydropyridin-3-yl)amino)ethyl)-2-oxo-6-(4,4,5,5-tetramethyl-1,3,2-dioxaborolan-2-yl)quinolin-1(2*H*)-yl)methyl pivalate [(*S*)-**30**]

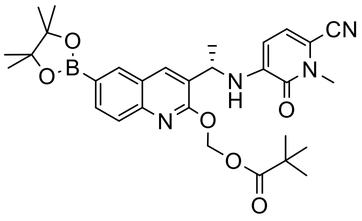

α_D: -0.119.8 (0.086 g/100mL)

R_f: 0.63

^{1}H-NMR (400 MHz, $CDCl_3$): δ 8.18 (d, *J* = 0.9 Hz, 1H), 8.00 (dt, *J* = 11.2, 5.6 Hz, 1H), 7.88 (s, 1H), 7.82 (dd, *J* = 12.9, 5.9 Hz, 1H), 6.60 (d, *J* = 7.8 Hz, 1H), 6.38 (d, *J* = 5.3 Hz, 1H), 6.35 (d, *J* = 5.3 Hz, 1H), 6.21 (d, *J* = 6.4 Hz, 1H), 5.74 (d, *J* = 8.0 Hz, 1H), 4.82 (p, *J* = 6.6 Hz, 1H), 3.78 (s, 3H), 1.63 (d, *J* = 6.7 Hz, 5H), 1.38 (s, 8H), 1.22 (s, 9H). ^{13}C-NMR (101 MHz, $CDCl_3$): δ 177.56 (s), 157.96 (s), 156.75 (s), 147.06 (s), 141.16 (s), 135.59 (s), 135.20 (s), 134.71 (s), 126.33 (s), 125.53 (s), 125.22 (s), 119.16 (s), 114.71 (s), 105.85 (s), 104.60 (s), 84.06 (s), 81.98 (s), 47.48 (s), 34.60 (s), 26.92 (s), 24.92 (s), 24.87 (s), 21.99 (s).

HRMS (ESI) calcd for $C_{30}H_{37}BN_4O_6$ $[M+H]^+$: 560.46, found: 561.2878; M.p. = amorphic solid

Preparation of (*R*)-(3-(1-((6-cyano-1-methyl-2-oxo-1,2-dihydropyridin-3-yl)amino)ethyl)-2-oxo-6-(4,4,5,5-tetramethyl-1,3,2-dioxaborolan-2-yl)quinolin-1(2*H*)-yl)methyl pivalate and (*R*)-((3-(1-((6-cyano-1-methyl-2-oxo-1,2-dihydropyridin-3-yl)amino)ethyl)-6-(4,4,5,5-tetramethyl-1,3,2-dioxaborolan-2-yl)quinolin-2-yl)oxy)methyl pivalate [(*R*)-29 and (*R*)-30]

(*R*)-**29** (8.1 mg, 14 µmol, 10%, white solid) and (*R*)-**30** (19 mg, 34 µmol, 23%, white solid) were prepared from the mixture of (*R*)-**27a** and (*R*)-**28a** (74 mg, 0.14 mmol, 1 eq.) using the same procedure as described for (*S*)-**29** and (*S*)-**30**.

(*R*)-((3-(1-((6-cyano-1-methyl-2-oxo-1,2-dihydropyridin-3-yl)amino)ethyl)-6-(4,4,5,5-tetramethyl-1,3,2-dioxaborolan-2-yl)quinolin-2-yl)oxy)methyl pivalate [(*R*)-**29**]

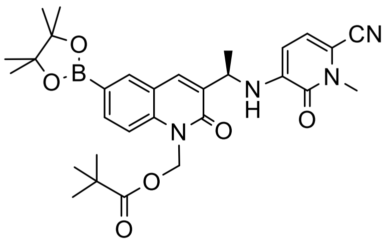

α_D: +33.8 (0.154 g/100mL

R_f: 0.44

^{1}H-NMR (400 MHz, $CDCl_3$): δ 7.97 (d, *J* = 1.1 Hz, 1H), 7.94 (dd, *J* = 8.5, 1.4 Hz, 1H), 7.59 (s, 1H), 7.29 (d, *J* = 7.6 Hz, 2H), 6.68 (d, *J* = 7.8 Hz, 1H), 6.43 – 6.33 (m, 2H), 6.13 (d, *J* = 6.4 Hz, 1H), 5.89 (d, *J* = 7.9 Hz, 1H), 4.84 (p, *J* = 6.4 Hz, 1H), 3.78 (s, 3H), 1.64 (d, *J* = 6.6 Hz, 4H), 1.36 (s, 11H), 1.22 (s, 8H). ^{13}C-NMR (101 MHz, $CDCl_3$): δ 177.81 (s), 161.32 (s), 156.74 (s), 141.13 (s), 140.00 (s), 136.67 (s), 136.39 (s), 135.23 (s), 132.39 (s), 119.58 (s), 119.34 (s), 114.76 (s), 113.25 (s), 104.64 (s), 84.20 (s), 66.39 (s), 47.88 (s), 34.63 (s), 27.02 (s), 24.91 (s), 24.83 (s), 21.28 (s).

HRMS (ESI) calcd for $C_{30}H_{37}BN_4O_6$ $[M+H]^+$: 560.46, found: 561.2878; M.p. = amorphic solid

(*R*)-(3-(1-((6-cyano-1-methyl-2-oxo-1,2-dihydropyridin-3-yl)amino)ethyl)-2-oxo-6-(4,4,5,5-tetramethyl-1,3,2-dioxaborolan-2-yl)quinolin-1(2*H*)-yl)methyl pivalate [(*R*)-**30**]

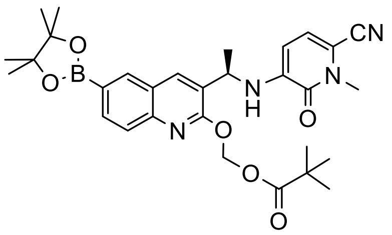

α_D: +76.9 (0.117 g/100mL)

R_f: 0.63

^{1}H-NMR (400 MHz, $CDCl_3$): δ 8.18 (s, 1H), 8.01 (dd, *J* = 8.4, 1.3 Hz, 1H), 7.88 (s, 1H), 7.84 (d, *J* = 8.4 Hz, 1H), 6.60 (d, *J* = 7.8 Hz, 1H), 6.38 (d, *J* = 5.3 Hz, 1H), 6.34 (d, *J* = 5.3 Hz, 1H), 6.21 (d, *J* = 6.3 Hz, 1H), 5.73 (d, *J* = 7.9 Hz, 1H), 4.82 (p, *J* = 6.5 Hz, 1H), 3.77 (s, 3H), 1.63 (d, *J* = 6.7 Hz, 3H), 1.37 (s, 12H), 1.22 (s, 9H). ^{13}C-NMR (101 MHz, $CDCl_3$): δ 177.57 (s), 157.97 (s), 156.74 (s), 147.05 (s), 141.15 (s), 135.60 (s), 135.21 (s), 134.70 (s), 126.33 (s), 125.53 (s), 125.22 (s), 119.18 (s), 114.73 (s), 105.83 (s), 104.62 (s), 84.06 (s), 81.96 (s), 47.48 (s), 38.85 (s), 34.61 (s), 26.92 (s), 24.90 (d, *J* = 5.6 Hz), 21.99 (s).

HRMS (ESI) calcd for $C_{30}H_{37}BN_4O_6$ $[M+H]^+$: 560.46, found: 561.2878; M.p. = amorphic solid

Preparation of 2-cyano-5-fluoropyridine 1-oxide (S3)

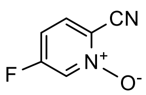

Trifluoroacetic anhydride (10.5 mL, 72.5 mmol, 2.1 eq.) was added dropwise under argon to a pre-cooled (ice-bath) solution of 5-fluoropicolinonitrile (**S2**, 4.2 g, 34.5 mmol) and urea hydrogen peroxide (UHP, 6.5 g, 69 mmol, 2 eq.) in CH_2Cl_2 (200 mL). After complete addition, the reaction mixture was allowed to warm to room temperature and vigorously stirred for 20 h, until TLC (Hex: AcOEt 3:2) indicated complete conversion of the starting material. The mixture was cooled to 0°C and stirred for 15 min with 100 mL aqueous Na_2SO_3 solution (8.7 g, 60 mmol) to quench remaining UHP. The layers where then separated and the aqueous layer was extracted with CH_2Cl_2. The combined organic fractions were dried over Na_2SO_4 and concentrated under reduced pressure to afford **S3** (4.02 g, 29.09 mmol, 85%) as a white solid that could be used without further purification.

R_f: 0.66 (**S2**) / 0.18 (**S3**)

^{1}H-NMR (400 MHz, $CDCl_3$): δ 8.33 – 8.11 (m, 1H), 7.71 (dd, *J* = 9.0, 6.5 Hz, 1H), 7.21 – 7.13 (m, 1H). ^{13}C-NMR (101 MHz, $CDCl_3$): δ 162.18 (d, *J* = 262.5 Hz), 131.45 (d, *J* = 10.5 Hz), 130.84 (d, *J* = 38.4 Hz), 113.46 (d, *J* = 21.4 Hz), 111.06 (s). ^{19}F-NMR (376 MHz, $CDCl_3$): δ -111.40 (s).

HRMS (ESI) calcd for $C_6H_3FN_2O$ $[M+H]^+$: 138.10, found: 138.0225; M.p. = 145 °C

Preparation of 6-cyano-3-fluoropyridin-2-yl acetate (S4)

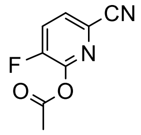

A solution of **S3** (4.02 g, 29.09 mmol) in acetic anhydride (40 mL) was refluxed for 16 h until TLC (Hex: AcOEt 3:2) indicated no further progression of the reaction, after which the mixture was allowed to cool to room temperature and the remaining acetic anhydride was evaporated. The crude product was then dissolved in EtOAc and purified by column chromatography (Hex:AcOEt 90%:10%) to afford **S4** (2.07g, 11.5 mmol, 40%) as a white solid.

R_f : 0.80

^{1}H-NMR (400 MHz, $CDCl_3$): δ 7.74 – 7.62 (m, 1H), 2.39 (d, J = 3.1 Hz, 2H). ^{13}C-NMR (101 MHz, $CDCl_3$): δ 167.18 (s), 152.59 (d, *J* = 289.1 Hz), 147.51 (d, *J* = 15.8 Hz), 129.35 (d, *J* = 4.9 Hz), 126.88 (d, *J* = 5.9 Hz), 126.66 (d, *J* = 18.4 Hz), 115.59 (s), 20.42 (s). ^{19}F-NMR (376 MHz, $CDCl_3$): δ -121.37 (s).

HRMS (ESI) calcd for $C_8H_5FN_2O_2$ $[M+H]^+$: 180.14, found: 180.0329; M.p. = 90 °C

6-Cyanopyridine-2,3-diyl diacetate (**S4b**) was isolated and characterized as a side-product

R_f : 0.54

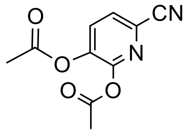

Yield : 13% (653.49 mg, 2.9 mmol)

^{1}H-NMR (400 MHz, $CDCl_3$): δ 7.85 (d, J = 8.2 Hz, 1H), 7.72 (d, J = 8.2 Hz, 1H), 2.39 (s, 3H), 2.36 (s, 3H).

MS (ESI) calcd for $C_{10}H_8N_2O_4$ $[M+H]^+$: 220.18, found: 221.18; M.p. = not measured

Preparation of 5-fluoro-6-oxo-1,6-dihydropyridine-2-carbonitrile (S5)

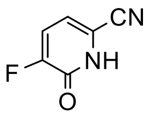

A solution of **S4** (2.07 g, 11.05 mmol) and K_2CO_3 (3.18 g, 23 mmol, 2 eq.) in MeOH (23 mL) was vigorously stirred at room temperature under argon for 1 h, until TLC (Hex: AcOEt 7:3) indicated complete consumption of the starting material. After removal of the solvent, the crude mixture was dissolved in H_2O (25 mL), acidified with concentrated HCl (4 mL) and extracted with EtOAc (150 mL). The organic phase was dried over Na_2SO_4 and concentrated under reduced pressure to afford **S5** (1.49 g, 10.8 mmol, 94%) as a white solid.

R_f : 0.0

^{1}H-NMR (400 MHz, $CDCl_3$): δ 12.28 (s, 6H), 7.28 (t, *J* = 2.9 Hz, 1H), 7.16 (ddd, *J* = 10.8, 5.7, 2.8 Hz, 9H), 6.87 (ddd, *J* = 7.6, 6.1, 2.9 Hz, 9H). ^{13}C-NMR (101 MHz, $CDCl_3$): δ 155.33 (d, *J* = 18.9 Hz), 152.36 (d, *J* = 265.3 Hz), 122.07 (d, *J* = 18.1 Hz), 118.58 (d, *J* = 4.5 Hz), 114.97 (s). ^{19}F-NMR (376 MHz, $CDCl_3$): δ -125.55 (s).

HRMS (ESI) calcd for $C_6H_3FN_2O$ $[M+H]^+$: 138.02, found: 138.0223; M.p. = 209 °C

Preparation of 5-fluoro-1-methyl-6-oxo-1,6-dihydropyridine-2-carbonitrile (31)

A solution of **S5** (1.49g, 10.8 mmol) and K_2CO_3 (2.99 g, 21.6 mmol, 2 eq.) in DMF (30 mL) was vigorously stirred at room temperature under argon for 15 minutes. The mixture was cooled to 0 °C with an ice bath and MeI (740 µL, 11.9 mmol, 1.1 eq.) was added dropwise, after which the reaction mixture was allowed to warm to room temperature. TLC analysis (Hex: AcOEt 1:1) after 2 hours indicated complete consumption of the starting material but presence of two products. 2 M NaOH (2 mL) was then added to quench excess MeI and the reaction mixture was extracted with EtOAc until the aqueous phase was free of products. The combined organic fractions were dried over Na_2SO_4 and concentrated under reduced pressure, after which the crude products were purified by column chromatography (Hex:AcOEt 80%:20%) to afford **31** (670 mg, 4.4 mmol, 40%) as a white solid.

5-Fluoro-1-methyl-6-oxo-1,6-dihydropyridine-2-carbonitrile (**31**)

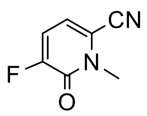

R$_f$: 0.56

^{1}H-NMR (400 MHz, $CDCl_3$): δ 7.12 (t, *J* = 7.9 Hz, 1H), 6.77 (dd, *J* = 7.7, 4.5 Hz, 1H), 3.78 (s, 3H). ^{13}C-NMR (101 MHz, $CDCl_3$): δ 155.82 (d, *J* = 263.1 Hz), 155.35 (d, *J* = 26.3 Hz), 118.32 (d, *J* = 18.9 Hz), 117.19 (d, *J* = 6.1 Hz), 114.29 (d, *J* = 6.6 Hz), 112.29 (s, *J* = 2.0 Hz), 34.96 (s, *J* = 1.5 Hz). ^{19}F-NMR (376 MHz, $CDCl_3$): δ -116.91 (s).

HRMS (ESI) calcd for $C_7H_5FN_2O$ $[M+H]^+$: 152.13, found: 152.0381; M.p. = 107 °C

The *O*-protected analog 5-fluoro-6-methoxypicolinonitrile **(31b)** was identified as a side-product

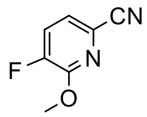

R$_f$: 0.85

Yield : not determined

^{1}H-NMR (400 MHz, $CDCl_3$): δ 7.43 (dd, J = 9.1, 8.0 Hz, 1H), 7.34 (dd, J = 8.0, 3.1 Hz, 1H), 4.06 (s, 3H). ^{13}C-NMR (101 MHz, $CDCl_3$): δ 154.21 (d, J = 12.4 Hz), 149.93 (d, J = 269.5 Hz), 124.85 (d, J = 6.5 Hz), 123.44 (d, J = 5.2 Hz), 123.34 (d, J = 8.1 Hz), 116.64 (s), 54.65 (s). ^{19}F-NMR (376 MHz, $CDCl_3$): δ -129.40 (s).

MS (ESI) calcd for $C_7H_5FN_2O$ $[M+H]^+$: 152.13, found: 153.02 ; M.p. = not determined

2.2.3 Preparation of *O*-protected precursor **39** and reference compound **5**.

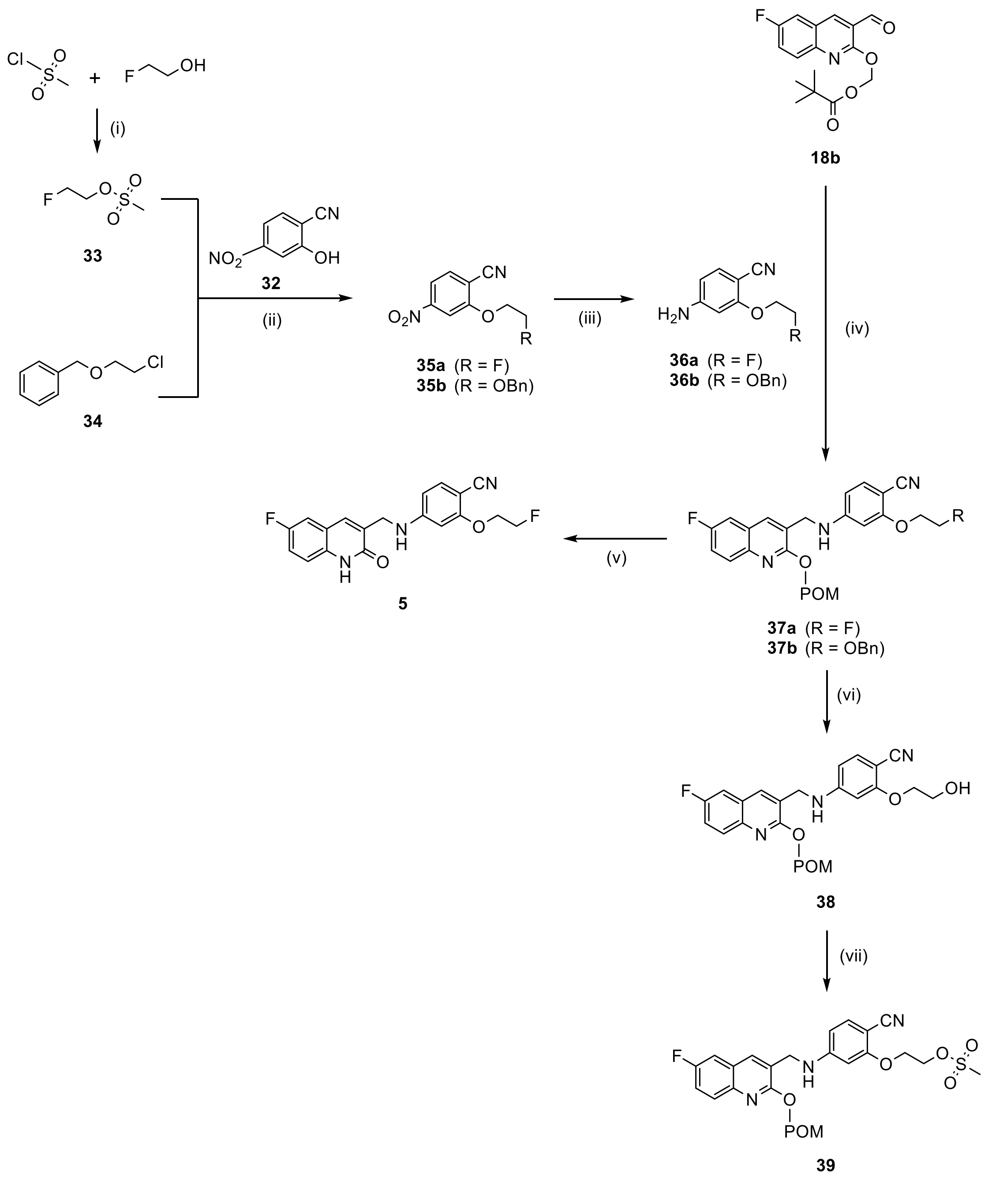

Scheme S3. *Preparation of radiolabeling precursor* ***39*** *and reference compound* ***5****.*
Procedure: i) Ms-Cl, Et_3N, CH_2Cl_2, rt, 3 h; ii) ***32*** *Cs_2CO_3, DMF, 60 °C, 2.5 h; iii) zinc powder, NH_4Cl, EtOH, rt; iv)* ***36a,b****, TMS-OTf, $NaBH_4$, DMF, 0 °C, 5 h; v) 40% $NaOH_{aq}$, MeOH, 50 °C, 1 h; vi) Pd/charcoal, EtOH, rt, 5 h; vii) Ms-Cl, Et_3N, CH_2Cl_2, 0 °C to rt, 2.5 h.*

Preparation of 2-fluoroethyl methanesulfonate (33)

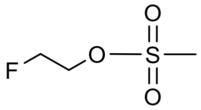

Et$_3$N (3.30 mL, 23.41 mmol, 1.5 eq.) was added under argon to a solution of fluoroethanol (1 g, 15.61 mmol, 1 eq.) in CH_2Cl_2 (15 mL, [1.0]). The mixture was cooled to 0 °C and methanesulfonyl chloride (1.32 mL, 17.61 mmol, 1.1 eq.) was added, upon which the solution turned turbid and yellow. After stirring at room temperature for 3 h, the mixture was diluted with CH_2Cl_2 and successively washed with ice water, 10% HCl, saturated $NaHCO_3$ and brine. The organic phase was dried over Na_2SO_4 and concentrated under reduced pressure to afford **33** (2.10g, 14.77 mmol, 95%) as an orange oil.

R_f : 0.54 (Hex:AcOEt 3:2)

^{1}H-NMR [400 MHz, $(CD_3)_2SO$]: δ 4.78 – 4.69 (m, 1H), 4.65 – 4.59 (m, 1H), 4.52 – 4.47 (m, 1H), 4.44 – 4.39 (m, 1H), 3.22 (s, 3H). ^{19}F-NMR [376 MHz, $(CD_3)_2SO$]: δ -223.51 (s).

MS (ESI) calcd for $C_3H_7FO_3S$ $[M+H]^+$: 142.14, found: not detectable; M.p. = oil

Preparation of 2-(2-fluoroethoxy)-4-nitrobenzonitrile (35a)

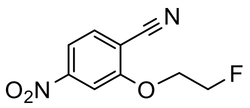

33 (250.00 mg, 1.52 mmol, 1.3 eq.) was added under argon to a solution of 2-hydroxy-4-nitrobenzonitrile (**32**, 192.0 mg, 1.17 mmol, 1 eq.) and Cs_2CO_3 (495.2 mg, 1.52 mmol, 1.3 eq.) in DMF (5.8 mL, [0.2]). The reaction mixture was vigorously stirred at 60 °C for 2.5 h until TLC (Hex: AcOEt 3:2) indicated complete conversion of the starting material. The solvent was evaporated and the residue was dissolved in a mixture of EtOAc and H_2O. The organic phase was separated, dried over Na_2SO_4 and concentrated under reduced pressure. The crude product thus obtained was purified by column chromatography to afford **35a** (319 mg, 1.51 mmol, 100%) as a dark orange oil.

R_f : 0.26

^{1}H-NMR [400 MHz, $(CD_3)_2SO$]: δ 8.11 (d, J = 8.5 Hz, 1H), 8.04 (d, J = 2.0 Hz, 1H), 7.95 (dd, J = 8.5, 2.1 Hz, 1H), 4.91 – 4.86 (m, 1H), 4.79 – 4.74 (m, 1H), 4.69 – 4.64 (m, 1H), 4.62 – 4.57 (m, 1H). ^{13}C-NMR [101 MHz, $(CD_3)_2SO$]: δ 160.85 (s), 151.79 (s), 135.79 (s), 116.59 (s), 115.24 (s), 108.76 (s), 107.12 (s), 82.14 (d, J = 167.4 Hz), 69.81 (d, J = 18.8 Hz). ^{19}F-NMR [376 MHz, $(CD_3)_2SO$]: δ -222.61 (s, J = 19.7 Hz).

MS (ESI) calcd for $C_9H_7FN_2O_3$ $[M+H]^+$: 210.16, found: not detectable; M.p. = oil

Preparation of 2-(2-(benzyloxy)ethoxy)-4-nitrobenzonitrile (35b)

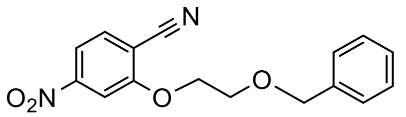

35b (2.95 g, 10.11 mmol, 85%, yellow solid) was prepared from **34** (2.55 g, 11.88 mmol, 1.3 eq.) using the same procedure as described for **35a**.

R_f : 0.23

^{1}H-NMR [400 MHz, $(CD_3)_2SO$]: δ 8.09 (d, *J* = 8.5 Hz, 1H), 8.04 (d, *J* = 2.0 Hz, 1H), 7.92 (dd, *J* = 8.5, 2.1 Hz, 1H), 7.39 – 7.22 (m, 5H), 4.61 (s, 2H), 4.55 – 4.48 (m, 2H), 3.88 – 3.79 (m, 2H). ^{13}C-NMR [101 MHz, $(CD_3)_2SO$]: δ 161.34 (s), 151.75 (s), 138.69 (s), 135.66 (s), 128.72 (s), 127.88 (s), 127.78 (s), 116.25 (s), 115.41 (s), 108.78 (s), 107.08 (s), 72.48 (s), 70.12 (s), 68.15 (s).

MS (ESI) calcd for $C_{16}H_{14}N_2O_4$ $[M+H]^+$: 298.30, found: not detectable; M.p. > 300

Preparation of 4-amino-2-(2-fluoroethoxy)benzonitrile (36a)

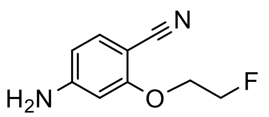

A mixture of **35a** (972 mg, 4.62 mmol, 1 eq.), NH_4Cl (2.4 g, 46.25 mmol, 10 eq.) and zinc powder (3.0 g, 46.25 mmol, 10 eq.) in EtOH (23 mL, [0.2]) was vigorously stirred at room temperature for 40 h until mass spectrometry indicated complete conversion of the starting material. The remaining zinc powder was removed by filtration over a celite pad and rinsed with EtOH. The filtrate was dried over Na_2SO_4 and concentrated under reduced pressure. The crude product thus obtained was purified by column chromatography to afford **36a** (616 mg, 3.42 mmol, 74%) as a white solid.

R_f : 0.38

^{1}H-NMR [400 MHz, $(CD_3)_2SO$]: δ 7.26 (d, *J* = 8.4 Hz, 1H), 6.27 – 6.20 (m, 2H), 6.19 (s, 2H), 4.85 – 4.77 (m, 1H), 4.74 – 4.66 (m, 1H), 4.27 (dd, *J* = 4.6, 3.2 Hz, 1H), 4.20 (dd, *J* = 4.6, 3.2 Hz, 1H). ^{13}C-NMR [101 MHz, $(CD_3)_2SO$]: δ 161.75 (s), 155.30 (s), 134.88 (s), 118.66 (s), 107.27 (s), 96.95 (s), 86.22 (s), 82.32 (d, *J* = 167.1 Hz), 67.88 (d, *J* = 19.1 Hz). ^{19}F-NMR [376 MHz, $(CD_3)_2SO$]: δ -222.25 (s).

MS (ESI) calcd for $C_9H_9FN_2O$ $[M+H]^+$: 180.18, found: 181.35; M.p. =133.6°C

Preparation of 4-amino-2-(2-(benzyloxy)ethoxy)benzonitrile (36b)

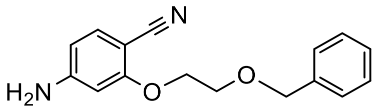

36b (1.63 g, 6.07 mmol, 60%) was prepared from **35b** (2.95 g, 10.11 mmol, 1 eq.) using the same procedure as described for **36a** (reaction time: 30 h).

R_f : 0.30

^{1}H-NMR [400 MHz, $(CD_3)_2SO$]: δ 7.40 – 7.27 (m, 5H), 7.25 (d, *J* = 8.4 Hz, 1H), 6.25 (d, *J* = 1.9 Hz, 1H), 6.19 (dd, *J* = 8.4, 1.9 Hz, 1H), 6.16 (s, 2H), 4.61 (s, 2H), 4.14 (dd, *J* = 5.3, 3.8 Hz, 2H), 3.78 (dd, *J* = 5.3, 3.9 Hz, 2H). ^{13}C-NMR [101 MHz, $(CD_3)_2SO$]: δ 161.96 (s), 157.69 (s),

138.83 (s), 134.47 (s), 128.74 (s), 127.83 (s), 118.29 (s), 105.01 (s), 95.17 (s), 89.34 (s), 72.52 (s), 68.51 (s), 68.18 (s).

HRMS (ESI) calcd for $C_{16}H_{16}N_2O_2$ $[M+H]^+$: 268.32, found: 269.1286; M.p. = oil

Preparation of ((3-(((4-cyano-3-(2-fluoroethoxy)phenyl)amino)methyl)-6-fluoroquinolin-2-yl)oxy)methyl pivalate (37a)

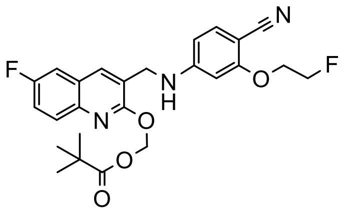

Trimethylsilyl chloride (TMS-OTf, 303 µL, 1.67 mmol, 2.5 eq.) and $NaBH_4$ (25.3 mg, 0.67 mmol, 1 eq.) were added under argon to a pre-cooled (0 °C) solution of **18b** (206.10 mg, 0.67 mmol, 1 eq.) and **36a** (126.00 mg, 0.67 mmol, 1 eq.) in anhydrous DMF (450 µL, [0.5]). The reaction mixture was stirred at 0 °C for 1 h until TLC (Hex:AcOEt 3:2) indicated complete conversion of the starting materials. The resulting mixture was diluted with AcOEt and remaining TMS-OTf quenched by addition of Na_2CO_3 (s.s.), after which the organic phase was dried over Na_2SO_4 and concentrated under reduced pressure. The crude product thus obtained was purified by column chromatography to afford **37a** (138.00 mg, 0.29 mmol, 44%) as a white solid.

R_f : 0.45

^{1}H-NMR [400 MHz, $(CD_3)_2SO$]: δ 8.12 (s, 1H), 7.82 (dd, *J* = 9.2, 5.3 Hz, 1H), 7.77 (dd, *J* = 9.4, 2.9 Hz, 1H), 7.58 (td, *J* = 8.8, 2.9 Hz, 1H), 7.33 (d, *J* = 8.6 Hz, 1H), 7.26 (t, *J* = 6.0 Hz, 1H), 6.34 (d, *J* = 1.7 Hz, 1H), 6.26 (dd, *J* = 8.7, 1.8 Hz, 1H), 6.24 (s, 2H), 4.79 (dd, *J* = 4.6, 3.1 Hz, 1H), 4.67 (dd, *J* = 4.6, 3.0 Hz, 1H), 4.43 (d, *J* = 5.5 Hz, 2H), 4.32 – 4.28 (m, 1H), 4.25 – 4.21 (m, 1H), 1.13 (s, 9H). ^{13}C-NMR [101 MHz, $(CD_3)_2SO$]: δ 177.03 (s), 161.14 (d, *J* = 124.8 Hz), 158.11 (s), 157.84 (d, *J* = 2.1 Hz), 154.22 (s), 141.89 (s), 136.42 (d, *J* = 4.5 Hz), 134.88 (s), 129.47 (d, *J* = 9.0 Hz), 126.47 (d, *J* = 10.3 Hz), 124.06 (s), 119.60 (d, *J* = 25.5 Hz), 118.40 (s), 111.68 (d, *J* = 22.2 Hz), 105.92 (s), 95.87 (s), 87.20 (s), 82.85 (d, *J* = 50.6 Hz), 81.44 (s), 68.09 (d, *J* = 19.1 Hz), 41.40 (s), 26.99 (s). ^{19}F-NMR [376 MHz, $(CD_3)_2SO$]: δ -116.13 (s), -222.26 (s).

HRMS (ESI) calcd for $C_{25}H_{25}F_2N_3O_4$ $[M+H]^+$: 469.49, found: 470.1889; M.p. = 161.2 °C

Preparation of 2-(2-cyano-5-(((6-fluoro-2-((pivaloyloxy)methoxy)quinolin-3-yl)methyl)amino)phenoxy)ethyl benzoate (37b) and 2-(2-cyano-5-(((6-fluoro-2-oxo-1-((pivaloyloxy)methyl)-1,2-dihydroquinolin-3-yl)methyl)amino)phenoxy)ethyl benzoate (S6)

37b (885.20 mg, 1.58 mmol, 44%, clear oil) and **S6** (553.10 mg, 0.95 mmol, 27%, white solid) were prepared from a mixture of **17b** and **18b** (1.09 g, 3.57 mmol, 1 eq.) and **36b** (957.04 mg, 3.57 mmol, 1 eq.) using the same procedure as described for **37a**.

2-(2-Cyano-5-(((6-fluoro-2-((pivaloyloxy)methoxy)quinolin-3-yl)methyl)amino)phenoxy)ethyl benzoate (**37b**)

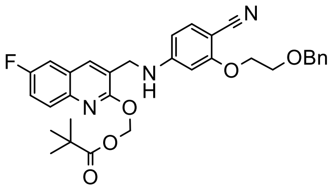

R_f: 0.6

^{1}H-NMR [400 MHz, $(CD_3)_2SO$]: δ 8.11 (s, 1H), 7.82 (dd, *J* = 9.2, 5.3 Hz, 1H), 7.75 (dd, *J* = 9.4, 2.9 Hz, 1H), 7.58 (td, *J* = 8.8, 2.9 Hz, 1H), 7.36 – 7.21 (m, 7H), 6.33 (d, *J* = 1.5 Hz, 1H), 6.27 – 6.23 (m, 3H), 4.55 (s, 2H), 4.42 (d, *J* = 5.7 Hz, 2H), 4.16 (dd, *J* = 5.3, 3.7 Hz, 2H), 3.76 – 3.71 (m, 2H), 1.12 (s, 9H). ^{13}C-NMR [101 MHz, $(CD_3)_2SO$]: δ 177.02 (s), 162.17 (s), 160.52 (s), 157.98 (d, *J* = 26.9 Hz), 154.17 (s), 141.88 (s), 138.76 (s), 136.37 (d, *J* = 4.6 Hz), 134.79 (s), 129.47 (d, *J* = 9.1 Hz), 128.71 (s), 127.83 (d, *J* = 2.2 Hz), 126.47 (d, *J* = 10.2 Hz), 124.08 (s), 119.59 (d, *J* = 25.3 Hz), 118.58 (s), 111.67 (d, *J* = 22.1 Hz), 105.77 (s), 95.78 (s), 87.24 (s), 82.59 (s), 72.50 (s), 68.37 (s), 68.15 (s), 41.38 (s), 38.72 (s), 26.99 (s). ^{19}F-NMR [376 MHz, $(CD_3)_2SO$]: δ -116.12 (s).

HRMS (ESI) calcd for $C_{32}H_{32}FN_3O_5$ $[M+H]^+$: 557.62, found: 558.2401; M.p. = oil

2-(2-Cyano-5-(((6-fluoro-2-oxo-1-((pivaloyloxy)methyl)-1,2-dihydroquinolin-3-yl)methyl)amino)phenoxy)ethyl benzoate (**S6**)

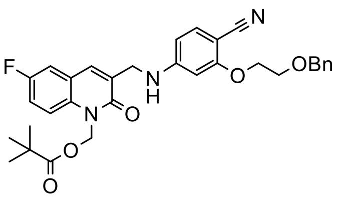

R_f: 0.3

^{1}H-NMR [400 MHz, $(CD_3)_2SO$]: δ 7.77 (s, 1H), 7.66 (dd, *J* = 8.6, 2.1 Hz, 1H), 7.54 – 7.47 (m, 2H), 7.36 – 7.24 (m, 7H), 7.19 (t, *J* = 6.1 Hz, 1H), 6.37 – 6.25 (m, 4H), 4.56 (s, 2H), 4.29 (d, *J* = 5.6 Hz, 2H), 4.18 (dd, *J* = 5.3, 3.7 Hz, 2H), 3.75 (dd, *J* = 5.2, 3.8 Hz, 2H), 1.12 (s, 9H). ^{13}C-NMR [101 MHz, $(CD_3)_2SO$]: δ 177.21 (s), 161.53 (d, *J* = 135.6 Hz), 159.29 (s), 156.90 (s), 154.28 (s), 138.78 (s), 135.47 (d, *J* = 2.4 Hz), 134.75 (s), 131.08 (s), 128.71 (s), 127.82 (d, *J* = 1.9 Hz), 121.31 (d, *J* = 9.1 Hz), 118.64 (s), 118.49 (d, *J* = 23.8 Hz), 116.99 (d, *J* = 8.3 Hz), 114.27 (d, *J* = 23.0 Hz), 105.89 (s), 95.73 (s), 87.09 (s), 72.51 (s), 68.40 (s), 68.19 (s), 67.02 (s), 42.24 (s), 38.89 (s), 27.12 (s). ^{19}F-NMR [376 MHz, $(CD_3)_2SO$]: δ -120.26 (s).

MS (ESI) calcd for $C_{32}H_{32}FN_3O_5$ $[M+H]^+$: 557.62, found: 558.15; M.p. = 196.2 °C

Preparation of 4-(((6-fluoro-2-oxo-1,2-dihydroquinolin-3-yl)methyl)amino)-2-(2-fluoroethoxy)benzonitrile (5)

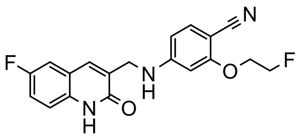

A suspension of **37a** (138.0 mg, 0.29 mmol) in 40% $NaOH_{aq.}$ (2 mL) and MeOH (2 mL) was stirred at 50 °C for 1 h until formation of a clear solution and TLC (Hex:AcOEt 3:2) indicated complete conversion of the starting material. After removal of MeOH and cooling of the aqueous phase at 4 °C in the fridge overnight, the resulting precipitate was recovered by filtration to afford **5** (10.65 mg, 0.03 mmol, 10%) as a white solid.

R_f : 0.0

^{1}H-NMR [400 MHz, $(CD_3)_2SO$]: δ 12.02 (d, *J* = 16.0 Hz, 1H), 7.72 (s, 1H), 7.55 (dd, *J* = 9.3, 2.5 Hz, 1H), 7.38 (dd, *J* = 8.9, 2.7 Hz, 1H), 7.33 (d, *J* = 8.6 Hz, 2H), 7.18 (t, *J* = 6.0 Hz, 1H), 6.35 (d, *J* = 1.6 Hz, 1H), 6.28 (dd, *J* = 8.6, 1.6 Hz, 1H), 4.82 – 4.77 (m, 1H), 4.70 – 4.64 (m, 1H), 4.35 – 4.30 (m, 1H), 4.25 (t, *J* = 5.9 Hz, 3H), 3.18 (d, *J* = 5.2 Hz, 2H). ^{13}C-NMR [101 MHz, $(CD_3)_2SO$]: δ 161.79 (s), 161.73 (s), 157.49 (d, *J* = 237.7 Hz), 154.38 (s), 135.14 (s), 134.91 (d, *J* = 3.0 Hz), 134.81 (s), 131.92 (s), 120.19 (d, *J* = 9.0 Hz), 118.51 (s), 118.28 (d, *J* = 24.4 Hz), 117.13 (d, *J* = 8.5 Hz), 112.92 (d, *J* = 22.9 Hz), 106.05 (s), 95.70 (s), 86.85 (s), 82.30 (d, *J* = 167.1 Hz), 68.08 (d, *J* = 19.0 Hz), 41.85 (s). ^{19}F-NMR [376 MHz, $(CD_3)_2SO$]: δ -121.03 (s), -222.24 (s).

HRMS (ESI) calcd for $C_{19}H_{15}F_2N_3O_2$ $[M+H]^+$: 355.34, found: 356.1206; M.p. >300 °C

Preparation of ((3-(((4-cyano-3-(2-hydroxyethoxy)phenyl)amino)methyl)-6-fluoroquinolin-2-yl)oxy)methyl pivalate (38)

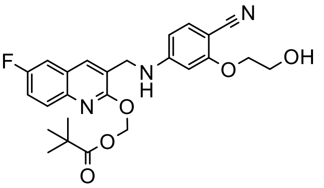

Palladium/charcoal (15% mass, 110 mg) was added to a solution of **37b** (885.2 mg, 1.58 mmol) in EtOH (15 mL, [0.1]) and the mixture was flushed with hydrogen gas for ten minutes. The flask was closed with a rubber septum and a balloon filled with hydrogen was placed on top. The mixture was stirred at room temperature for 5 h until TLC (Hex:AcOEt 3:2) indicated complete conversion of the starting material. The charcoal was removed by filtration over a celite pad and rinsed with EtOH. The organic phase was dried over Na_2SO_4 and concentrated under reduced pressure to afford **38** (718 mg, 1.53 mmol, 97%) as a dard solid.

R_f : 0.15

^{1}H-NMR [400 MHz, $(CD_3)_2SO$]: δ 8.12 (s, 1H), 7.82 (dd, *J* = 9.2, 5.3 Hz, 1H), 7.76 (dd, *J* = 9.4, 2.9 Hz, 1H), 7.58 (td, *J* = 8.8, 2.9 Hz, 1H), 7.30 (d, *J* = 8.5 Hz, 1H), 7.22 (t, *J* = 6.0 Hz, 1H), 6.33 (d, *J* = 1.6 Hz, 1H), 6.27 – 6.20 (m, 3H), 4.88 (t, *J* = 5.3 Hz, 1H), 4.42 (d, *J* = 5.7 Hz, 2H), 4.01 (t, *J* = 5.0 Hz, 2H), 3.70 (dd, *J* = 10.2, 5.2 Hz, 2H), 1.13 (s, 9H). ^{13}C-NMR [101 MHz, $(CD_3)_2SO$]: δ 177.02 (s), 162.26 (s), 160.53 (s), 157.98 (d, *J* = 27.1 Hz), 154.17 (s), 141.88 (s),

136.39 (d, *J* = 4.6 Hz), 134.88 (s), 129.47 (d, *J* = 9.1 Hz), 126.47 (d, *J* = 10.2 Hz), 124.11 (s), 119.59 (d, *J* = 25.4 Hz), 118.62 (s), 111.68 (d, *J* = 22.1 Hz), 105.43 (s), 95.92 (s), 87.28 (s), 82.59 (s), 70.46 (s), 59.70 (s), 41.39 (s), 38.73 (s), 27.00 (s). ^{19}F-NMR [376 MHz, $(CD_3)_2SO$]: δ -116.12 (s).

HRMS (ESI) calcd for $C_{25}H_{26}FN_3O_5$ $[M+H]^+$: 467.50, found: 468.1932; M.p. >300

Preparation of ((3-(((4-cyano-3-(2-((methylsulfonyl)oxy)ethoxy)phenyl)amino)methyl)-6-fluoroquinolin-2-yl)oxy)methyl pivalate (39)

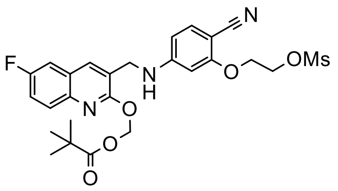

Et_3N (320 µL, 2.29 mmol, 1.5 eq.) and Ms-Cl (142 µL, 1.83 mmol, 1.2 eq.) were added under argon to a pre-cooled (0 °C) solution of **38** (718 mg, 1.53 mmol) in anhydrous CH_2Cl_2 (7.5 mL). After stirring for 30 min at 0 °C, TLC still indicated the presence of unreacted starting materials, so that the mixture was stirred at room temperature for another 2 h. As TLC after 2 h indicated no further reaction, the mixture was diluted with H_2O and the organic layer was separated and washed with additional H_2O. The aqueous layer and washes were further extracted with ethyl acetate, which was combined with the organic layer, dried over Na_2SO_4 and concentrated under reduced pressure. The crude product thus obtained was purified by column chromatography (Hex:AcOEt 75%:25%) to afford **39** (650 mg, 1.19 mmol, 78%) as a white solid.

R_f : 0.23 (Hex:AcOEt 3:2)

^{1}H-NMR [400 MHz, $(CD_3)_2SO$]: δ 8.12 (s, 1H), 7.82 (dd, *J* = 9.1, 5.3 Hz, 1H), 7.76 (dd, *J* = 9.4, 2.9 Hz, 1H), 7.58 (td, *J* = 8.9, 2.9 Hz, 1H), 7.34 (d, *J* = 8.6 Hz, 1H), 7.27 (t, *J* = 5.9 Hz, 1H), 6.36 (d, *J* = 1.2 Hz, 1H), 6.27 (dd, *J* = 8.7, 1.3 Hz, 1H), 6.24 (s, 2H), 4.54 (dd, *J* = 5.2, 3.1 Hz, 2H), 4.44 (d, *J* = 5.6 Hz, 2H), 4.28 (dd, *J* = 5.1, 3.2 Hz, 2H), 3.24 (s, 3H), 1.13 (s, 9H). ^{13}C-NMR [101 MHz, $(CD_3)_2SO$]: δ 177.03 (s), 161.56 (s), 160.53 (s), 157.99 (d, *J* = 26.2 Hz), 154.27 (s), 141.89 (s), 136.41 (d, *J* = 4.7 Hz), 134.88 (s), 129.47 (d, *J* = 9.1 Hz), 126.48 (d, *J* = 10.4 Hz), 124.05 (s), 119.61 (d, *J* = 25.2 Hz), 118.44 (s), 111.69 (d, *J* = 22.1 Hz), 106.01 (s), 95.92 (s), 87.10 (s), 82.60 (s), 68.90 (s), 66.74 (s), 41.40 (s), 38.74 (s), 37.25 (s), 27.00 (s). ^{19}F-NMR [376 MHz, $(CD_3)_2SO$]: δ -116.12 (s).

HRMS (ESI) calcd for $C_{26}H_{28}FN_3O_7S$ $[M+H]^+$: 545.58, found: 546.1704; M.p. = 129.7 °C

3. Radiochemistry

3.1 General Conditions

[^{18}F]Fluoride ([^{18}F]F^{-}) was produced via the $^{18}O(p,n)^{18}F$ nuclear reaction by bombardment of enriched [^{18}O]H_2O with 17 MeV protons at the BC1710 cyclotron (The Japan Steel Works, Tokyo, Japan) of the INM-5 (Forschungszentrum Jülich). Radioactivity was measured using a Curiementor 2 dose calibrator by PTW Freiburg (Freiburg, Germany). Sep-Pak Accell Plus QMA carbonate cartridges (130 mg sorbent, part no. 186004051; preconditioned with 1 mL of H_2O) were used for [^{18}F]fluorine fixation and Oasis HLB (30 mg sorbent, part no. WAT094225, by Waters (Munich, Germany) for solid phase extraction.

All reactions were performed under air in Wheaton v-vials equipped with PTFE magnetic stirring bars and sealed with silicone septa. The reactors were placed in an aluminum block on the magnetic stirrer, which was equipped with boreholes matching the reaction vessels.

A glass stirring bar was used for radiofluorinations when the product was used for biological evaluations in order to ensure higher molar activities.

3.2. Analytical high-performance liquid chromatography (HPLC)

High-performance liquid chromatography (HPLC) analyses were performed on a Dionex Ultimate® 3000 System (Thermo Fisher Scientific Inc., Bremen, Germany) with Ultimate® 3000 RS variable wavelenght detector (Thermo Fisher Scientific Inc., Bremen, Germany) coupled in series with a HERM LB500 radiodetector (Berthold Technologies, Bad Wildbad, Germany). Two Rheodyne 6-port injections valves equipped with equal sample loops (20 µL) were installed before (for regular sample injection) and behind (for post-column injection) the chromatographic column. For internal reference, the total activity in all samples was determined by post-column injection of a second aliquot to avoid errors due to incomplete recovery of activity from the column. No post-column injection was performed for quality controls. The UV and radioactivity detectors were connected in series, giving a time delay of 0.25 min between the corresponding responses at a flow rate of 1 mL/min.

For the analysis of all tracers reported in this work a Synergi Hydro-RP 4 µm (80 Å) 250 × 4.6 mm (Phenomenex, Aschaffenburg, Germany) was used (Table S1). For the analysis of tracers containing a stereocenter, a CHIRALPACK AD column was used (Table S2). The HPLC conditions and retention times for the different radiotracers are summarized in Tables S1 and S2.

Table S1: Analytical HPLC conditions for the synthesized radiotracers using a Synergi Hydro-RP 4 µm (80 Å) 250 × 4.6 mm column.

Tracer	Analytical HPLC	
	Eluent	t_R (min)
[^{18}F]mIDH-138 ([^{18}F]**4**)	35% ACN + gradient 35-80%	17.7
(*S*)-[^{18}F]mIDH-23 [(*S*)-[^{18}F]**2**]	32% ACN + gradient 32-80%	12.3
(*R*)-[^{18}F] mIDH-23 [(*R*)-[^{18}F]**2**]	32% ACN + gradient 32-80%	12.3
[^{18}F]FE-mIDH-138 ([^{18}F]**5**)	40% ACN + 0.1%TFA + gradient 40-90%	12.4

Table S2: Analytical HPLC conditions for (*S*)- and (*R*)-[^{18}F]**2** using a CHIRALPACK AD, 10 µm (80 Å) 250 × 4.6 mm column.

Tracer	Chiral Analytical HPLC	
	Eluent	t_R (min)
(*S*)-[^{18}F]mIDH-23 [(*S*)-[^{18}F]**2**]	20% iPrOH in hexane	21.8
(*R*)-[^{18}F] mIDH-23 [(*R*)-[^{18}F]**2**]	20% iPrOH in hexane	19.4

3.3. Semipreparative HPLC

Semipreparative HPLC was performed on a dedicated semipreparative HPLC system consisting of a Knauer K-100 pump (Knauer Wissenschaftliche Geräte GmbH, Berlin, Germany), a Knauer K-2501 UV Detector (Knauer Wissenschaftliche Geräte GmbH, Berlin, Germany), a Rheodyne 6-port injection valve equipped with a 2 mL injection loop and a custom-made Geiger counter. The HPLC conditions and retention times for the different radiotracers are summarized in Table S3.

Table S3: Semi-preparative HPLC conditions for the synthesized radiotracers using a Synergi Hydro RP 10 µm (80 Å) 250 x 10 mm LC column.

Tracer	Semi-preparative HPLC	
	Eluent	t_R (min)
[^{18}F]mIDH-138 ([^{18}F]**4**)	30% ACN	42-47

(*S*)-[^{18}F]mIDH-23 [(*S*)-[^{18}F]**2**]	28% ACN	35-39
(*R*)-[^{18}F] mIDH-23 [(*R*)-[^{18}F]**2**]	28% ACN	35-39
[^{18}F]FE-mIDH-138 ([^{18}F]**5**)	35% ACN + 0.1%TFA	62-68

3.4. Radiosynthesis of [^{18}F]**4**

3.4.1. Copper-mediated radiofluorination of non-protected precursor **14**

Precursor **14** was radiolabeled according to a modification of the copper-mediated radiofluorination protocol published by Schäfer in 2016 (**Scheme S4**). To this end, [^{18}F]F^{-} in [^{18}O]H_2O was loaded (from the male side) on a QMA cartridge and the cartridge was rinsed (from the male side) with MeOH and dried with air. The [^{18}F]F^{-} was then eluted (from the female side) with a solution of Et_4NHCO_3 (1 mg, 5.22 µmol) in MeOH (1 mL) and the MeOH was removed at 85 °C under reduced pressure within 5 min, which resulted in an elution efficiency of around 99%. A solution of precursor **14** (12.9 mg, 30 µmol) and Cu(4-PhePy)$_4$(ClO$_4$)$_2$ (27.0 mg, 30 µmol) in DMA (0.8 mL) was then added and the reaction mixture was stirred at 110 °C for 10 minutes, followed by mild acidification with trifluoroacetic acid (TFA).

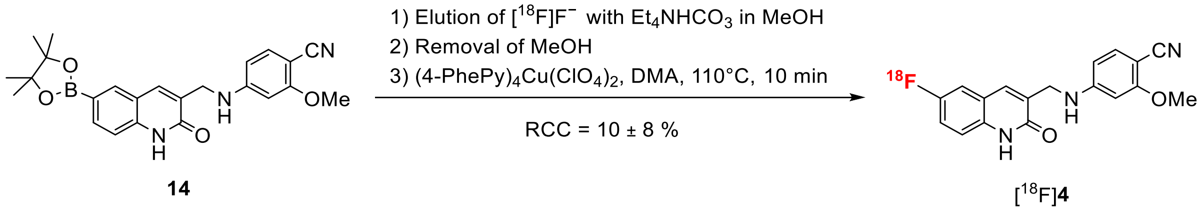

Scheme S4. Radiosynthesis of [^{18}F]**4** by copper-mediated radiofluorination of precursor **14**.

This protocol afforded the desired ^{18}F-labeled probe [^{18}F]**4**, but only in low radiochemical conversions (RCCs) of 10 ± 8 %, as determined by HPLC analysis with post-column injection. Since coordinating heteroatoms can generate unreactive copper species and thus interfere with copper-mediated radiofluorination, a series of spiking experiments was performed to assess whether the low ^{18}F-incorporation was related to any specific moiety present in the precursor.

3.4.2. Spiking experiment

Radiofluorination of 4-acetylbenzeneboronic acid (**S7**) was selected as a benchmark reaction for the spiking experiments and carried out under the same conditions (time, temperature and copper mediator) as described above, which yielded 4-[^{18}F]fluoroacetophenone ([^{18}F]**S8**) in RCCs of 72 ± 5% (**Scheme S5A**). In contrast, when the reaction was spiked with an equimolar

amount of compound **13a**, which contains the same functional groups as precursor **14** except for the BPin leaving group, formation of the product was completely prevented (**Scheme S5B**). As this observation suggested that at least one of the two NH groups present in **13a** and radiolabeling precursor **14** interferes with copper-mediated radiofluorination, additional spiking experiments were performed to distinguish between the effects of the two groups. Addition of the unprotected brominated quinolone **12a** also completely prevented copper-mediated radiofluorination of **S7** (**Scheme S5C**), indicating the need for an appropriate protecting group to shield the proton of the amide moiety. This was further confirmed by spiking experiments with an *N*-ethylated analog of **12a** (**S1a**), which afforded [^{18}F]**S8** in RCCs of 55 ± 17% (**Scheme S5D**). In addition, radiofluorination of **S7** in the presence of **17a**, which contains an easily cleavable POM-protecting group instead of the ethyl group, afforded [^{18}F]**S8** in RCCs of 60 ± 17% (**Scheme S5E**). Finally, when the reaction was spiked with the POM-protected bromo quinolone **19a** to assess the role of the benzylic amine linker, [^{18}F]**S8** was still obtained in RCCs of 61 ± 11% (**Scheme S5F**), indicating that the free amine group on the benzylic linker does not significantly interfere with copper-mediated radiofluorination.

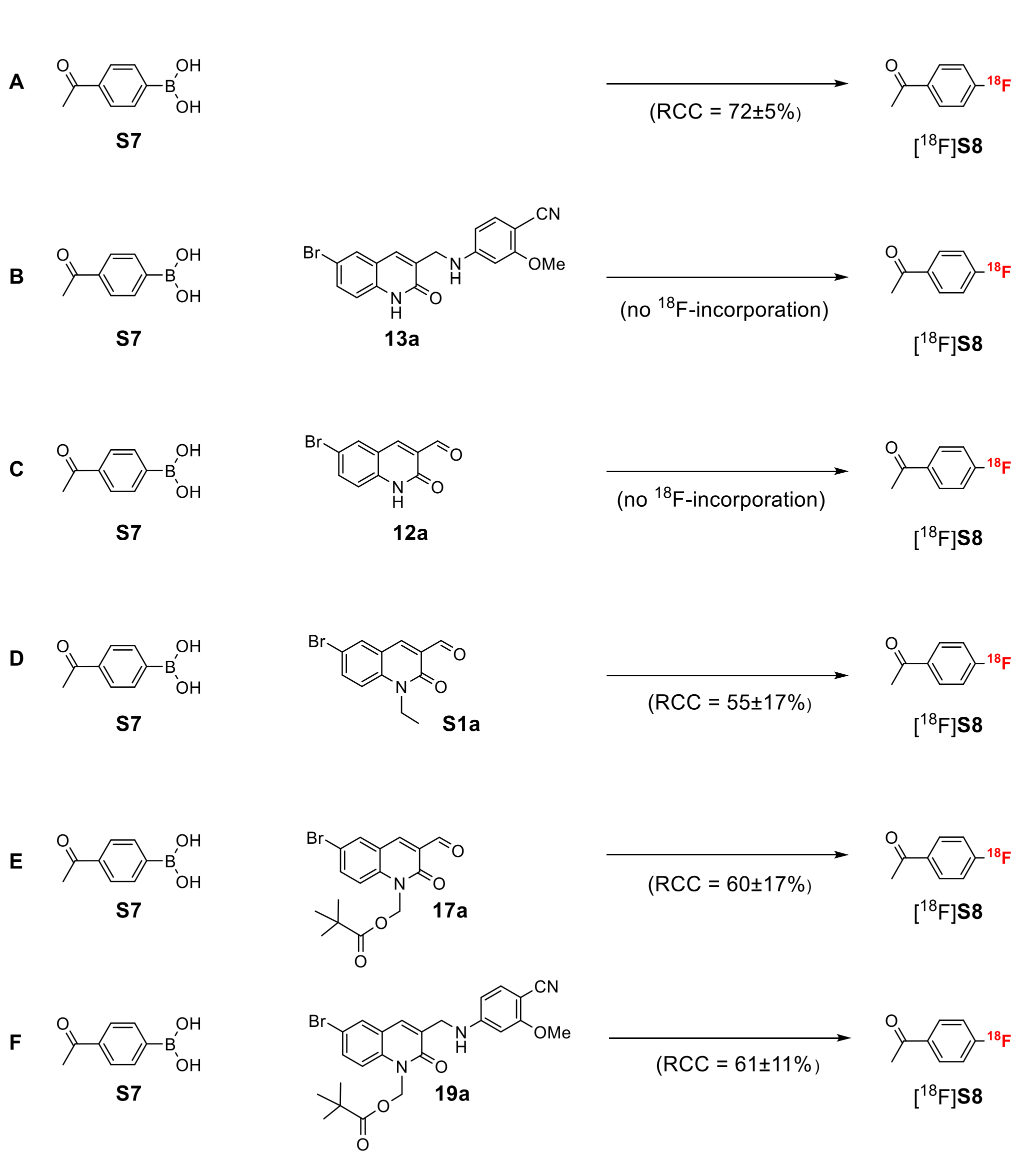

Scheme S5. Effect of different additives on Cu-mediated radiofluorination of **S5**.
Procedure: (i) elution of $[^{18}F]F^-$ with Et_4NHCO_3 in MeOH followed by removal of MeOH; (ii) addition of $Cu(4\text{-PhePy})_4(ClO_4)_2$ and precursors (30 µmol each) in DMA (0.8 mL); (iii) 110°C, 10 min.

3.4.3. Copper mediated radiofluorination of POM-protected precursors **21** and **22**

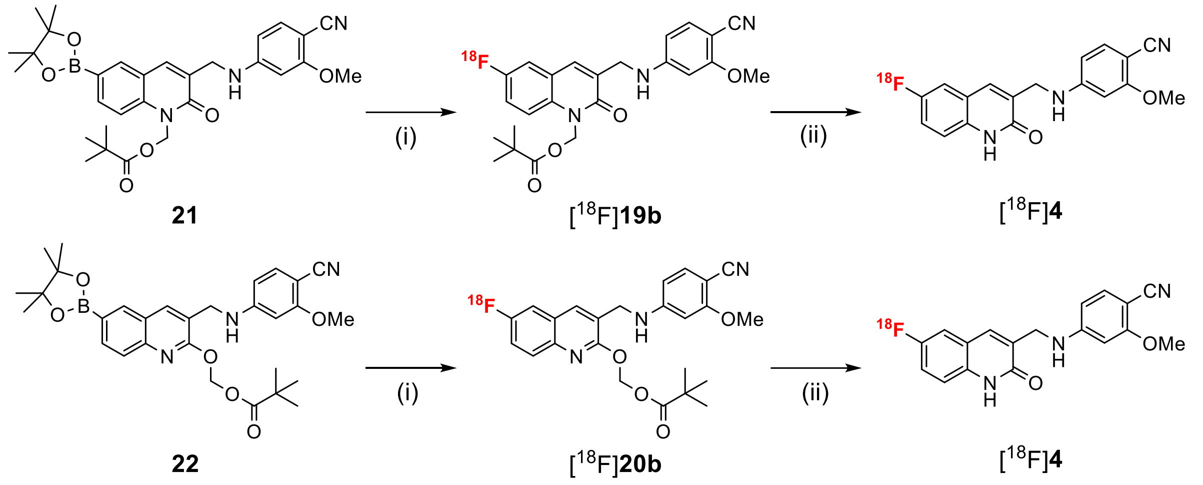

Scheme S6. Radiosynthesis of [^{18}F]**4** by copper-mediated radiofluorination of precursors **21** and **22**.
Conditions: (i) elution of [^{18}F]F^{-} with Et_4NHCO_3 in MeOH followed by evaporation of MeOH, addition of **21** or **22** and Cu(4-PhePy)$_4$(ClO_4)$_2$ (10 µmol of each) in DMA, 110°C, 10 min; ii) 0.25 M $NaOH_{aq}$ in MeOH, 80 °C, 3 min.

Based on the results of the spiking experiments, the radiosynthesis of [^{18}F]**4** was performed by copper-mediated radiofluorination of the *N*- or *O*-POM protected precursors **21** and **22** (**Scheme S6**). To this end, [^{18}F]F^{-} in [^{18}O]H_2O was loaded (from the male side) onto a QMA cartridge and the cartridge was rinsed (from the male side) with MeOH (1 mL) and dried with air (10 mL). The [^{18}F]F^{-} was eluted with a solution of Et_4NHCO_3 (1 mg, 5.22 µmol) in MeOH (1 mL) and the MeOH was removed at 85 °C under reduced pressure (200 bar) within 5 min. A solution of precursor **21** or **22** (5.45 mg, 10 µmol) and Cu(4-PhePy)$_4$(ClO_4)$_2$ (9 mg, 10 µmol) in DMA (0.5 mL) was then added and the reaction mixture was stirred at 110 °C for 10 minutes. The mixture was allowed to cool for a few minutes before the radiolabeled intermediates were de-protected by addition of 0.25 M $NaOH_{aq.}$ (200 µL) in 50% MeOH and stirring at 80 °C for 3 min. An aliquot of the reaction mixture was removed and diluted with MeCN and H_2O to determine the RCCs, while the rest was diluted with H_2O (2 mL) and loaded onto a SPE cartridge. The cartridge was washed with H_2O (6 mL) and the product eluted with 30% MeCN in H_2O (1.5 mL) followed by purification via semi-preparative HPLC (**Figure S1 & S2**). The product fraction was collected, diluted with H_2O (30 mL) and loaded onto a SPE cartridge. After elution of the product with 80% EtOH in H_2O (1 mL) and evaporation of the solvent under full vacuum at 40 °C, the residue was formulated with 1% Tween 80 in phosphate buffer (200 µL) to afford [^{18}F]**4** as a ready-to-inject solution. Using precursor **21**, [^{18}F]**4** was obtained in RCCs of 26 ± 12% (n = 15), RCYs of 22 ± 11% (n = 11), an A_m of 13-180 GBq/µmol (from

1-4 GBq starting activities) (n = 7) and RCPs of >99%. Radiofluorination of precursor **22** afforded [^{18}F]**4** in RCCs of 41 ± 20% (n = 38), RCYs of 29 ± 11% (n = 30), an A_m of 13-540 GBq/µmol (from 1-4 GBq starting activities) (n = 18) and RCPs of >99%. The identity of [^{18}F]**4** was confirmed by HPLC analysis of the purified tracer spiked with the non-radioactive reference compound **4** (**Figure S3**), while efficient separation from the major impurity formed by degradation of the boronic precursor was confirmed by HPLC analysis of the purified tracer spiked with compound **13c** (**Figure S4**).

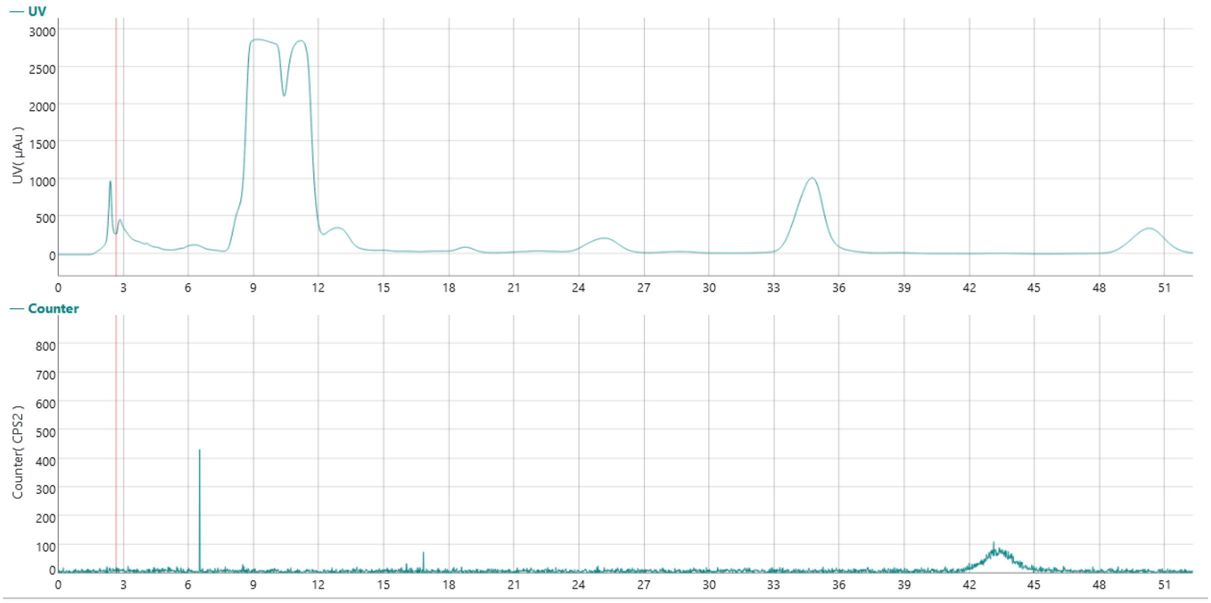

Figure S1. HPLC chromatogram for the purification of [^{18}F]**4** prepared from precursor **21** by semipreparative HPLC (Top: UV chromatogram, 254 nm; bottom: radio chromatogram). Column: Synergi Hydro RP 10µm (80 Å), LC column 250 x 10 mm; eluent: 30% MeCN in H_2O; flow rate: 4.7 mL/min.

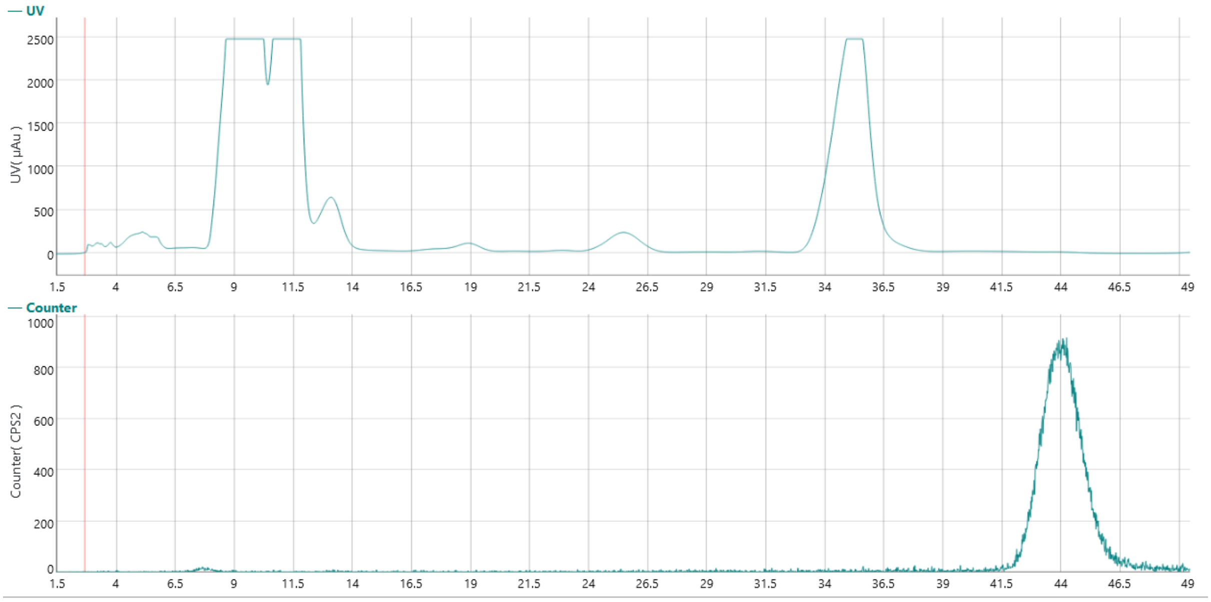

Figure S2. HPLC chromatograms for the purification of [^{18}F]**4** prepared from precursor **22** by semipreparative HPLC (Top: UV chromatogram, 254 nm; bottom: radio chromatogram). Column: Synergi Hydro RP 10µm (80 Å), LC column 250 x 10 mm; eluent: 30% MeCN in H_2O; flow rate: 4.7 mL/min.

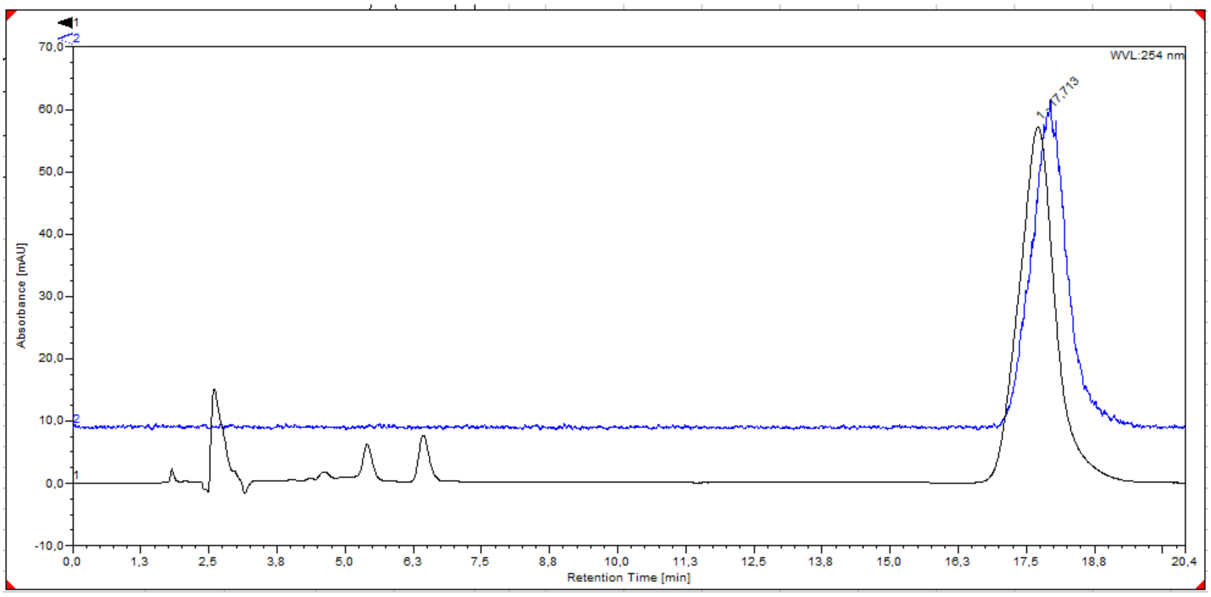

Figure S3. HPLC chromatogram of purified [^{18}F]**4** co-injected with the non-radioactive reference compound **4** (95% pure) (Black: UV chromatogram, 254 nm; blue: radio chromatogram). Column: Synergi Hydro-RP 4 µm (80 Å) 250 × 4.6 mm; eluent: 35% MeCN in H_2O; flow rate: 1 mL/min.

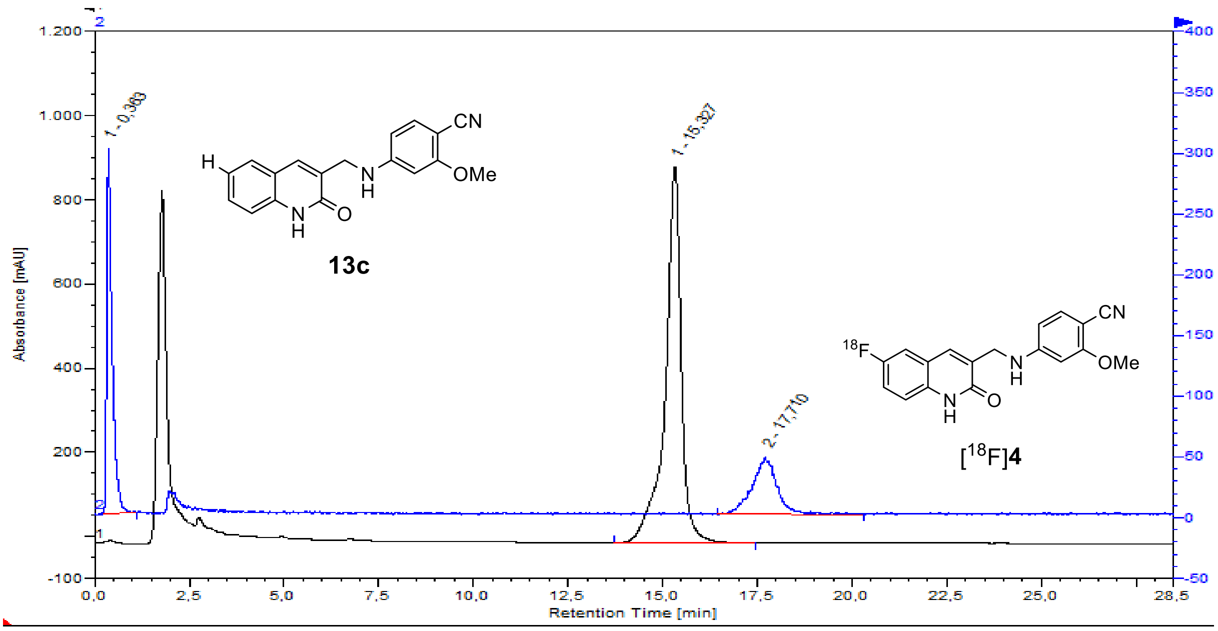

Figure S4. HPLC chromatogram of purified [^{18}F]**4** co-injected with the protodeboronated impurity **13c** (Black: UV chromatogram, 272 nm; blue: radio chromatogram). Column: Synergi Hydro-RP 4 µm (80 Å) 250 × 4.6 mm; eluent: 32% MeCN in H_2O; flow rate: 1 mL/min.

3.4.4. Determination of carrier amount and molar activity

After HPLC purification, the tracer was dissolved in 80% EtOH in H_2O (1 mL) and an aliquot (20 µL) was used to determine the carrier amount and the molar activity by analytical HPLC. The concentration of the carrier was determined from the peak area at 254 nm using a calibration curve (**Figure S5** and **Table S4**) and the molar activity (A_m) was calculated according to:

$$A_m = {}^{Activity\ (GBq)}\Big/_{moles\ of\ carrier\ (\mu mol)} \quad \text{Eq. 1}$$

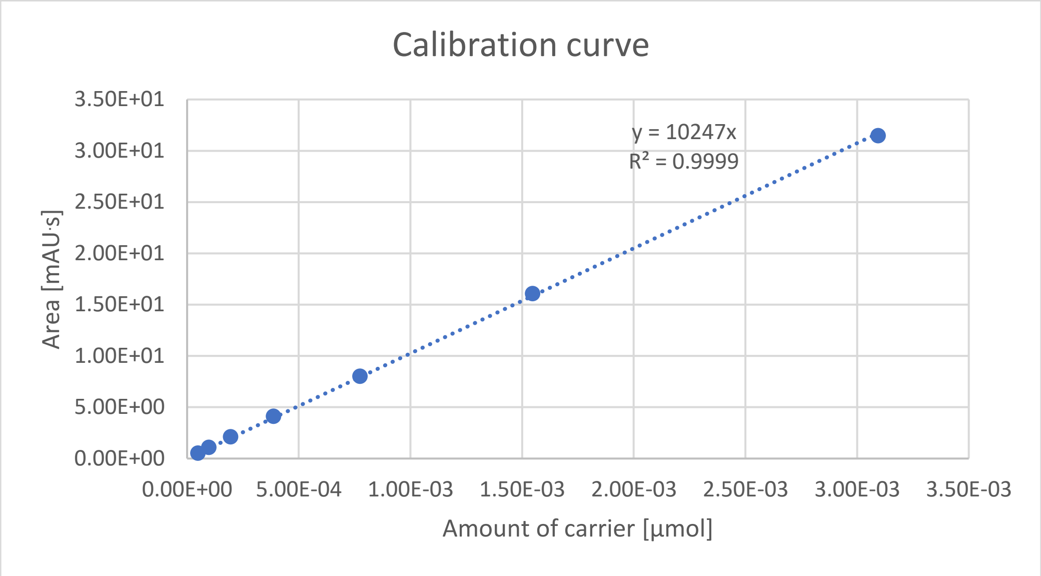

Figure S5. Calibration curve for determination of the molar activity of [^{18}F]**4**.

Table S4. Calibration data for determination of the molar activity of [^{18}F]**4**.

**mg/1 mL**	**mg/20 µL**	**g/20 µL**	**mol (g/323.13 g/mol)**	**µmol**	**Average Area**
**5.00E-02**	1.00E-03	1.00E-06	3.09E-09	3.09E-03	3.15E+01
**2.50E-02**	5.00E-04	5.00E-07	1.55E-09	1.55E-03	1.61E+01
**1.25E-02**	2.50E-04	2.50E-07	7.73E-10	7.73E-04	8.05E+00
**6.25E-03**	1.25E-04	1.25E-07	3.87E-10	3.87E-04	4.12E+00
**3.13E-03**	6.25E-05	6.25E-08	1.93E-10	1.93E-04	2.14E+00
**1.56E-03**	3.13E-05	3.13E-08	9.67E-11	9.67E-05	1.09E+00
**7.81E-04**	1.56E-05	1.56E-08	4.83E-11	4.83E-05	5.57E-01

3.5. Radiosynthesis of (*S*)-[^{18}F]**2** and (*R*)-[^{18}F]**2**

3.5.1. Copper-mediated radiofluorination of (*S*)- and (*R*)-**29** and (*S*)- and (*R*)-**30**

When the radiosynthesis of (*S*)- and (*R*)-[^{18}F]**2** was performed under the same conditions as described above for [^{18}F]**4** (i.e. elution of [^{18}F]F^{-} with Et_4NHCO_3 in MeOH followed by radiofluorination with Cu(4-PhePy)$_4$(ClO$_4$)$_2$ at 110°C for 10 minutes and addition of 0.25 M $NaOH_{aq}$ to cleave the protection groups), no product formation was observed in the case of the *N*-protected precursors (*S*)- and (*R*)-**29**, while use of the *O*-protected precursors (*S*)- and (*R*)-**30** afforded the desired radiotracers in rather low RCCs of roughly 34% (**Table S5**, entry a). Therefore, the reaction conditions for radiofluorination of the *O*-protected precursors were further optimized with regard reaction temperature and time, reaction solvent, [^{18}F]F^{-} elution and precursor amount. As summarized in **Table S5**, ^{18}F-incorporation could neither be improved by an increase (entry b) nor by a decrease (entry c) of the reaction time at 110 °C. In addition, an incremental decrease of the reaction temperature from 110 °C to 80 °C was associated with a progressive decrease of the RCCs after 10 min (entries d-f). Likewise, radiofluorinations performed for 15 min at 90 °C afforded the desired tracers in low RCCs of about 15% (entry g). In contrast, a significant improvement of the RCCs to roughly 57% was observed when the reactions were carried out at 100 °C for 15 min, using either DMA (entry h) or DMI (entry i) as the reaction solvent. In additional experiments, elution of [^{18}F]F^{-} was either carried out with non-basic Et_4NOTf instead of Et_4NHCO_3 (entry j) or with Et_4NHCO_3 in *n*BuOH instead of MeOH (entry k), both of which proved to be detrimental for ^{18}F-incorporation. Likewise, reducing the amount of precursor and copper mediator from 10 µmol to 5 µmol was associated with a decrease of the RCCs to roughly 10% (entry l). Since none of the reaction conditions listed in **Table S5** resulted in any product formation when the *N*-protected precursors (*S*)- and (*R*)-**29** were used (n = 2 per condition), we also examined their effect on radiofluorination of 4-acetylbenzeneboronic acid (**S5**) by spiking experiments analogous to those described in section 3.3.2. However, addition of (*S*)- or (*R*)-**29** did not affect the RCCs of the benchmark reaction (data not shown), suggesting that the lack of ^{18}F-incorporation was not related to interference of the precursors with copper-mediated radiofluorination. As such, the radiosynthesis of (*S*)- and (*R*)-[^{18}F]**2** was performed using the *O*-protected precursors (*S*)- or (*R*)-**30** and the best reaction conditions identified during the optimization studies (i.e. entry i in **Table S5**).

Table S5. Optimization of conditions for radiosynthesis of (*S*)- and (*R*)-[^{18}F]**2** from the O-protected precursors (*S*)- and (*R*)-**30**

Entry	Eluting salt (1 mg/mL)	Solvent	T (°C)	Time	RCC (%)		
a	Et_4NHCO_3 in MeOH	DMA	110	10 min	33.64	±	1.7 (n = 2)
b	Et_4NHCO_3 in MeOH	DMA	110	15 min	13.60	±	0.5 (n = 2)
c	Et_4NHCO_3 in MeOH	DMA	110	5 min	33.38	±	0.9 (n = 2)
d	Et_4NHCO_3 in MeOH	DMA	100	10 min	32.24	±	5.11 (n = 2)
e	Et_4NHCO_3 in MeOH	DMA	90	10 min	31.18	±	6.61 (n = 2)
f	Et_4NHCO_3 in MeOH	DMA	80	10 min	21.01	±	5.08 (n = 2)
g	Et_4NHCO_3 in MeOH	DMA	90	15 min	14.98	±	7.70 (n = 2)
h	Et_4NHCO_3 in MeOH	DMA	100	15 min	57.39	±	3.29 (n = 2)
i	Et_4NHCO_3 in MeOH	DMI	100	15 min	56.57	±	15.84 (n = 7)
j	Et_4NOTf in MeOH	DMI	100	15 min	51.93	±	13.44 (n = 3)
k	Et_4NHCO_3 in *n*BuOH	DMI	100	15 min	21.24	±	5.67 (n = 2)
l*	Et_4NHCO_3 in MeOH	DMI	100	15 min	10.08	±	2.50 (n = 2)

* experiment carried out with 5 µmol of precursor

To this end, [^{18}F]F^- in [^{18}O]H_2O was loaded (from the male side) onto a QMA cartridge and the cartridge was rinsed (from the male side) with MeOH (1 mL) and dried with air (10 mL). The [^{18}F]F^- was eluted with a solution of Et_4NHCO_3 (1 mg, 5.22 µmol) in MeOH (1 mL) and the MeOH was removed at 85 °C under reduced pressure (200 bar) within 5 min. A solution of precursor (*S*)-**30** or (*R*)-**30** (5.6 mg, 10 µmol) and Cu(4-PhePy)$_4$(ClO_4)$_2$ (9 mg, 10 µmol) in DMI (0.5 mL) was then added and the reaction mixture was stirred at 100 °C for 15 minutes. The mixture was allowed to cool for a few minutes before the radiolabeled intermediates were de-protected by addition of 0.25 M $NaOH_{aq.}$ (200 µL) in MeOH and stirring at 80 °C for 3 min, followed by acidification with 5% TFA in MeCN (200 µL). The crude reaction mixture was then purified by SPE, the product was eluted with 28% MeCN in H_2O (1.5 mL) and further purified by semi-preparative HPLC (**Figure S6**). The product fraction (retention time = 35-39 minutes) was collected, diluted with H_2O (to around 3 times of its original volume) and loaded onto a SPE cartridge. Subsequent elution of the products with EtOH (1 mL) afforded (*S*)- or (*R*)-[^{18}F]**2** with a radiochemical conversion of 61 ± 13% (n = 6), radiochemical yield of 50 ± 10% (n = 4), a molar activity of 102-275 GBq/µmol (start activities of about 1 GBq) (n = 2) and a radiochemical purity >99%.

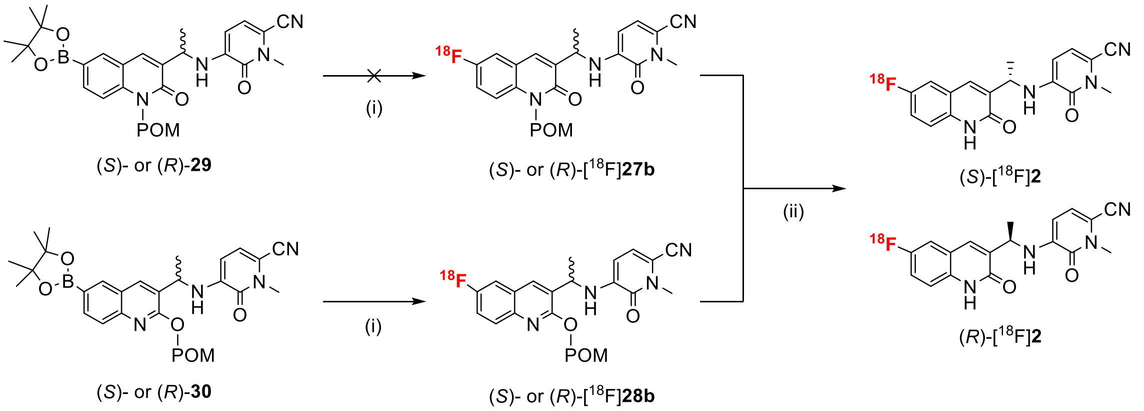

Scheme S7. Radiosynthesis of (*S*)- and (*R*)-[^{18}F]**2** by copper-mediated radiofluorination of precursors (*S*)- or (*R*)-**30**.
Conditions: i) elution of [^{18}F]F^{-} with Et_4NHCO_3 in MeOH followed by evaporation of MeOH, addition of precursor and Cu(4-PhePy)$_4$(ClO_4)$_2$ (10 µmol each) in DMI, 100 °C, 15 min; ii) 0.25 M $NaOH_{aq}$ in MeOH, 80 °C, 3 min followed by quench with 5% TFA in MeCN.

The identity of (*S*)- and (*R*)-[^{18}F]**2** was confirmed by HPLC analysis of the purified tracers spiked with the non-radioactive reference compounds (*S*)- and (*R*)-**2** (**Figure S7**). The protodeboronated impurities, formation of which was confirmed by HPLC analysis**,** could be separated from the desired radiotracers (**Figure S8**) by semi-preparative HPLC and the residual solvent and copper contents were well below the acceptable limit.

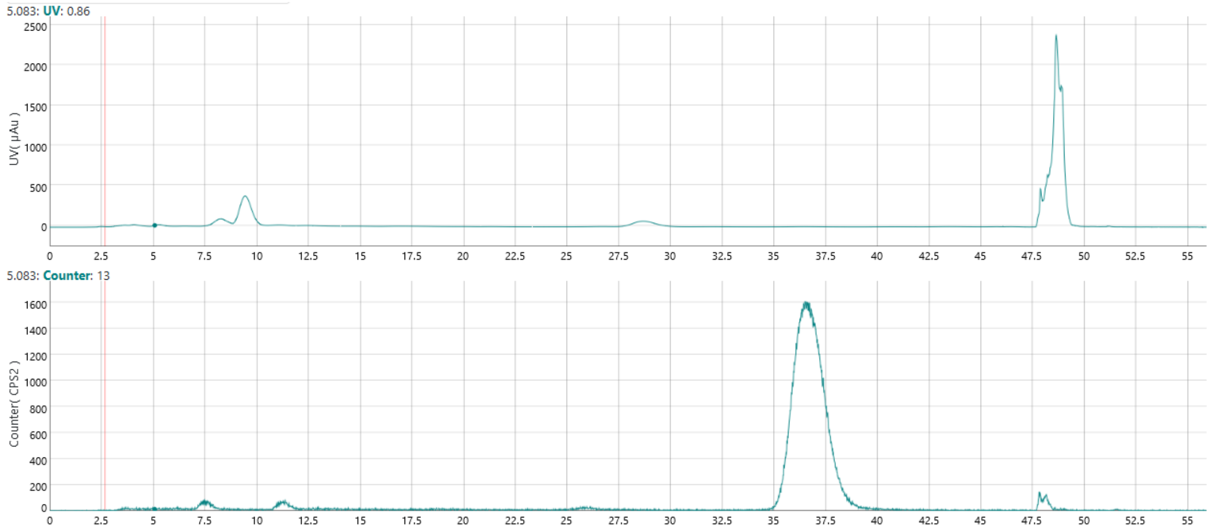

Figure S6. HPLC chromatograms for the purification of (*S*)- or (*R*)-[^{18}F]**2** prepared from precursors (*S*)- or (*R*)-**30** by semi-preparative HPLC (Top: UV chromatogram, 270 nm; bottom: radio chromatogram). Column: Synergi Hydro RP 10µm (80 Å), LC column 250 x 10 mm; eluent: 28% MeCN in H_2O; flow rate: 4.7 mL/min.

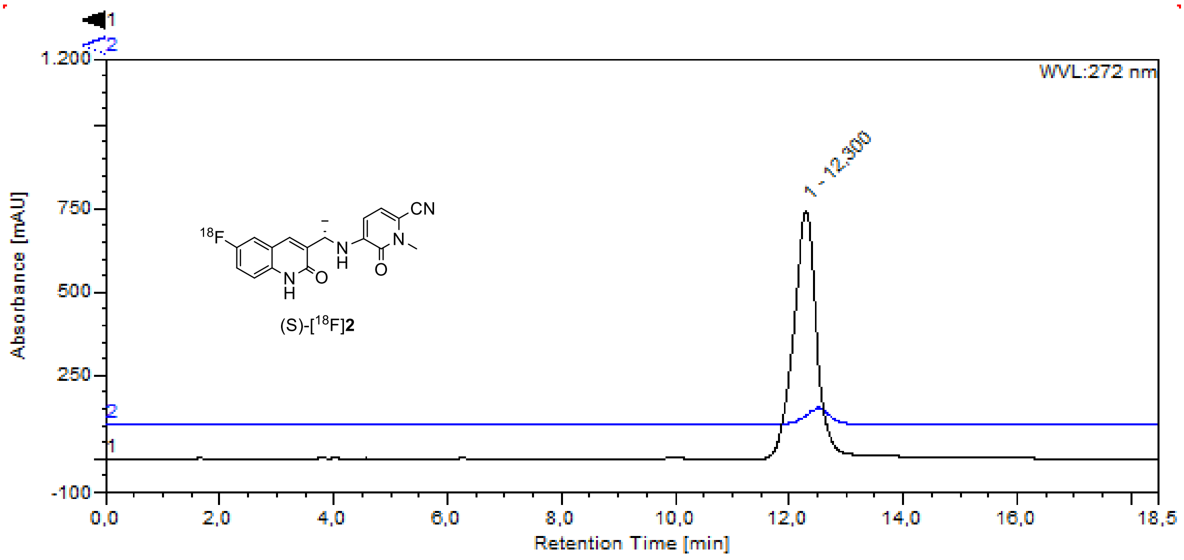

Figure S7. HPLC chromatogram of purified (*S*)-[^{18}F]**2** co-injected with the non-radioactive reference compound (*S*)-**2** (99% pure) (Black: UV chromatogram, 272 nm; blue: radio chromatogram). Column: Synergi Hydro-RP 4 µm (80 Å) 250 × 4.6 mm; eluent: 32% MeCN in H_2O; flow rate: 1 mL/min.

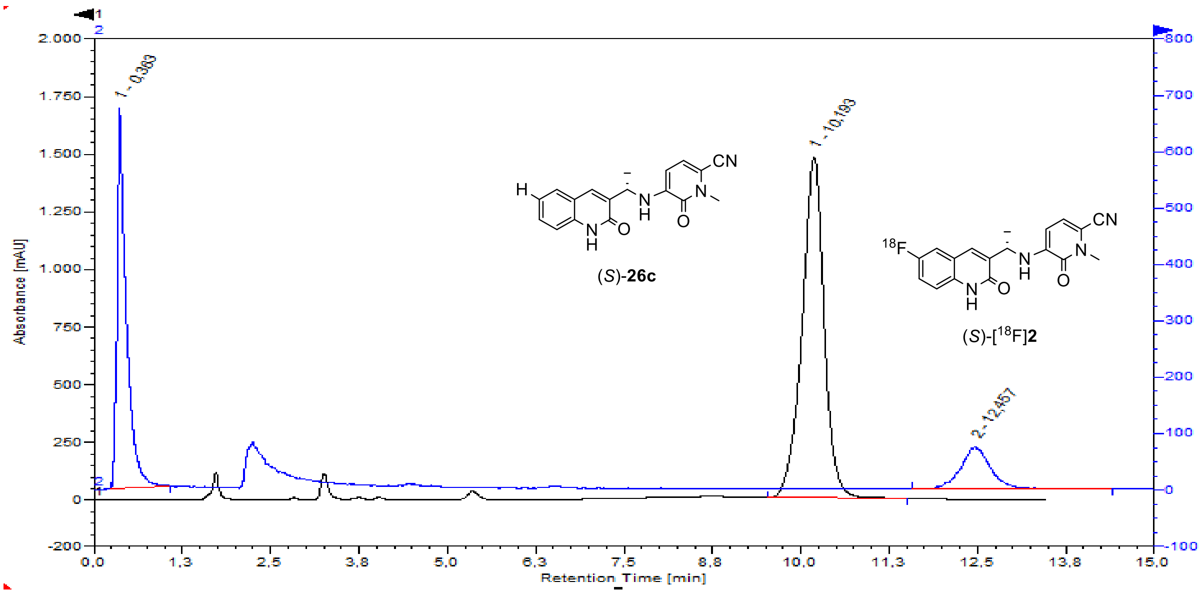

Figure S8. HPLC chromatogram of purified (*S*)-[^{18}F]**2** co-injected with the protodeboronated impurity (*S*)-**26c** (Black: UV chromatogram, 272 nm; blue: radio chromatogram). Column: Synergi Hydro-RP 4 µm (80 Å) 250 × 4.6 mm; eluent: 32% MeCN in H_2O; flow rate: 1 mL/min.

3.5.2. Determination of carrier amount and molar activity

The calibration curve for calculation of the molar activity of (*S*)- and (*R*)-[^{18}F]**2** was measured at 272 nm (**Figure S9** and **Table S6**).

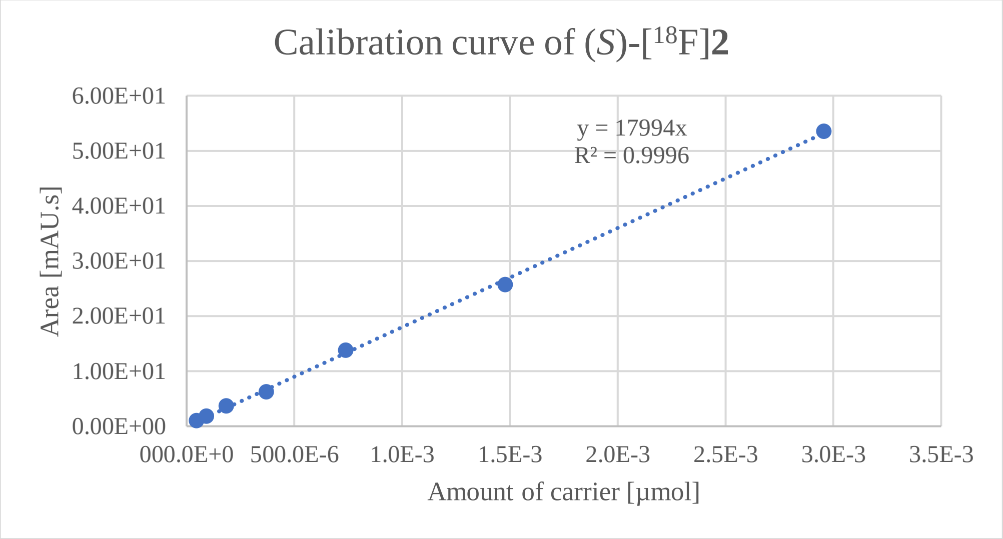

Figure S9. Calibration curve for determination of the molar activities of (*S*)- and (*R*)-[^{18}F]**2**.

Table S6. Calibration data for determination of the molar activities of (*S*)- and (*R*)-[^{18}F]**2**.

mg/1 mL	mg/20 µL	g/20 µL	mol (g/323.13 g/mol)	µmol	Average Area
**5.00E-02**	1.00E-03	1.00E-06	2.96E-09	2.69E-03	5.35E+01
**2.50E-02**	5.00E-04	5.00E-07	1.48E-09	1.48E-03	2.57E+01
**1.25E-02**	2.50E-04	2.50E-07	7.39E-10	7.39E-04	1.38E+00
**6.25E-03**	1.25E-04	1.25E-07	3.69E-10	3.69E-04	6.23E+00
**3.13E-03**	6.25E-05	6.25E-08	1.85E-10	1.85E-04	3.65E+00
**1.56 E-03**	3.12E-05	3.13E-08	9.24E-11	9.24E-05	1.82E+00
**7.81E-04**	1.56E-05	1.56E-08	4.62E-11	4.62E-05	1.02E+00

3.5.3. Determination of absolute configuration

Methylation of sulfinimines introduces a chiral center, which results in two diastereoisomers with a good diastereomeric excess. The major product results from the nucleophile's attack on the face occupied by the chiral auxiliary, while the minor product results from the attack on the opposite face. Following the methylation of compounds **23a-c**, it was possible to identify and

isolate the two diastereoisomers. The *S*,*R*-stereochemistry was assigned to the major product [(*S*,*R*)-**24a-c**], while the *R*,*R*-stereochemistry was assigned to the minor product [(*R*,*R*)-**24a-c**]. This assignment was applied to all subsequent intermediates of the syntheses and to the final reference compounds (*S*)- or (*R*)-**2** and radiofluorination precursors (*S*)- or (*R*)-**29** and (*S*)- or (*R*)-**30**, since it was impossible to verify their absolute stereochemistry by X-ray technique and none of them was literature known. The optical rotation of the reference compounds and *O*- and *N*-protected precursors was analyzed by polarimetry (Krüss P8000-TF), and the opposite chirality of each pair was confirmed (**Table S7**). After publication of the work by Weber et al, who also assigned the absolute configuration via circular dichroism, it was possible to compare the NMR spectra of the fluorinated intermediates (*S*,*S*)-**24b** and (*R*,*R*)-**24b** with those reported in their work. The reported stereochemistry was in line with that assigned to all intermediates and final compounds on the base of literature. After the stereochemistry of compounds (*S*,*S*)-**24b** and (*R*,*R*)-**24b** had been confidently assigned, it was also possible to infer the absolute stereochemistry of the reference compounds and, assuming that radiofluorination could not have resulted in a complete reversal of the stereochemistry at the methyl site, of the radiotracers. Indeed, by means of chiral HPLC it was possible to confirm an enantiomeric excess of 100% for the radiotracers prepared by radiofluorination of (*R*)-**30** and (*S*)-**30**. The assignment of the stereochemistry was performed by chiral HPLC analysis of the radiotracers spiked with the non-radioactive reference compounds (**Figure S10** and **S11**). The absolute configuration of the 6-H products was not assigned due to their role as reference compounds for analytical HPLC chromatography.

Table S7. Optical rotation of reference compounds and precursors[a].

Compound	Temperature	N (mg/mL)	Optical rotation	Specific rotation
**(*S*)-2**	20.1 °C	0.187	0.162	86.6
**(*R*)-2**	20.1 °C	0.124	-0.079	-63.7
**(*S*)-29**	20.4 °C	0.095	-0.051	-53.7
**(*R*)-29**	20.2 °C	0.154	0.052	43.8
**(*S*)-30**	20.1 °C	0.086	-0.103	-119.8
**(*R*)-30**	20.4 °C	0.116	0.090	79.9

[a] **For analysis of the optical rotation and specific rotation, reference compounds (*S*)- or (*R*)-2 and radiofluorination precursors (*S*)- or (*R*)-29 and (*S*)- or (*R*)-30 were dissolved in $CHCl_3$ (~1 mg/mL) and injected into the cell of the polarimeter.** Baseline-correction was performed based on **a blank measurement** with **pure** $CHCl_3$**.**

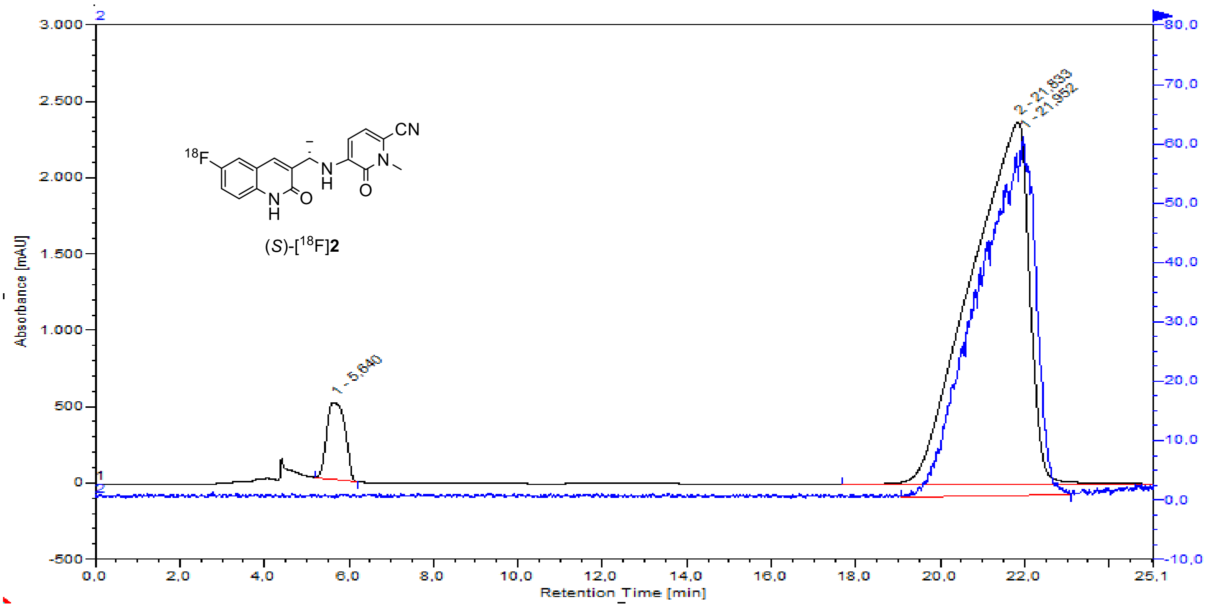

Figure S10. HPLC chromatogram of purified (*S*)-[^{18}F]**2** co-injected with the non-radioactive reference compound (*S*)-**2** (>99% pure) (Black: UV chromatogram, 272 nm; blue: radio chromatogram). Column: CHIRALPACK AD, 10 µm (80 Å) 250 × 4.6 mm; eluent: 20% *i*PrOH in hexane; flow rate: 1 mL/min.

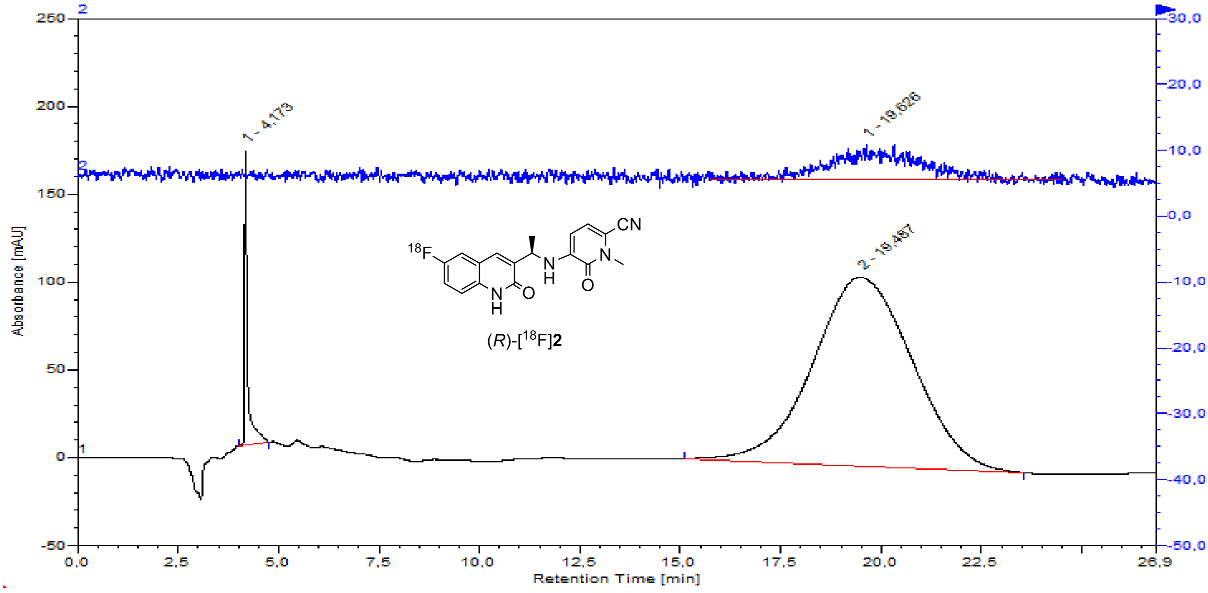

Figure S11. HPLC chromatogram of (*R*)-[^{18}F]**2** co-injected with the non-radioactive reference compound (*R*)-**2** (>99% pure) (Black: UV chromatogram, 272 nm; blue: radio chromatogram). Column: CHIRALPACK AD, 10 µm (80 Å) 250 × 4.6 mm; eluent: 20% *i*PrOH in hexane; flow rate: 1 mL/min.

3.6. Radiosynthesis of [^{18}F]**5**

3.6.1. Aliphatic radiofluorination of precursor **39**

Radiofluorination of the *O*-protected precursor **39** was initially performed according to the conventional protocol for aliphatic radiofluorination in MeCN, while preprocessing of [^{18}F]F^{-} was carried out with the same "minimalist-like" approach as described above for the other tracers. Accordingly, [^{18}F]F^{-} was loaded onto a QMA cartridge, eluted with a solution of TEAB (1 mg, 5.22 µmol) in MeOH (1 mL) and the MeOH was removed under reduced pressure at 85 °C. A solution of precursor **39** (10 µmol) in MeCN (0.5 mL) was then added to the reactor, the reactor was sealed with a rubber septum, and the reaction mixture was stirred at 85 °C for 10 min. The POM-protecting group in the radiolabeled intermediate was then cleaved by addition of 0.25 M $NaOH_{aq}$ (0.2 mL) and stirring of the resulting mixture at 85 °C for 3 minutes (**Table S8**). This procedure yielded [^{18}F]**5** without radioactive side-product but in low RCCs of 9.67 ± 3.20% (entry a), which could not be improved by increasing the reaction time to 15 minutes (entry b).

Table S8. Radiosynthesis of [^{18}F]**5.**

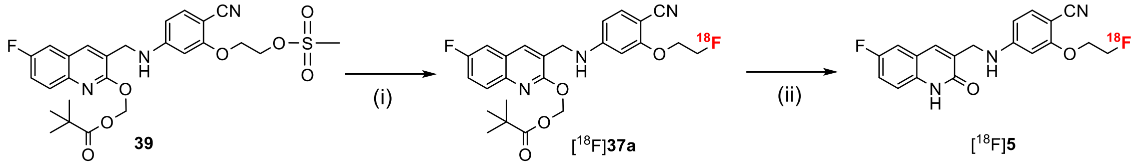

Entry	39 [µmol]	Solvent	T	Time	Salt in MeOH	RCC [%]
a	10 (5.4 mg)	MeCN	85 °C	10 min	1 mg/mL Et_4NHCO_3	9.67 ± 3.20 (n = 3)
b	10 (5.4 mg)	MeCN	85°C	15 min	1 mg/mL Et_4NHCO_3	8.30 ± 2.99 (n = 3)

Conditions: i) elution of [^{18}F]F^{-} with Et_4NHCO_3 in MeOH followed by removal of MeOH and addition of precursor **39** (10 µmol) in MeCN, 85 °C, 10 min; ii) 0.25 M $NaOH_{aq}$, 80 °C, 3 min.

HPLC analysis of a test reaction before and after deprotection showed that addition of NaOH was associated with a drastic increase in the amount of [^{18}F]F^{-}, suggesting that the deprotection step resulted in degradation of the base-sensitive [^{18}F]fluoroethyl substituent. Accordingly, a series of optimization experiments was performed to identify more suitable reaction conditions for cleavage of the POM-protecting group. To this end, radiofluorination of precursor **39** was carried out as described above (**Table S8**, entry a) and the conditions for deprotection of the

radiolabeled intermediate [^{18}F]**37a** (concentration, volume and solvent of NaOH solution, temperature and duration) were varied (**Table S9**).

Table S9. Optimization of the reaction conditions for deprotection of **[^{18}F]37a**.

Entry	T	Time	Volume	NaOH	Impurity (4.4 min)	[^{18}F]**5** (12.56 min)	[^{18}F]**37a** (21.80 min)
a	85 °C	3 min	200 µL	0.05 M in H_2O	6.0%	4.0%	2.0%
b	rt	10 min	200 µL	0.25 M in H_2O	9.4%	31.5%	0%
c	rt	10 min	200 µL	0.25 M in H_2O	0%	24.0%	27%
d	rt	10 min	200 µL	0.25 M in H_2O	3.0%	2.4%	2.9%
e	rt	10 min	200 µL	0.25 M in H_2O	3.7%	5.8%	5.0%
f	30 °C	10 min	175 µL	0.1 M in EtOH	6.3%	14.4%	3.3%
g	30 °C	10 min	175 µL	0.1 M in EtOH	9.8%	7.2%	0%
h	30 °C	7 min	175 µL	0.1 M in EtOH	0%	46.0%	4.7%
i*	30 °C	10 min	175 µL	0.1 M in EtOH	0%	47.4%	0%
j	30 °C	10 min	175 µL	0.1 M in EtOH	0%	10.0%	0%
k	35 °C	5 min	200 µL	0.1 M in EtOH	0%	30.3%	5.4%
l	35 °C	10 min	200 µL	0.1 M in EtOH	0%	24.0%	0%

* An aliquot was taken and analyzed without previous addition of water

Use of a more dilute solution of NaOH (0.05 M) resulted in formation of an undefined polar impurity and incomplete deprotection of [^{18}F]**37a**, so that [^{18}F]**5** was obtained in even lower RCCs (entry a). A reduction of the reaction temperature in combination with an increase of the reaction time to 10 min was often but not always associated with higher RCCs (entries b-e). Moreover, complete deprotection of **[^{18}F]37a** was not consistently achieved and formation of the polar impurity was typically also observed. Finally, replacement of aqueous NaOH with an ethanolic solution of NaOH led to improved RCCs. Thus, when a smaller volume of 0.1 M NaOH in EtOH (175 µL) was added after the radiofluorination and the mixture stirred at 30 °C for 10 min, the desired radiotracer was formed as the main product (entries f-j). Analysis of aliquots of the mixture removed after 7 and 10 min (entries h and i) indicated that a reaction time of 10 min was required to fully deprotect the intermediate [^{18}F]**37a**. Moreover, a slight

increase of the volume and temperature seemed reasonable to ensure complete conversion of [^{18}F]**37a** to [^{18}F]**5**, since deprotection of the intermediate was in some cases incomplete (probably due to loss of small amounts of the basic solution on the reactor walls). Accordingly, additional test reactions were performed with 200 µL of the solution at 35 °C, which resulted in incomplete deprotection after 5 min (entry k) but exclusive formation of [^{18}F]**5** after 10 min (entry l).

Table S10. Optimization of radiofluorination conditions for the preparation of **[^{18}F]37a**.

Entry	39 [µmol]	Solvent	T [°C]	Time [min]	Salt in MeOH	RCC (%)
a	10 (5.4 mg)	MeCN	85	10	1 mg Et_4NHCO_3 / 1 mL	5.30 ± 4.30 (n = 3)
b	10 (5.4 mg)	MeCN	85	10	0.5 mg Me_4NOTf / 0.5 mL	21.02 ± 5.64 (n = 3)
c	10 (5.4 mg)	MeCN	85	15	0.5 mg Me_4NOTf / 0.5 mL	23.51 ± 1.48 (n = 2)
d	3.5 (2 mg)	MeCN	85	15	0.5 mg Me_4NOTf / 0.5 mL	32.82 ± 9.93 (n = 3)
e	1.8 (1 mg)	MeCN	85	15	0.5 mg Me_4NOTf / 0.5 mL	24.14 ± 4.57 (n = 2)
f	3.5 (2 mg)	DMF	100	15	0.5 mg Me_4NOTf / 0.5 mL	5.22 ± 0.22 (n = 2)
g	3.5 (2 mg)	DMSO	100	15	0.5 mg Me_4NOTf / 0.5 mL	24.47 ± 2.78 (n = 2)
h	3.5 (2 mg)	DMSO	120	15	0.5 mg Me_4NOTf / 0.5 mL	12.71 ± 4.13 (n = 2)

Next the radiofluorination conditions were optimized with regard to the elution salt, reaction solvent, reaction temperature and reaction time (Table 10). To this end, the reaction was first carried out with 10 µmol of precursor **39** in 500 µL MeCN at 85°C for 10 minutes, followed by addition of 200 µL H_2O and removal of an aliquot for determination of the RCC. Under these conditions, elution of [^{18}F]F^- with 1 mg/mL Et_4NHCO_3 in MeOH yielded the protected intermediate [^{18}F]**37a** in RCCs of <10% (entry a). In contrast, elution of [^{18}F]F^- with 0.5 mL of a 1 mg/mL solution of the non-basic Me_4NOTf (1.66 µmol) resulted in RCCs of 20 ± 9.90 % (entry b), indicating that a lower basicity improves the conversions, presumably by reducing degradation of the precursor. An increase of the reaction time to 15 minutes (entry c) as well as a reduction of the precursor amount to 3.5 µmol (entry d) also increased the RCCs, while a

further decrease of the precursor amount to 1.8 µmol proved to be detrimental (entry e). Finally, replacement of MeCN by DMF or DMSO and radiofluorination at 100 °C (entries f and g) or 120 °C (entry h) resulted in lower RCCs, so that conditions (d) were selected for all further experiments and used to prepare the radiotracer for the *in vitro* evaluations (Scheme S8).

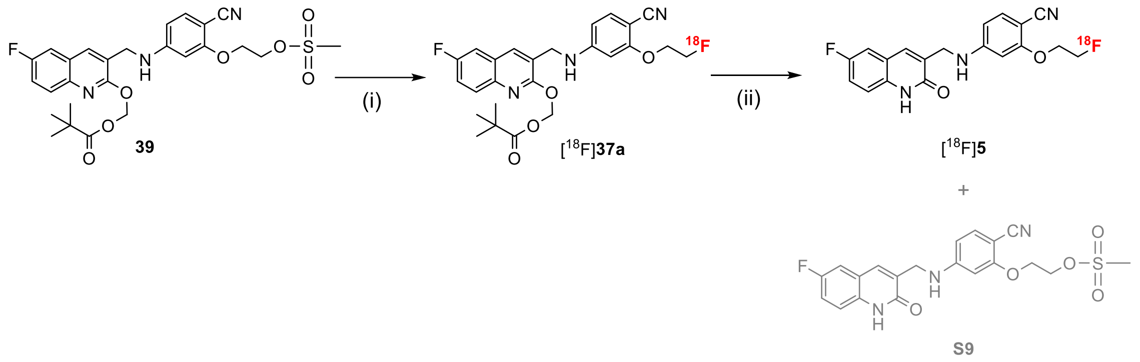

Scheme S8. Radiosynthesis of [^{18}F]**5**.
Conditions: i) Elution of [^{18}F]F^{-} with Me_4NOTf in MeOH, followed by removal of MeOH and addition of precursor **39** (2 mg) in MeCN (0.5 mL), 85 °C, 15 min; ii) 0.1 M NaOH in EtOH (100 µL), 35 °C, 10 min.

Since the amount of precursor was decreased to 2 mg, the amount of NaOH solution used for de-protection was also decreased to 100 µL. Under these conditions, [^{18}F]**5** was obtained in RCCs of 30 ± 8% (n = 6) after de-protection. The reaction mixture was quenched with H_2O (400 µL) and the resulting crude product solution was directly purified by semi-preparative HPLC (**Figure S12**). After isolation, the desired radiotracer [^{18}F]**5** was obtained in radiochemical yields of 26 ± 8% (n = 3), with a molar activity of 14-47 GBq/µmol (n = 4) (from around 1 GBq starting activity) and a radiochemical purity of >99%.

The identity of [^{18}F]**5** was confirmed by HPLC analysis of the purified tracer spiked with the non-radioactive reference compound **5** (**Figure S13**). Despite the mild deprotection conditions, a small amount of unreacted precursor was deprotected, which resulted in formation of 2-(2-cyano-5-(((6-fluoro-2-oxo-1,2-dihydroquinolin-3-yl)methyl)amino)phenoxy)ethyl methanesulfonate (**S9**) (**Scheme S8**). However, despite similar retention times (**Figure S14**), **S9** could be successfully separated from the radiotracer by semi-preparative HPLC, albeit at the expense of increased separation times.

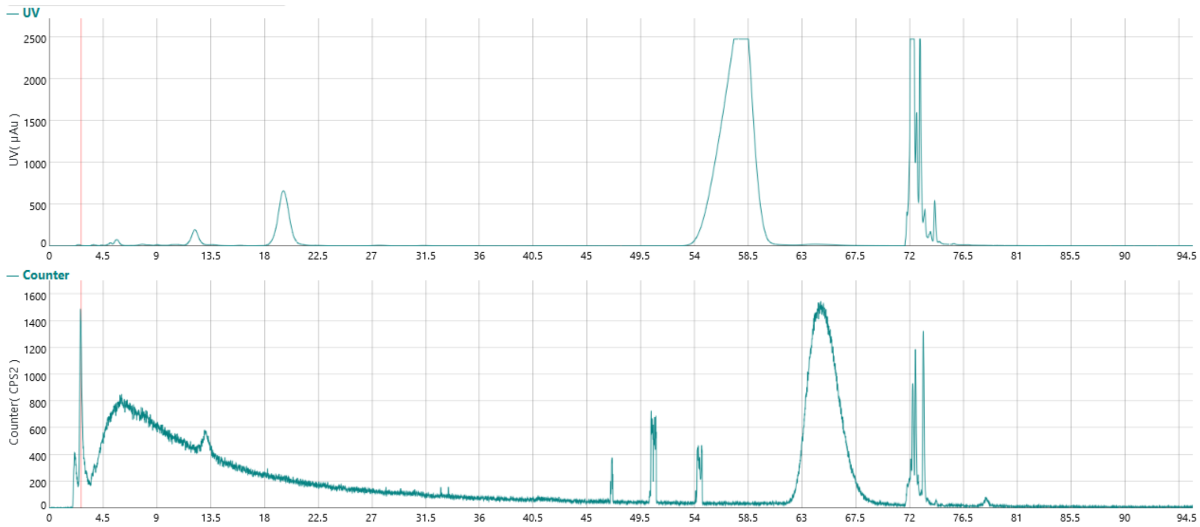

Figure S12. HPLC chromatograms for the purification of [^{18}F]**5** by semipreparative HPLC (Top: UV chromatogram, 270 nm; bottom: radio chromatogram). Column: Synergi Hydro RP 10µm (80 Å), LC column 250 x 10 mm; eluent: 35% MeCN in H_2O + 0.1% TFA; flow rate: 4.7 mL/min.

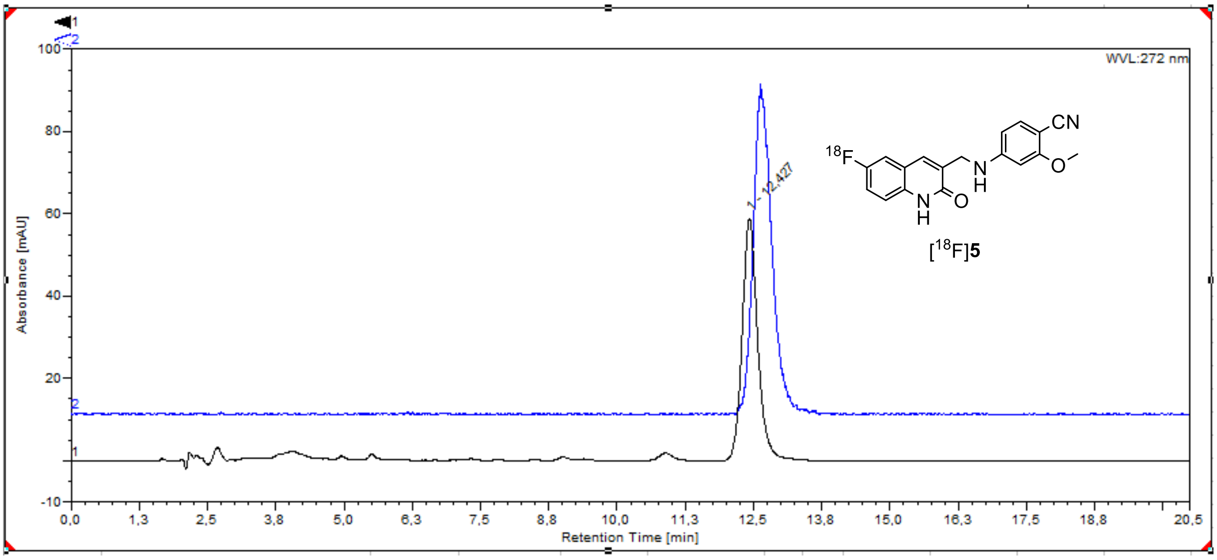

Figure S13. HPLC chromatogram of purified [^{18}F]**5** co-injected with the non-radioactive reference compound **5** (96% pure) (Black: UV chromatogram, 272 nm; blue: radio chromatogram). Column: Synergi Hydro-RP 4 µm (80 Å) 250 × 4.6 mm; eluent: 35% MeCN in H_2O + 0.1% TFA; flow rate: 1 mL/min.

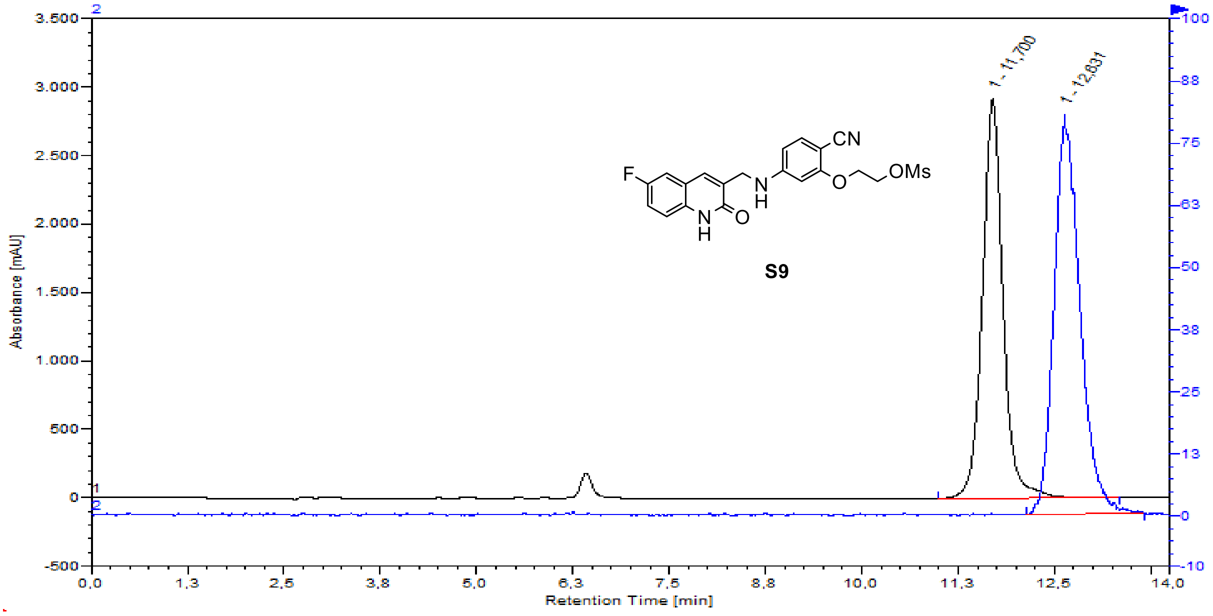

Figure S14. HPLC chromatogram of purified **[^{18}F]5** co-injected with the deprotected precursor **S9** (Black: UV chromatogram, 272 nm; blue: radio chromatogram). Column: Synergi Hydro-RP 4 µm (80 Å) 250 × 4.6 mm; eluent: 35% MeCN in H_2O + 0.1% TFA; flow rate: 1 mL/min.

For optimization of the radiofluorination conditions, the radiolabeled intermediate **[^{18}F]37a** was identified by co-injection of the corresponding protected reference compound **37a** (**Figure S15**).

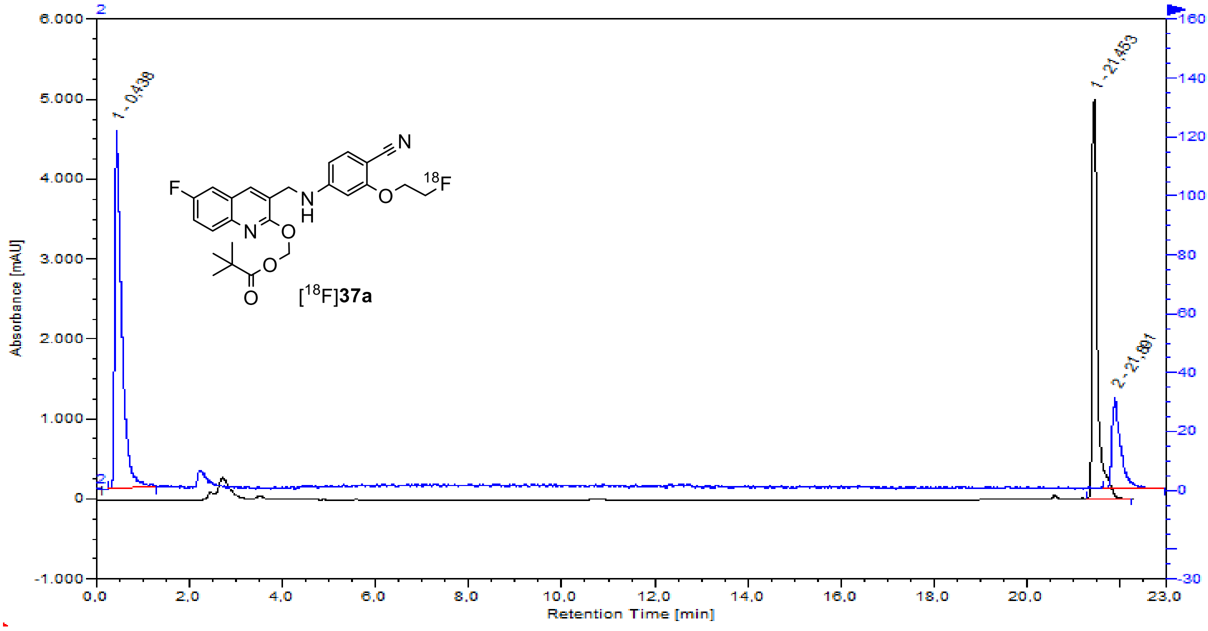

Figure S15. HPLC chromatogram of **[^{18}F]37a** co-injected with the non-radioactive reference compound **37a** (Blue: UV chromatogram, 272 nm; black: radio chromatogram). Column: Synergi Hydro-RP 4 µm (80 Å) 250 × 4.6 mm; eluent: 35% MeCN in H_2O + 0.1% TFA; flow rate: 1 mL/min.

3.6.2 Determination of carrier amount and molar activity

The calibration curve for calculation of the molar activity of [^{18}F]**5** was measured at 272 nm (**Figure S16** and **Table S11**).

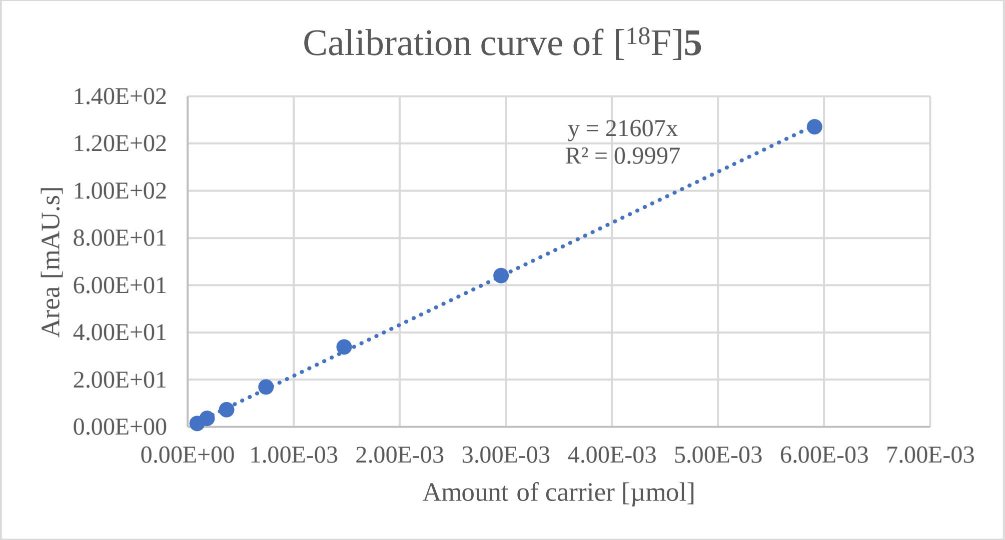

Figure S16. Calibration curve for determination of the molar activity of [^{18}F]**5**.

Table S11. Calibration data for determination of the molar activity of [^{18}F]**5**.

mg/1 mL	mg/20 µL	g/20 µL	mol (g/323.13 g/mol)	µmol	Average Area
**1.00E-01**	2.00E-03	2.00E-06	5.91E-09	5.91E-03	1.27E+02
**5.00E-02**	1.00E-03	1.00E-06	2.96E-09	2.96E-03	6.41E+01
**2.50E-02**	5.00E-04	5.00E-07	1.48E-09	1.48E-03	3.39E+01
**1.25E-02**	2.50E-04	2.50E-07	7.39E-10	7.39E-04	1.68E+01
**6.25E-03**	1.25E-04	1.25E-07	3.69E-10	3.69E-04	7.22E+00
**3.13E-03**	6.25E-05	6.25E-08	1.85E-10	1.85E-04	3.60E+00
**1.56E-03**	3.13E-05	3.13E-08	9.24E-11	9.24E-05	1.49E+00
**7.81E-04**	1.56E-05	1.56E-08	4.62E-11	4.62E-05	9.52E-01

4. Preclinical evaluation

4.1 *In vitro* stability and inhibitory potency

4.1.1. *In vitro* stability in DMSO

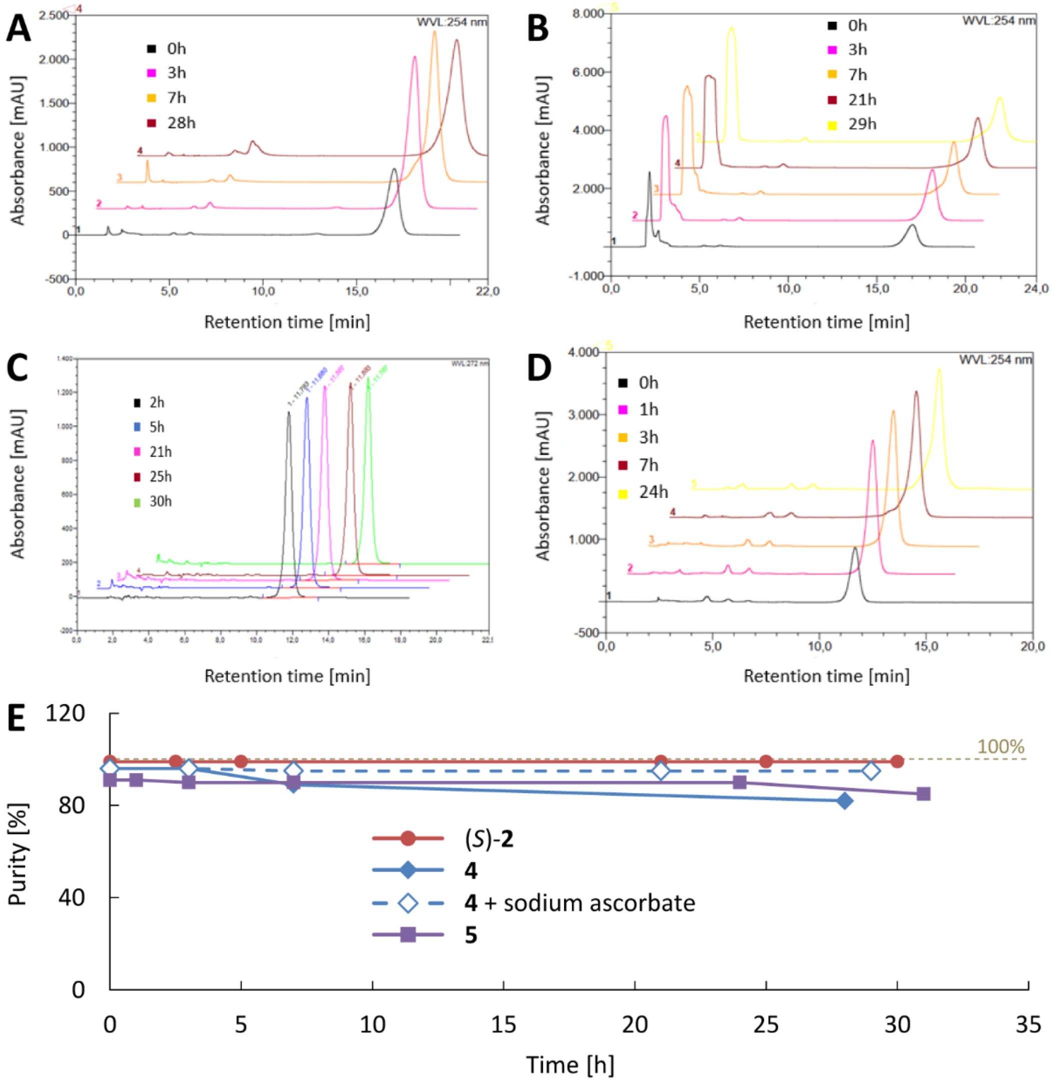

Figure S17: Stability of the reference compounds in DMSO. Shown are stacked chromatograms obtained after incubation of (A) **4**, (B) **4** + 10 mg/mL sodium ascorbate, (C) (*S*)-**2** or (D) **5** in DMSO for the indicated time-periods. Panel E illustrates the fraction of intact compounds determined from the data in A-D and plotted as a function of time.

4.1.2. *In vitro* stability in phosphate-buffered saline (PBS)

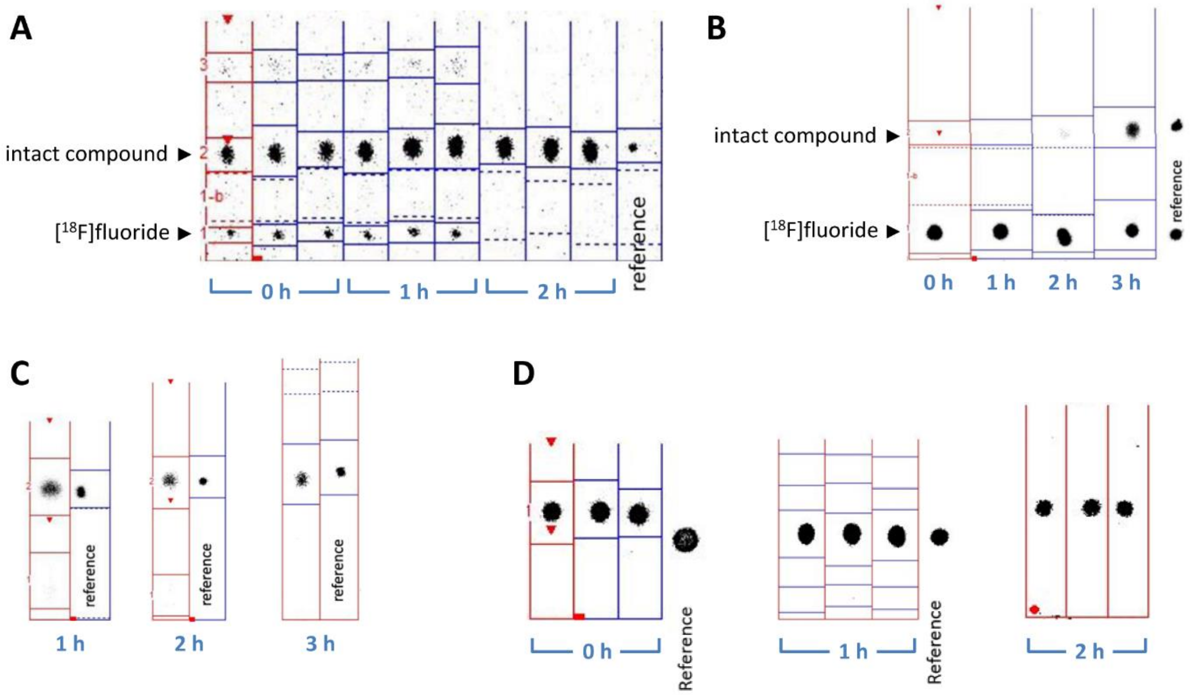

Figure S18: Stability of the candidate probes in PBS. Shown are radio TLC chromatograms (developed with 70-80% EtOAc in hexane) obtained after incubation of (A) [^{18}F]**4**, (B & C) (*S*)-[^{18}F]**2** or (D) [^{18}F]**5** in PBS for the indicated time periods. Note that silica-induced degradation of the samples collected during early time-points was evident when the TLC plates were developed after collection of the last sample (A & B), while no defluorination was observed when the samples were spotted on separate TLC plates that were immediately developed (C & D).

4.1.3. *In vitro* stability in rat serum

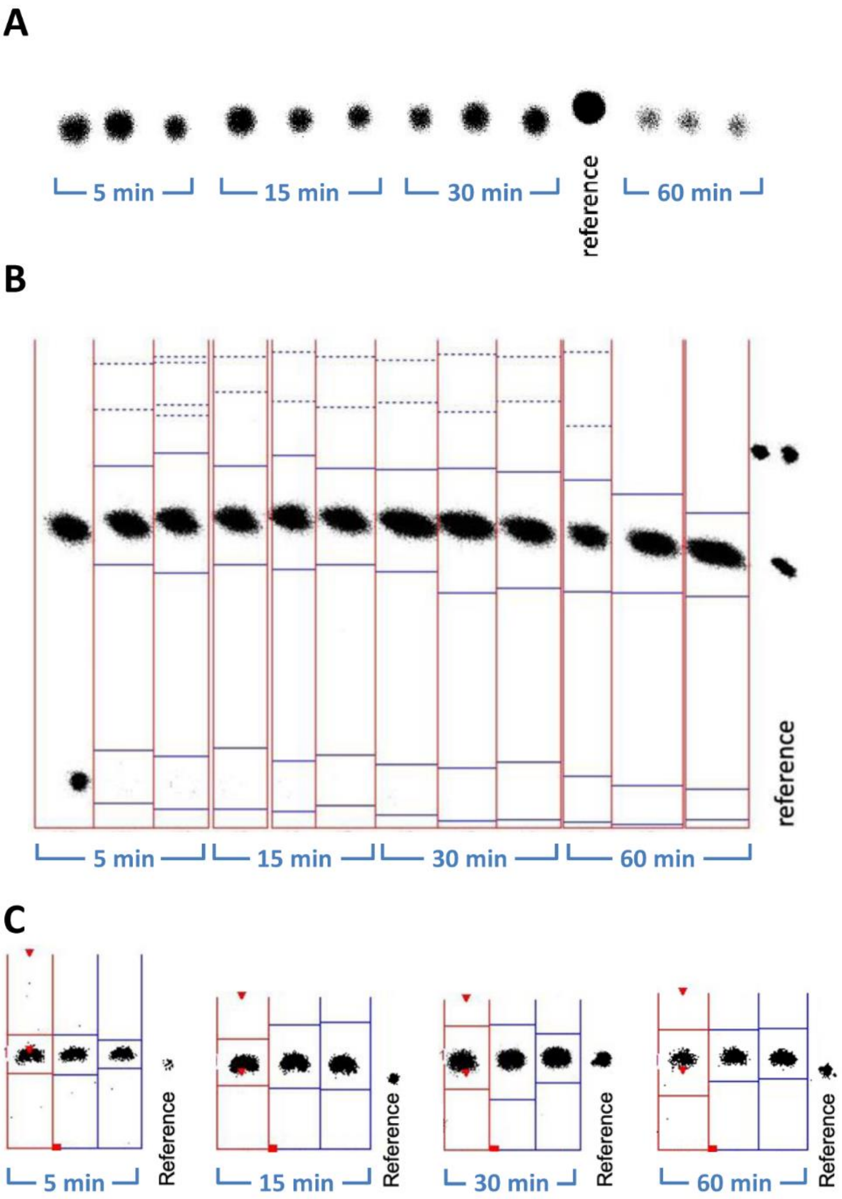

Figure S19: Stability of the candidate probes in rat serum. Shown are radio TLC chromatograms (developed with 70-80% EtOAc in hexane) obtained after incubation of (A) [^{18}F]**4,** (B) (*R*)-[^{18}F]**2** or (C) [^{18}F]**5** in rat serum at 37 °C for the indicated time periods.

4.1.4. Inhibitory potency (IC_{50})

Table S12. Fit parameters for inhibition of $mIDH1_{R132H}$ by the fluorinated olutasidenib derivatives.

	(*S*)-**2**	(*R*)-**2**	**4**	**5**
A_{min} [%]	33 (30−35)	28 (25−31)	1.8 (0.6−3.0)	3 (2−3)
A_{max} [%]	95 (93−97)	79 (72−87)	98 (96−100)	41 (36−45)
IC_{50} [nM]	102 (93−111)	1358 (1104−1671)	713 (684−745)	8279 (7047−9727)
Hill slope	5.6 (4.0−7.2)	3.0 (1.3−4.8)	2.8 (2.5−3.0)	2.1 (1.6−2.7)
R^2	0.9861	0.9402	0.9974	0.9852

Values in parenthesis indicate 95% confidence intervals.

5. NMR Spectra

5.1. 4-Bromoacetanilide (**7a**)

<u>^{1}H NMR spectrum of **7a**</u>

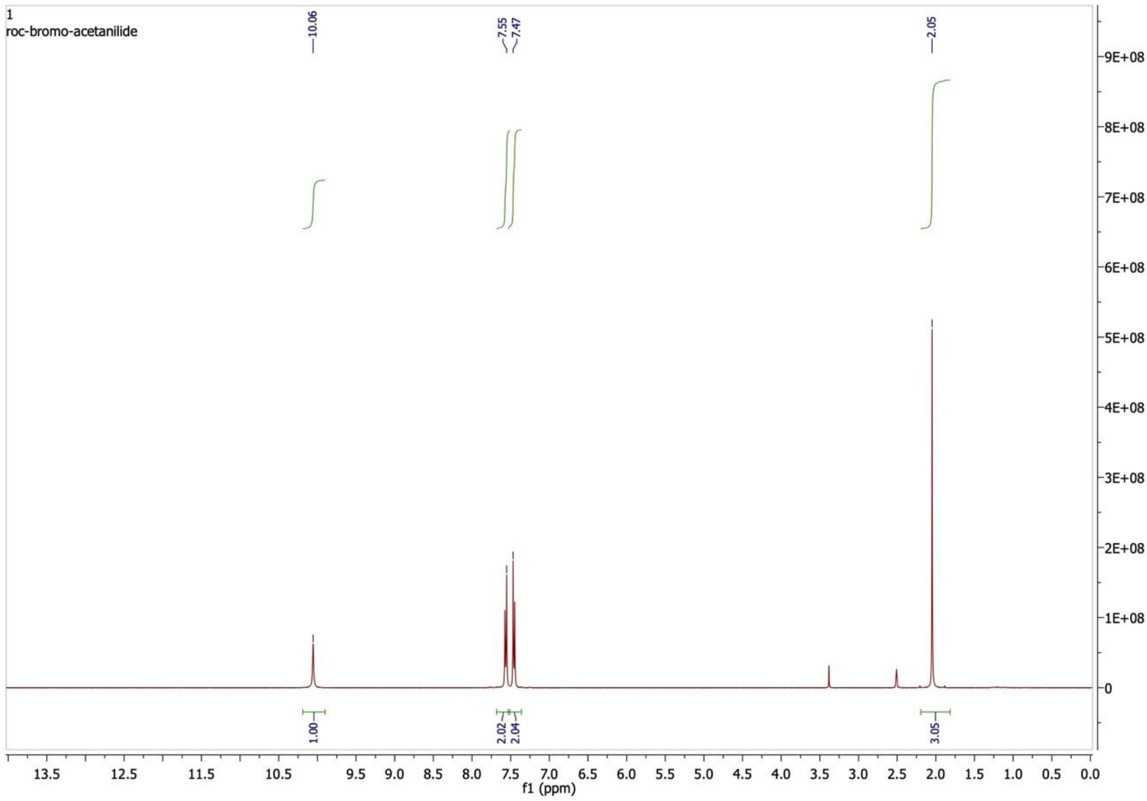

<u>^{13}C NMR spectrum of **7a**</u>

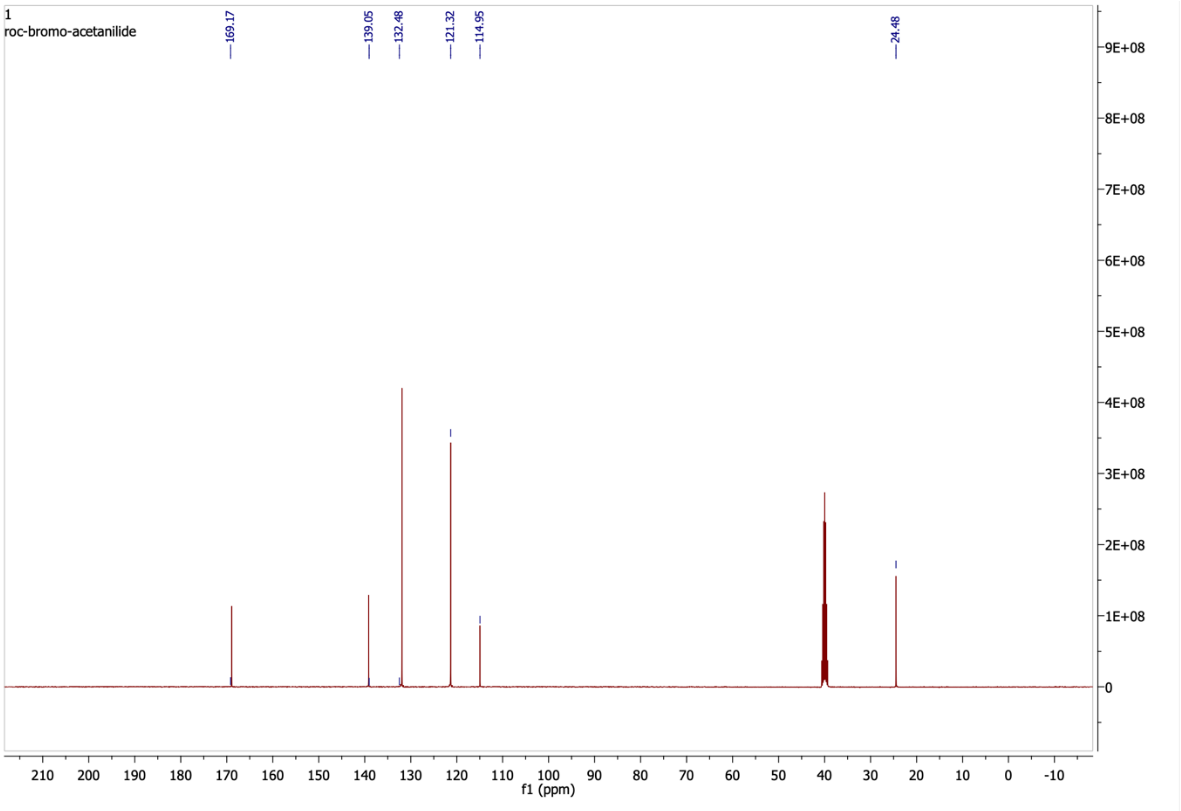

5.2. 4-Fluoroacetanilide (**7b**)

<u>^{1}H NMR spectrum of **7b**</u>

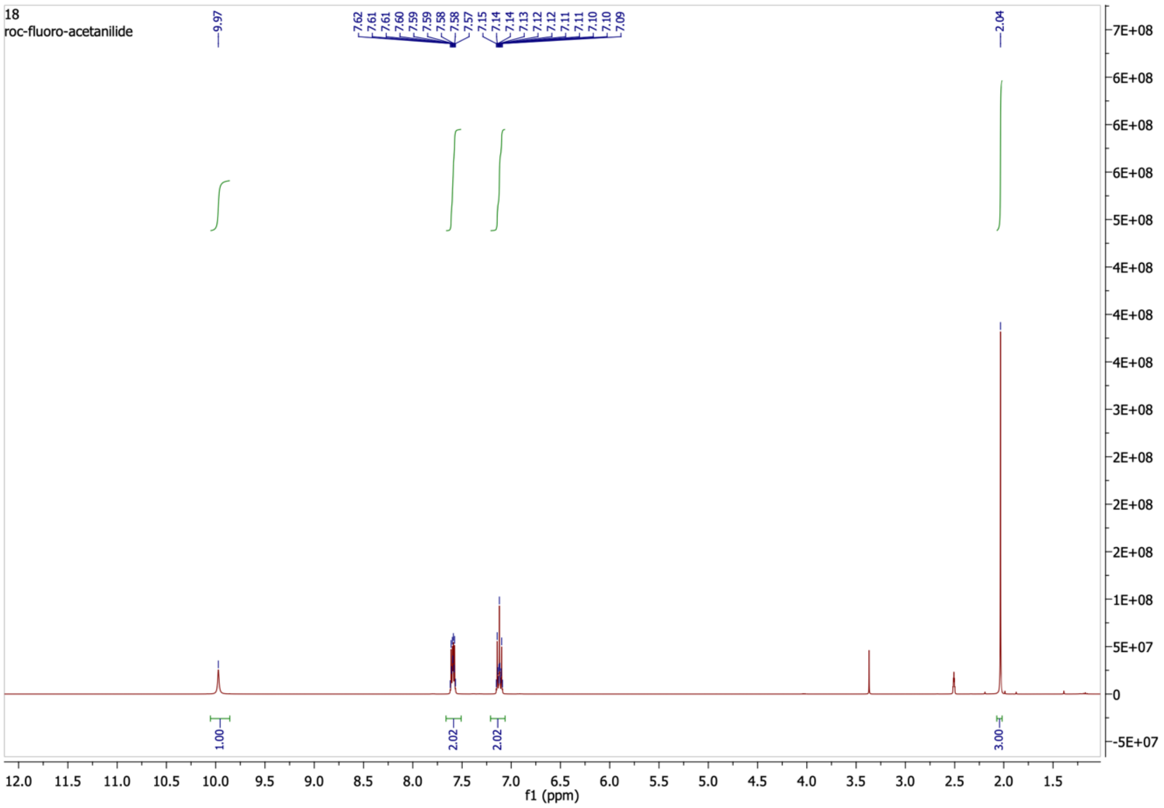

<u>^{13}C NMR spectrum of **7b**</u>

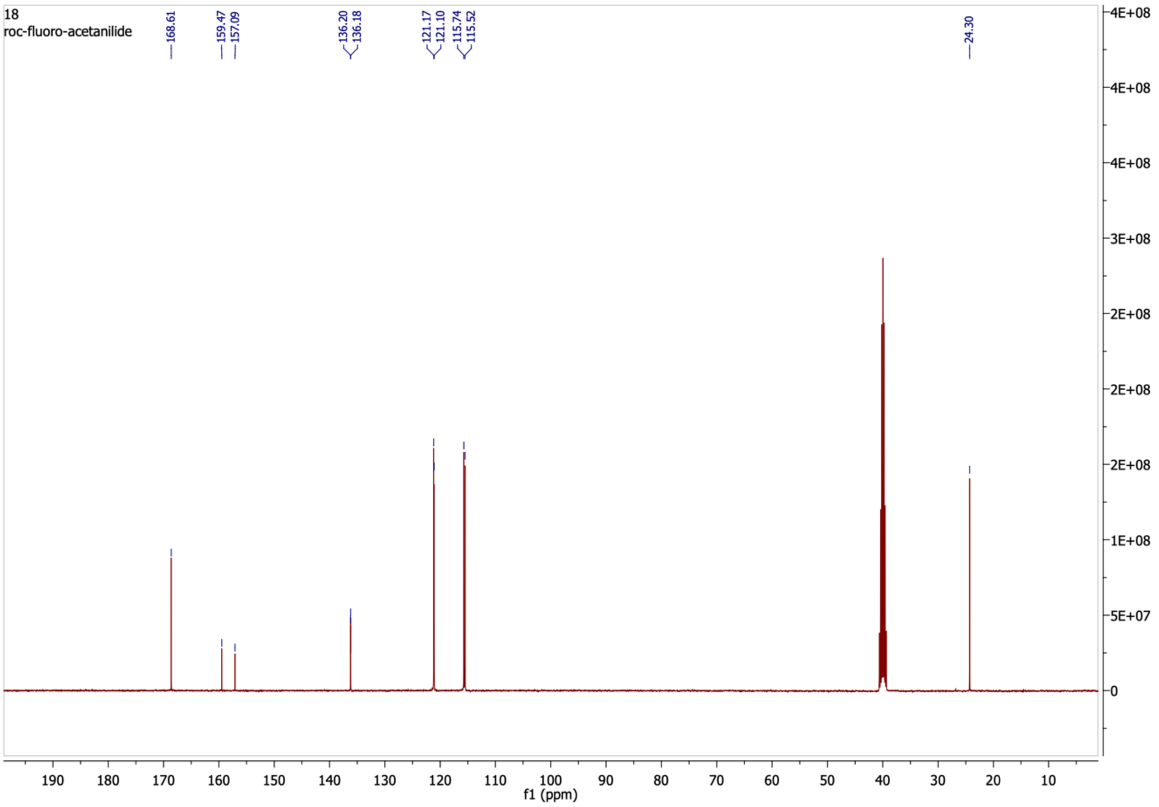

^{19}F NMR spectrum of **7b**

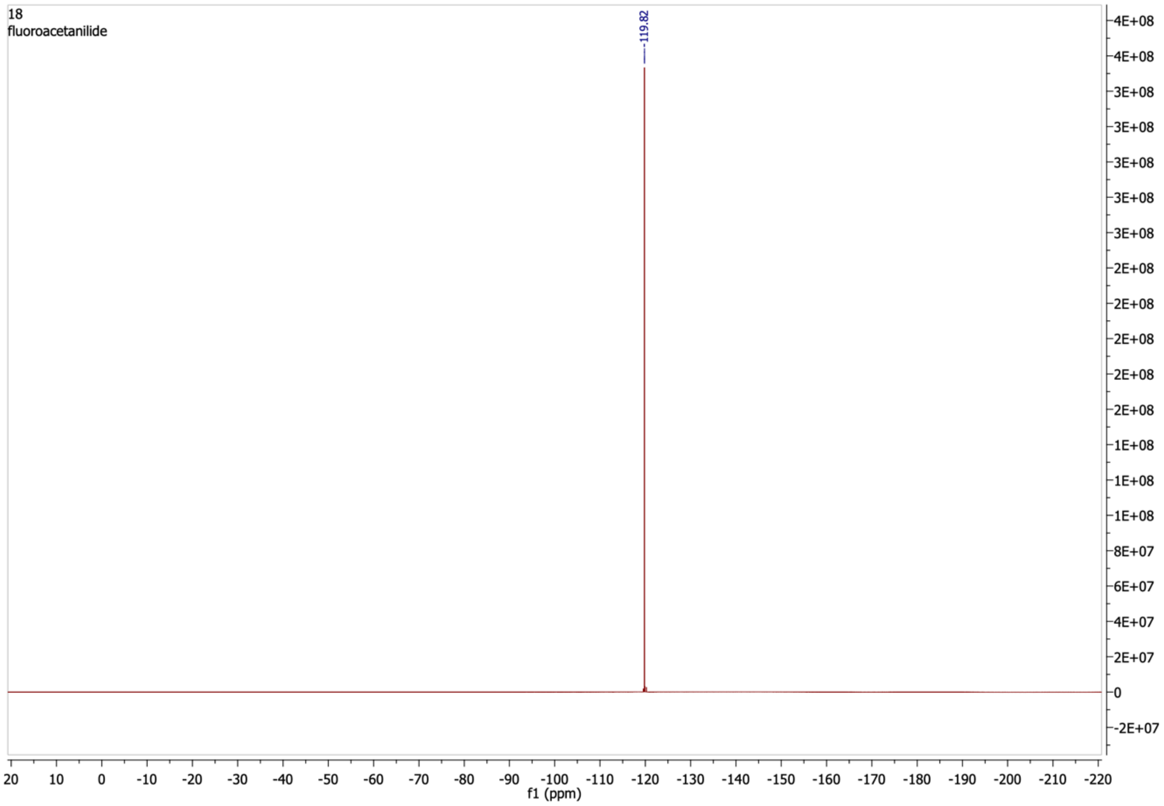

5.3. 6-Bromo-2-chloroquinoline-3-carbaldehyde (**10a**)

^{1}H NMR spectrum of **10a**

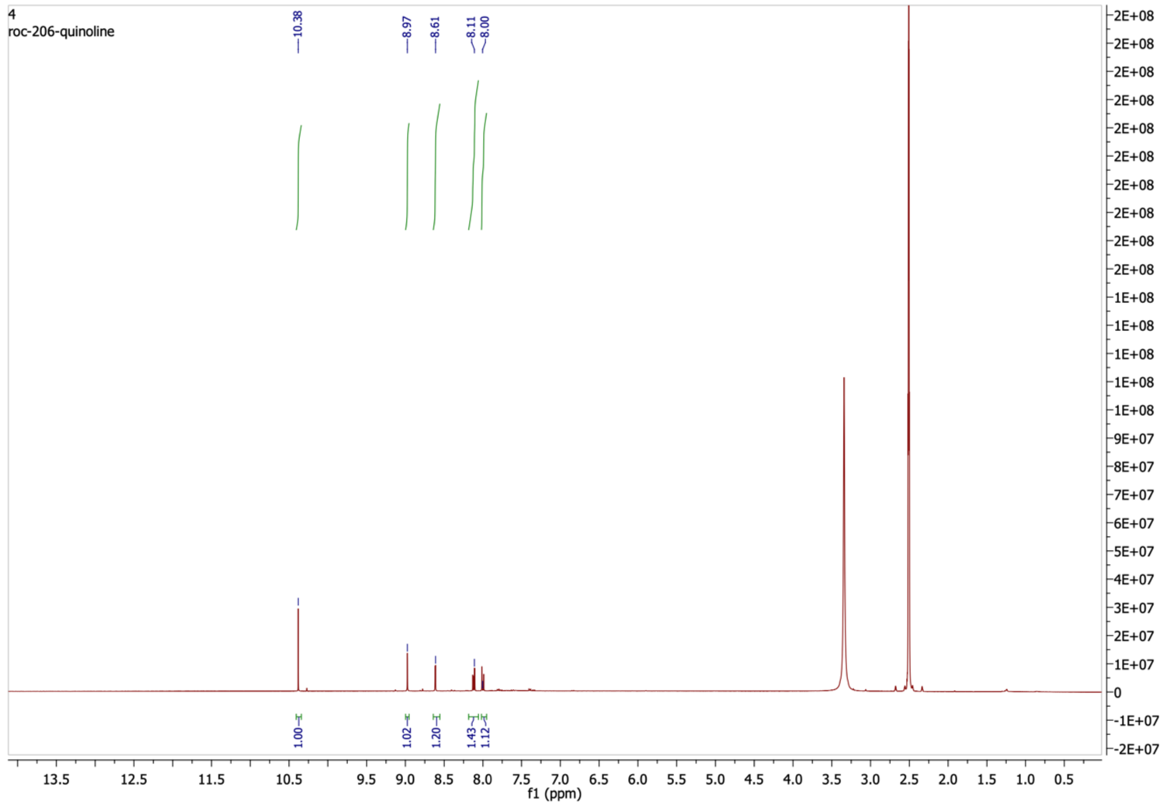

^{13}C NMR spectrum of **10a**

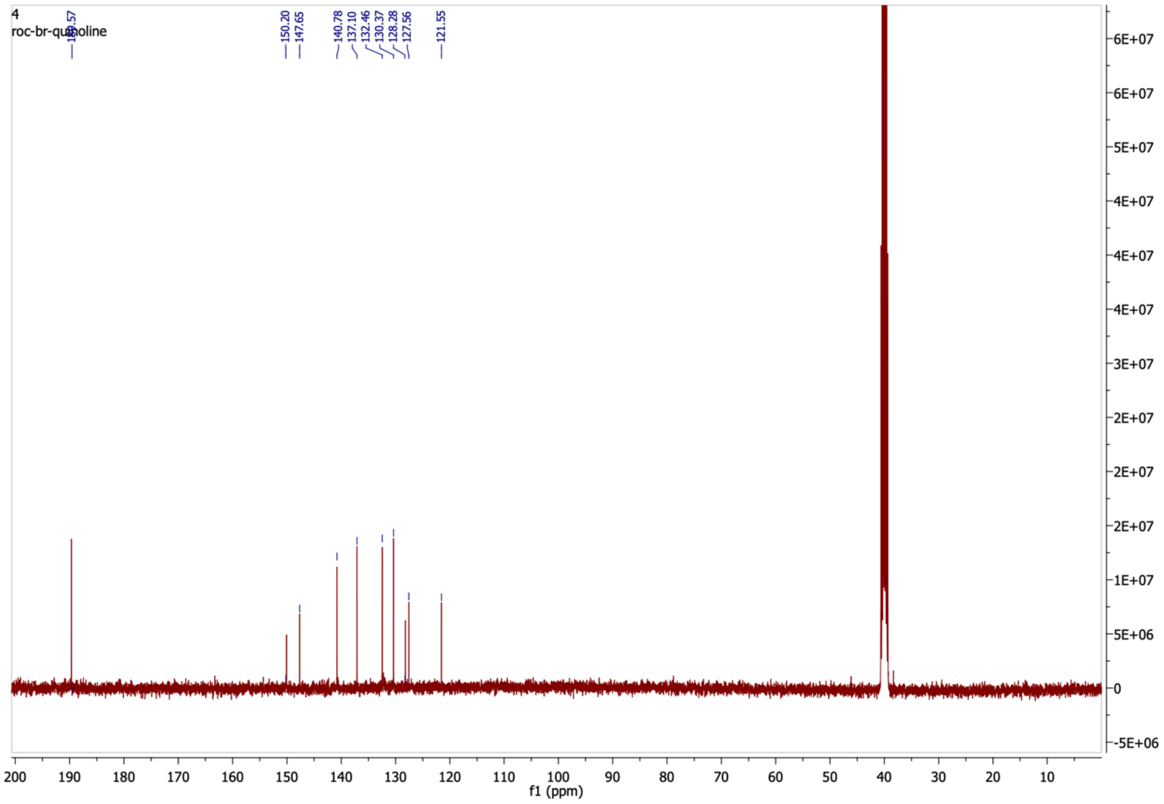

5.4. 2-Chloroquinoline-3-carbaldehyde (10c)

^{1}H NMR spectrum of **10c**

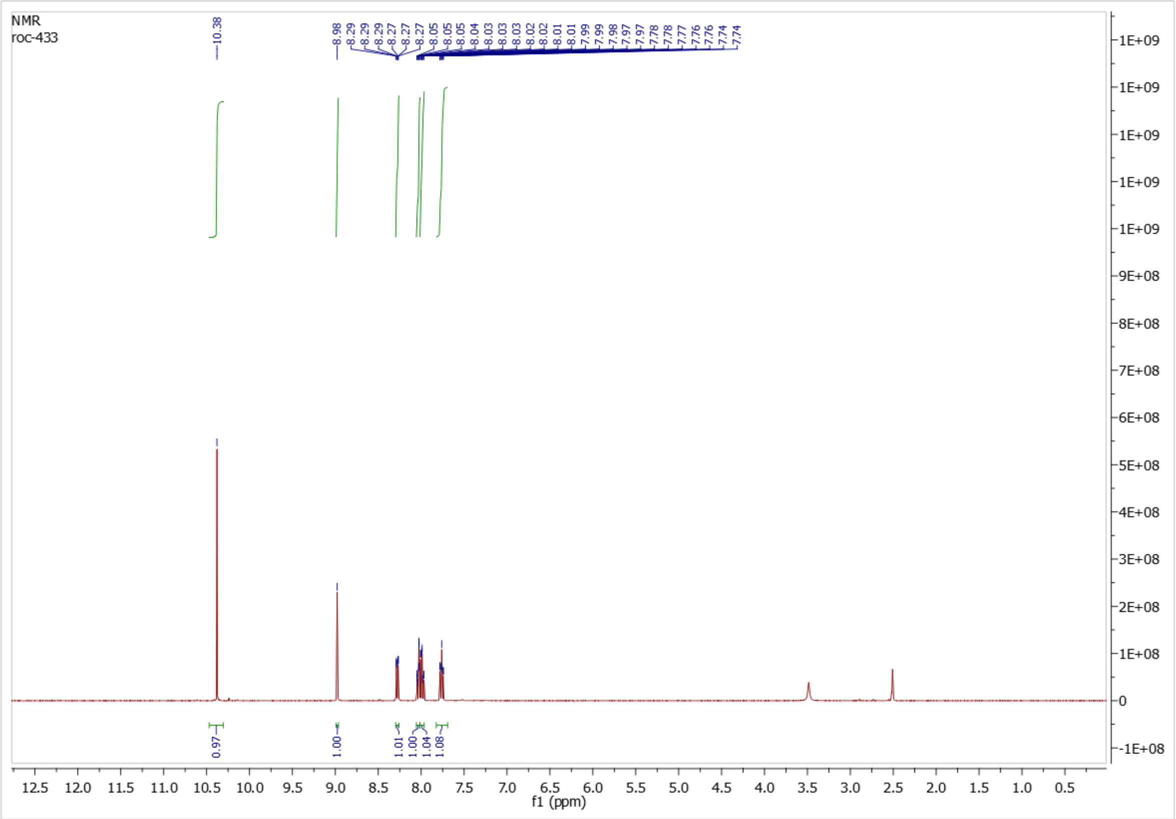

<u>^{13}C NMR spectrum of **10c**</u>

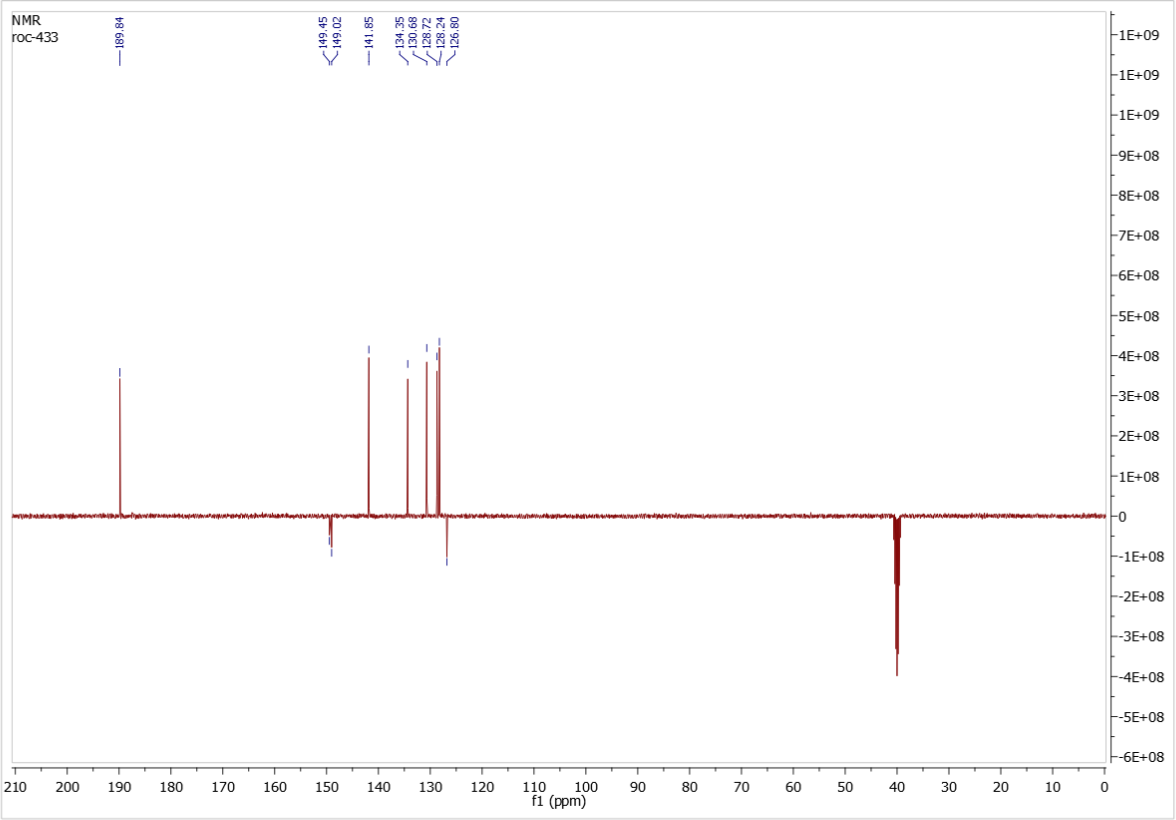

5.5. *N*-(4-Bromophenyl)-2-formyl-3-hydroxyacrylamide (**11a**)

<u>^{1}H NMR spectrum of **11a**</u>

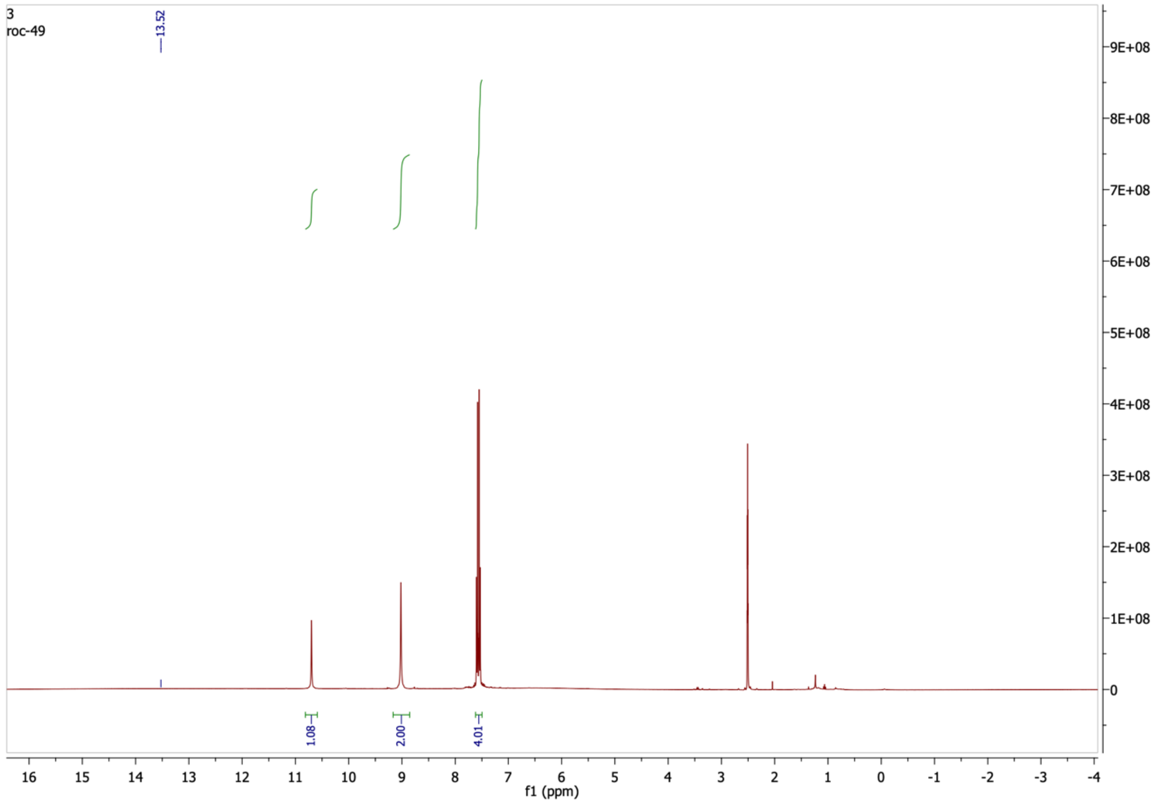

^{13}C NMR spectrum of **11a**

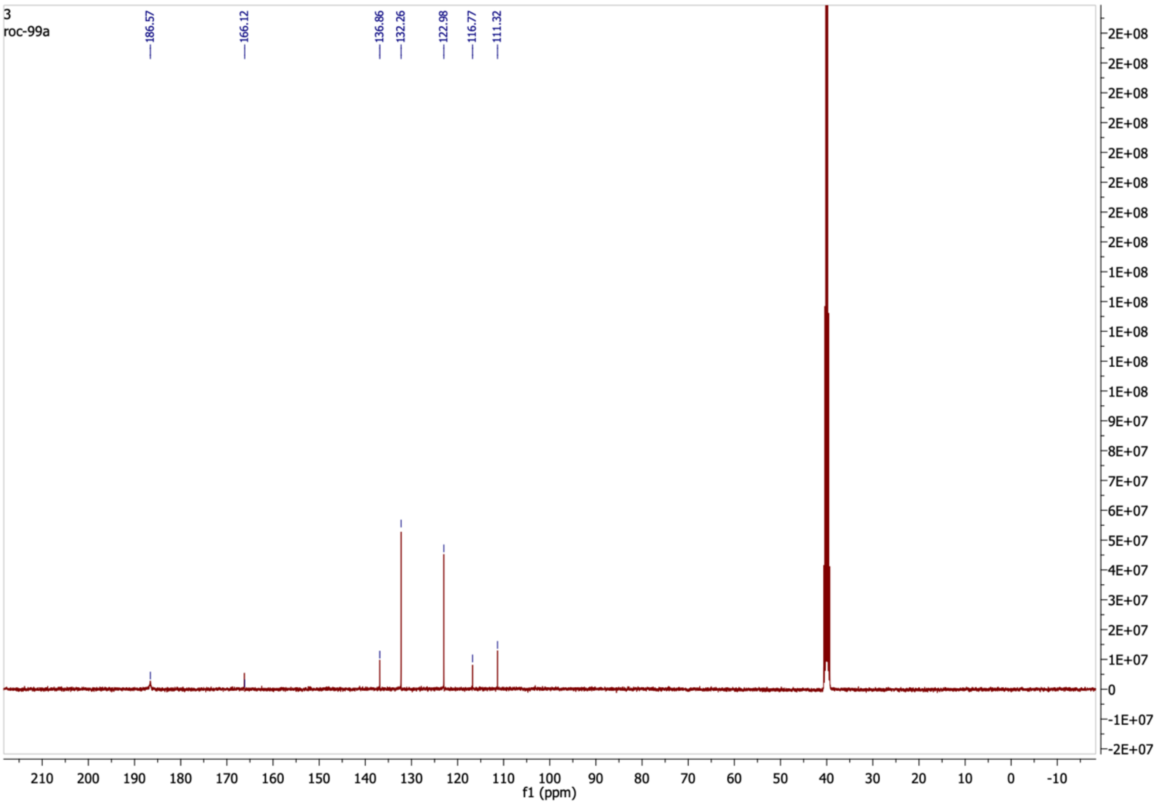

5.6. *N*-(4-Fluorophenyl)-2-formyl-3-hydroxyacrylamide (**11b**)

^{1}H NMR spectrum of **11b**

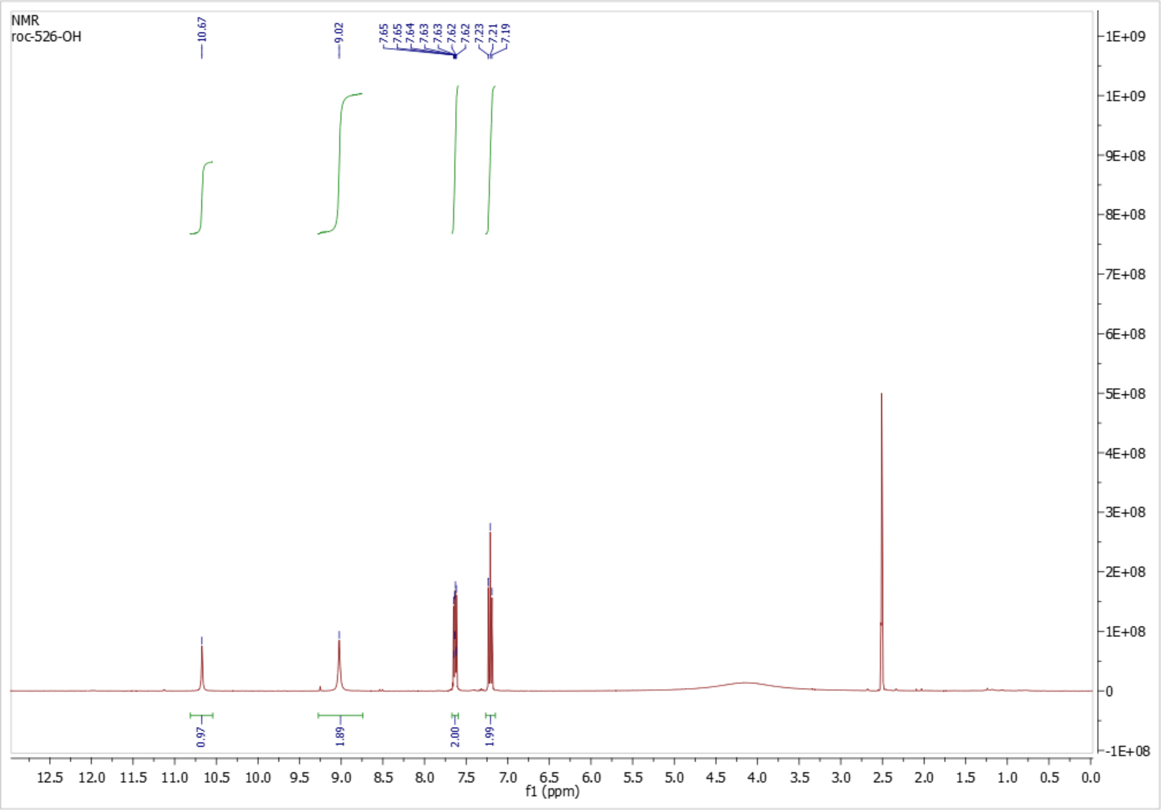

^{13}C NMR spectrum of **11b**

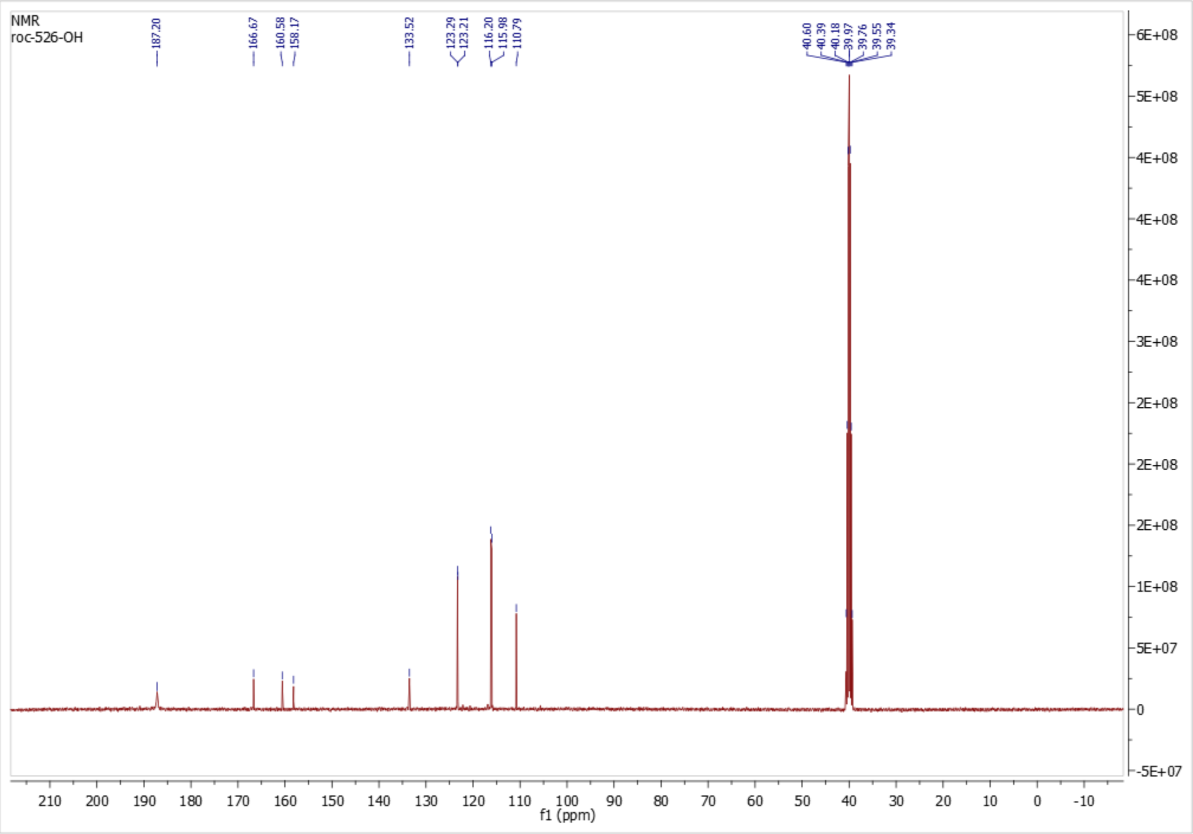

^{19}F NMR spectrum of **11b**

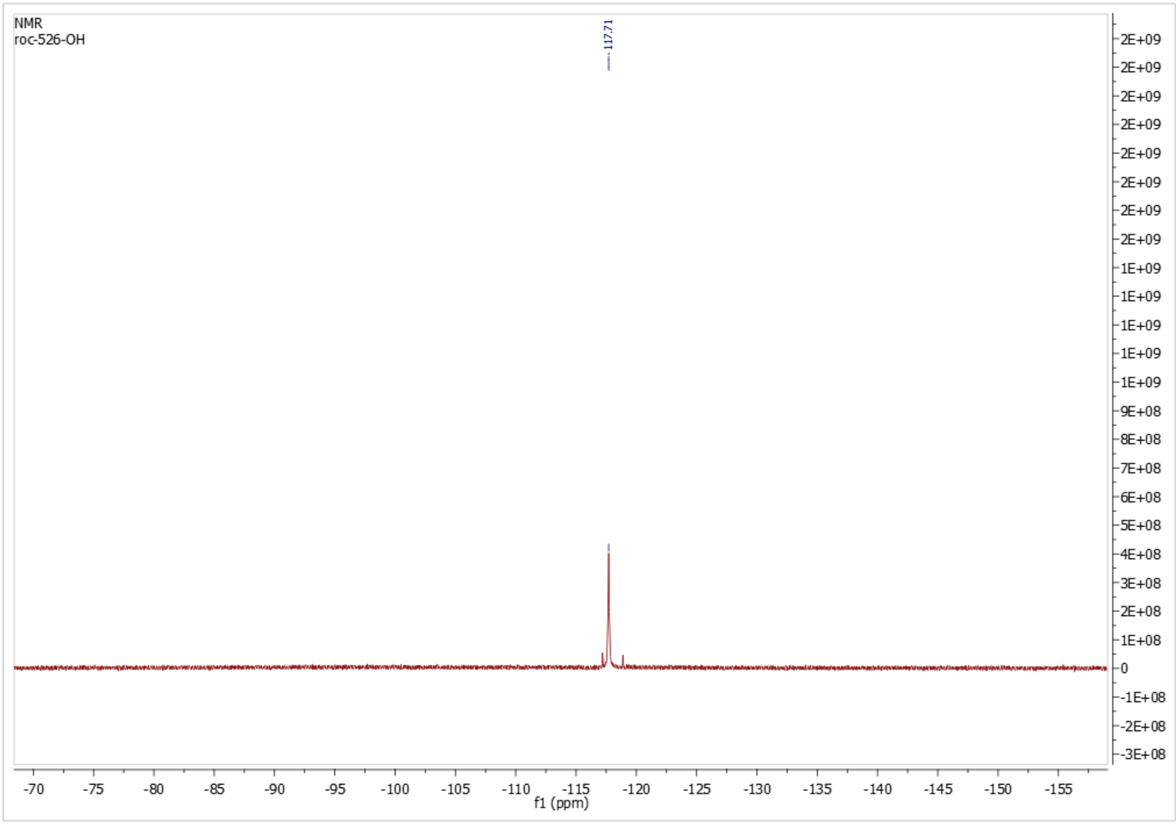

5.7. 6-Bromo-2-oxo-1,2-dihydroquinoline-3-carbaldehyde (**12a**)

^{1}H NMR spectrum of **12a**

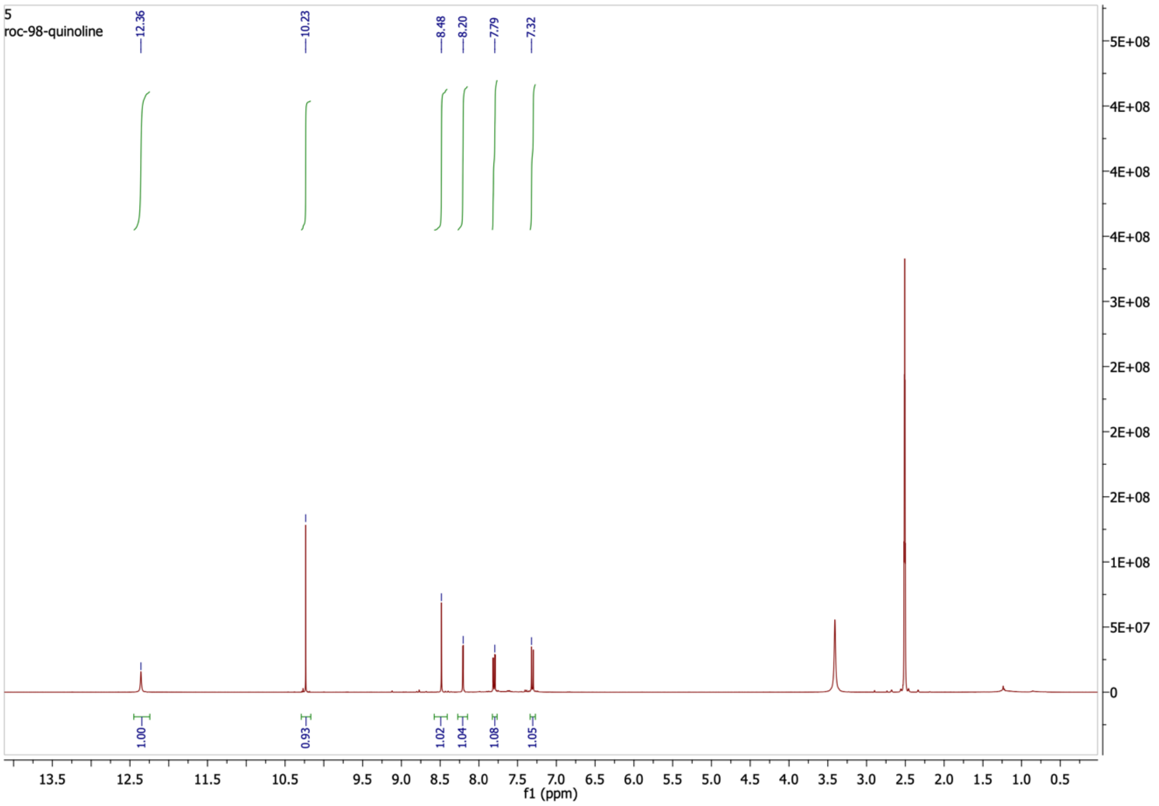

^{13}C NMR spectrum of **12a**

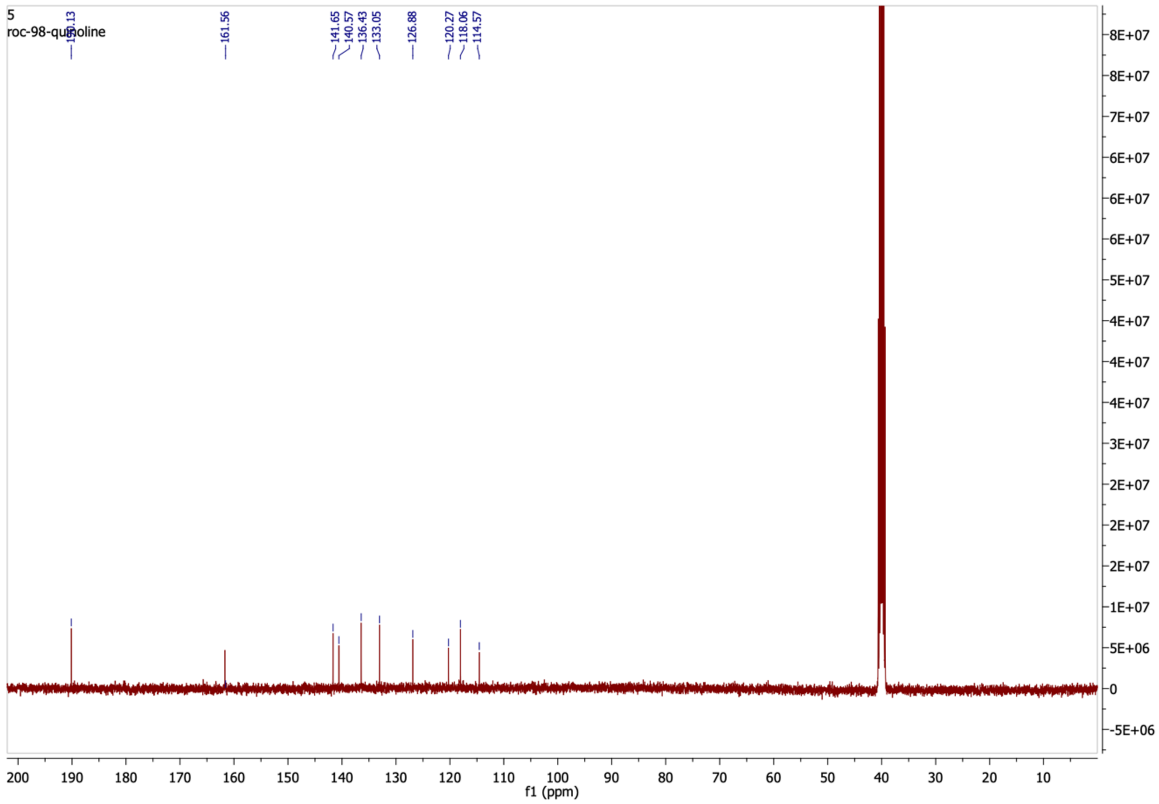

5.8. 6-Fluoro-2-oxo-1,2-dihydroquinoline-3-carbaldehyde (**12b**)

^{1}H NMR spectrum of **12b**

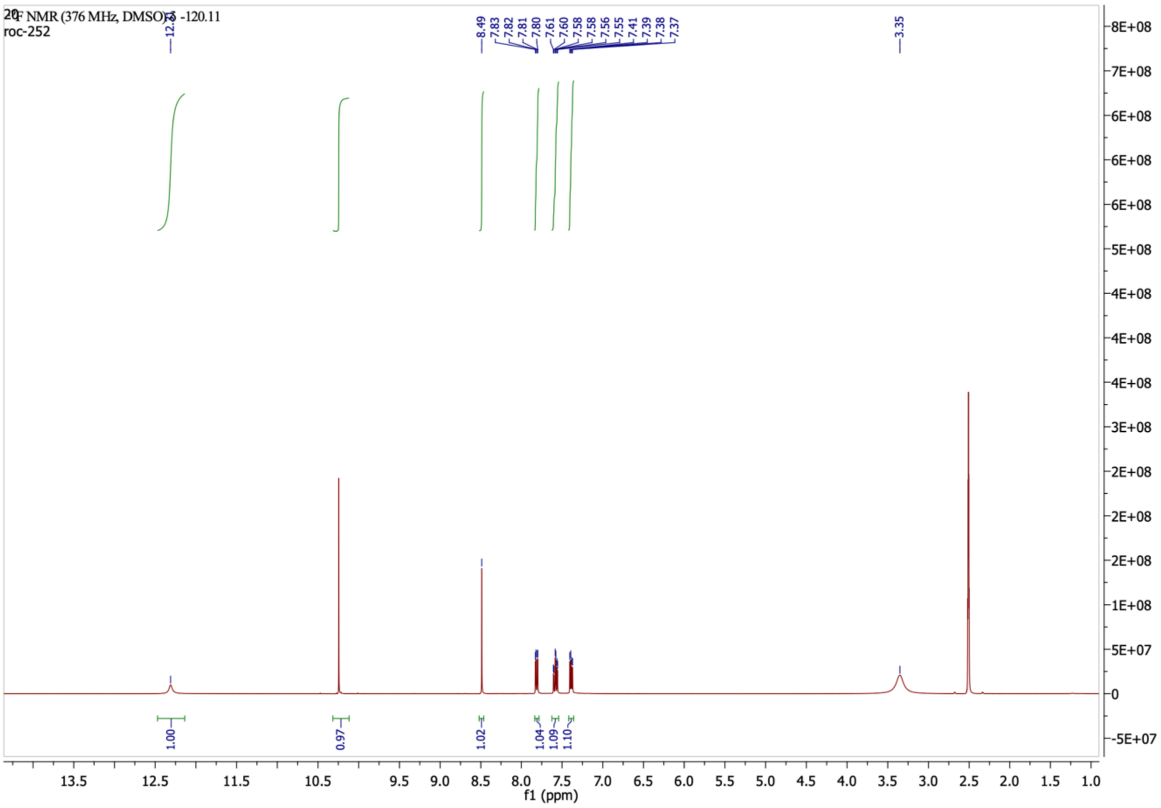

^{13}C NMR spectrum of **12b**

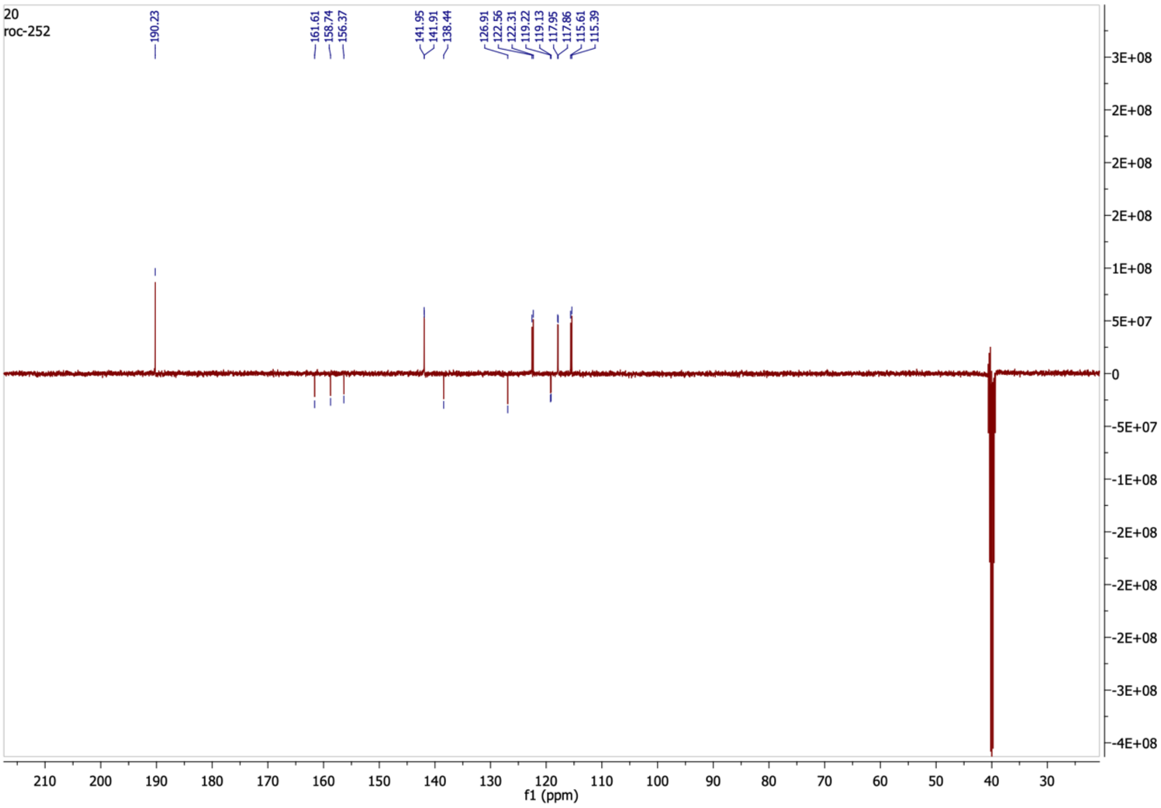

^{19}F NMR spectrum of **12b**

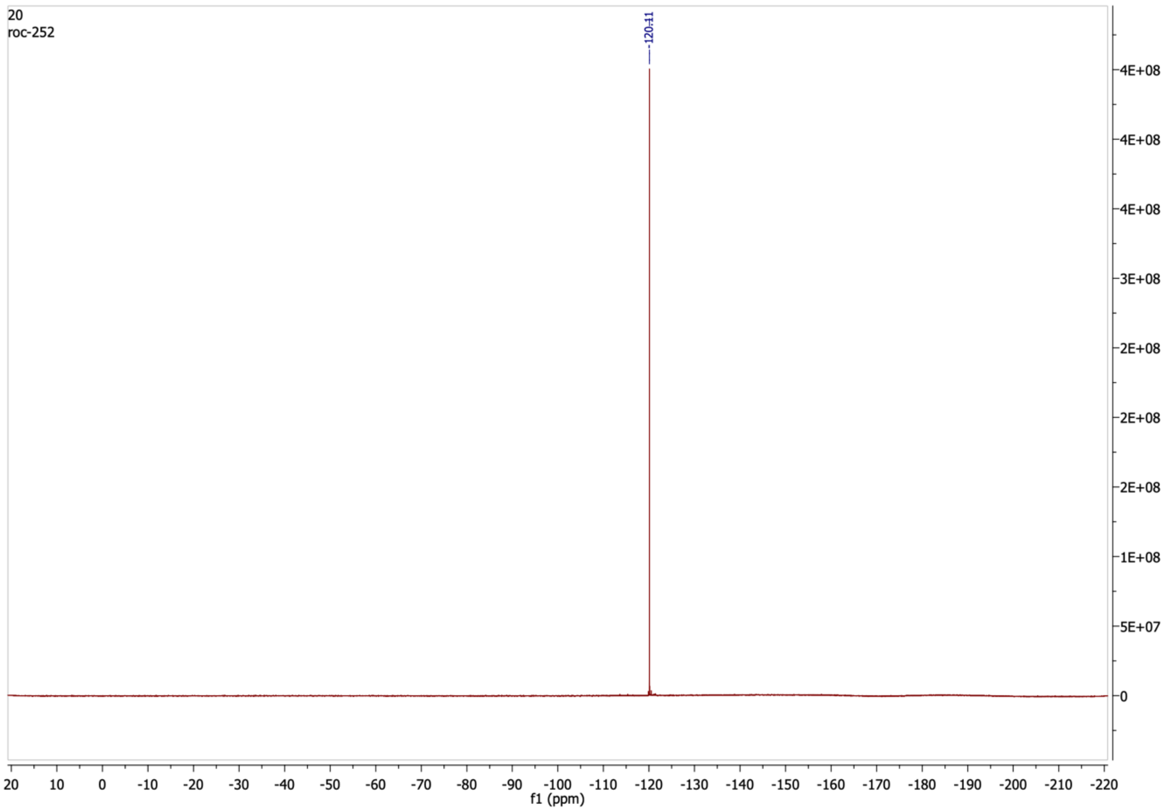

5.9. 2-Oxo-1,2-dihydroquinoline-3-carbaldehyde (**12c**)

^{1}H NMR spectrum of **12c**

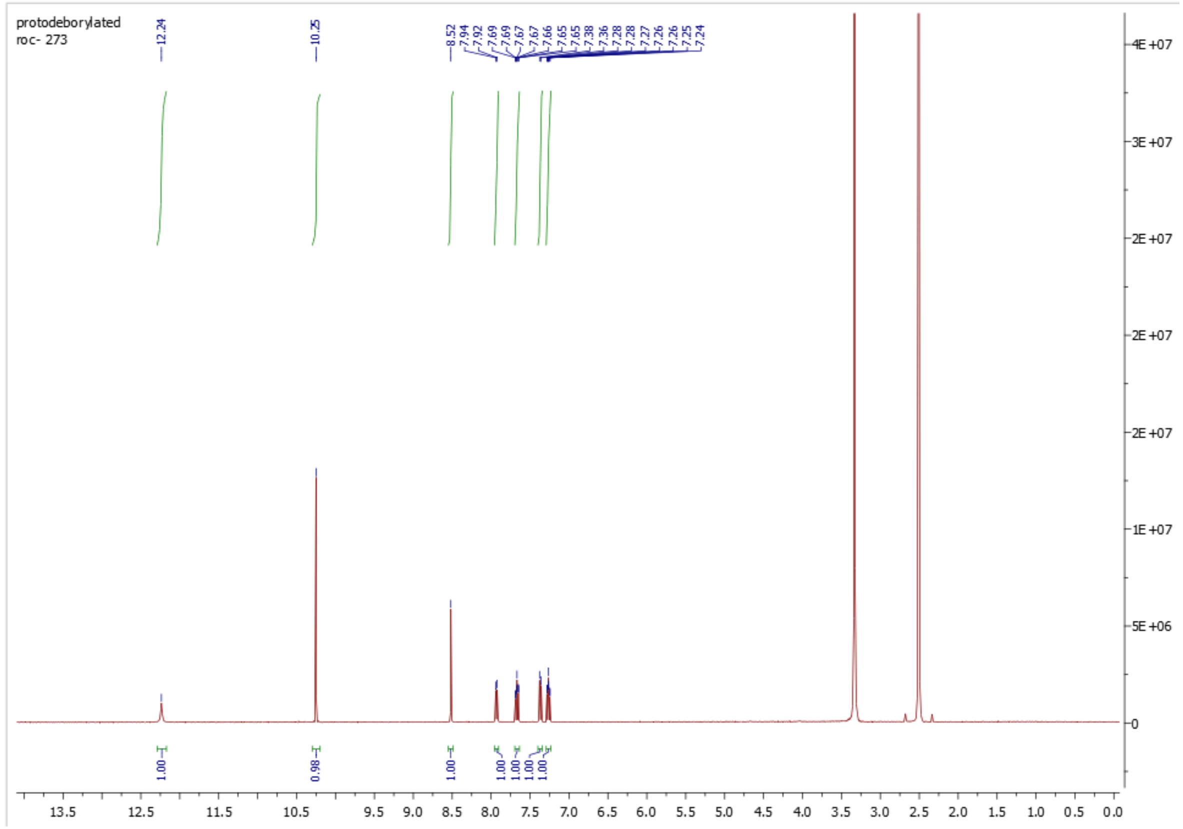

^{13}C NMR spectrum of **12c**

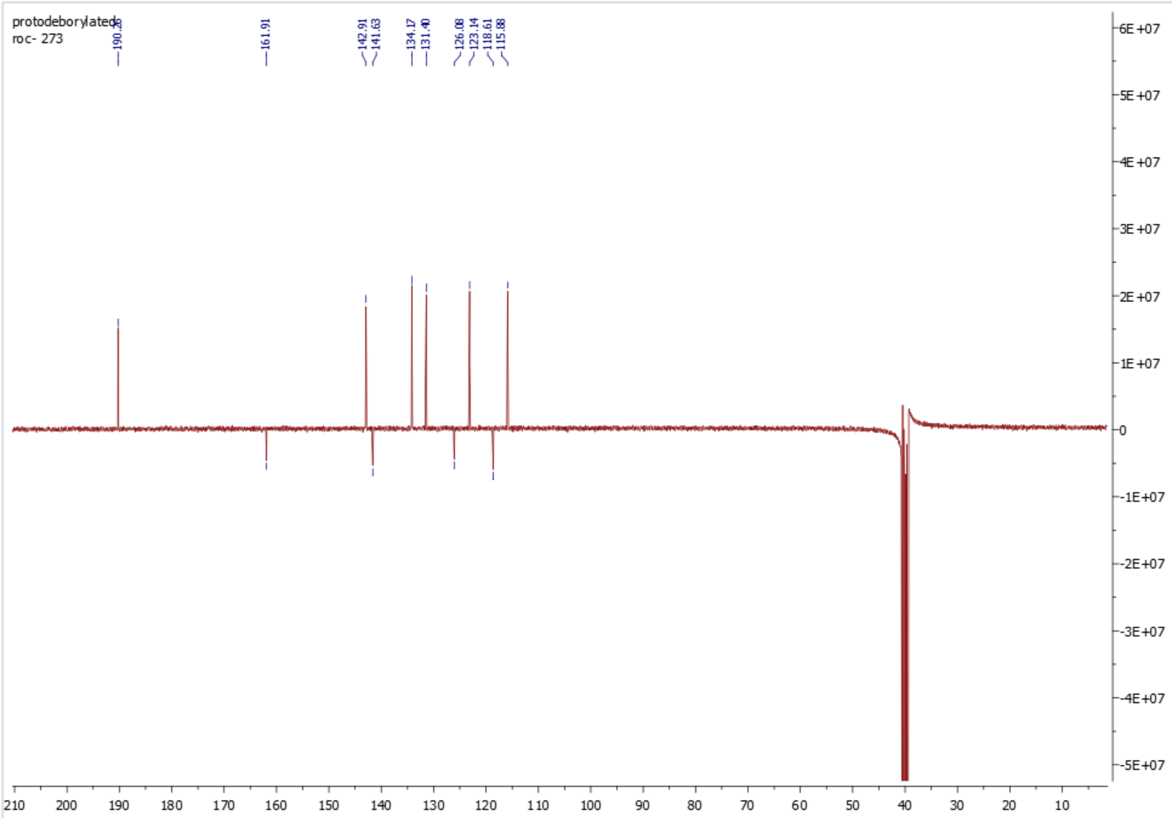

5.10. 4-{[(6-Bromo-2-oxo-1,2-dihydroquinolin-3-yl)methyl]amino}-2-methoxybenzonitrile (**13a**)

^{1}H NMR spectrum of **13a**

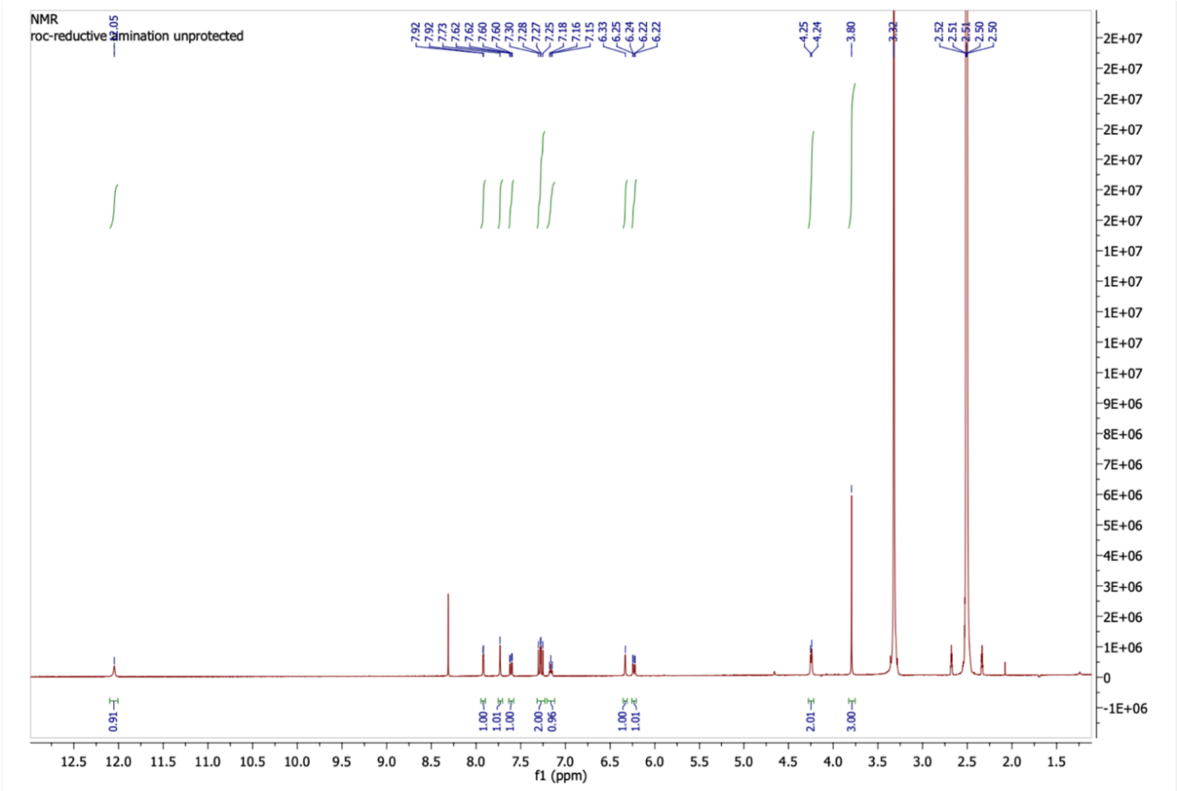

^{13}C NMR spectrum of **13a**

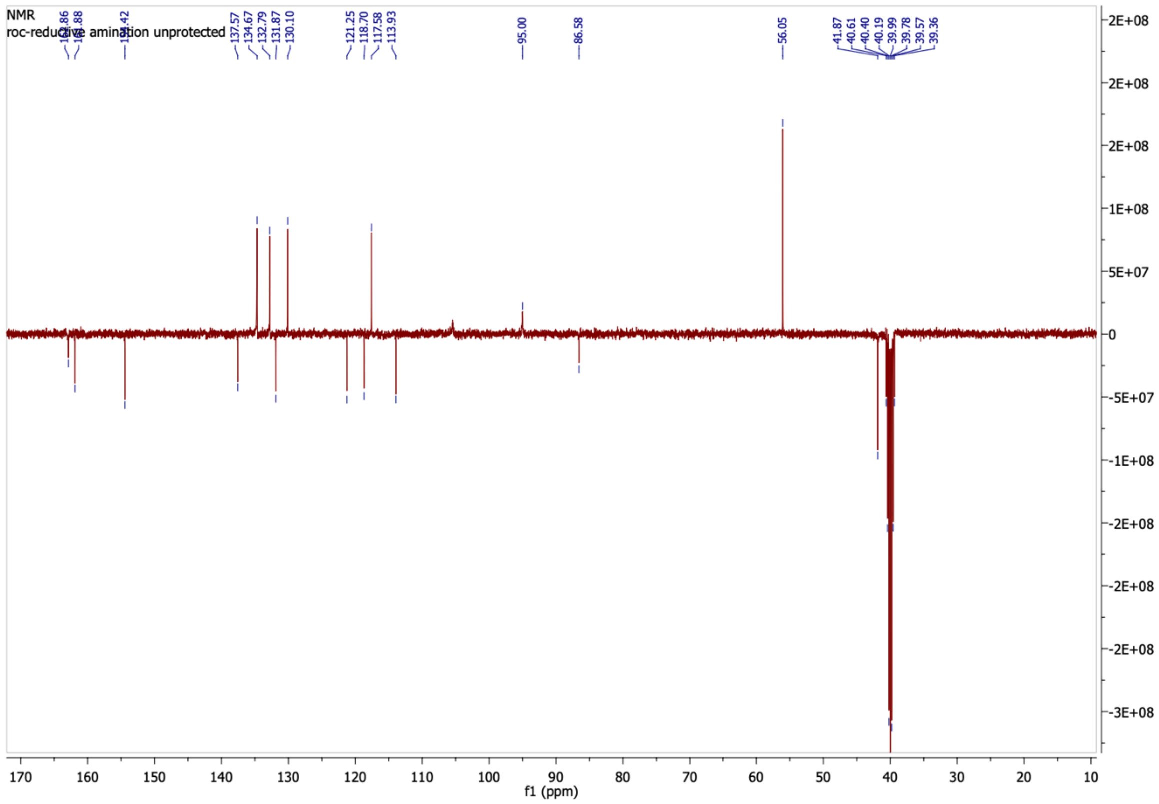

HSQC NMR spectrum of **13a**

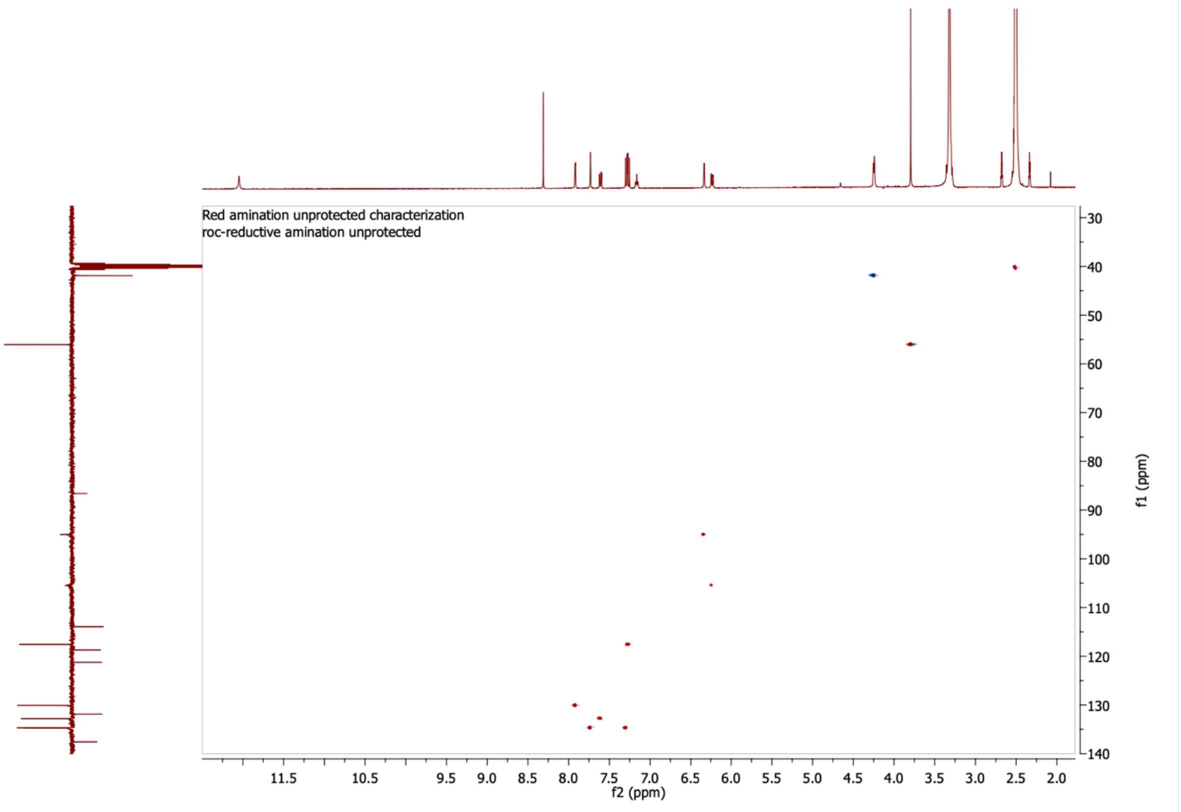

5.11. 4-{[(6-Fluoro-2-oxo-1,2-dihydroquinolin-3-yl)methyl]amino}-2-methoxybenzonitrile (**4**)

^{1}H NMR spectrum of **4**

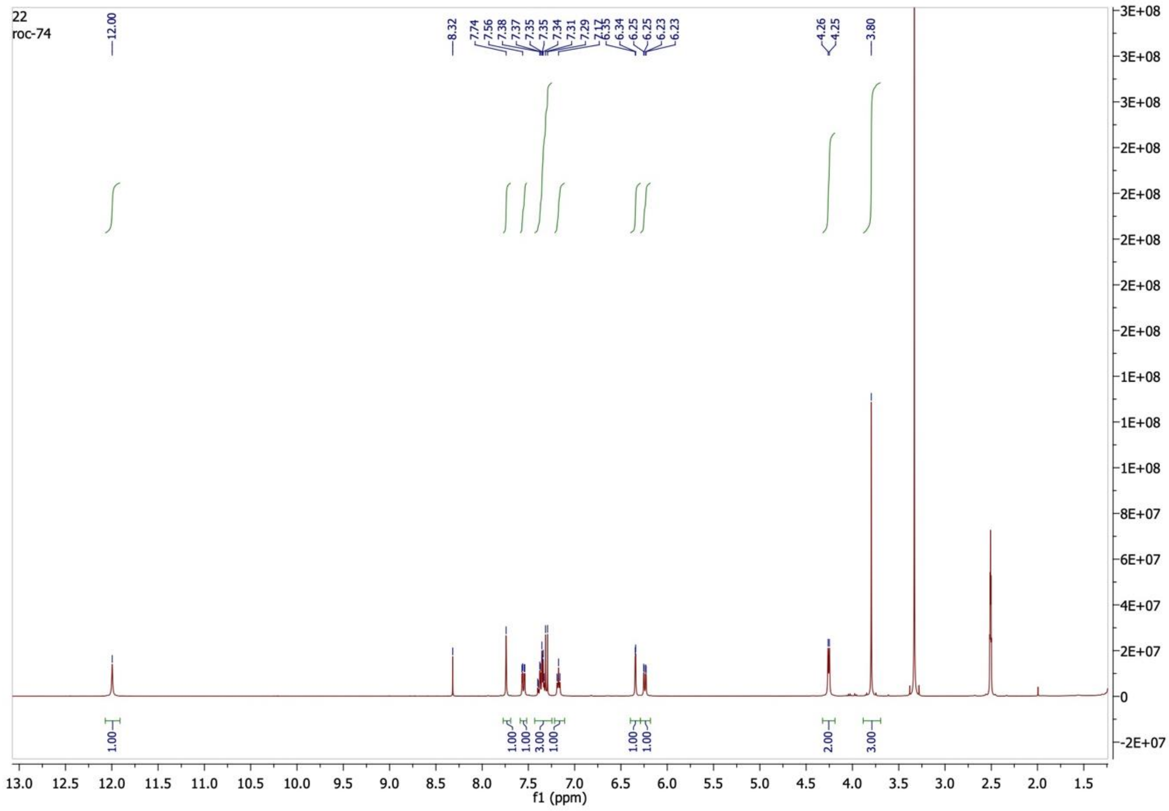

^{13}C NMR spectrum of **4**

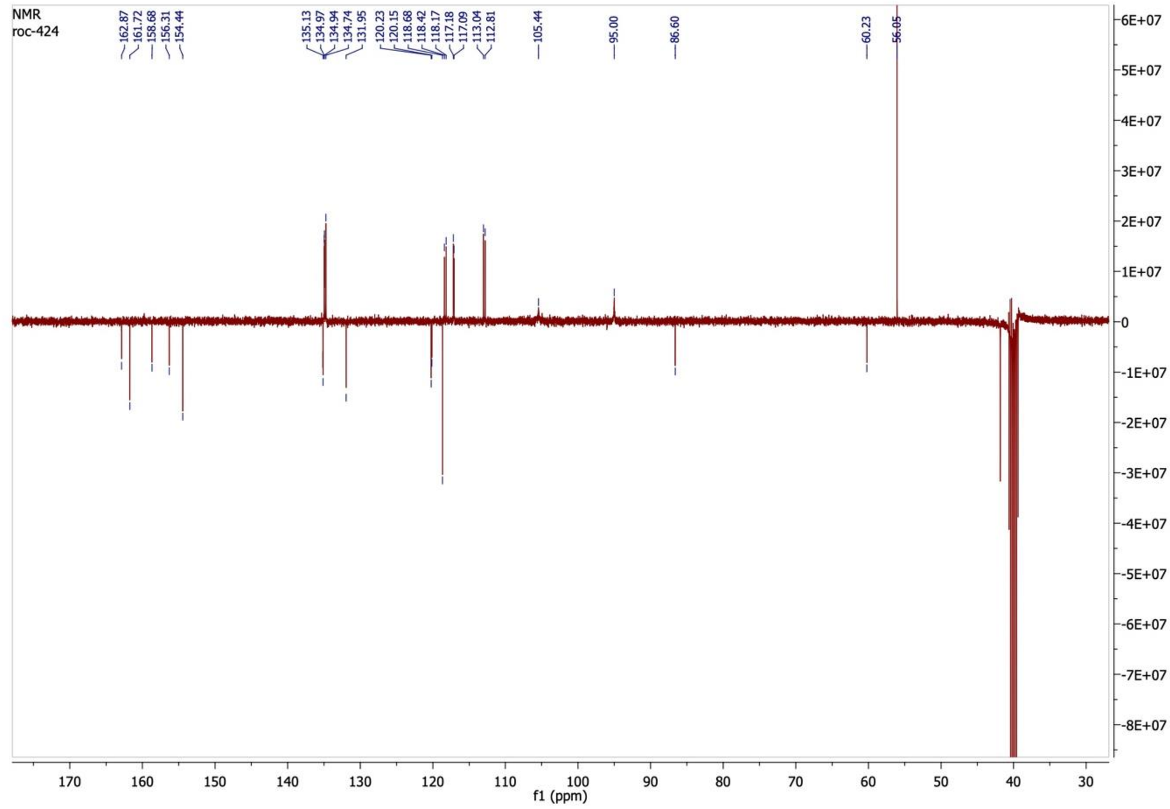

^{19}F NMR spectrum of **4**

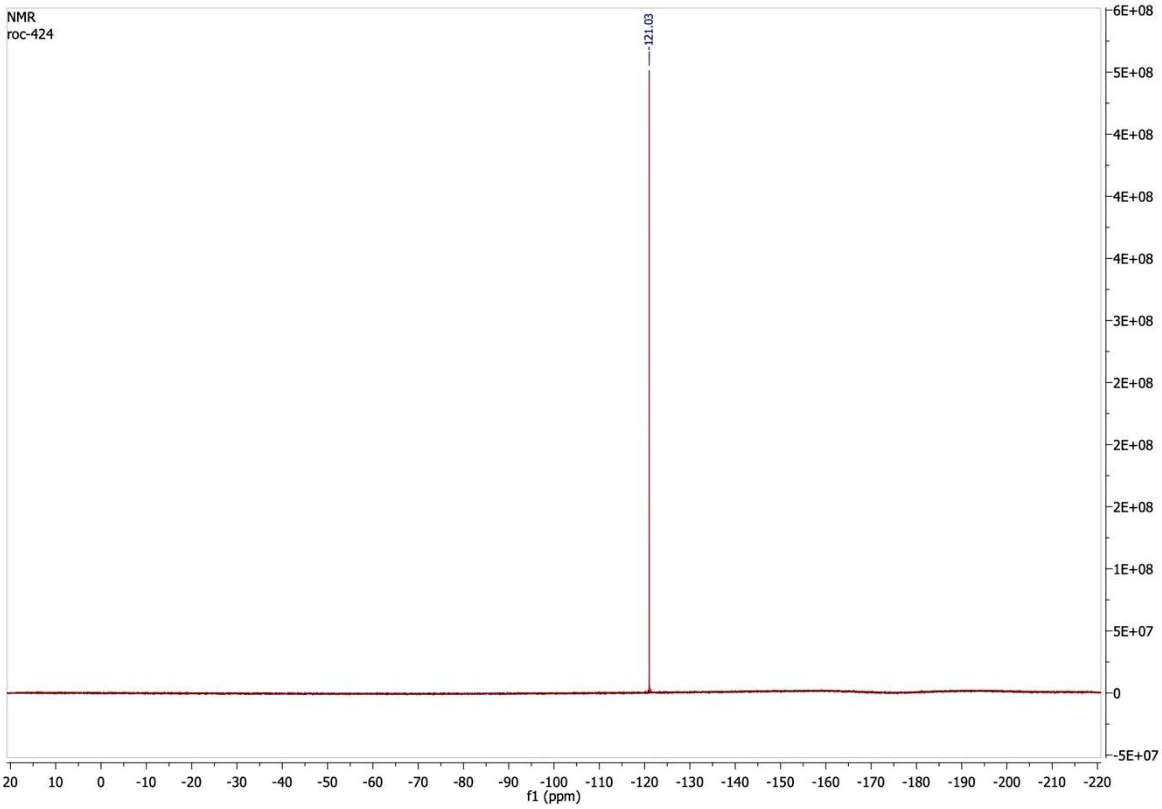

5.12. 2-Methoxy-4-(((2-oxo-1,2-dihydroquinolin-3-yl)methyl)amino)benzonitrile (**13c**)

^{1}H NMR spectrum of **13c**

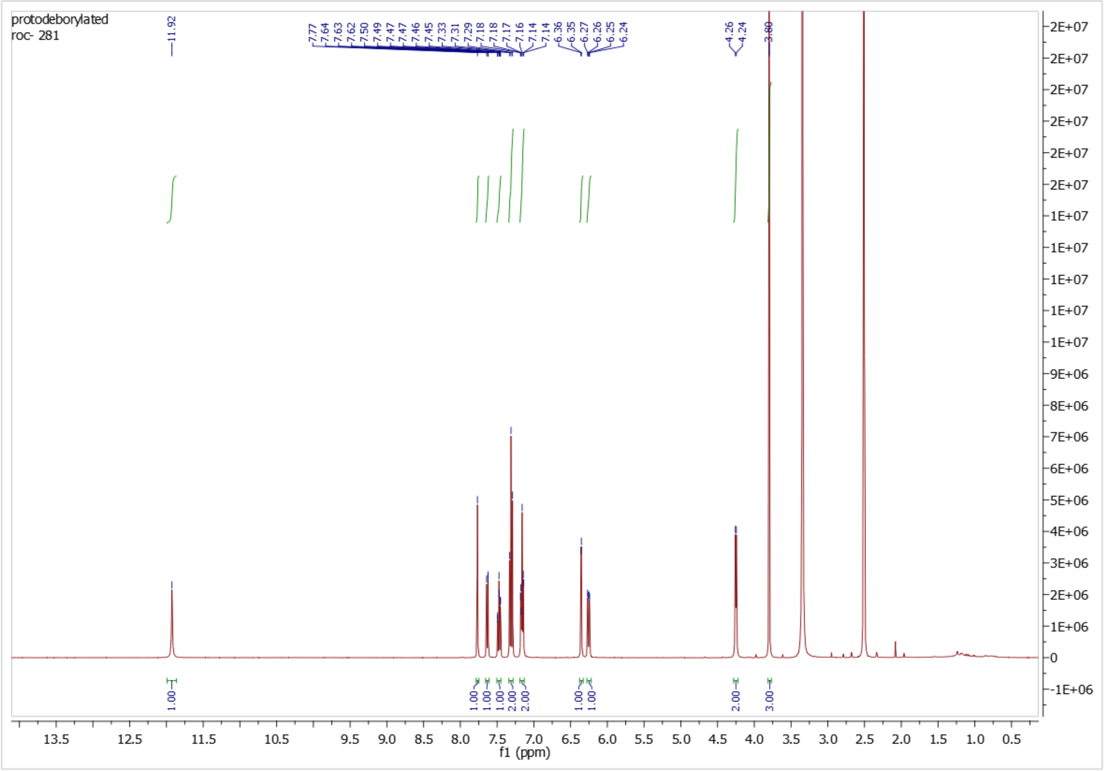

^{13}C NMR spectrum of **13c**

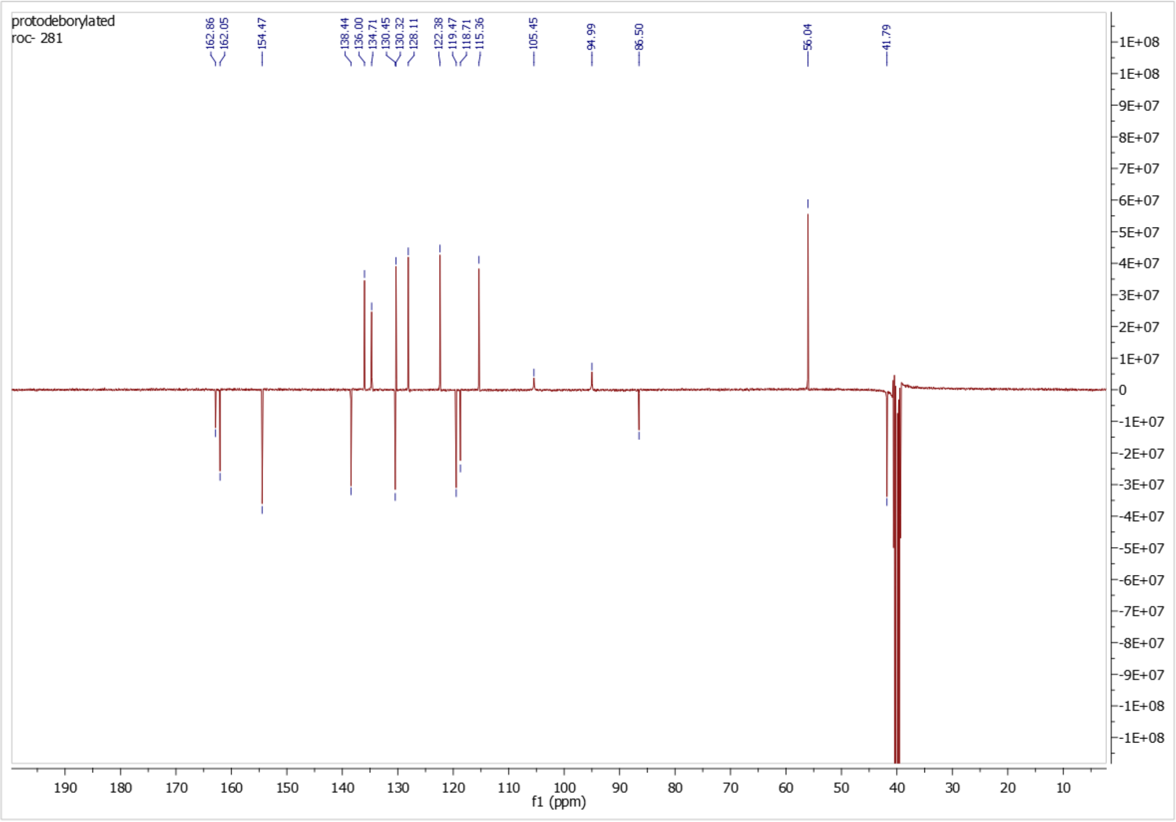

5.13. 2-Methoxy-4-{[(2-oxo-6-(4,4,5,5-tetramethyl-1,3,2-dioxaborolan-2-yl)-1,2-dihydroquinolin-3-yl)methyl]amino}benzonitrile (**14**)

^{1}H NMR spectrum of **14**

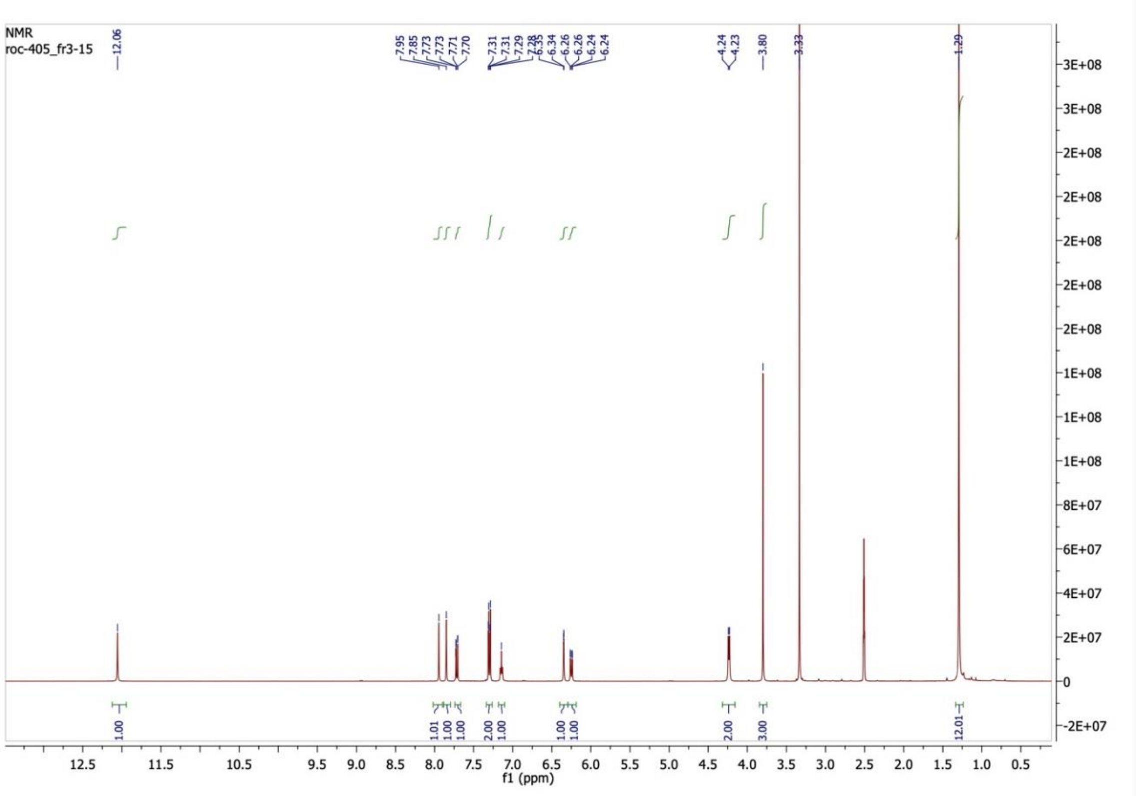

^{13}C NMR spectrum of **14**

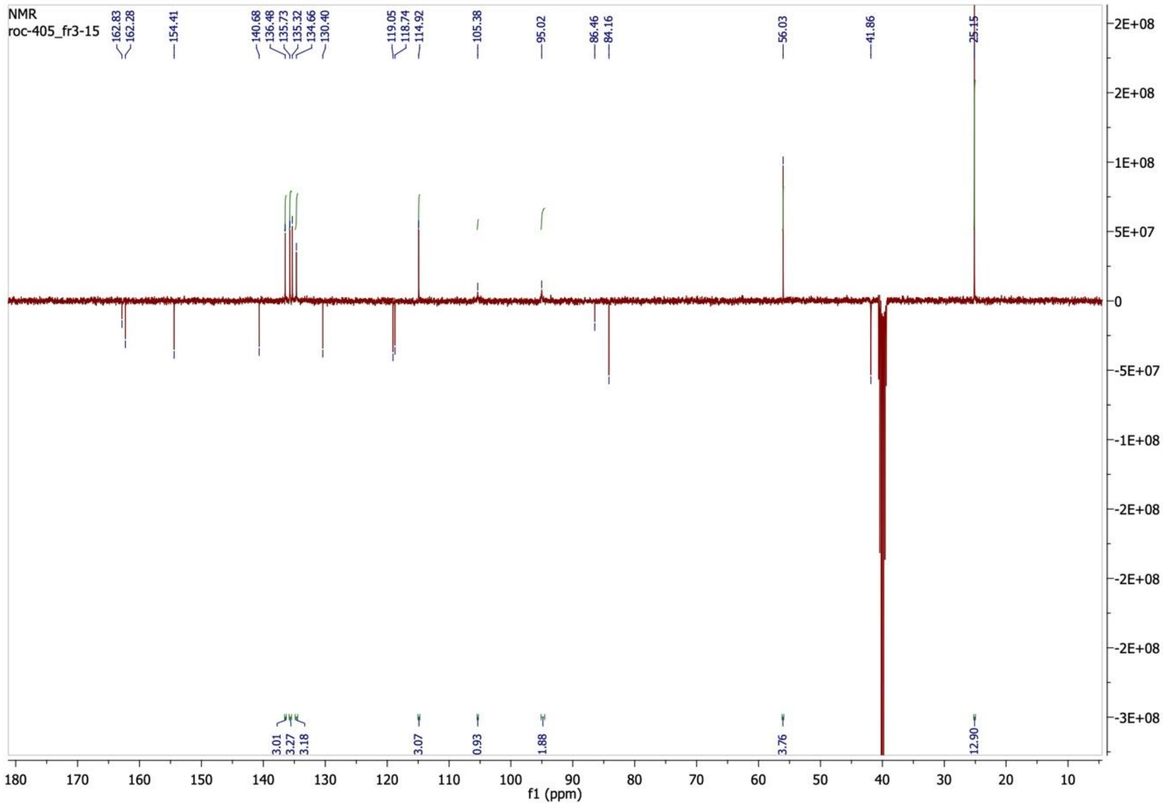

HSQC NMR spectrum of **14**

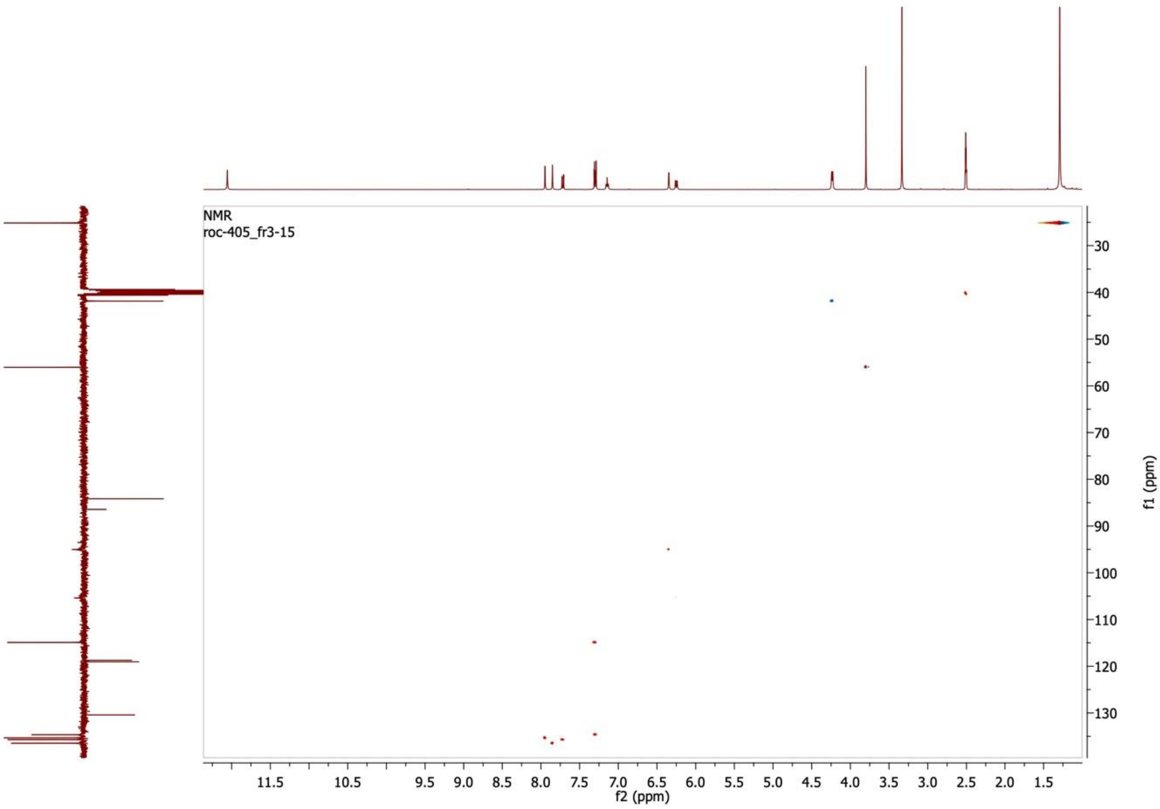

5.14. 4-Amino-2-methoxybenzonitrile (**16**)

^{1}H NMR spectrum of **16**

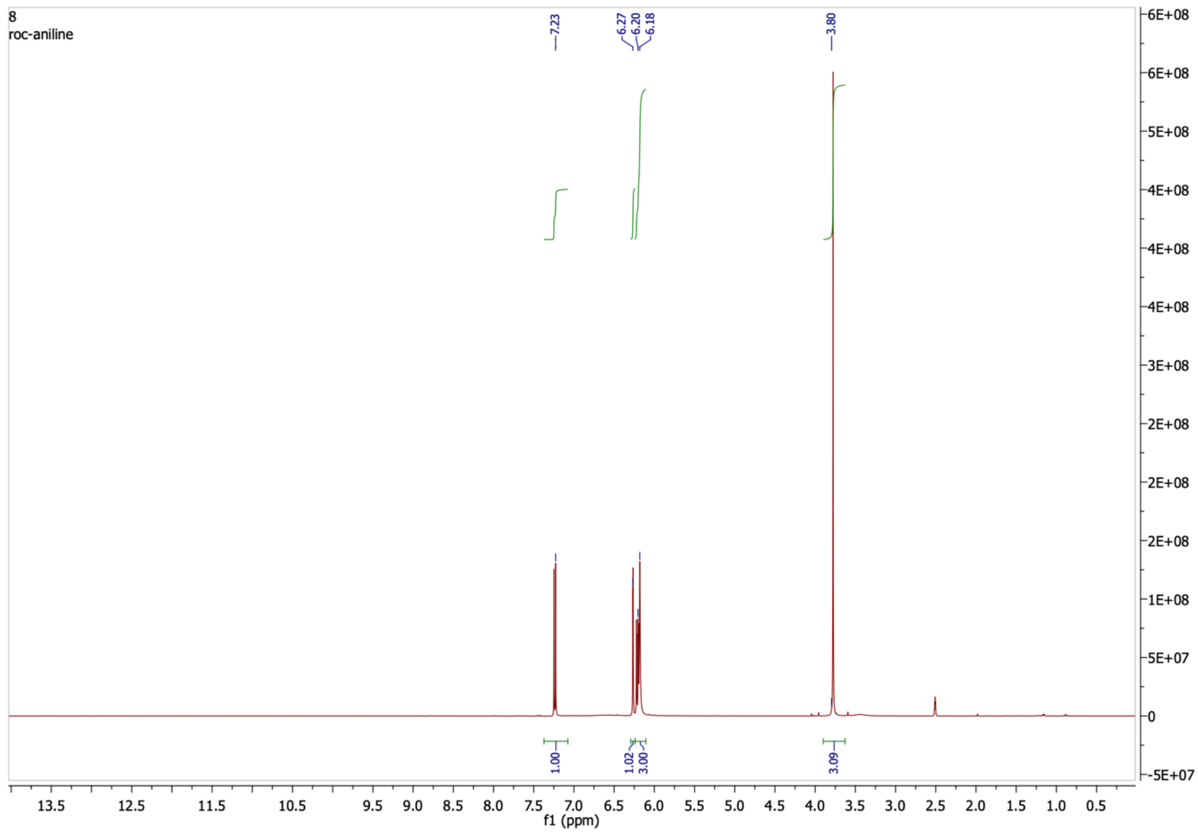

^{13}C NMR spectrum of **16**

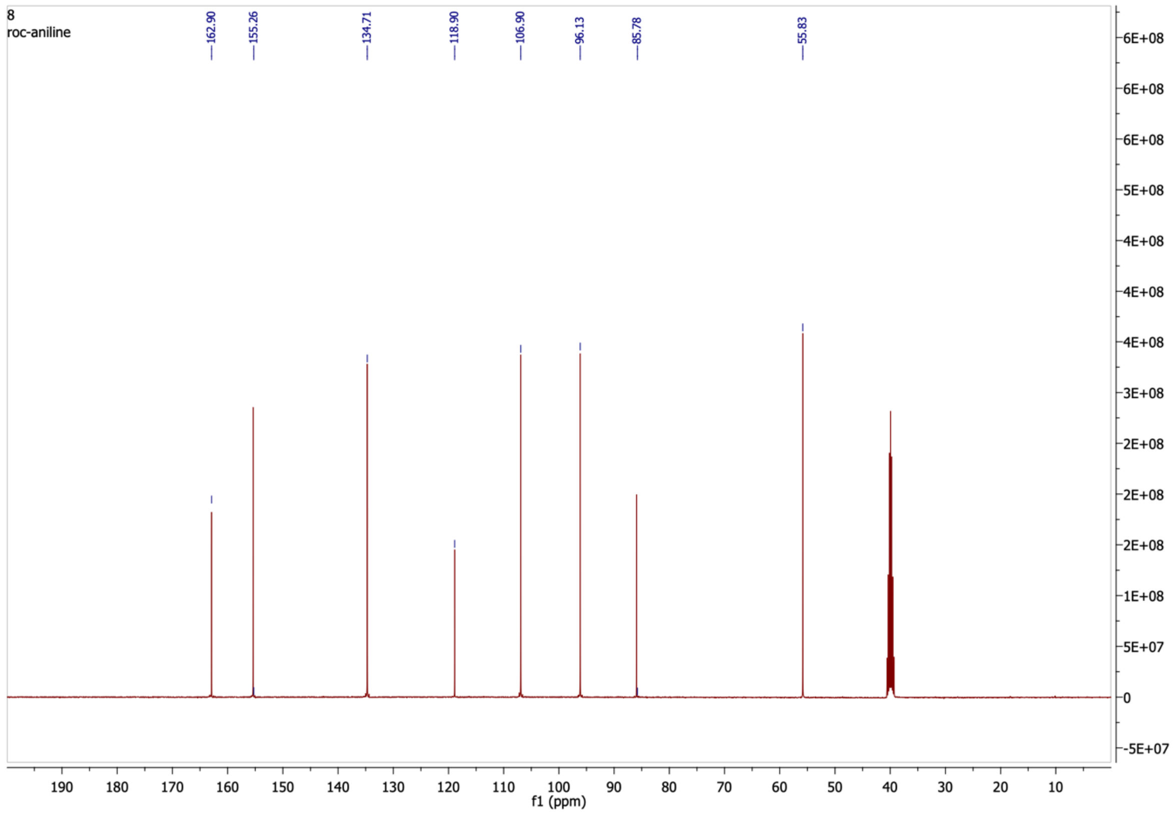

5.15. (6-Bromo-3-formyl-2-oxoquinolin-1(2*H*)-yl)methyl pivalate (**17a**)

^{1}H NMR spectrum of **17a**

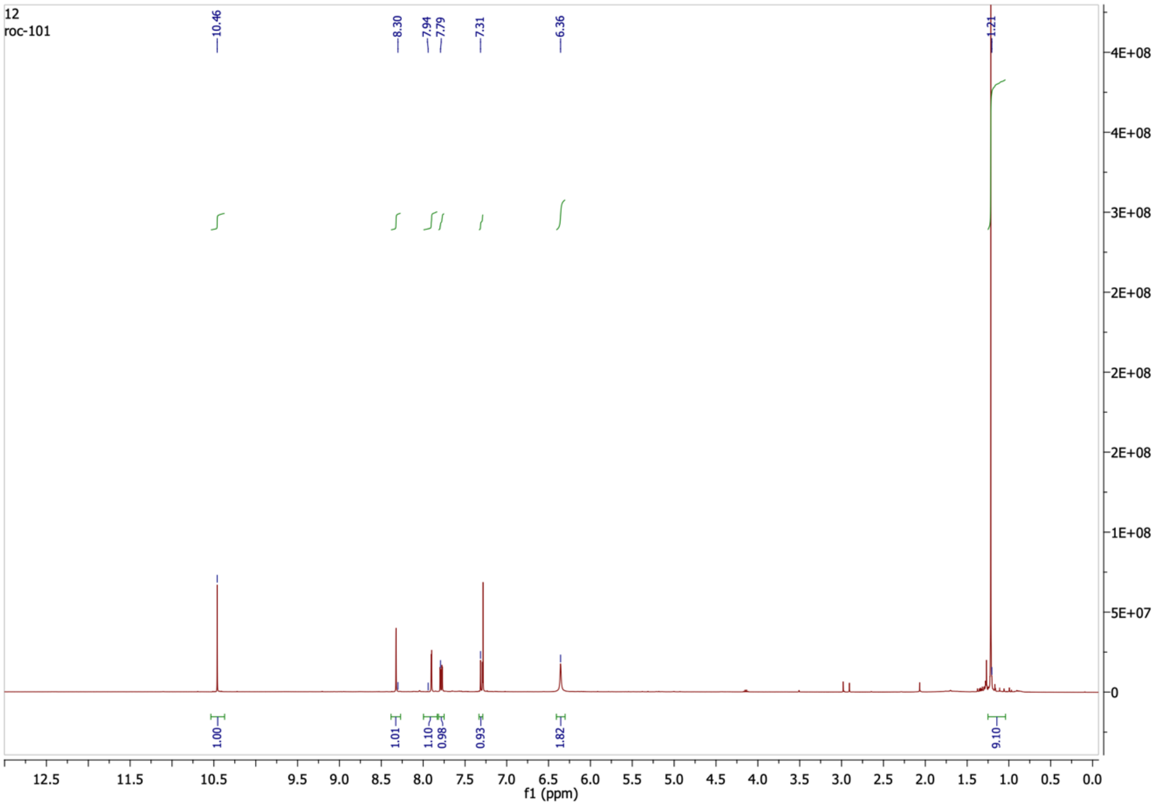

^{13}C NMR spectrum of **17a**

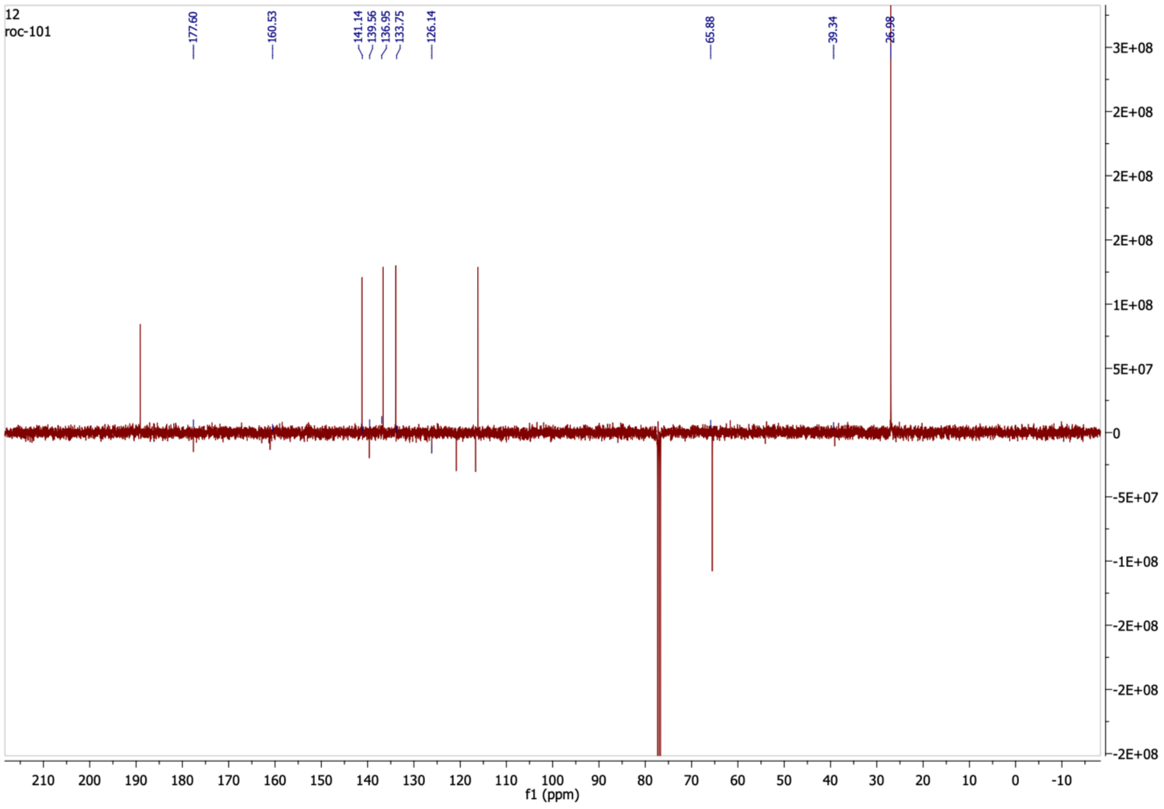

5.16. (6-Fluoro-3-formyl-2-oxoquinolin-1(2*H*)-yl)methyl pivalate (**17b**)

^{1}H NMR spectrum of **17b**

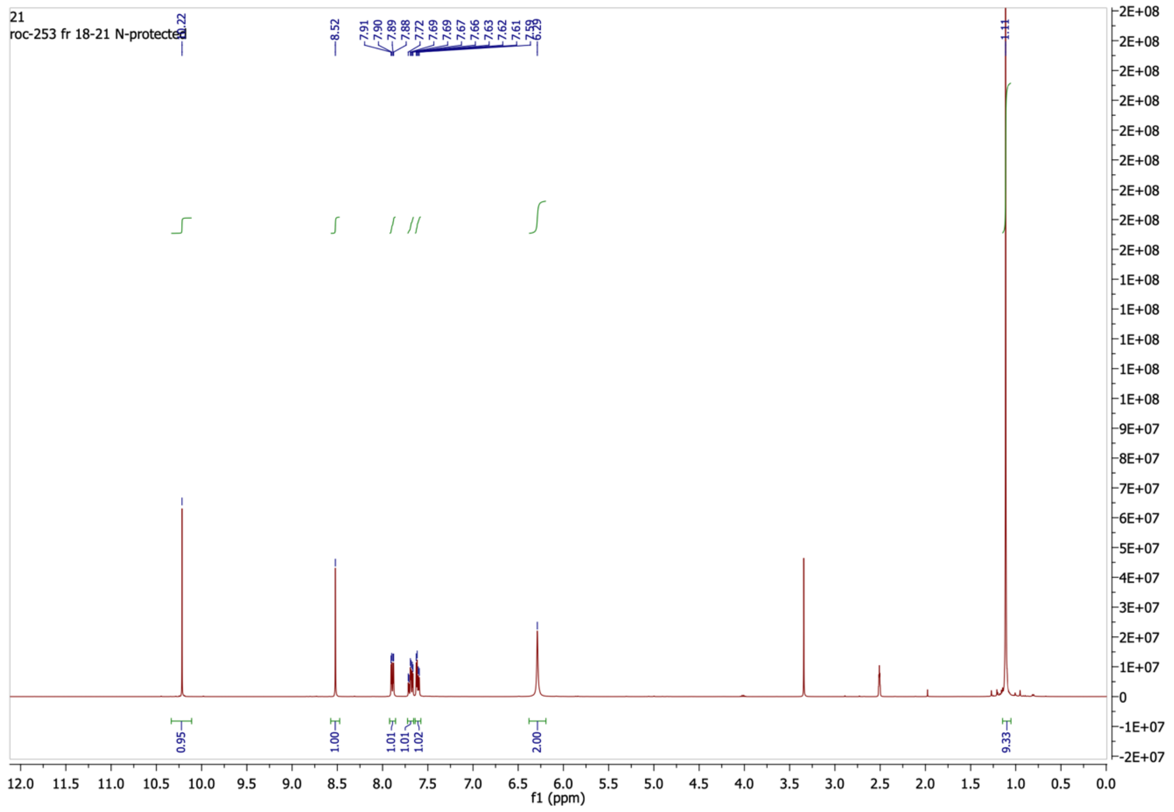

^{13}C NMR spectrum of **17b**

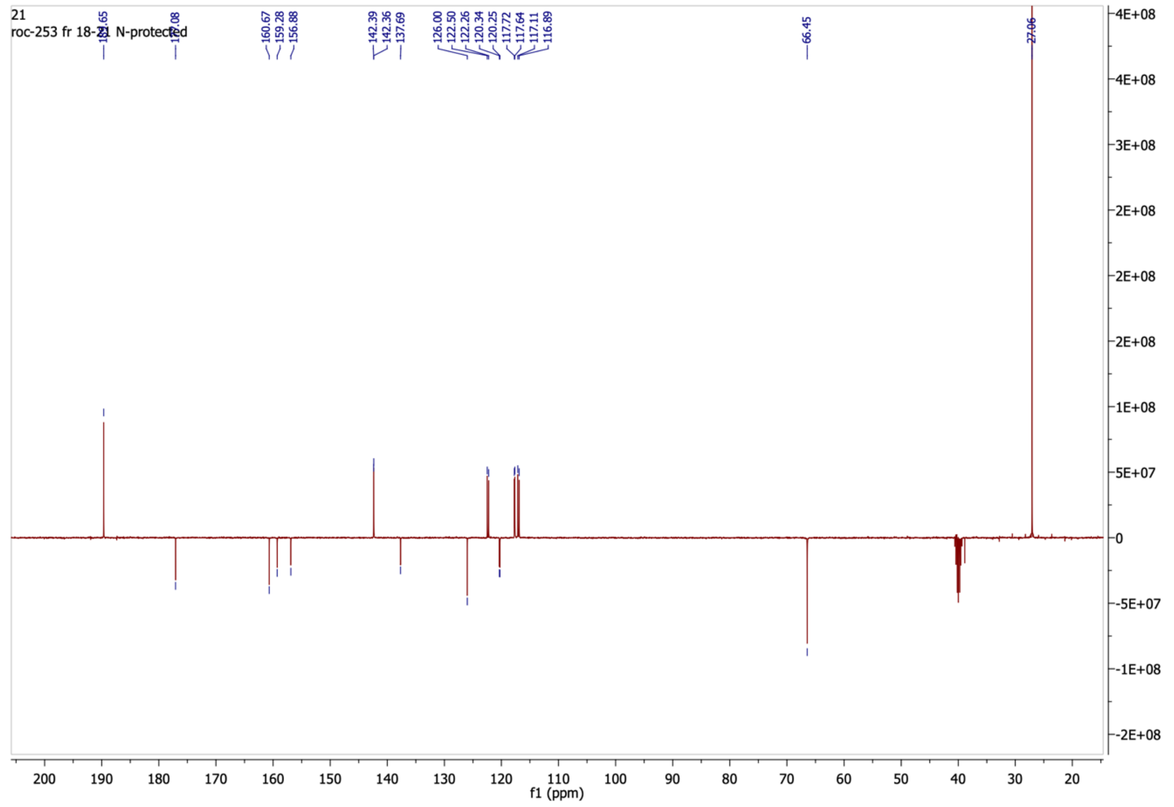

<u>^{19}F NMR spectrum of **17b**</u>

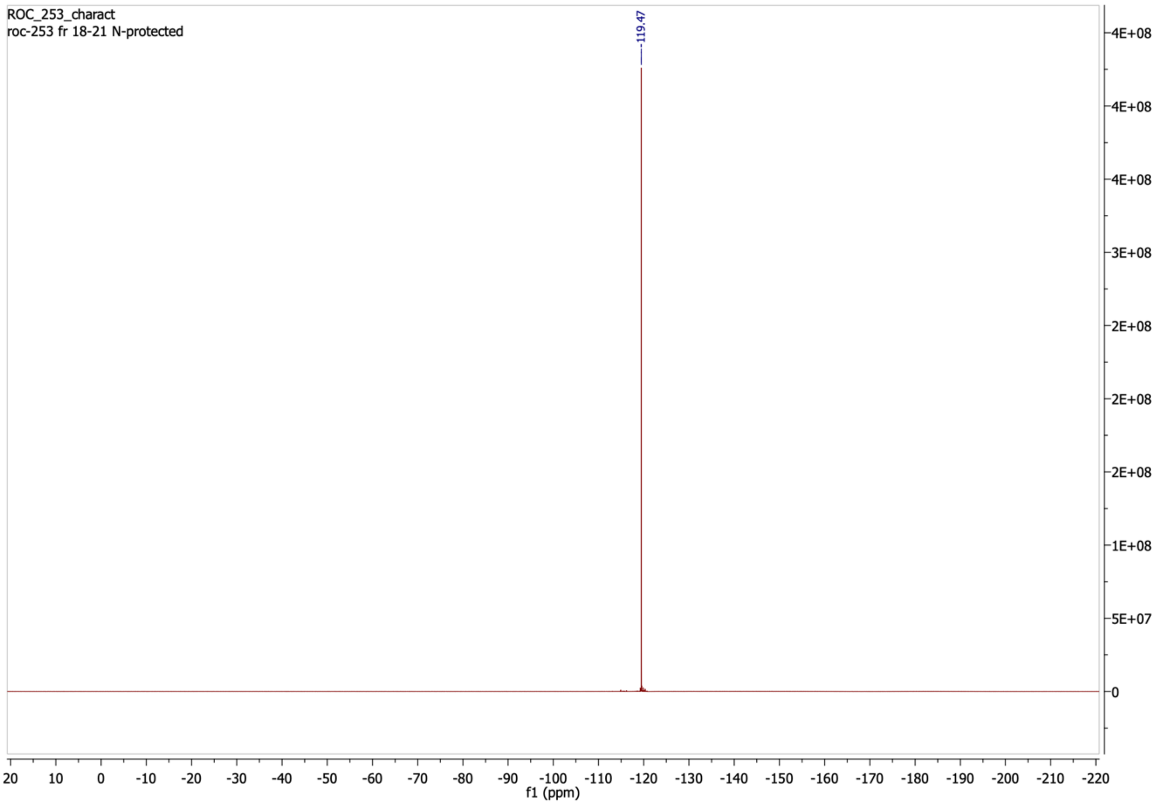

5.17. [(6-Bromo-3-formylquinolin-2-yl)oxy]methyl pivalate (**18a**)

<u>^{1}H NMR spectrum of **18a**</u>

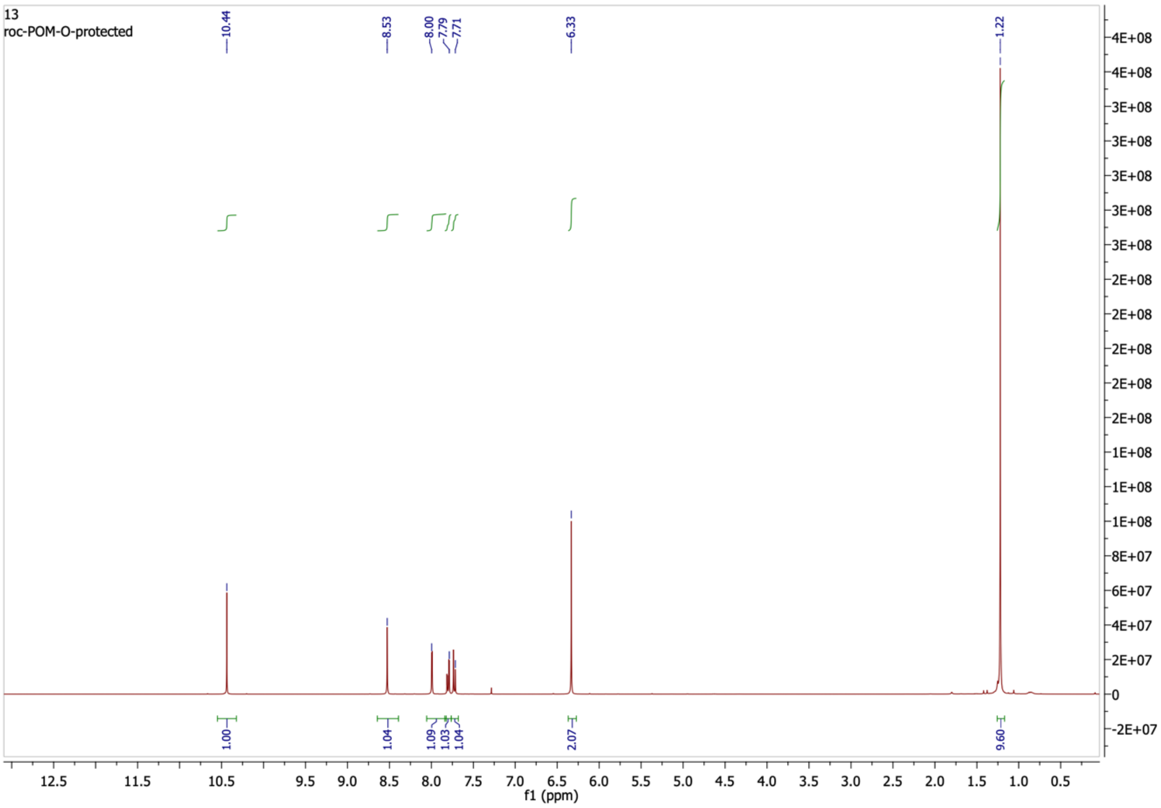

^{13}C NMR spectrum of **18a**

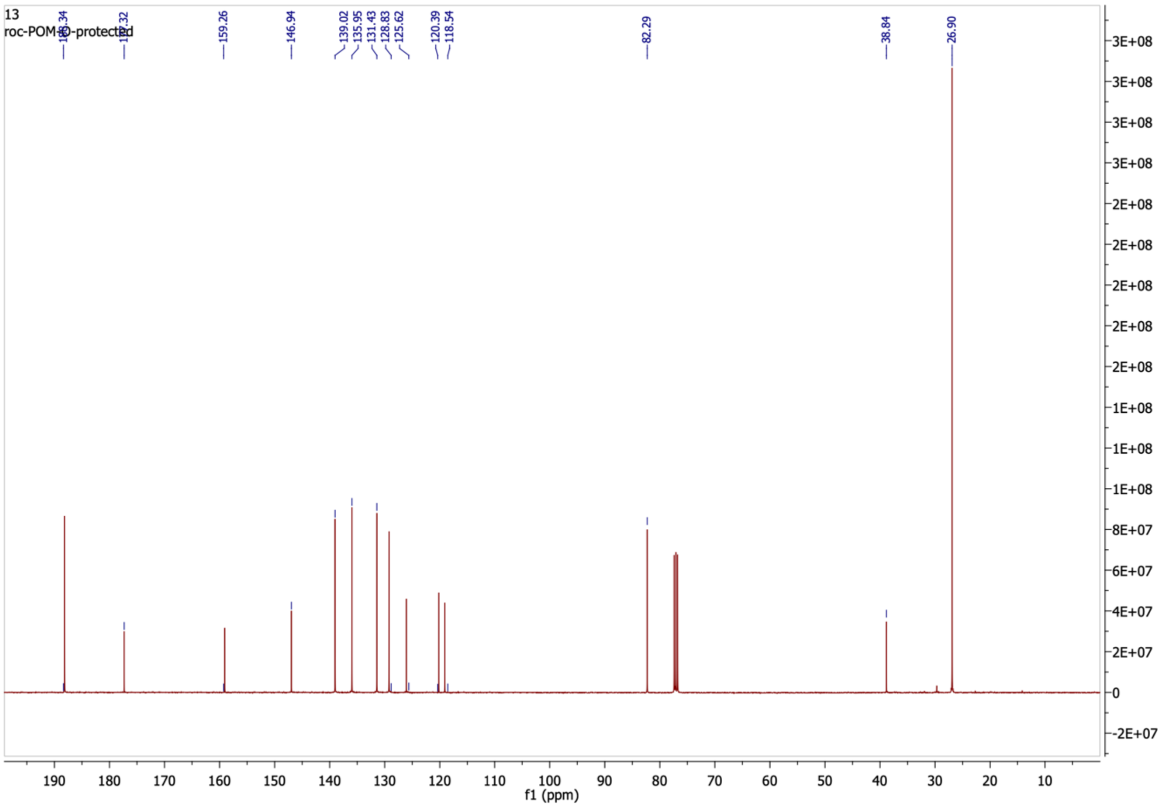

5.18. [(6-Fluoro-3-formylquinolin-2-yl)oxy]methyl pivalate (**18b**)

^{1}H NMR spectrum of **18b**

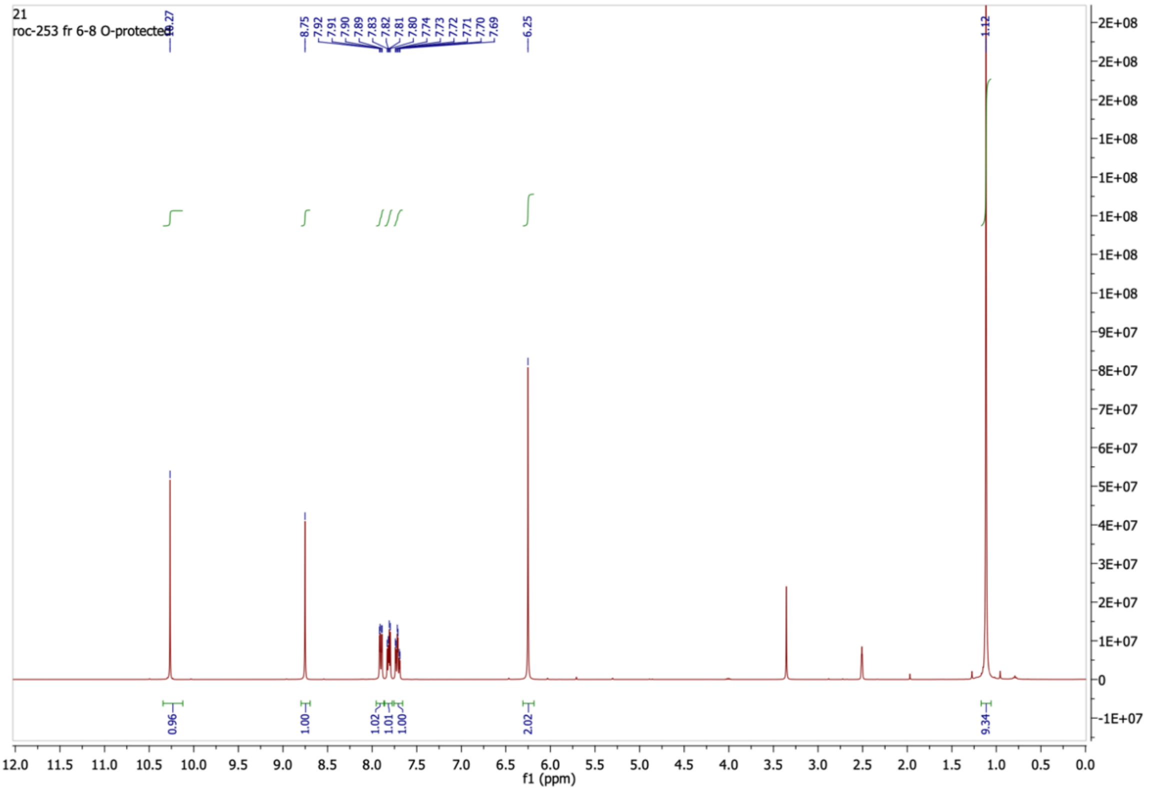

^{13}C NMR spectrum of **18b**

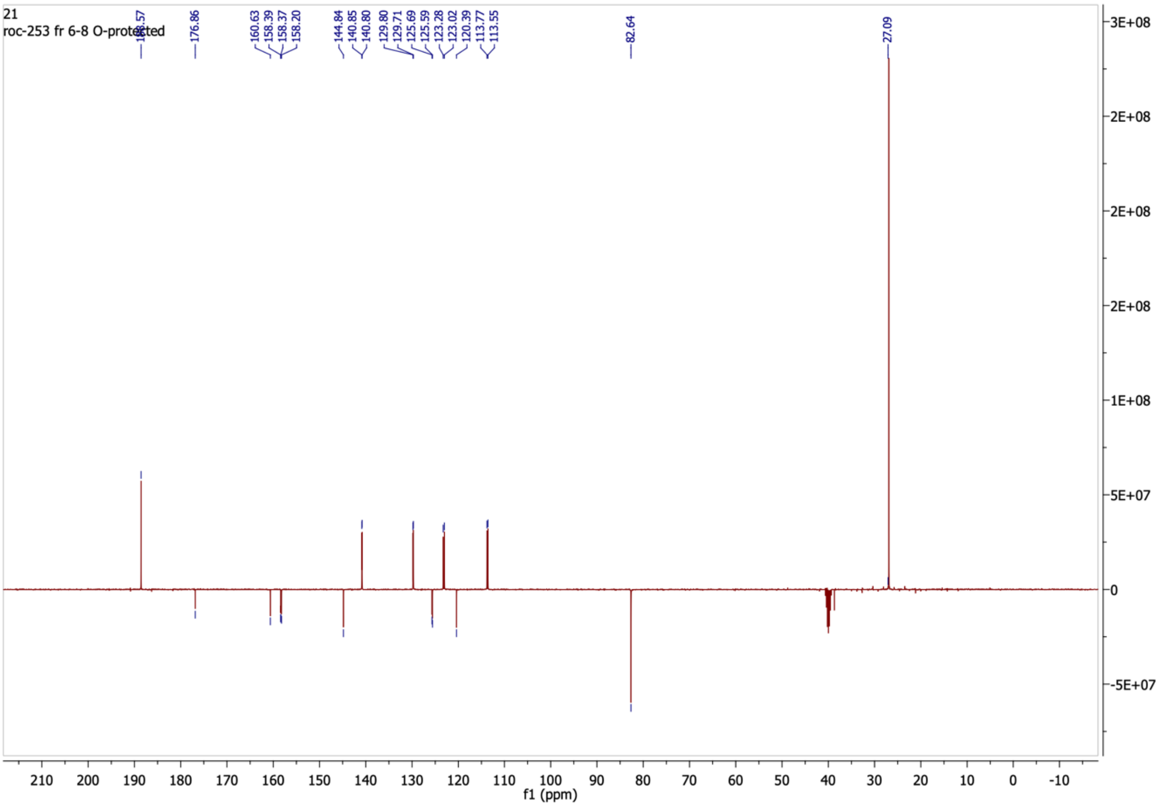

^{19}F NMR spectrum of **18b**

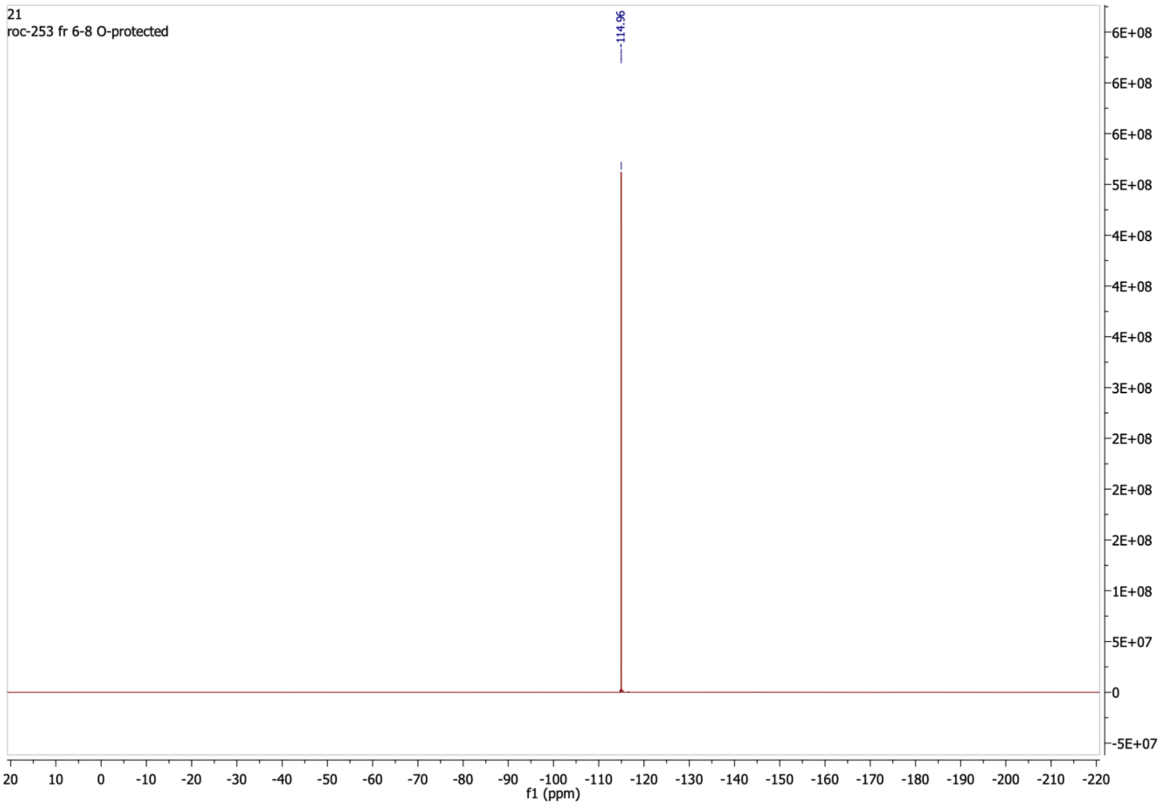

5.19. (3-Formyl-2-oxoquinolin-1(2*H*)-yl)methyl pivalate (**17c**) and [(3-formylquinolin-2-yl)oxy]methyl pivalate (**18c**)

^{1}H NMR spectrum of the mixture of **17c** and **18c**

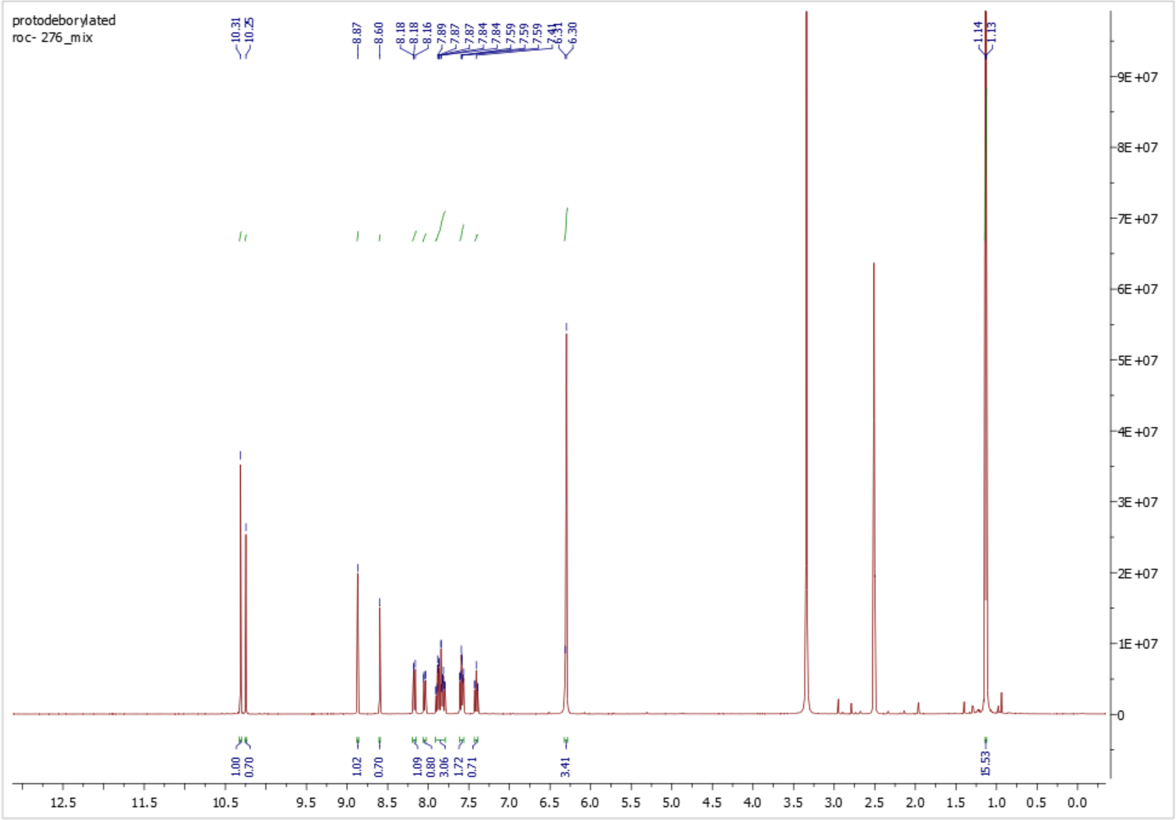

^{13}C NMR spectrum of the mixture of **17c** and **18c**

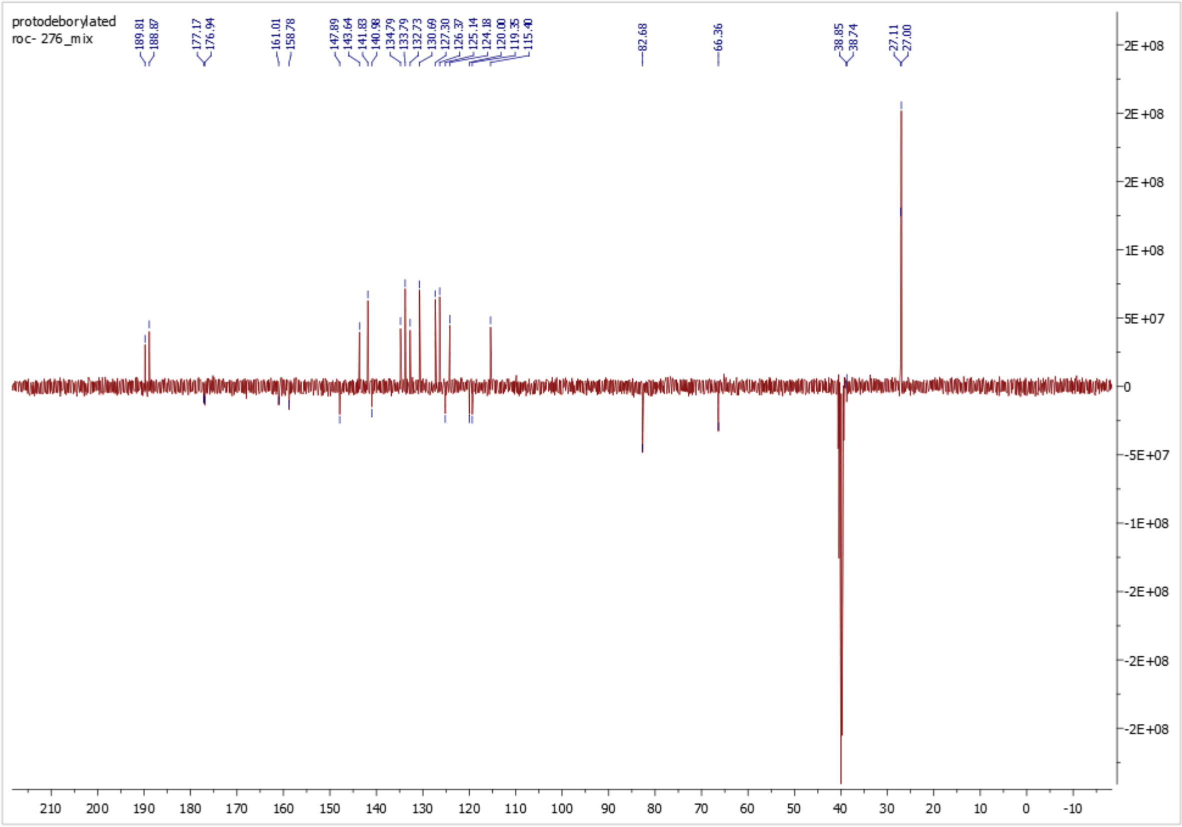

5.20. 6-Bromo-1-ethyl-2-oxo-1,2-dihydroquinoline-3-carbaldehyde (**S1a**)

<u>^{1}H NMR spectrum of **S1a**</u>

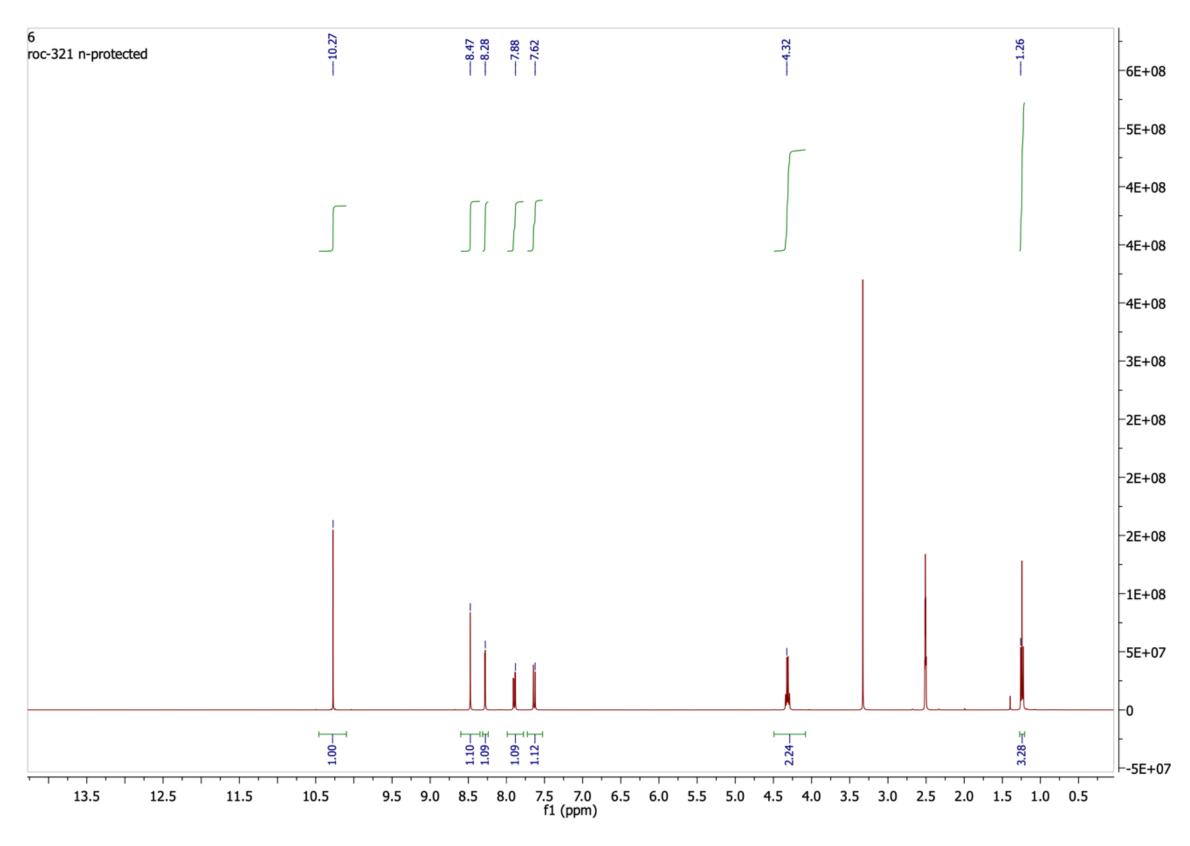

<u>^{13}C NMR spectrum of **S1a**</u>

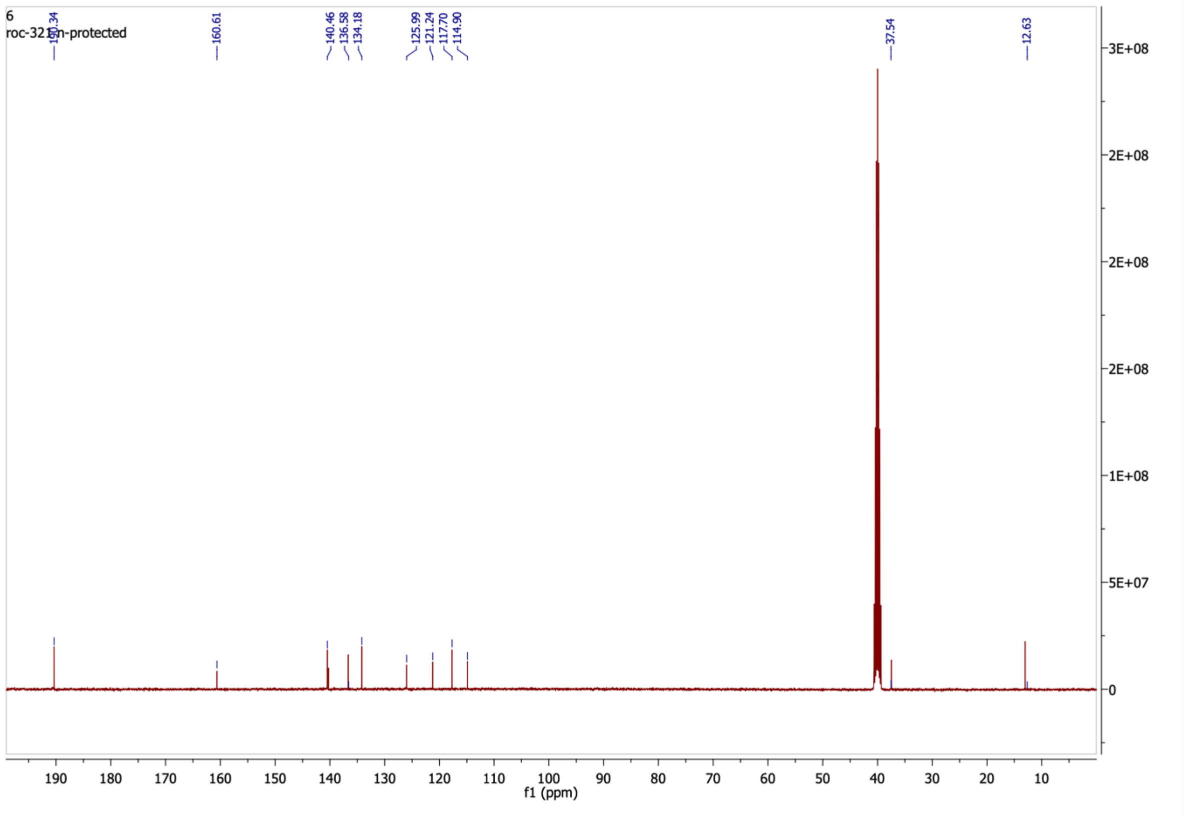

5.21. 6-Bromo-2-ethoxyquinoline-3-carbaldehyde (**S1b**)

^{1}H NMR spectrum of **S1b**

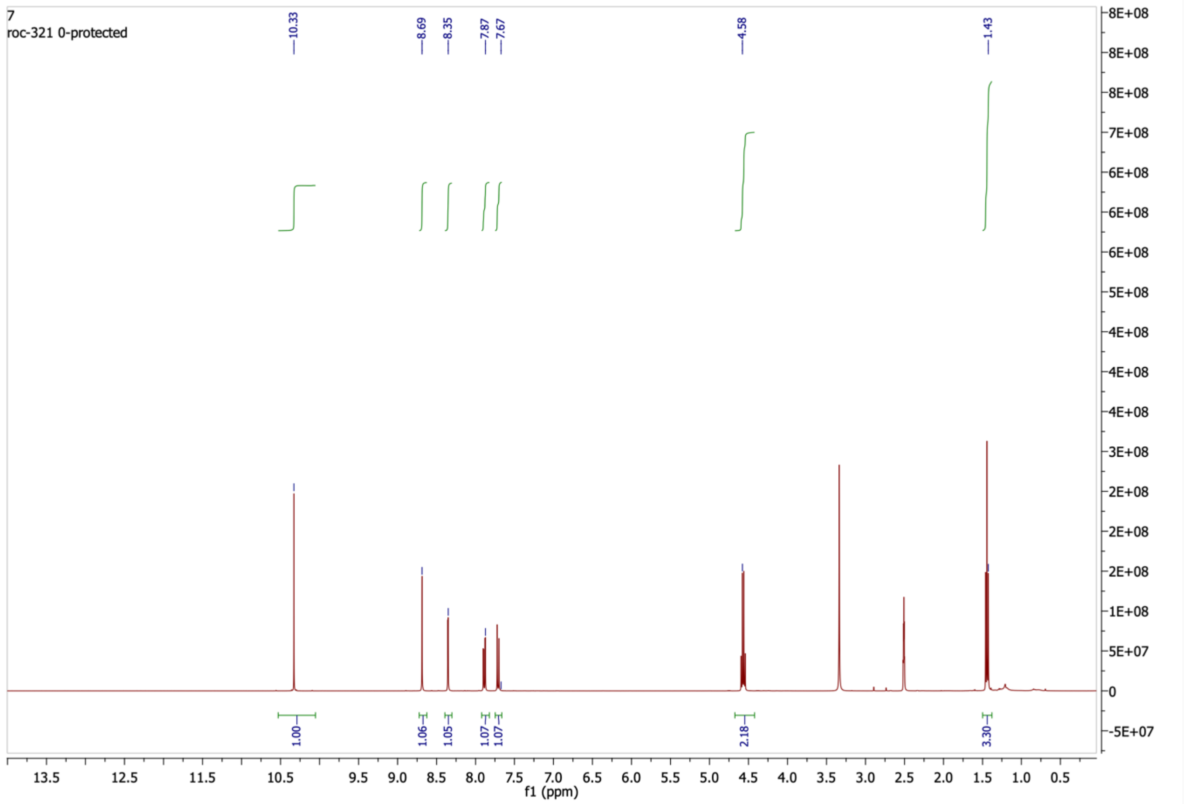

^{13}C NMR spectrum of **S1b**

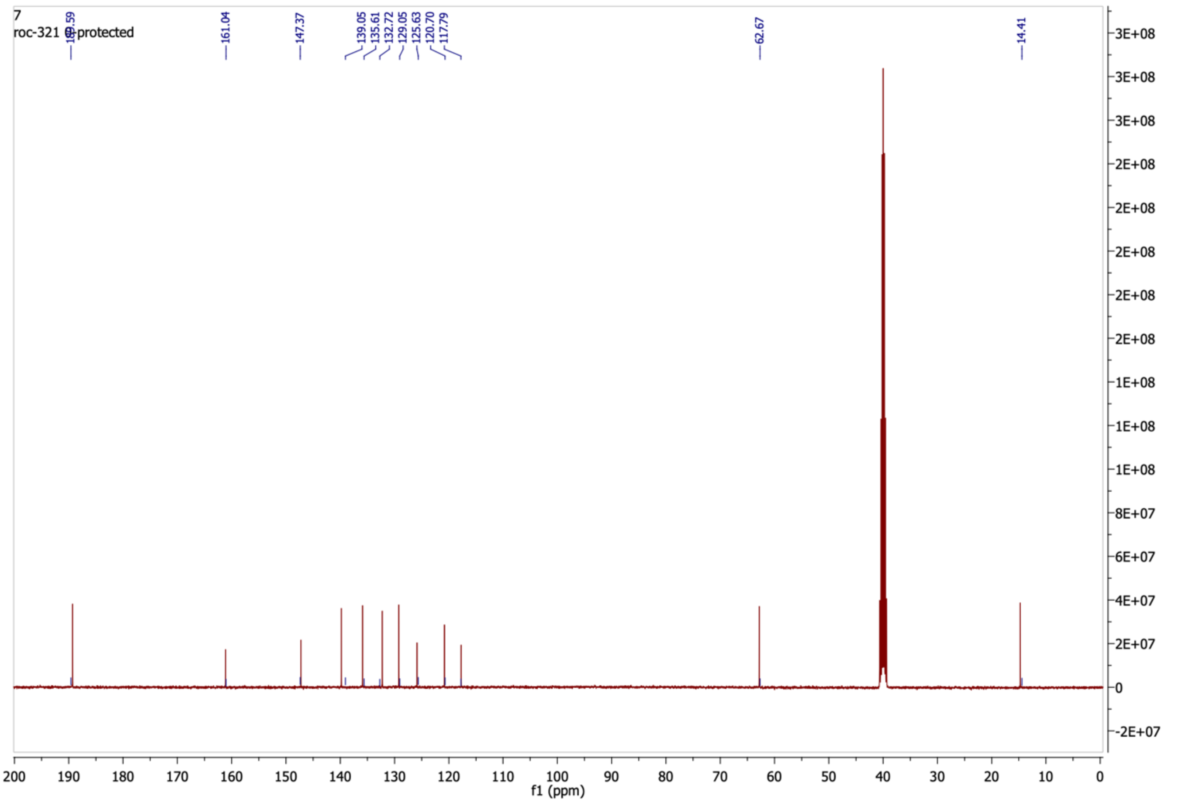

5.22. (6-Bromo-3-{[(4-cyano-3-methoxyphenyl)amino]methyl}-2-oxoquinolin-1(2*H*)-yl)methyl pivalate (**19a**)

^{1}H NMR spectrum of **19a**

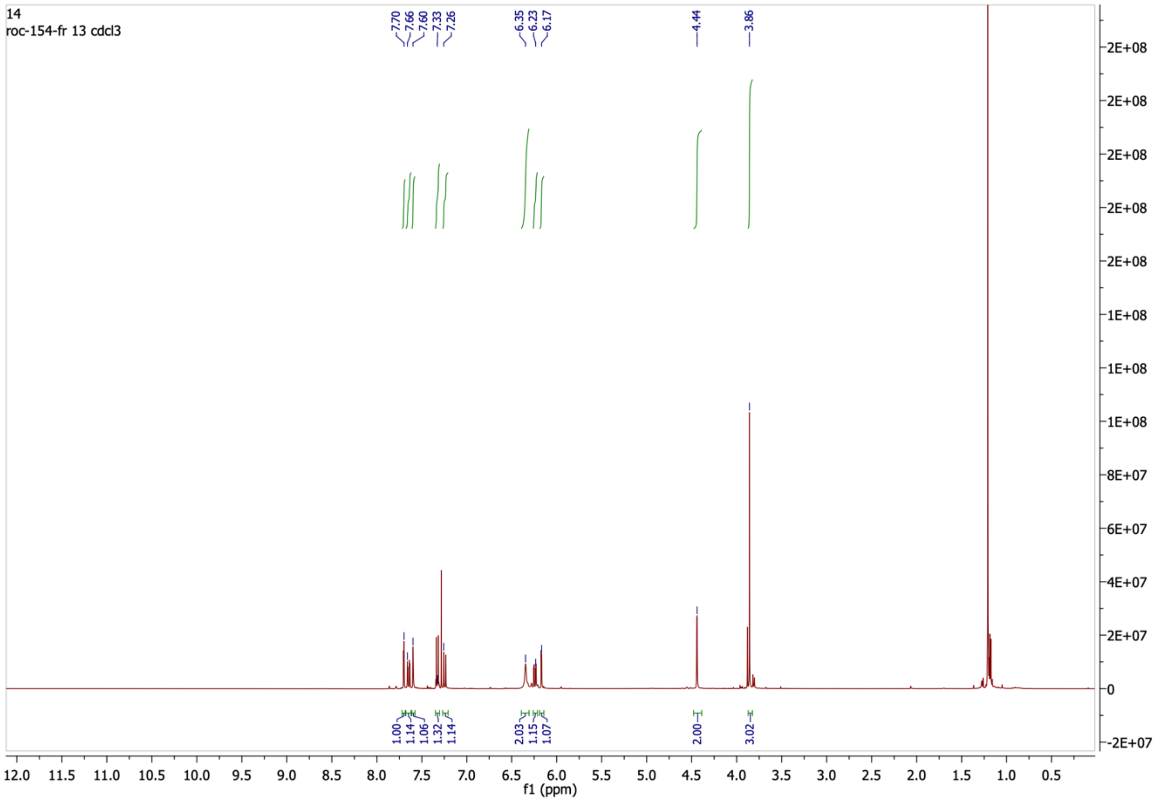

^{13}C NMR spectrum of **19a**

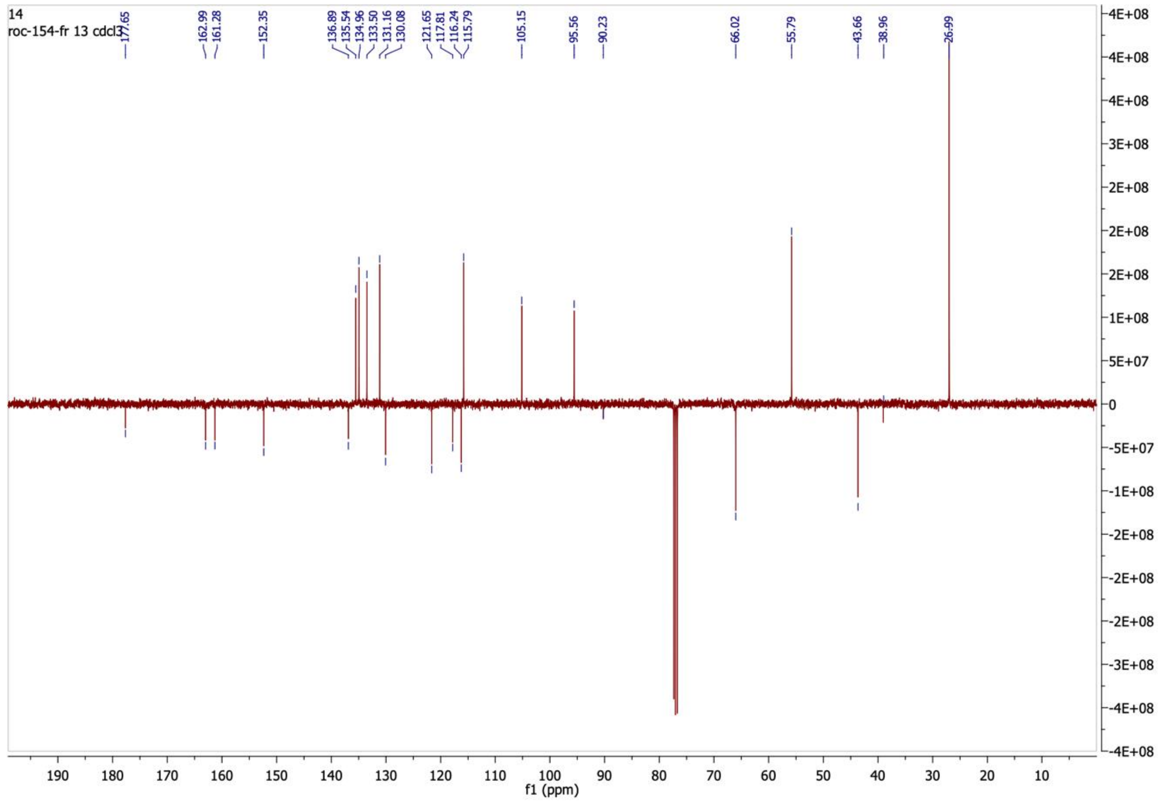

HSQC NMR spectrum of **19a**

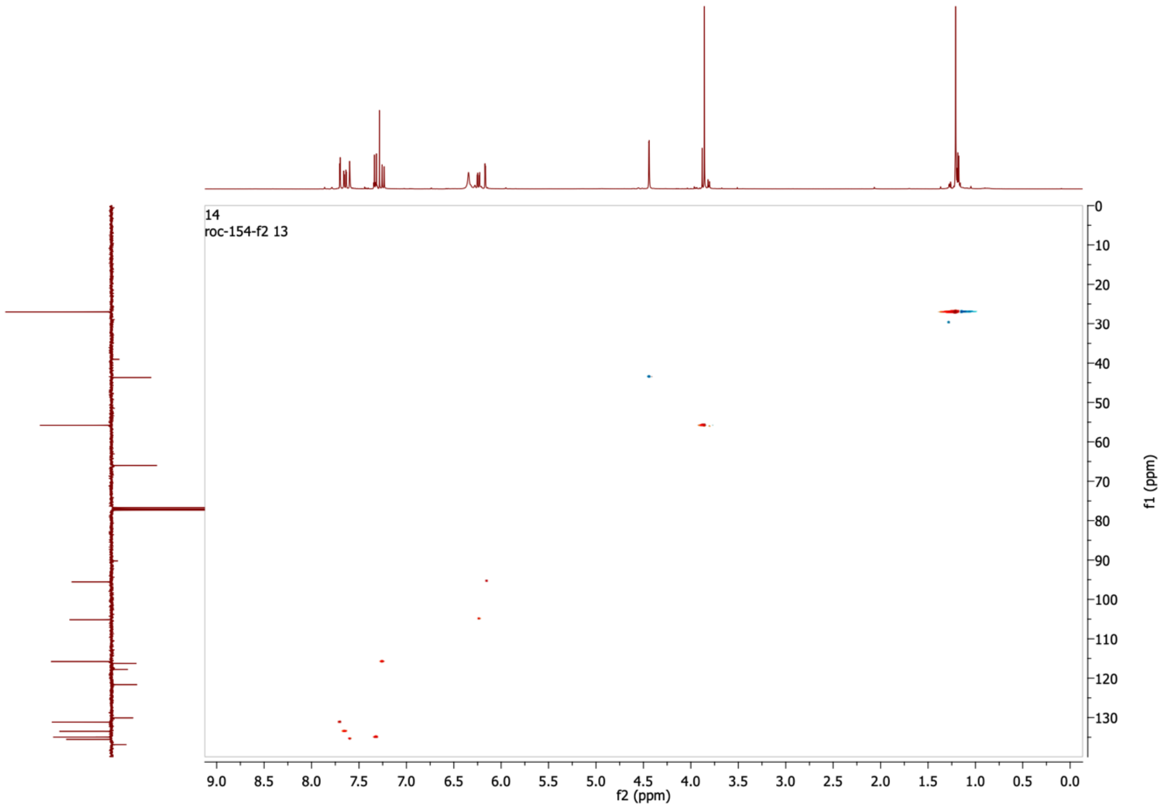

5.23. [(6-Bromo-3-[{(4-cyano-3-methoxyphenyl)amino}methyl]quinolin-2-yl)oxy]methyl pivalate (**20a**)

^{1}H NMR spectrum of **20a**

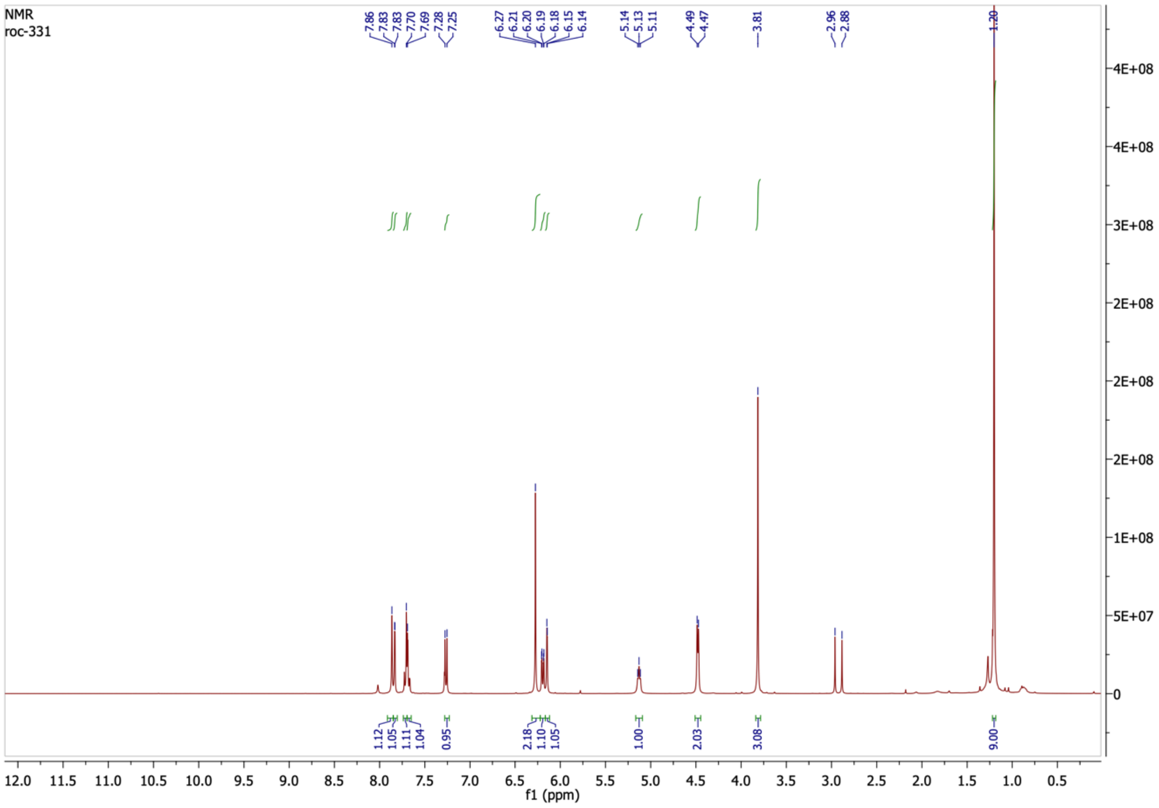

^{13}C NMR spectrum of **20a**

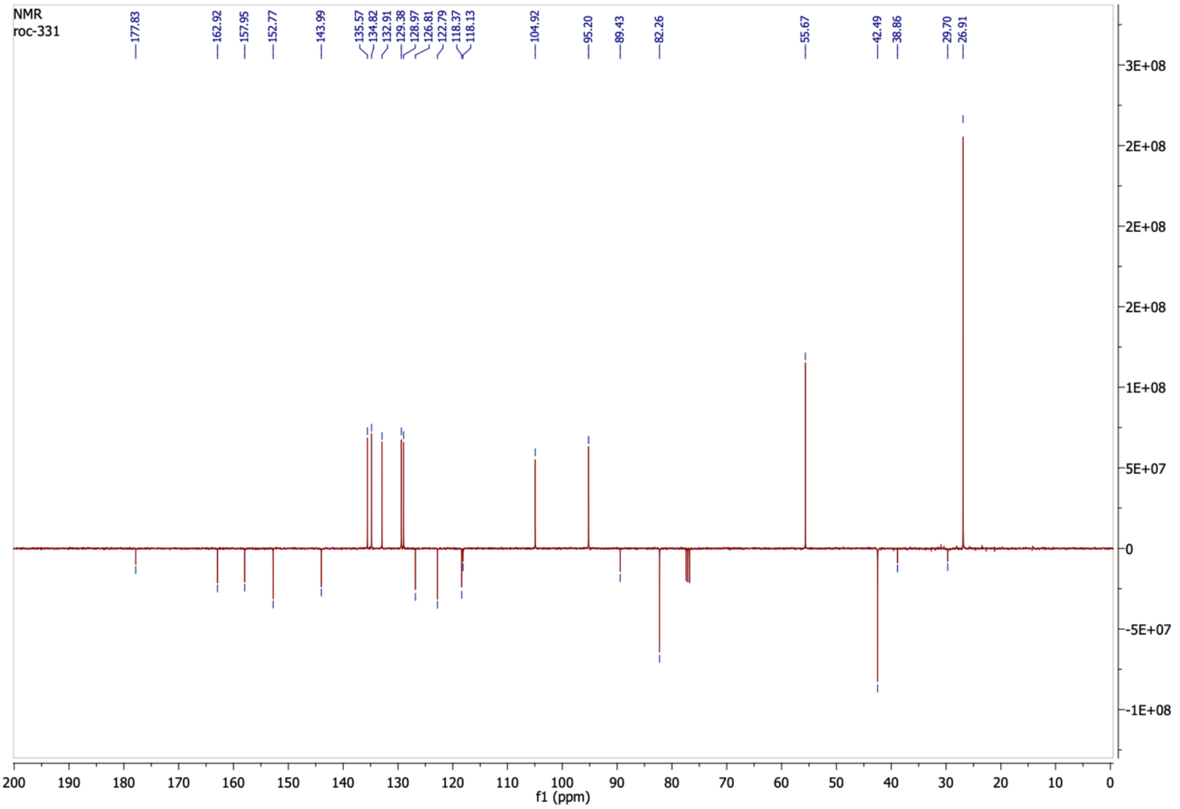

HSQC NMR spectrum of **20a**

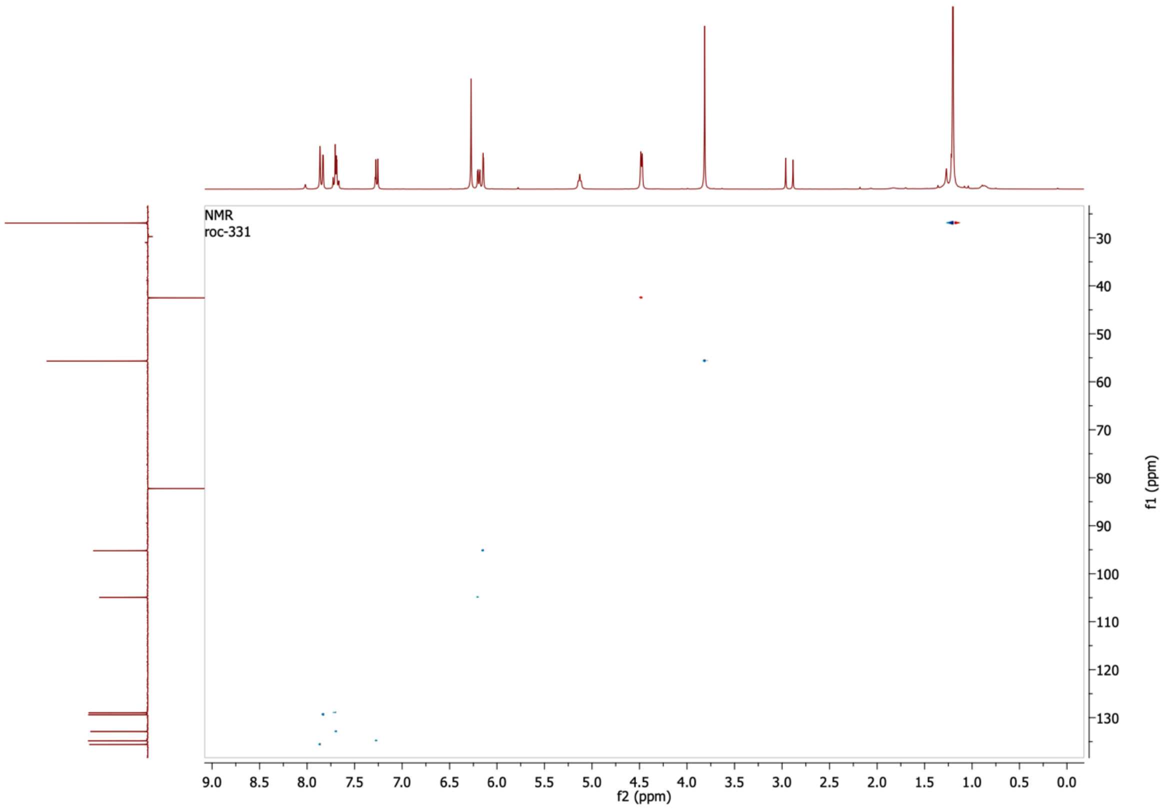

5.24. (3-[{(4-Cyano-3-methoxyphenyl)amino}methyl]-2-oxoquinolin-1(2*H*)-yl)methyl pivalate (**19c**)

^{1}H NMR spectrum of **19c**

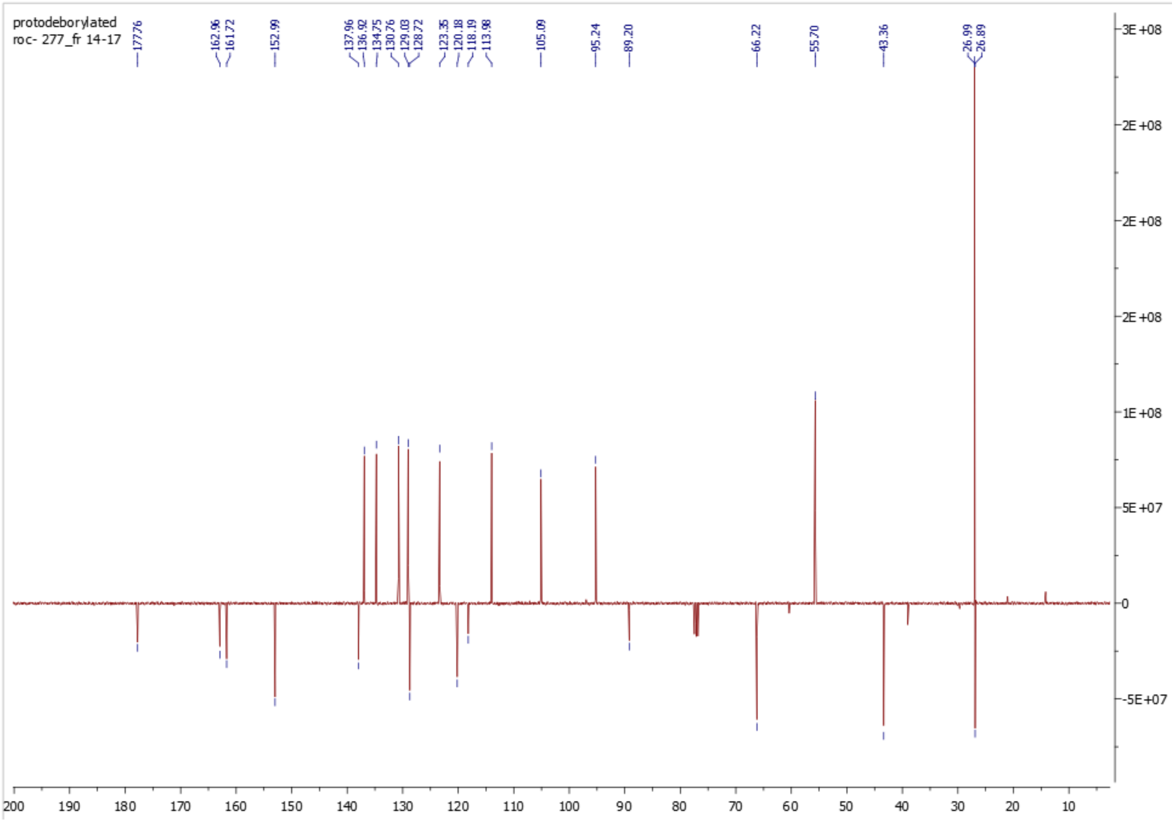

^{13}C NMR spectrum of **19c**

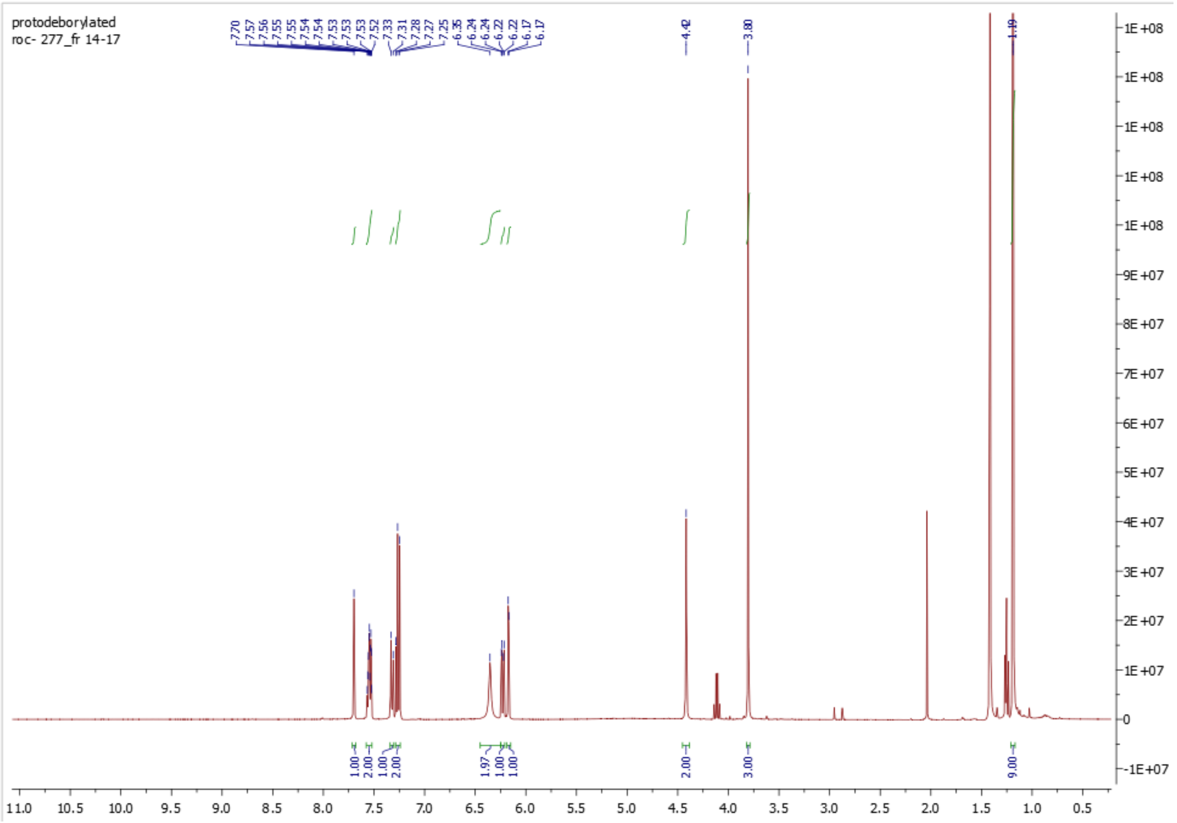

5.25. [(3-[{(4-Cyano-3-methoxyphenyl)amino}methyl]quinolin-2-yl)oxy]methyl pivalate (**20c**)

^{1}H NMR spectrum of **20c**

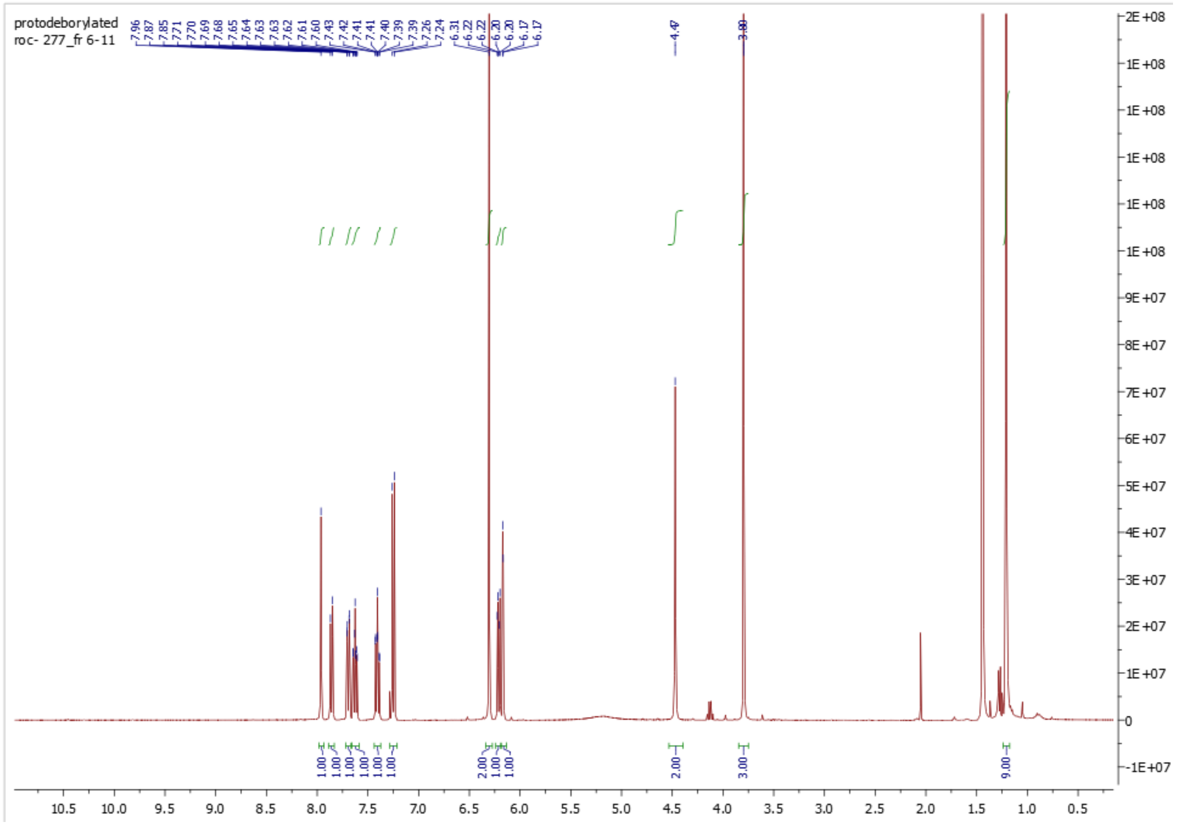

^{13}C NMR spectrum of **20c**

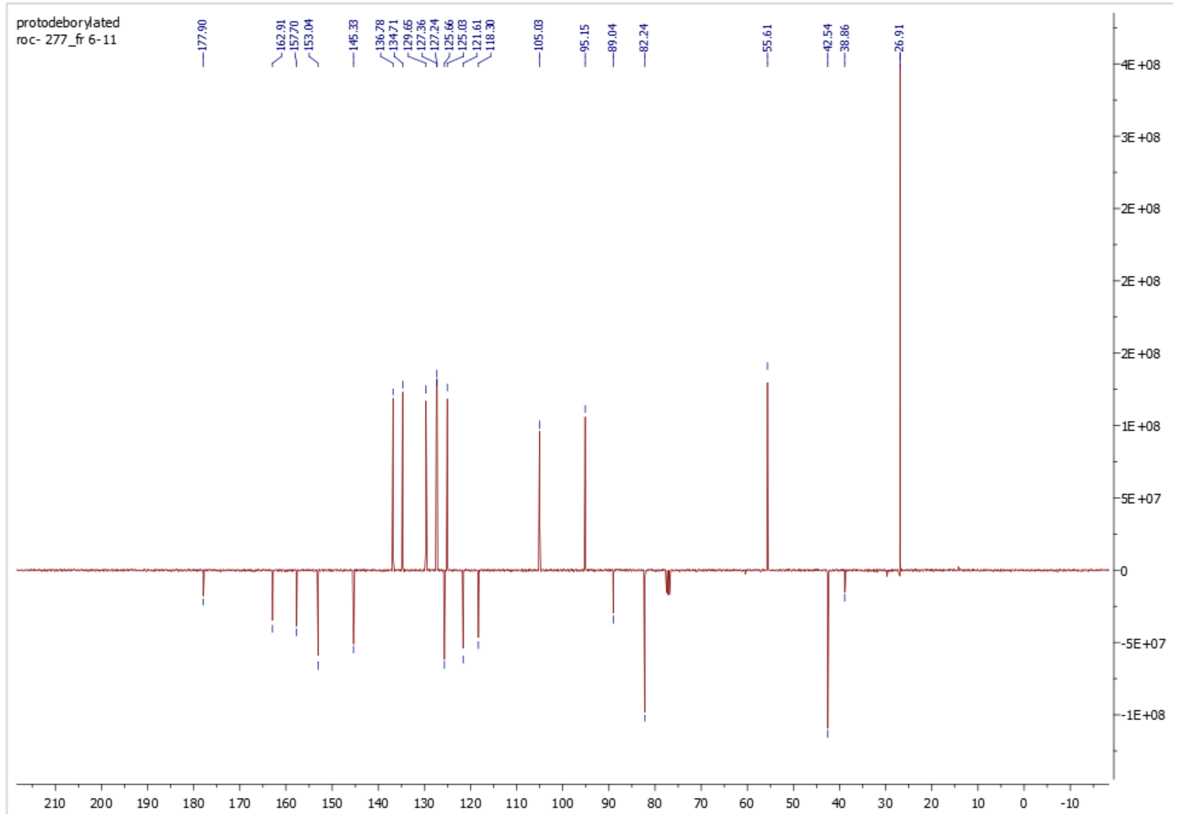

5.26. (3-[{(4-Cyano-3-methoxyphenyl)amino}methyl]-2-oxo-6-(4,4,5,5-tetramethyl-1,3,2-dioxaborolan-2-yl)quinoline-1(2*H*)-yl)methyl pivalate (**21**)

^{1}H NMR spectrum of **21**

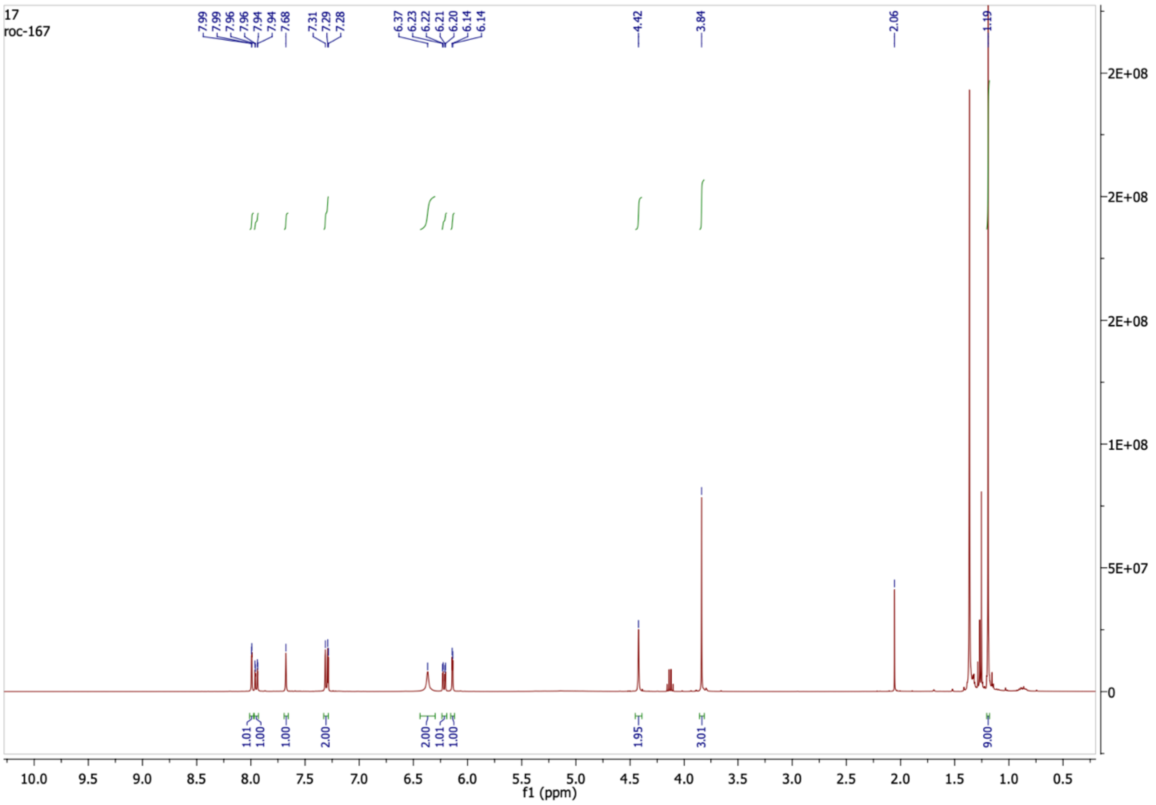

^{13}C NMR spectrum of **21**

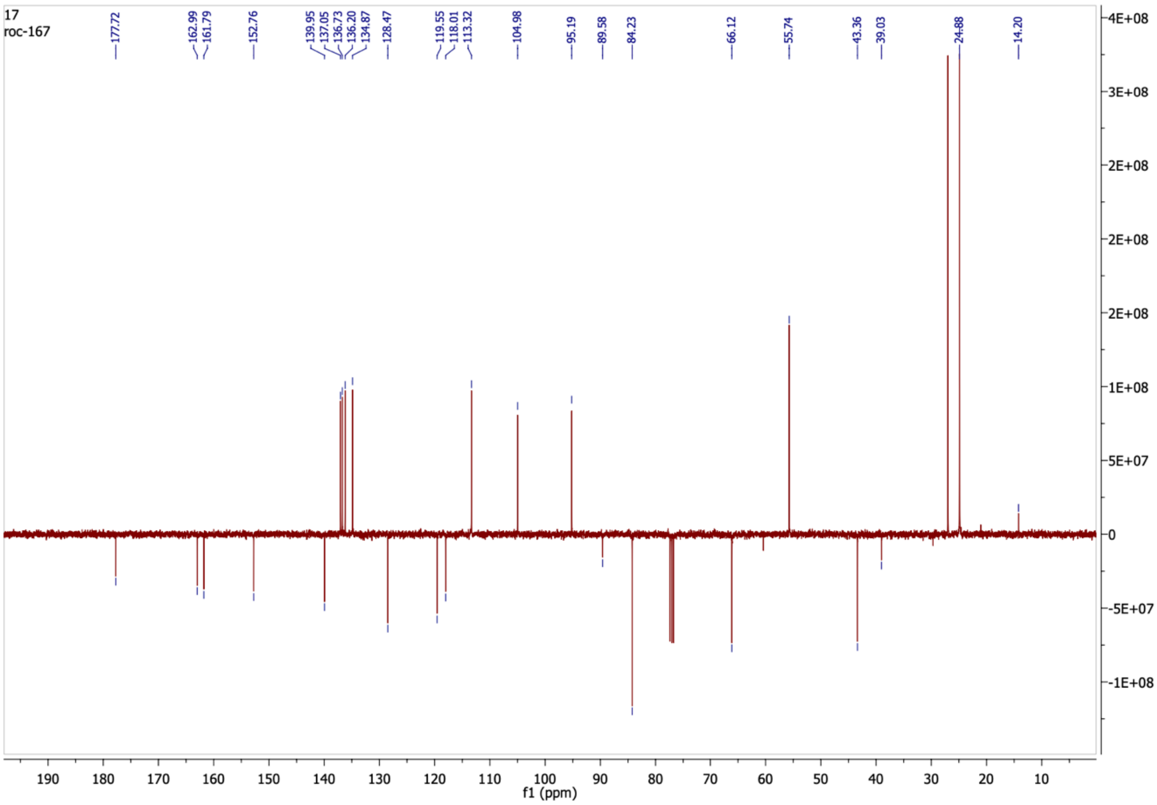

5.27. [(3-[{(4-Cyano-3-methoxyphenyl)amino}methyl]-6-(4,4,5,5-tetramethyl-1,3,2-dioxaborolan-2-yl)quinolin-2-yl)oxy]methyl pivalate (**22**)

^{1}H NMR spectrum of **22**

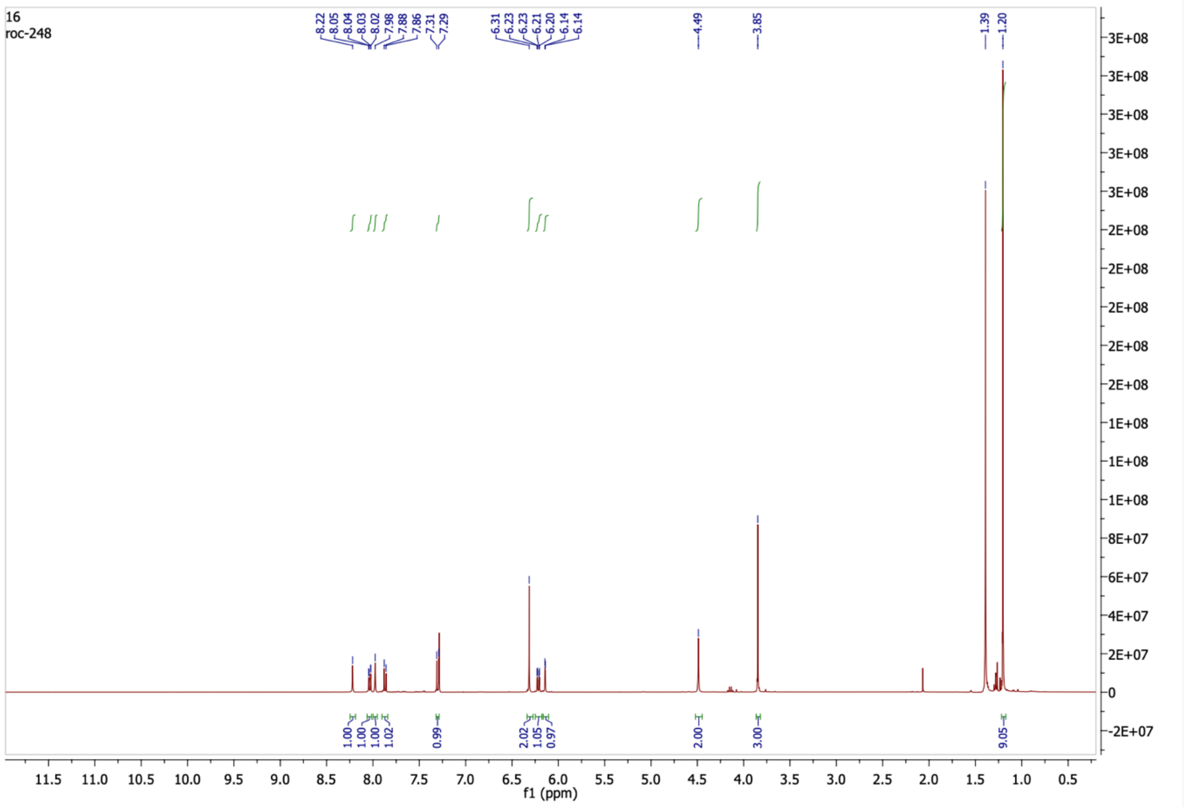

^{13}C NMR spectrum of **22**

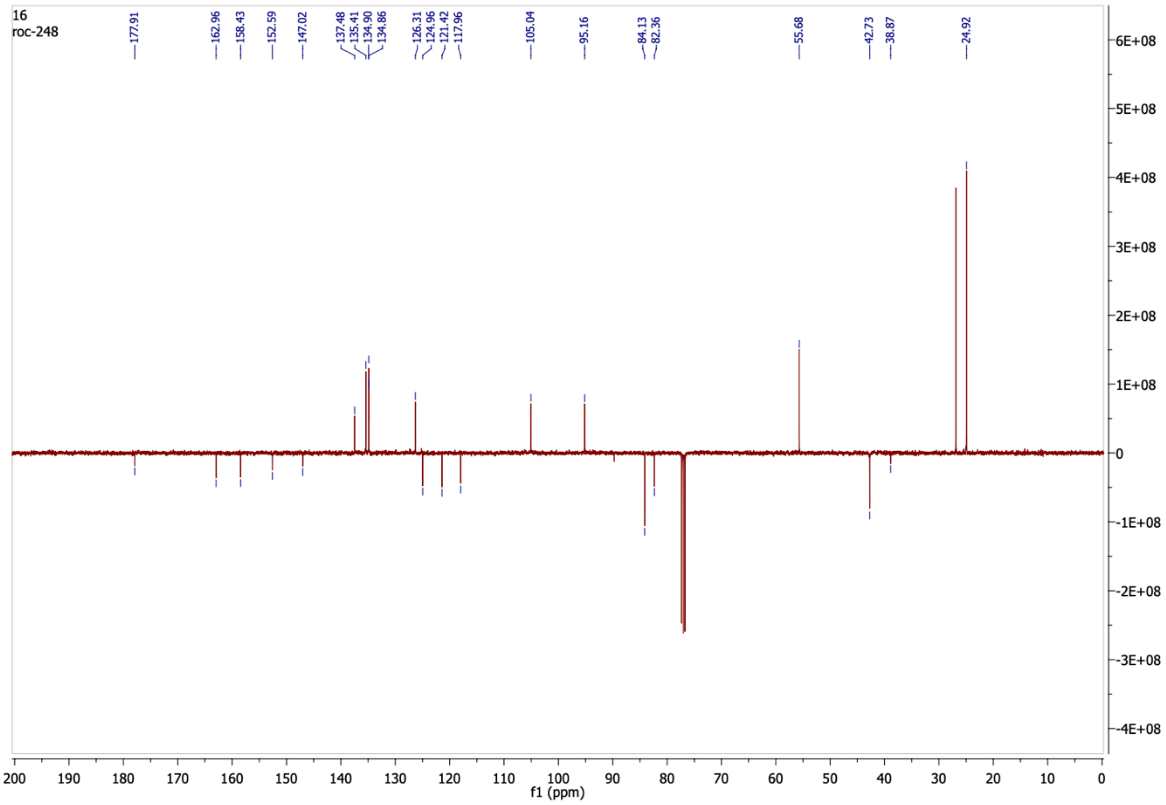

5.28. (*R*)-*N*-[(6-bromo-2-chloroquinolin-3-yl)methylene]-2-methylpropane-2-sulfinamide (**23a**)

^{1}H NMR spectrum of **23a**

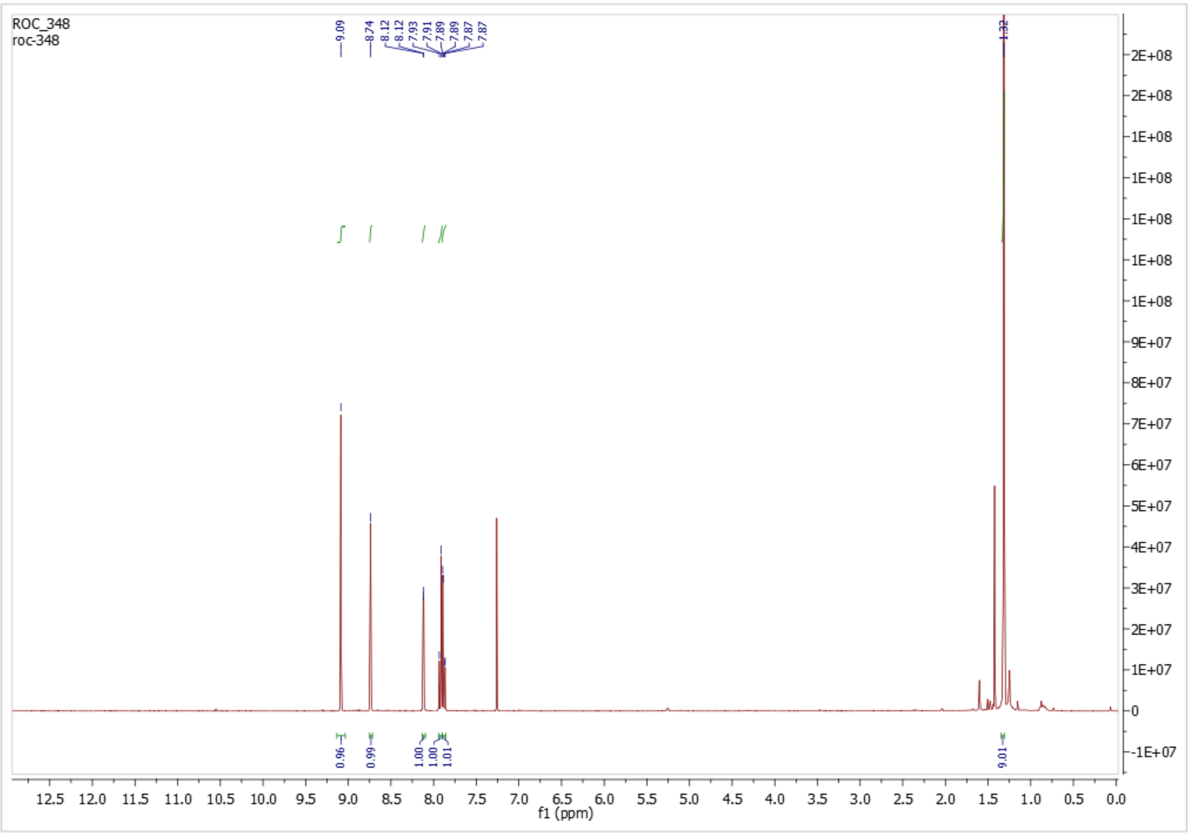

^{13}C NMR spectrum of **23a**

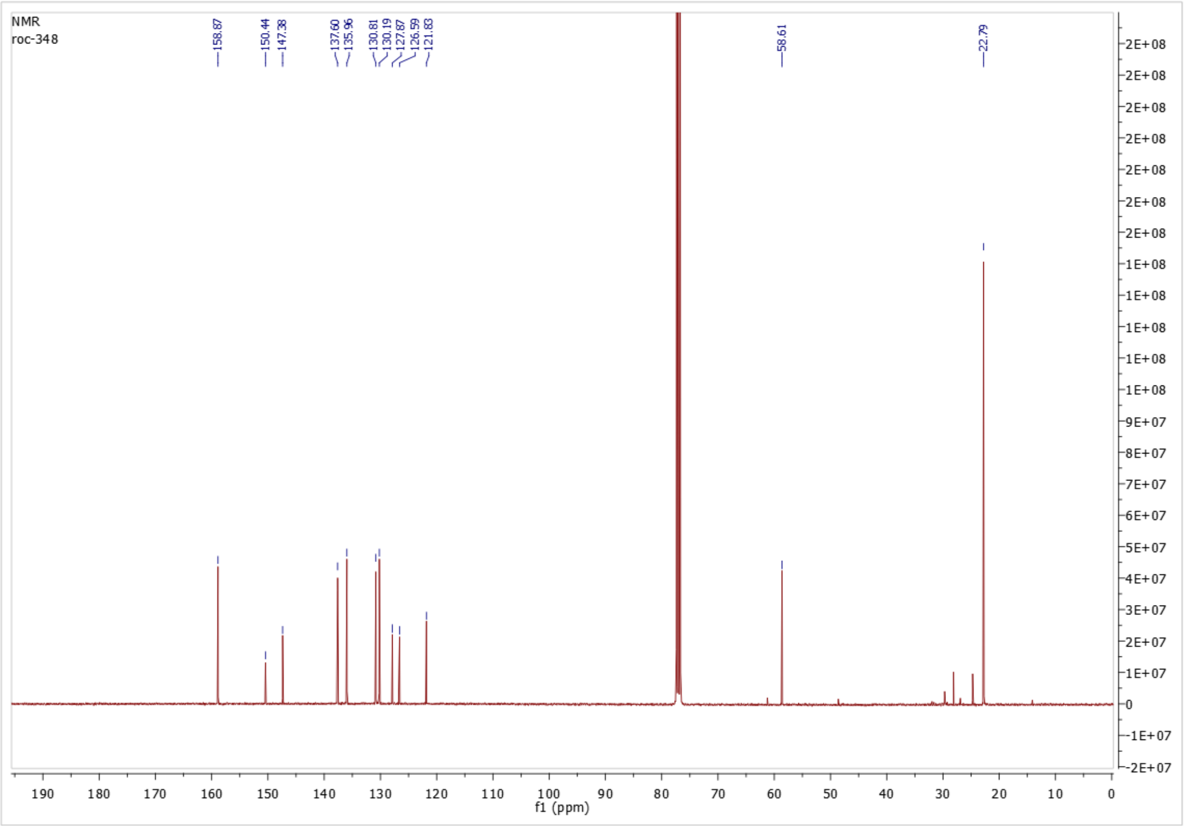

5.29. (*R*)-*N*-[(6-Fluoro-2-chloroquinolin-3-yl)methylene]-2-methylpropane-2-sulfinamide (**23b**)

^{1}H NMR spectrum of **23b**

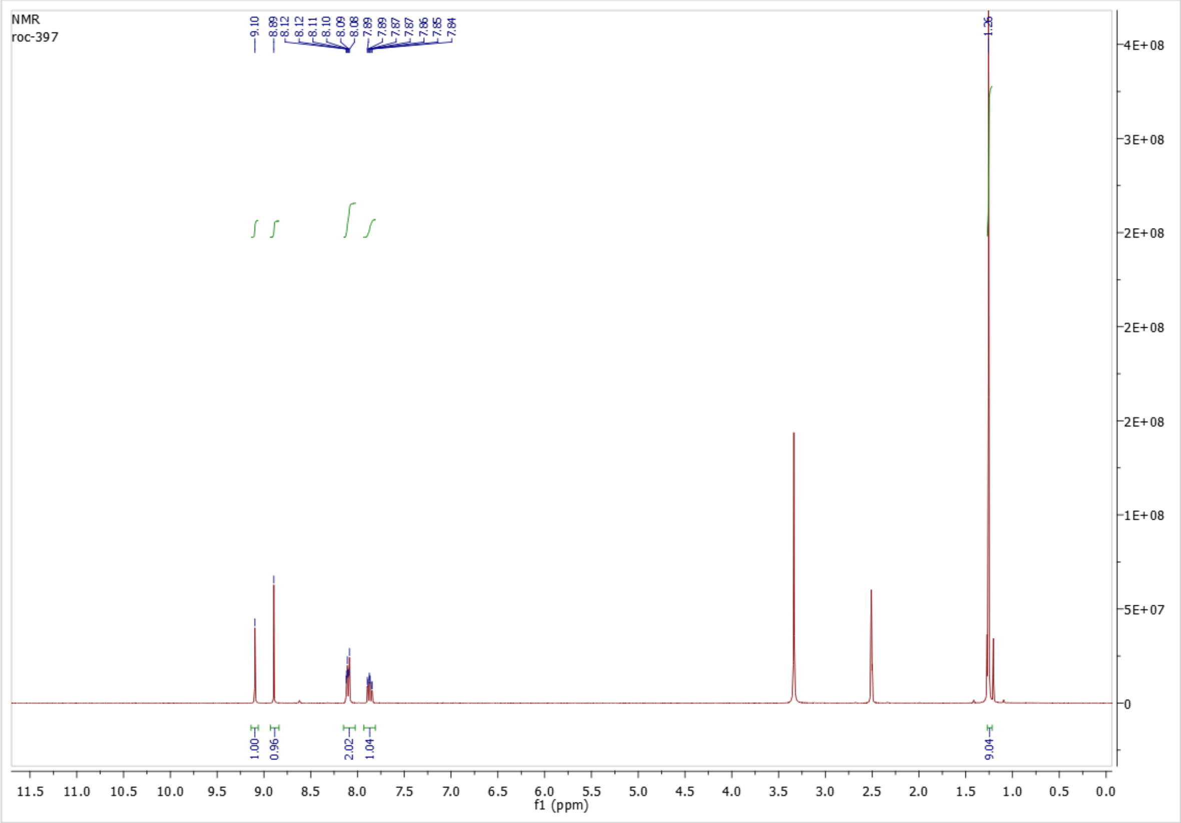

^{13}C NMR spectrum of **23b**

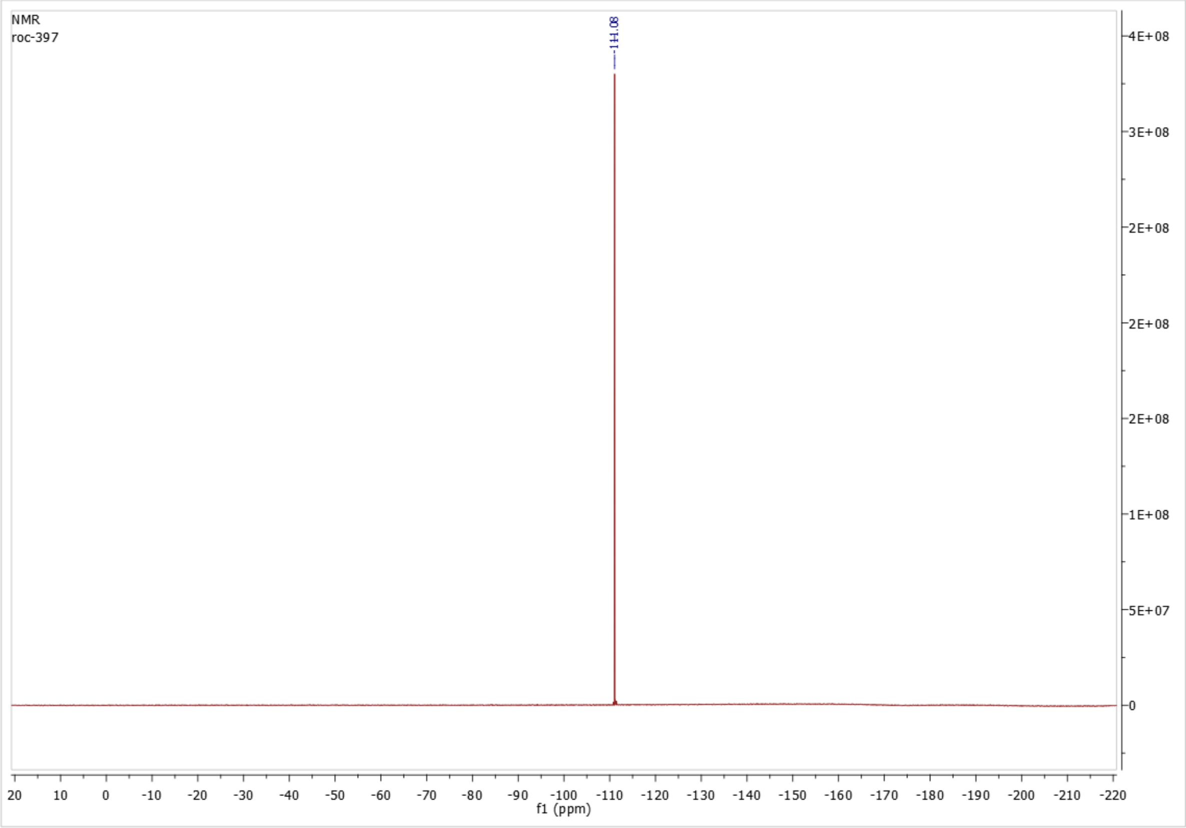

^{19}F NMR spectrum of **23b**

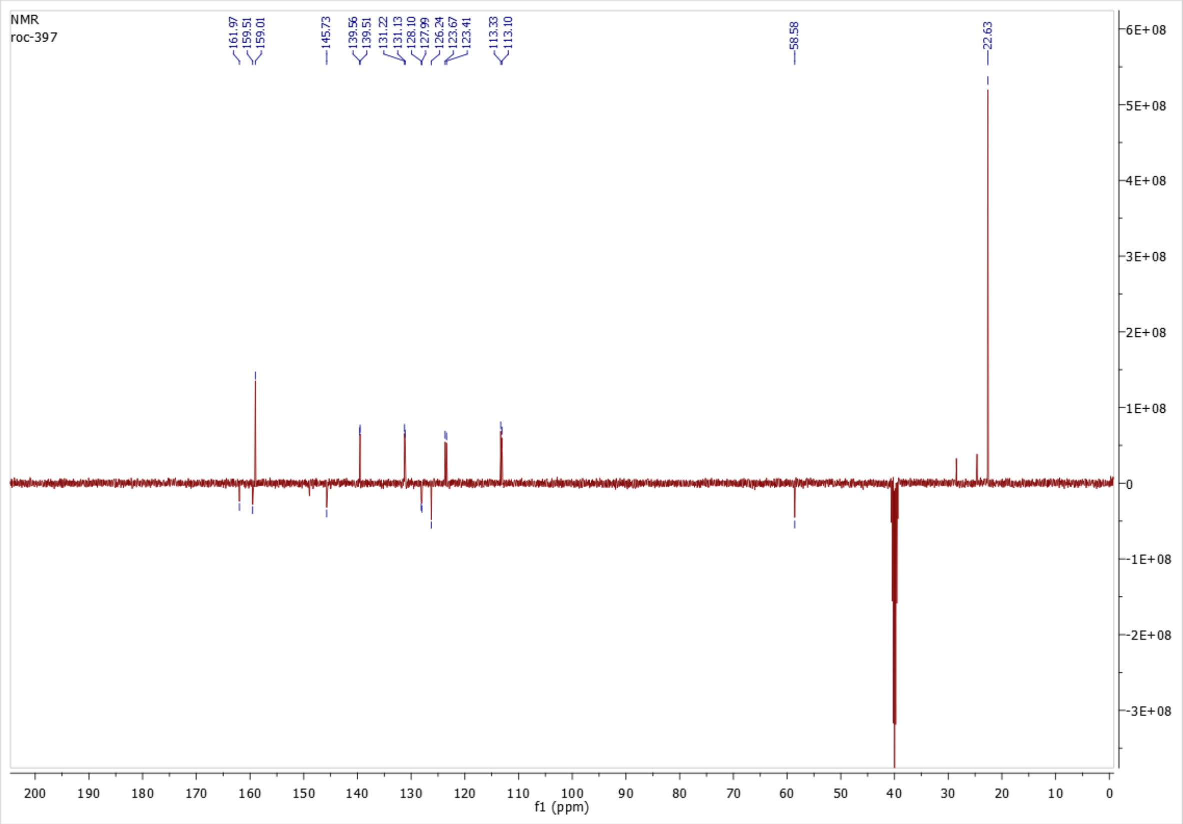

5.30. (*R*)-*N*-[(2-chloroquinolin-3-yl)methylene]-2-methylpropane-2-sulfinamide (**23c**)

^{1}H NMR spectrum of **23c**

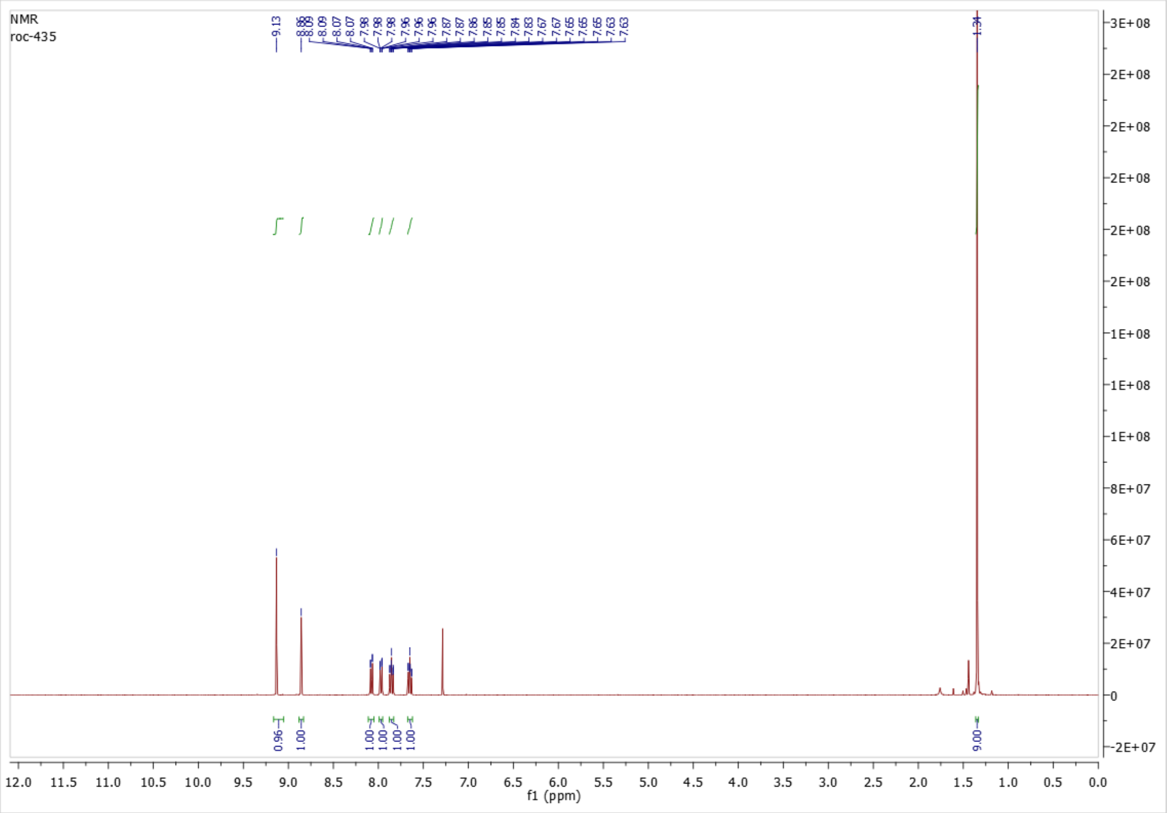

<u>^{13}C NMR spectrum of **23c**</u>

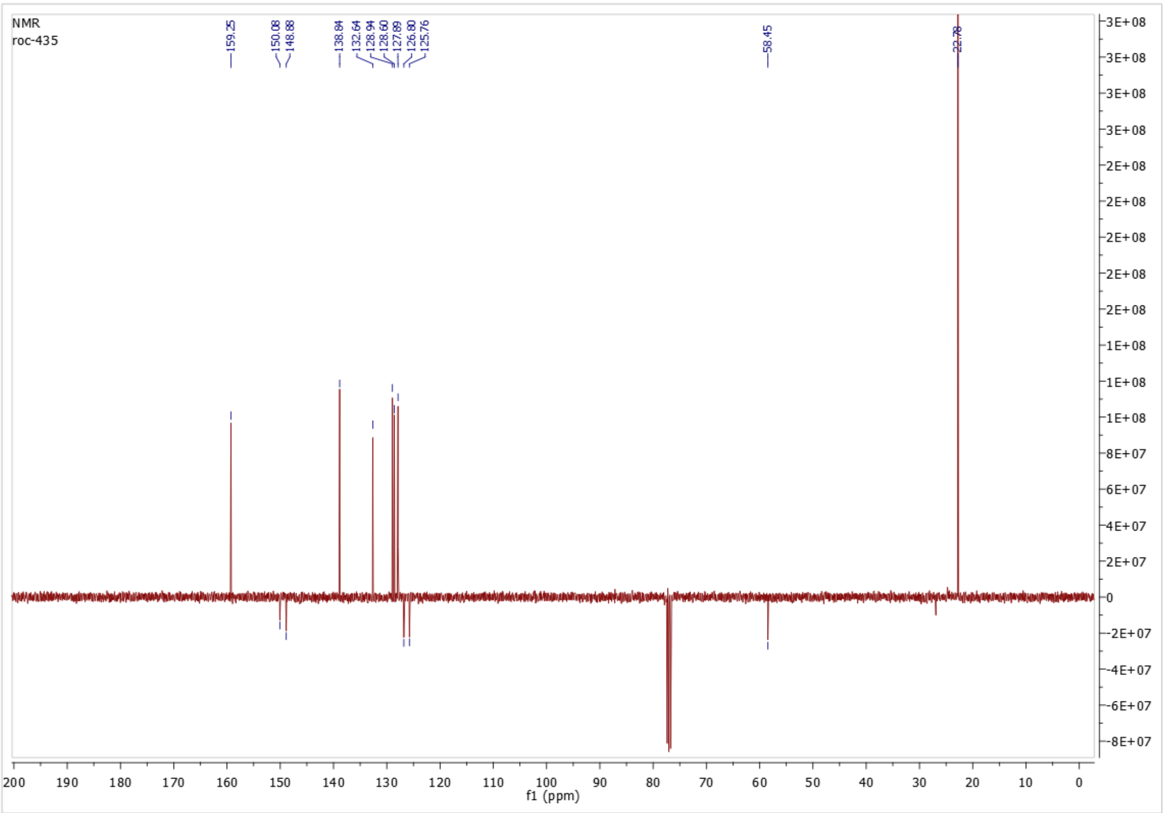

5.31. (*R*)-*N*-[(*S*)-1-(6-Bromo-2-chloroquinolin-3-yl)ethyl]-2-methylpropane-2-sulfinamide [(*S*,*R*)-**24a**]

<u>^{1}H NMR spectrum of (*S*,*R*)-**24a**</u>

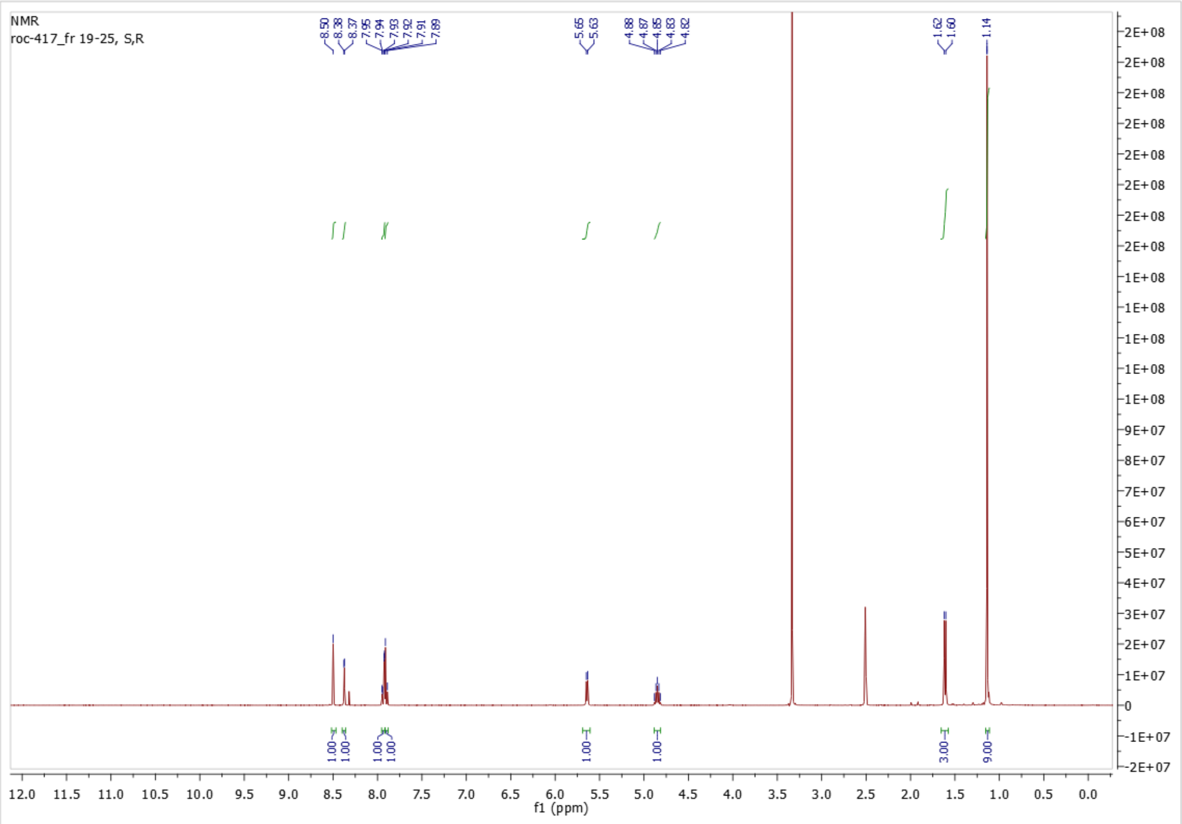

^{13}C NMR spectrum of (*S*,*R*)-**24a**

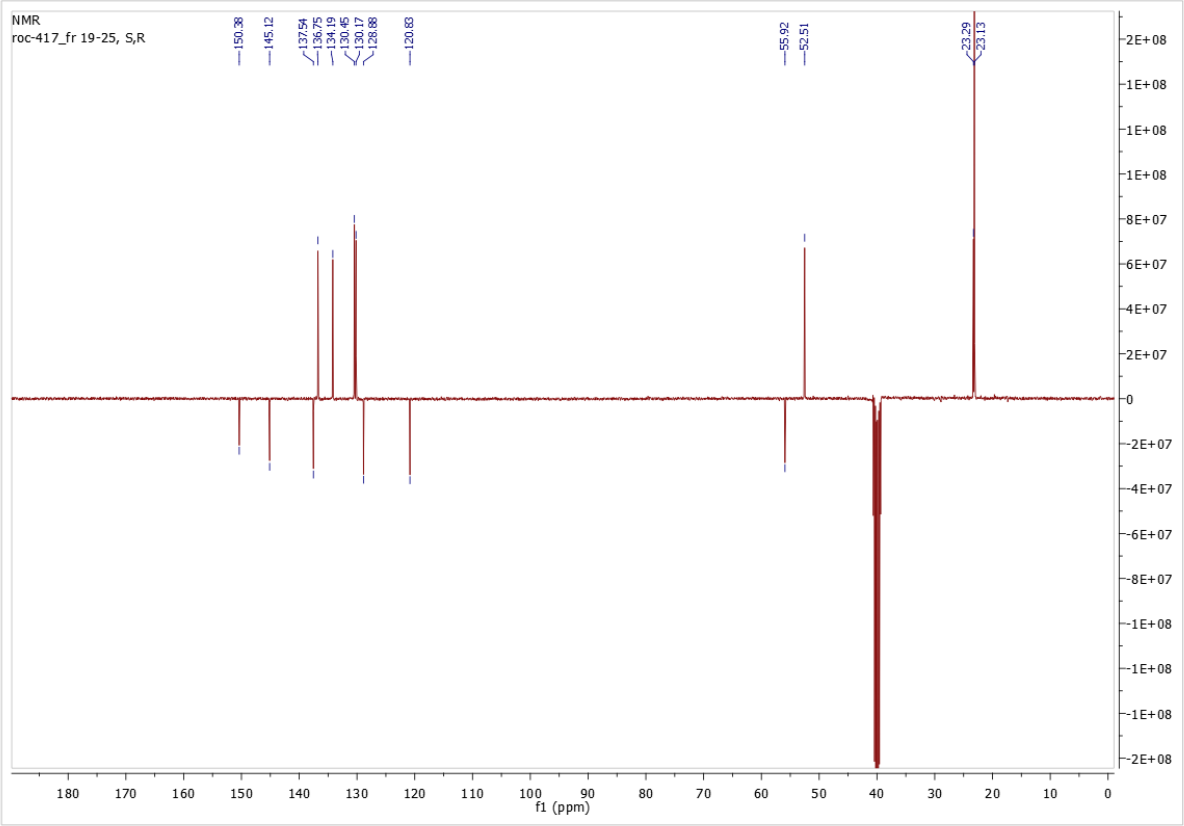

5.32. (*R*)-*N*-[(*R*)-1-(6-Bromo-2-chloroquinolin-3-yl)ethyl]-2-methylpropane-2-sulfinamide [(*R*,*R*)-**24a**]

^{1}H NMR spectrum of (*R*,*R*)-**24a**

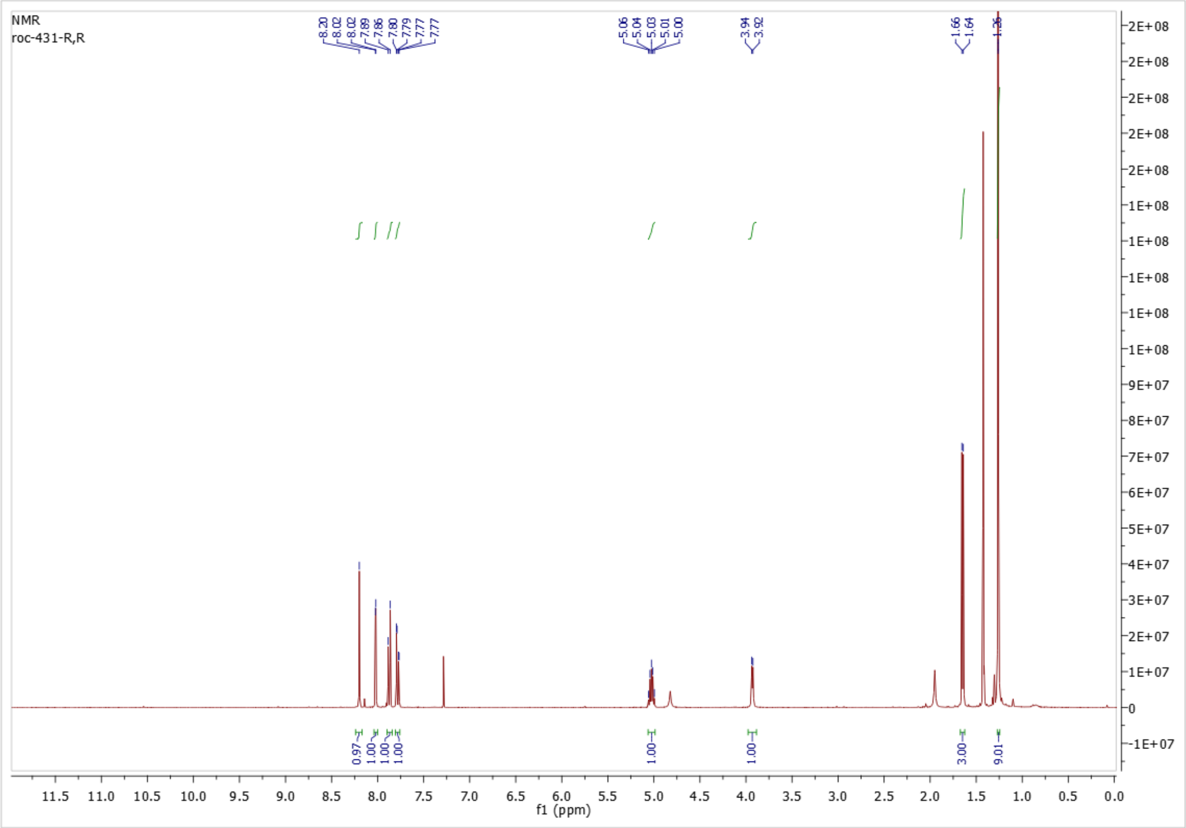

^{13}C NMR spectrum of (*R*,*R*)-**24a**

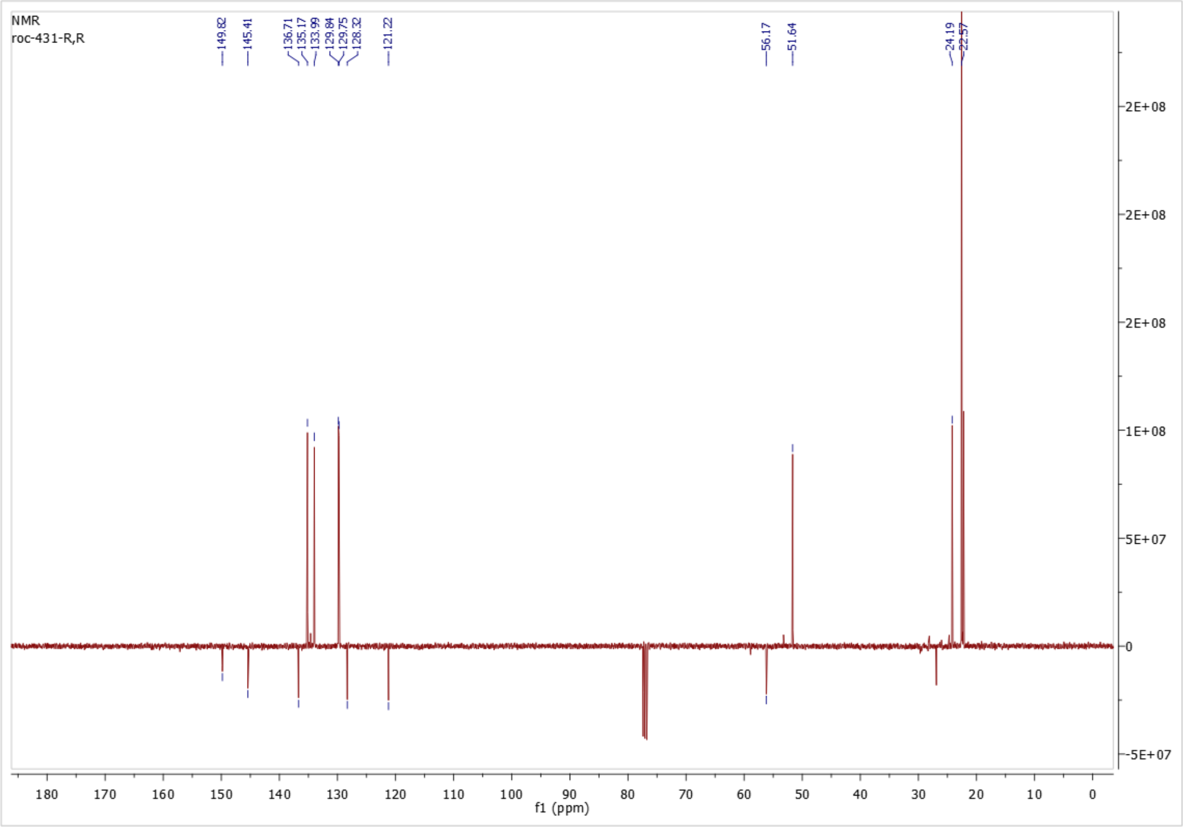

5.33. (*R*)-*N*-[(*S*)-1-(6-Fluoro-2-chloroquinolin-3-yl)ethyl]-2-methylpropane-2-sulfinamide [(*S*,*R*)-**24b**]

^{1}H NMR spectrum of (*S*,*R*)-**24b**

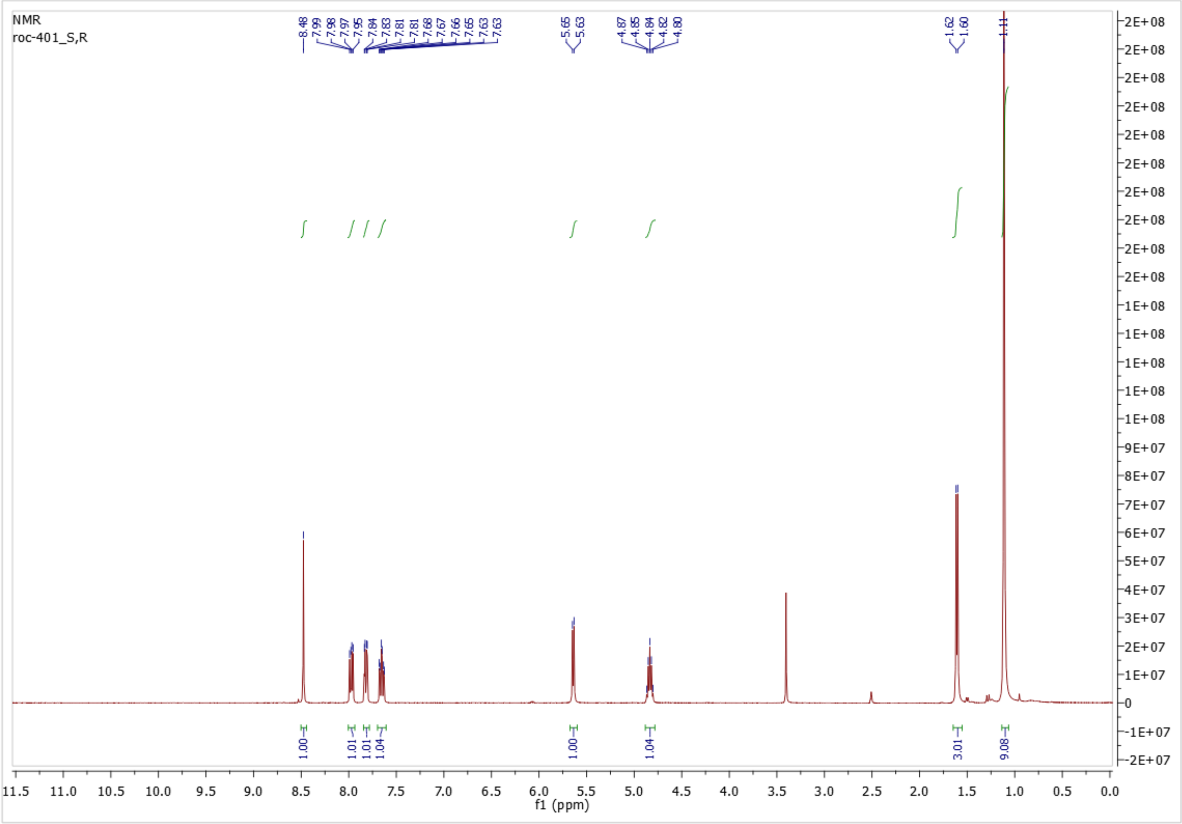

^{13}C NMR spectrum of (*S*,*R*)-**24b**

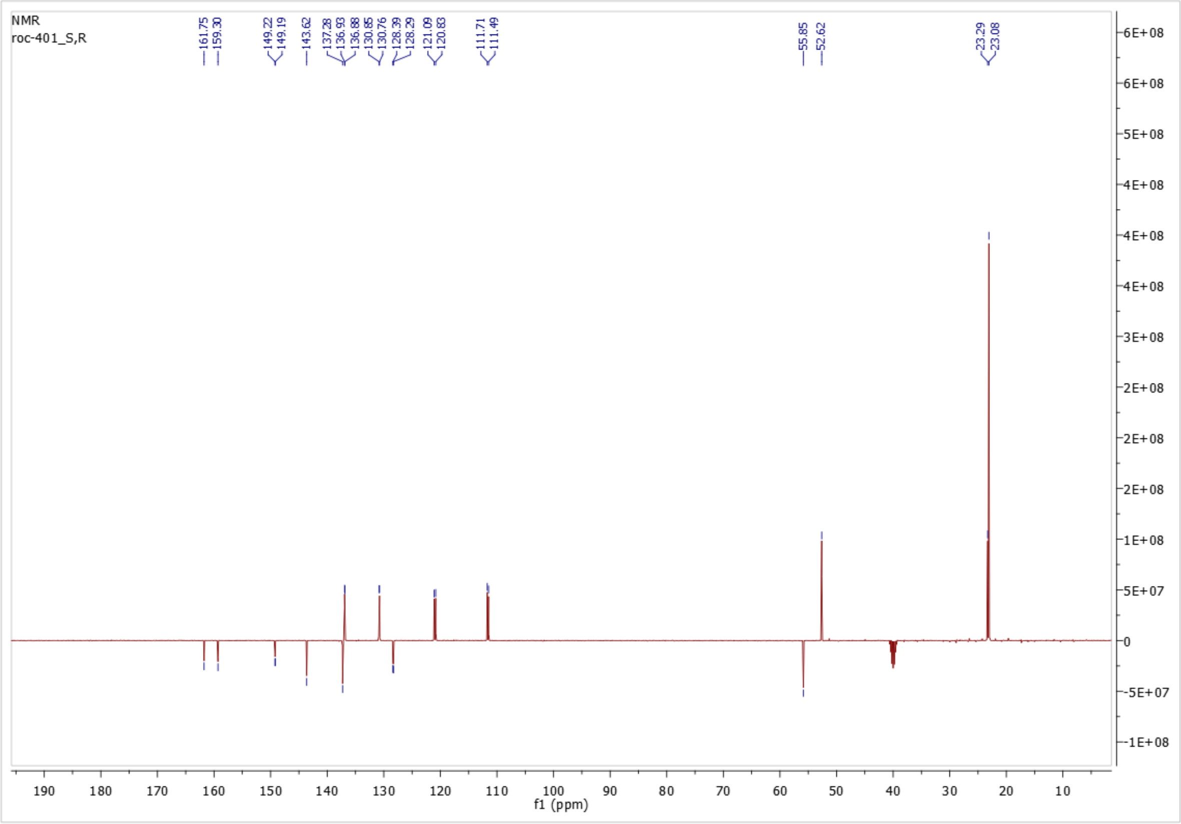

^{19}F NMR spectrum of (*S*,*R*)-**24b**

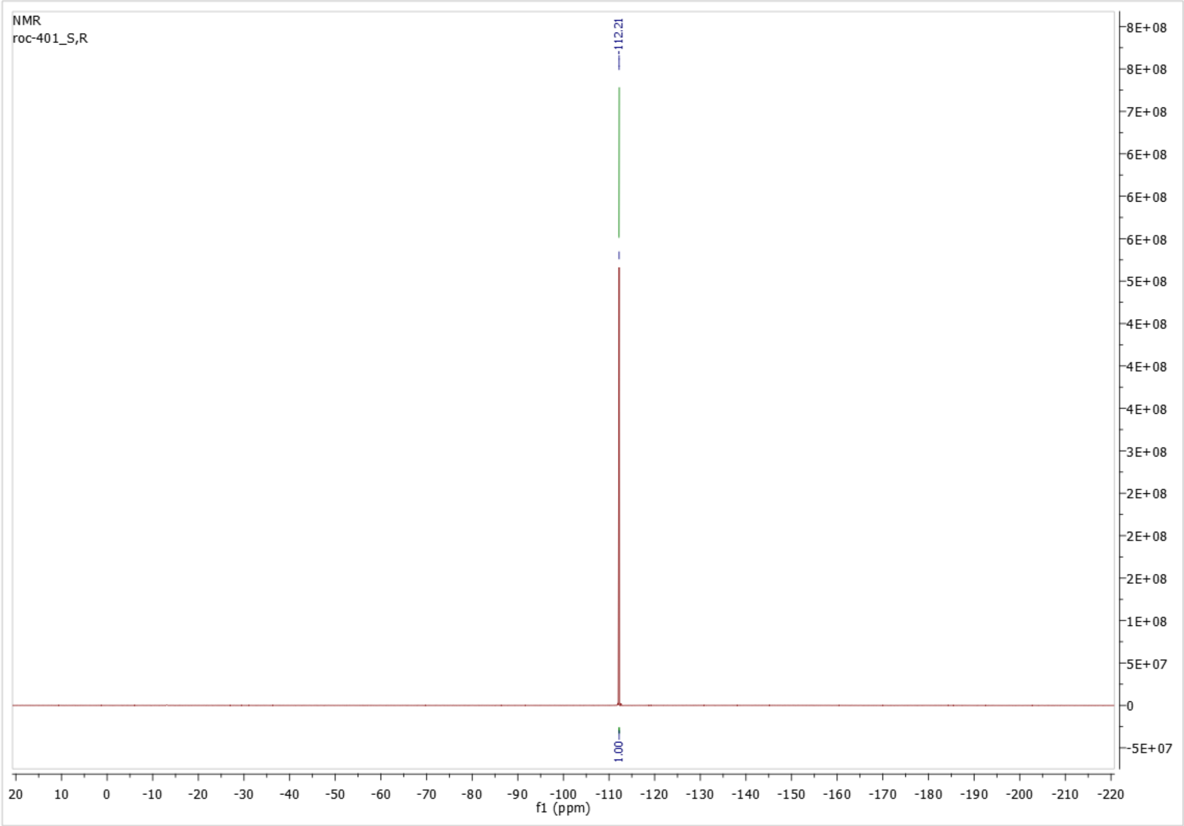

5.34. (*R*)-*N*-[(*R*)-1-(6-Fluoro-2-chloroquinolin-3-yl)ethyl]-2-methylpropane-2-sulfinamide [(*R*,*R*)-**24b**]

^{1}H NMR spectrum of (*R*,*R*)-**24b**

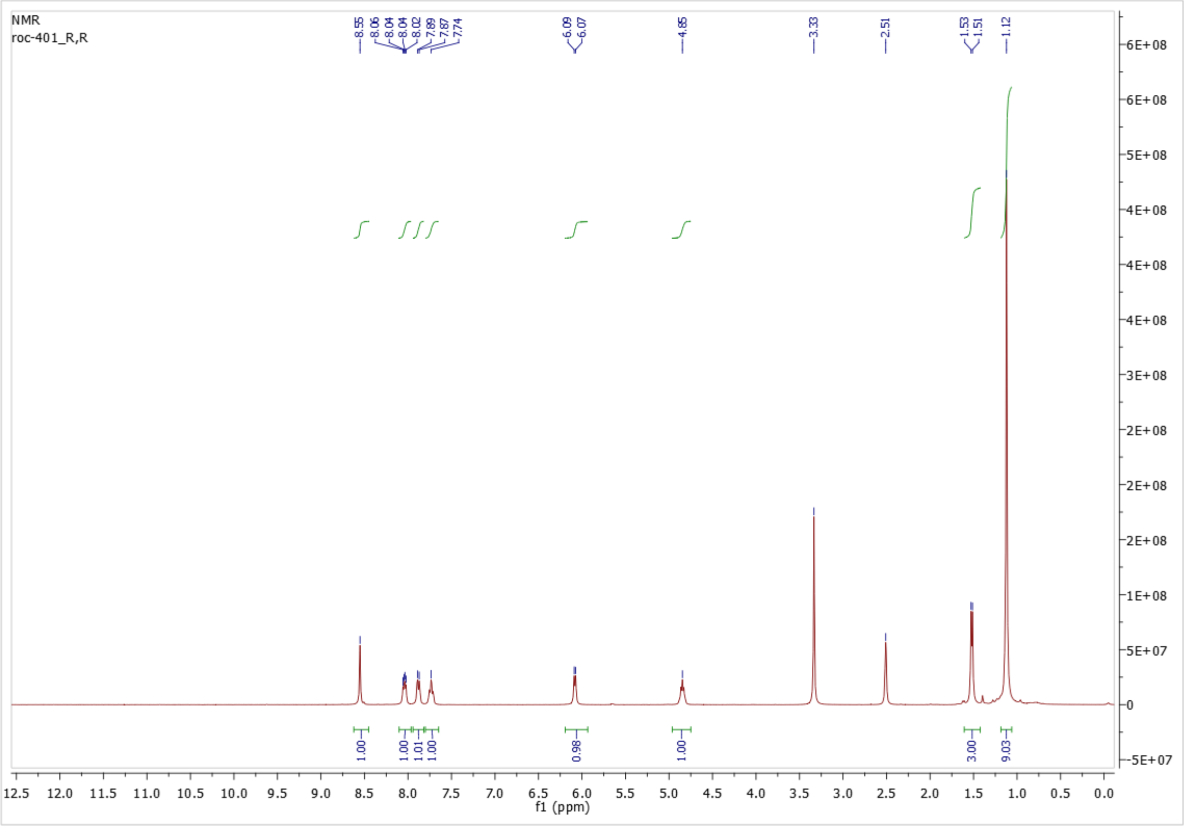

^{13}C NMR spectrum of (*R*,*R*)-**24b**

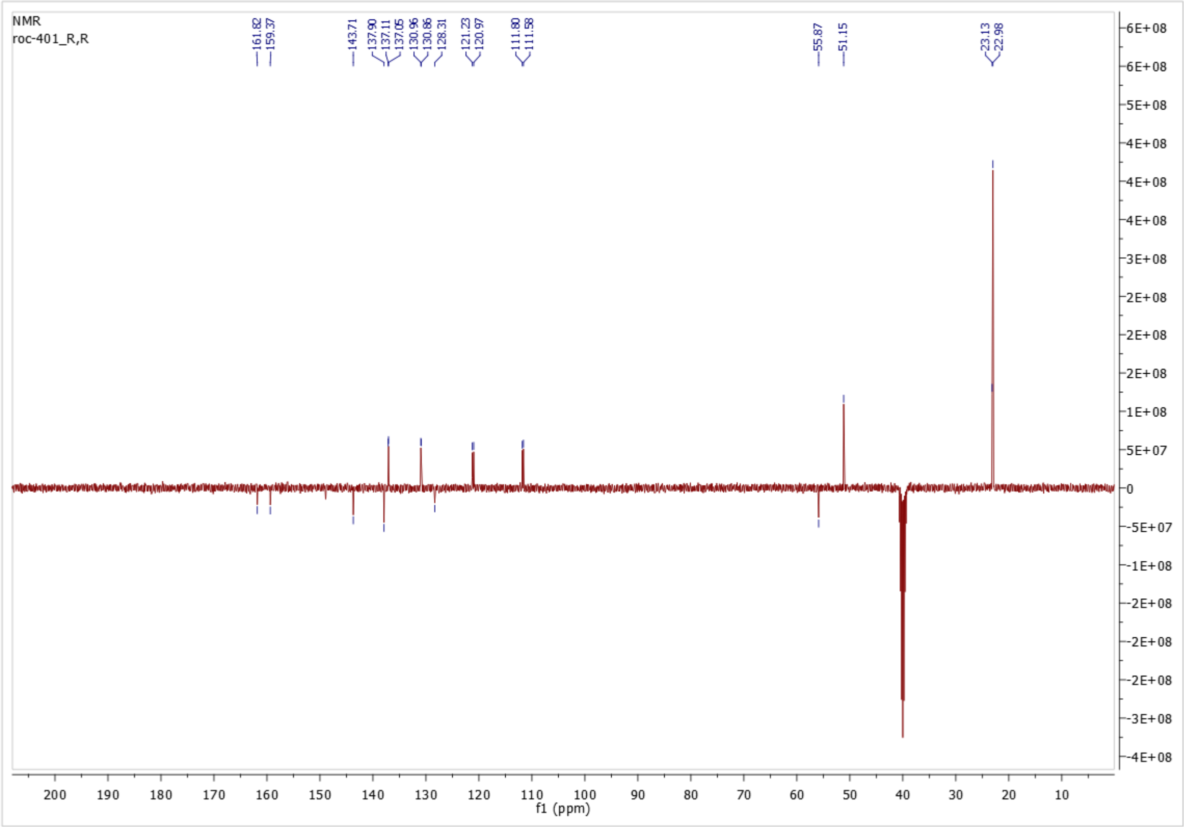

^{19}F NMR spectrum of (*R*,*R*)-**24b**

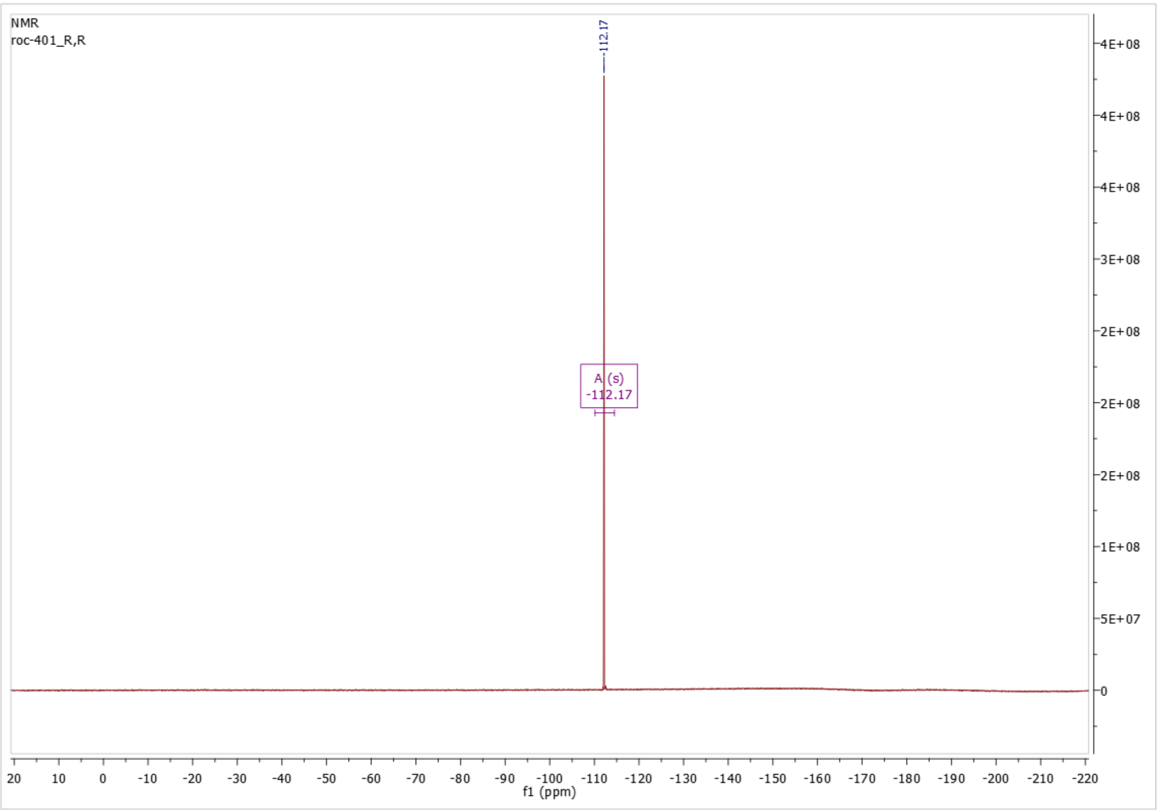

5.35. (*R*)-*N*-[(*S*)-1-(2-Chloroquinolin-3-yl)ethyl]-2-methylpropane-2-sulfinamide [(*S*,*R*)-**24c**]

^{1}H NMR spectrum of (*S*,*R*)-**24c**

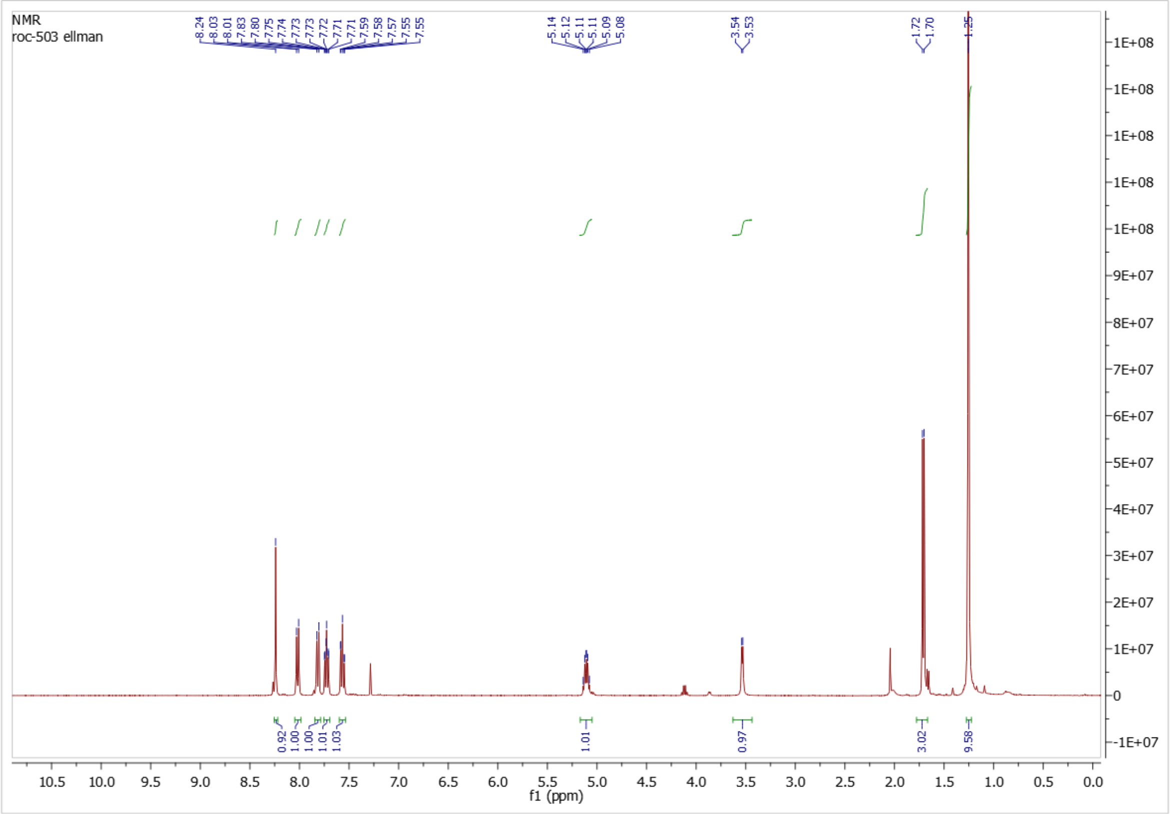

^{13}C NMR spectrum of (*S*,*R*)-**24c**

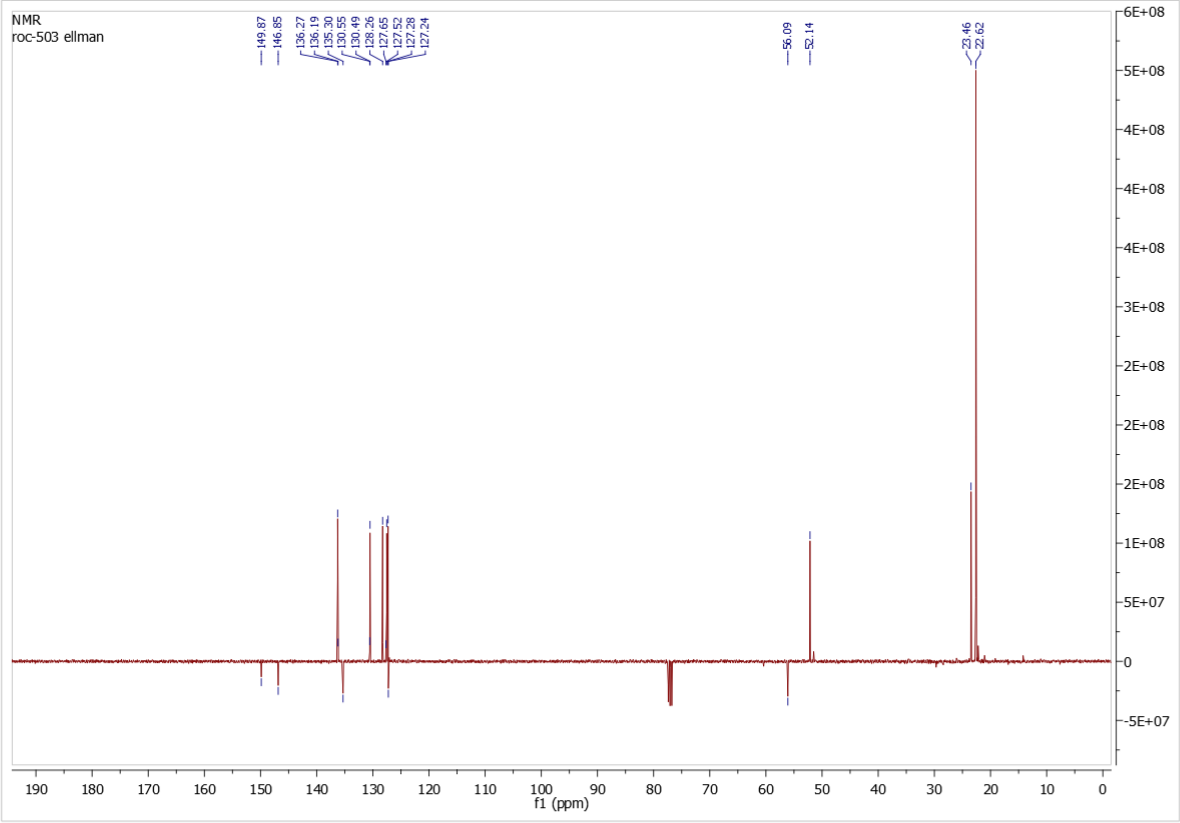

5.36. (*R*)-*N*-[(*R*)-1-(2-Chloroquinolin-3-yl)ethyl]-2-methylpropane-2-sulfinamide [(*R*,*R*)-**24c**]

^{1}H NMR spectrum of (*R*,*R*)-**24c**

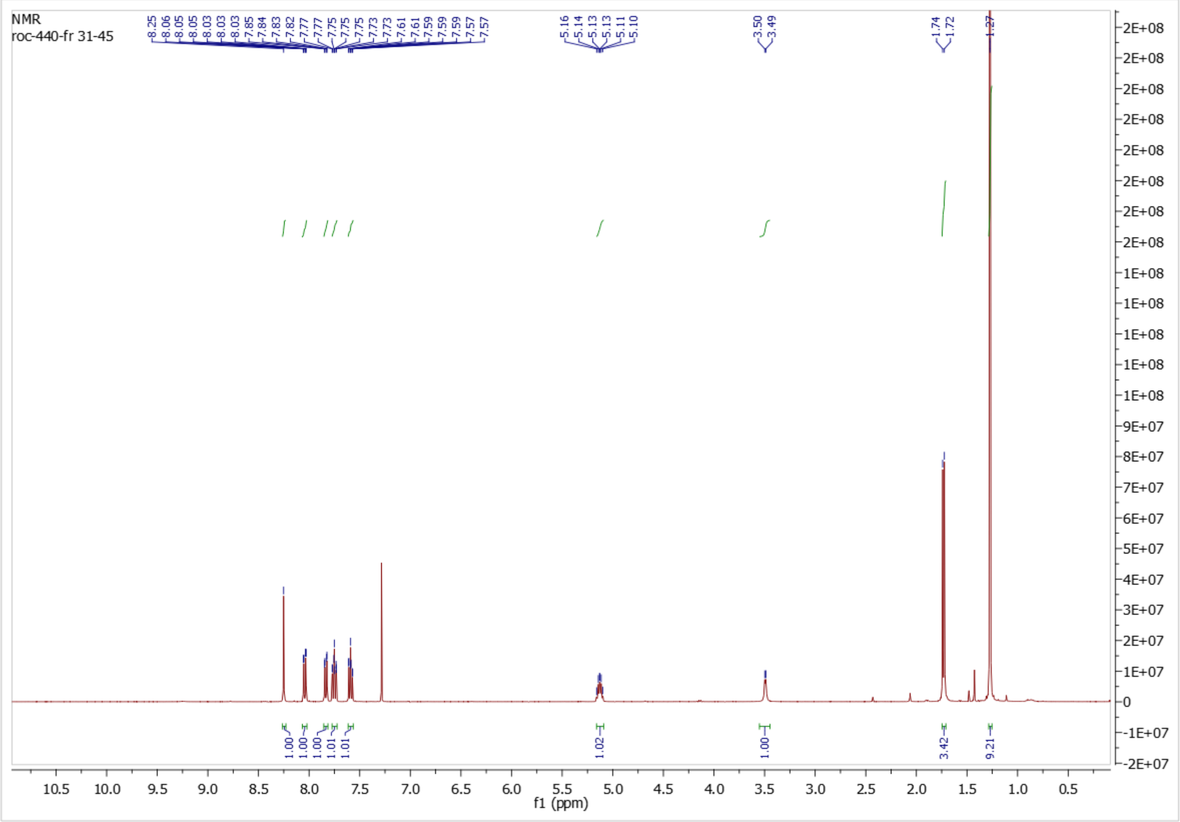

^{13}C NMR spectrum of (*R*,*R*)-**24c**

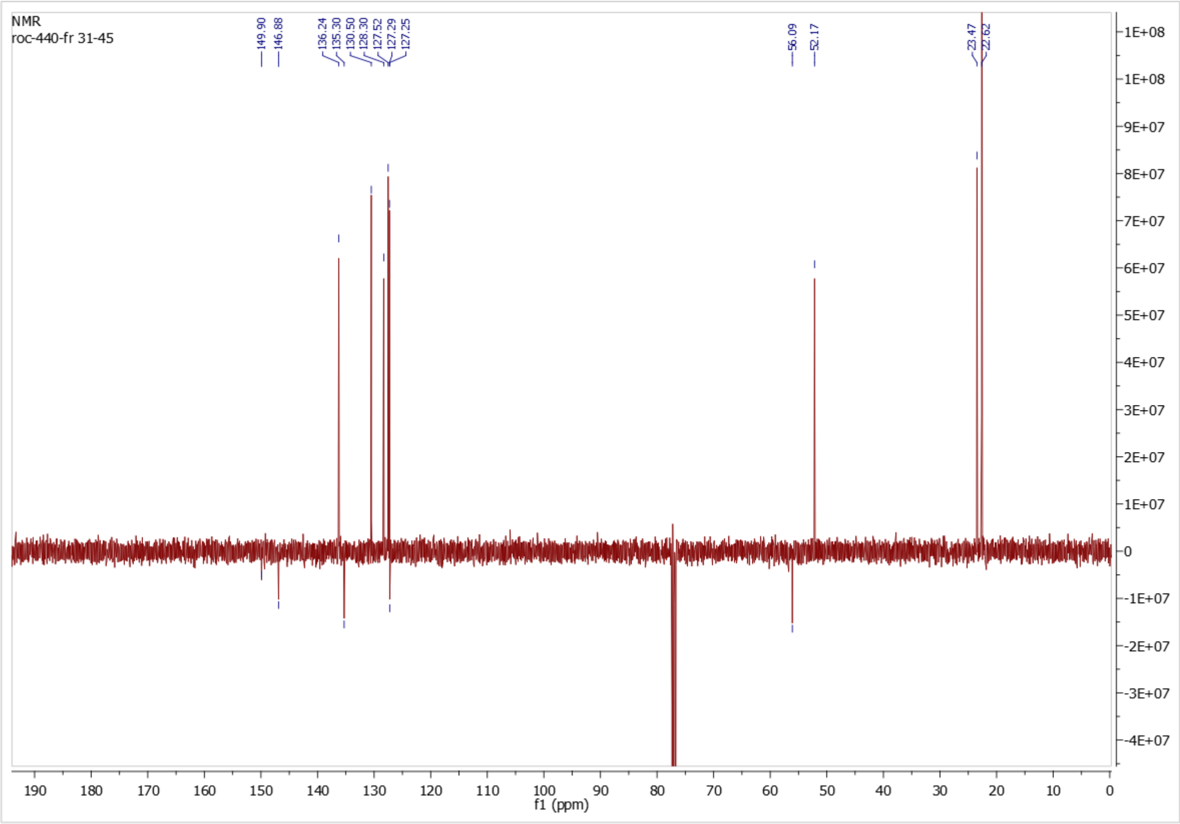

5.37. (*S*)-5-[{1-(6-Fluoro-2-oxo-1,2-dihydroquinoline-3-yl)ethyl}amino]-1-methyl-6-oxo-1,6-dihydropyridine-2-carbonitrile [(*S*)-**2**]

^{1}H NMR spectrum of (*S*)-**2**

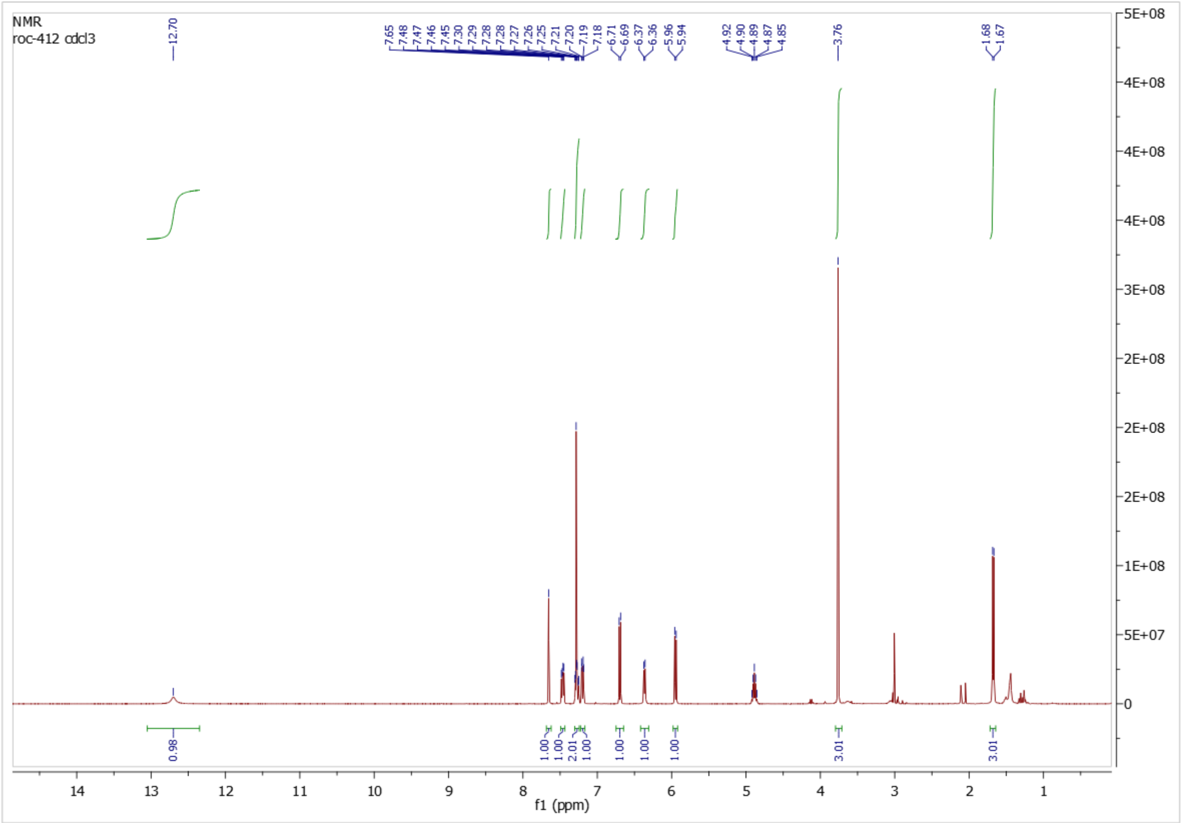

^{13}C NMR spectrum of (*S*)-**2**

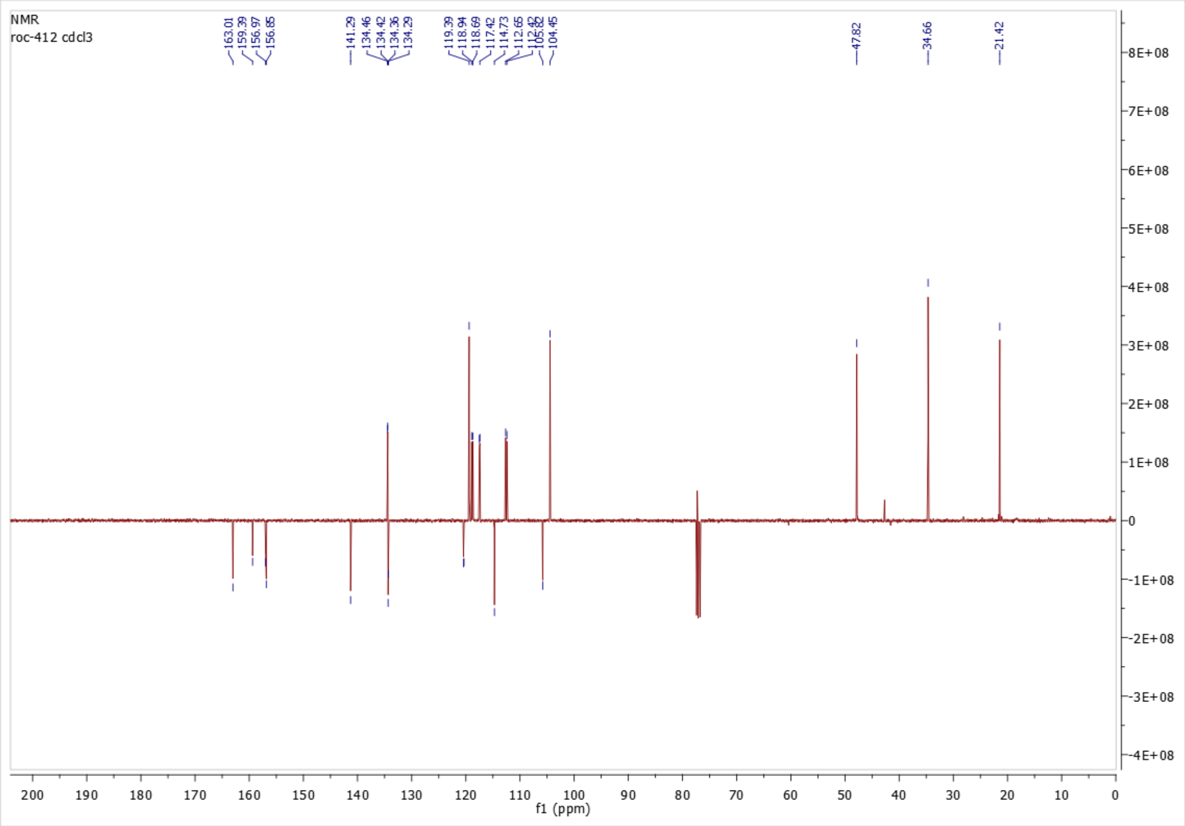

^{19}F NMR spectrum of (*S*)-**2**

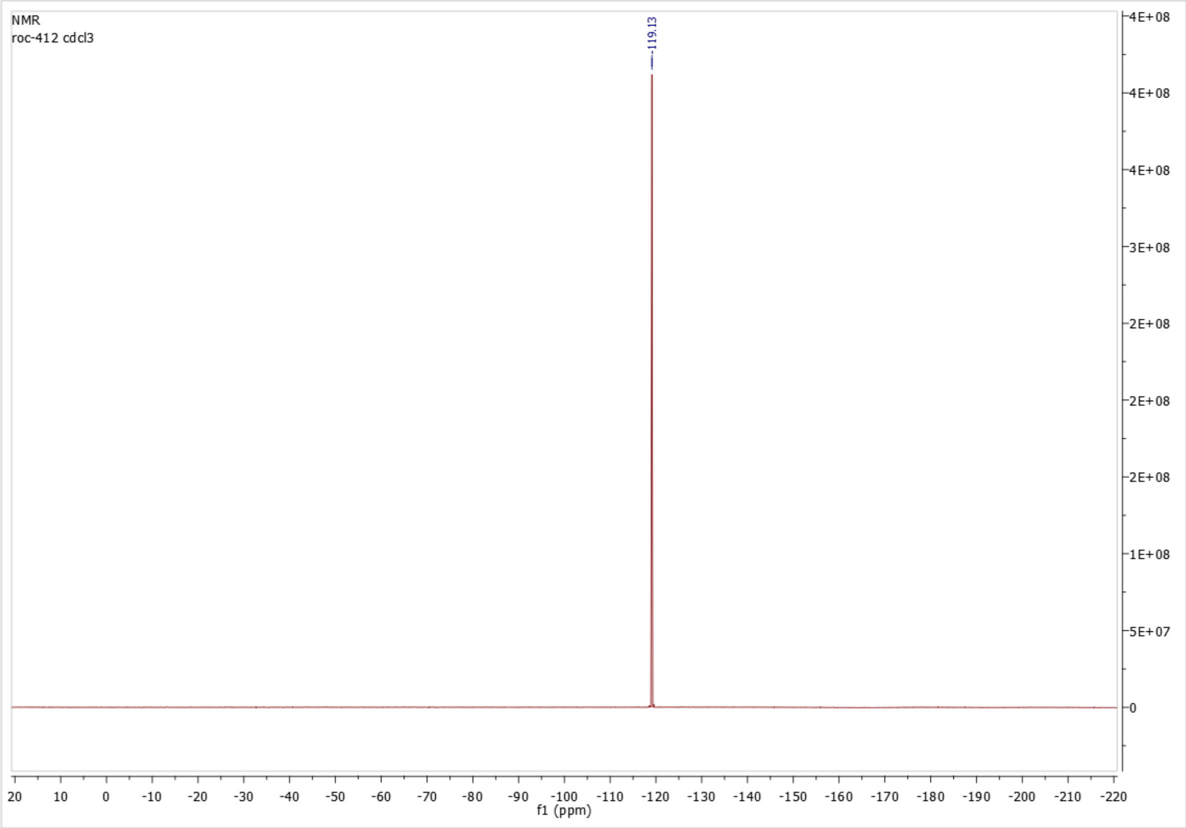

5.38. (*R*)-5-[{1-(6-Fluoro-2-oxo-1,2-dihydroquinoline-3-yl)ethyl}amino]-1-methyl-6-oxo-1,6-dihydropyridine-2-carbonitrile [(*R*)-**2**]

<u>^{1}H NMR spectrum of (*R*)-**2**</u>

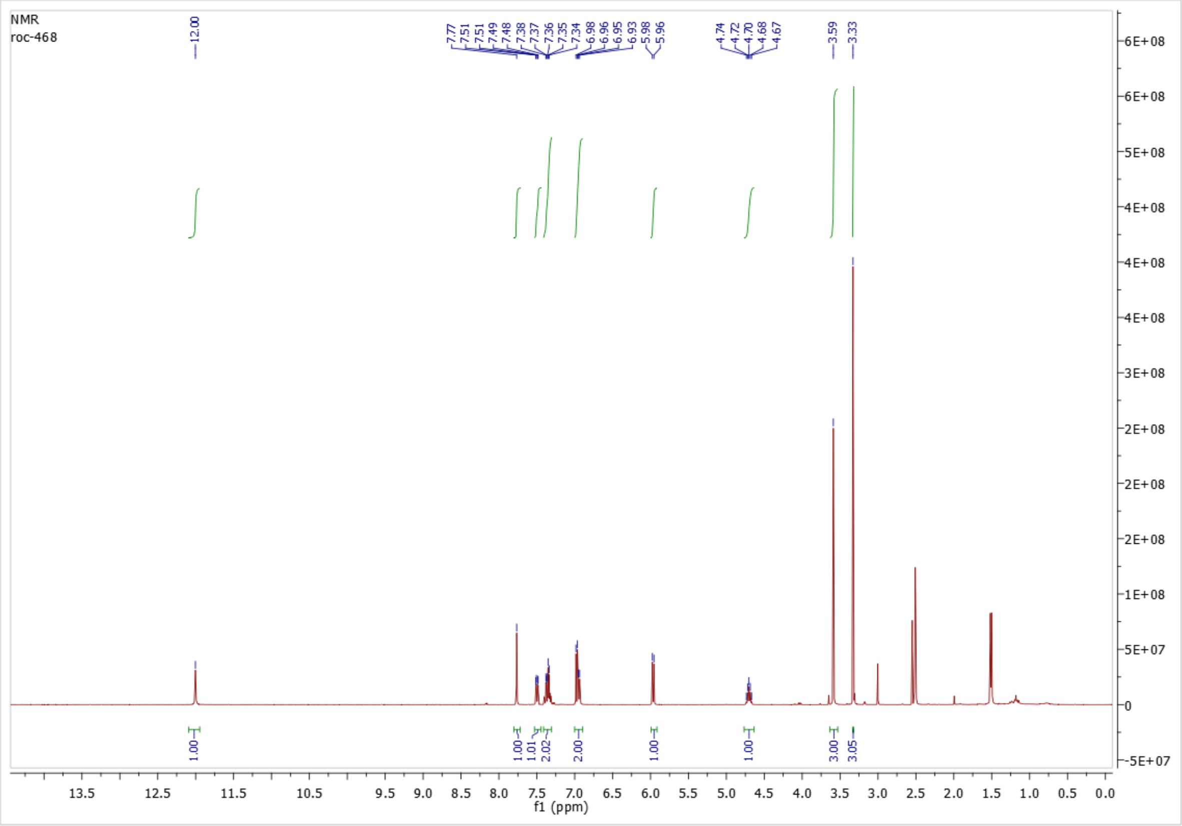

<u>^{13}C NMR spectrum of (*R*)-**2**</u>

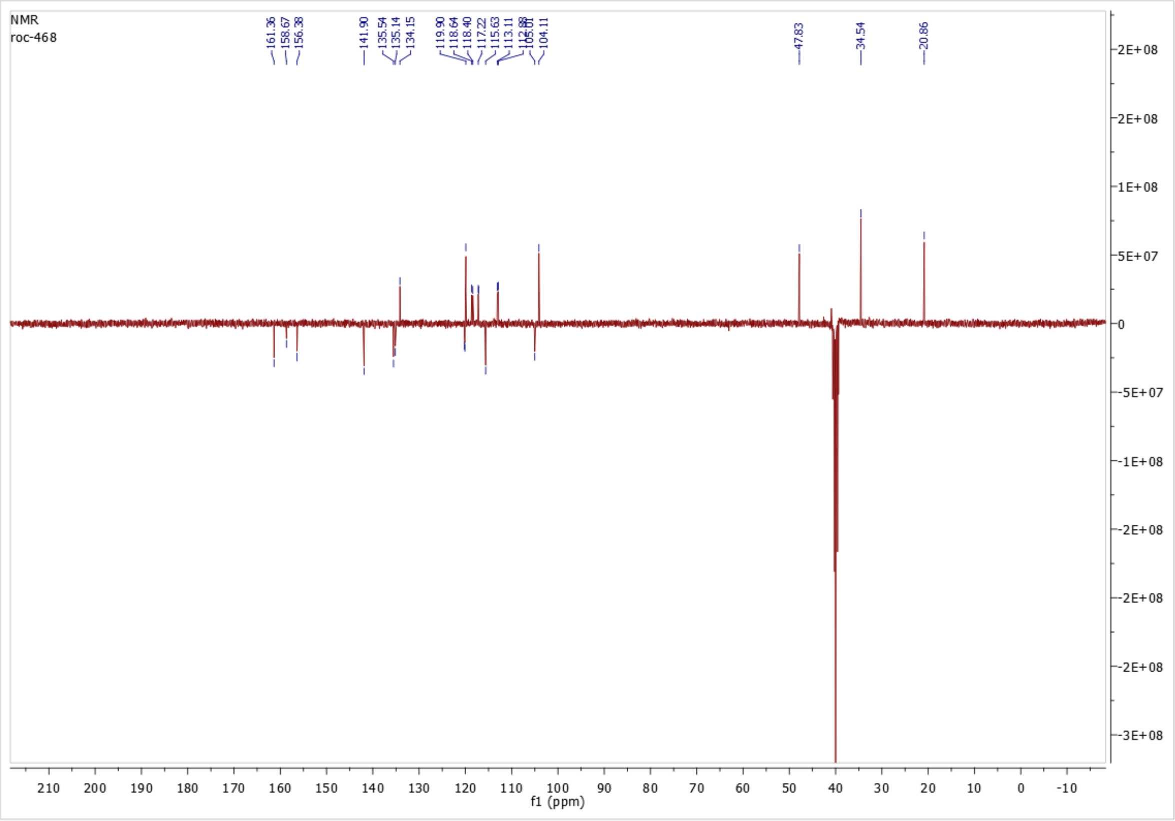

^{19}F NMR spectrum of (*R*)-**2**

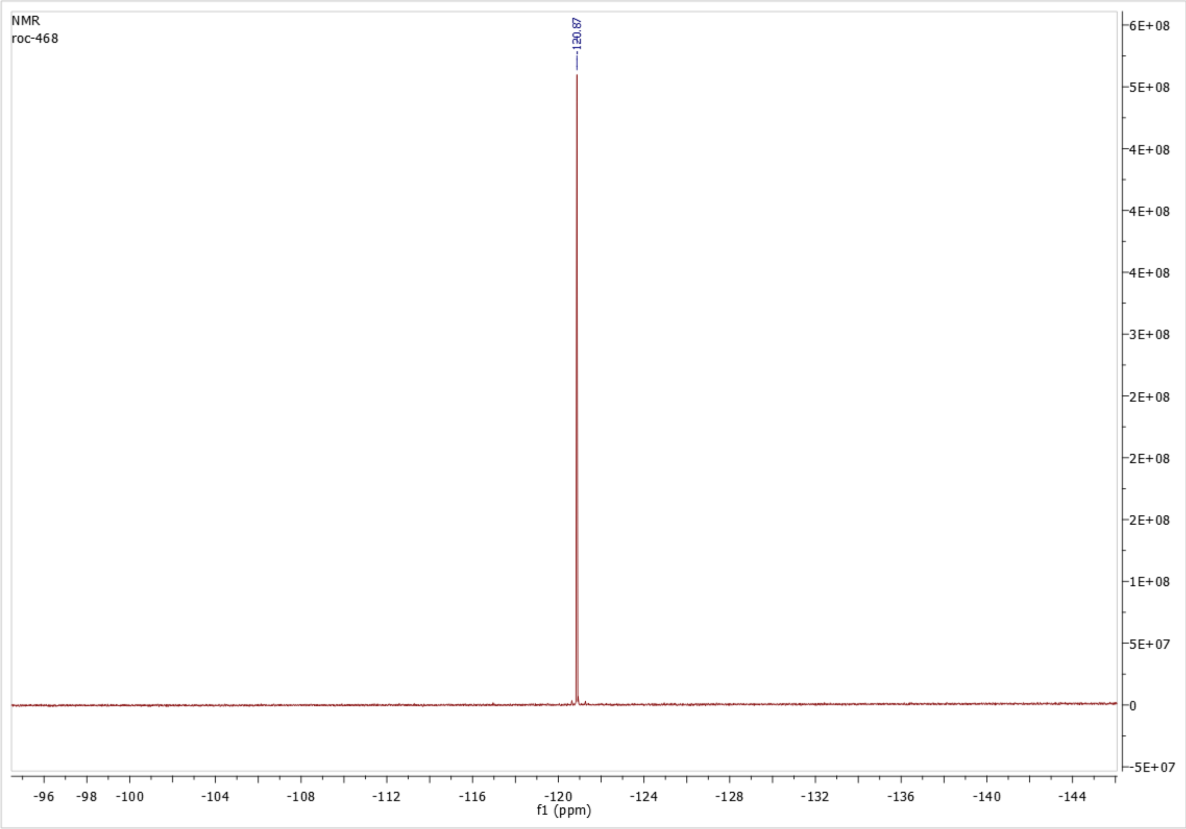

5.39. (*S*)-5-[{1-(6-Bromo-2-oxo-1,2-dihydroquinoline-3-yl)ethyl}amino]-1-methyl-6-oxo-1,6-dihydropyridine-2-carbonitrile [(*S*)-**26a**]

^{1}H NMR spectrum of (*S*)-**26a**

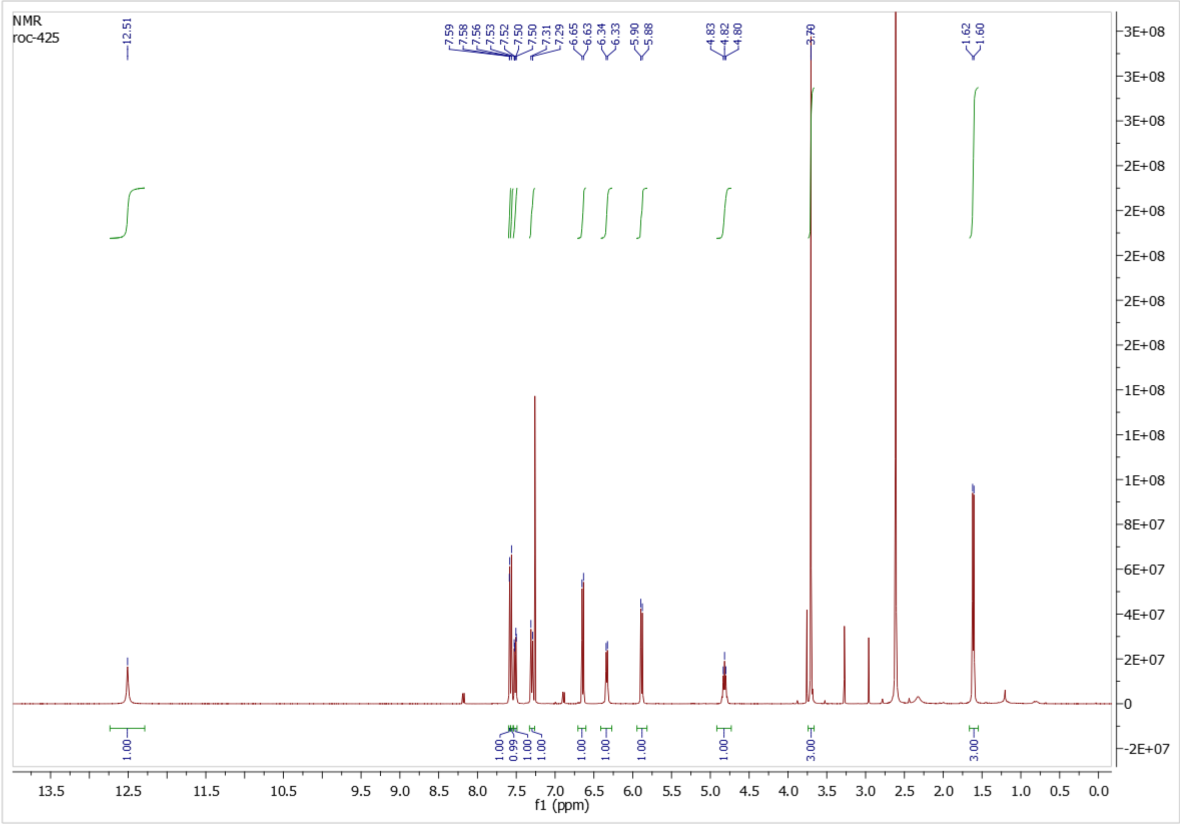

^{13}C NMR spectrum of (*S*)-**26a**

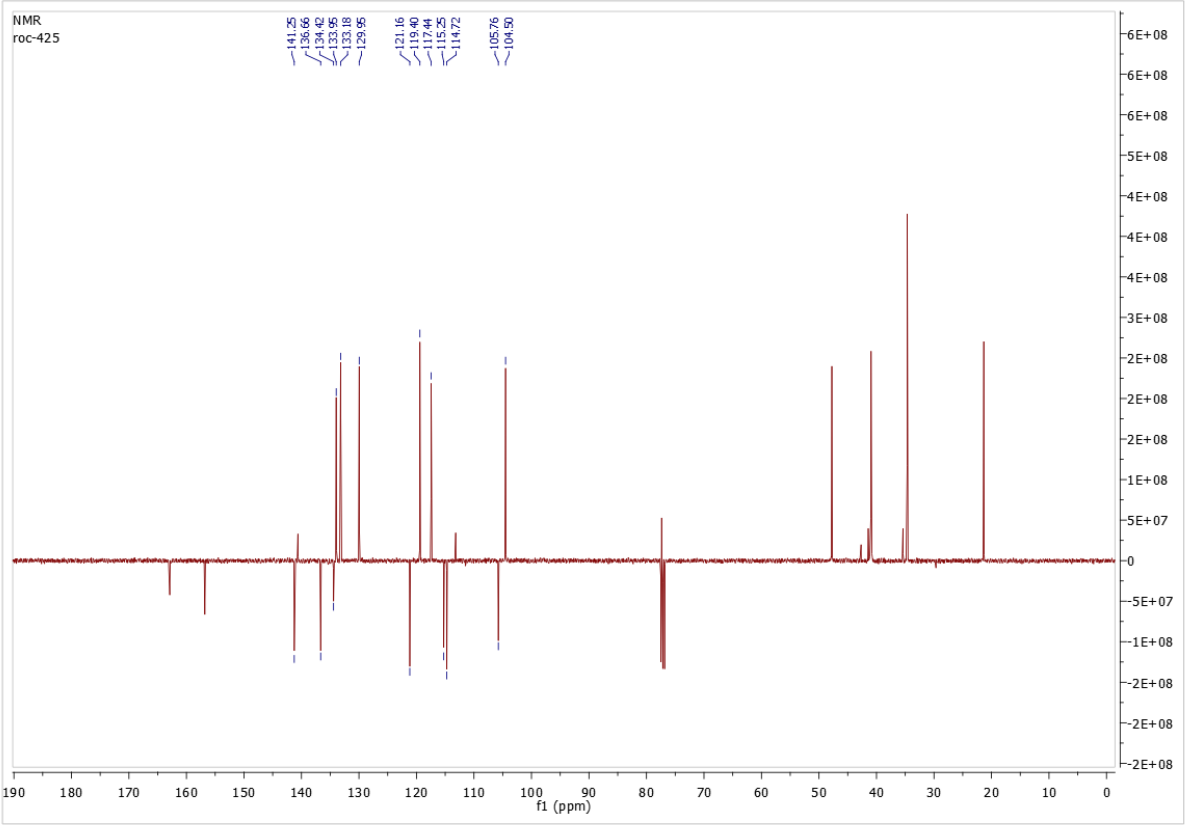

5.40. (*R*)-5-[{1-(6-Bromo-2-oxo-1,2-dihydroquinoline-3-yl)ethyl}amino]-1-methyl-6-oxo-1,6-dihydropyridine-2-carbonitrile [(*R*)-**26a**]

^{1}H NMR spectrum of (*R*)-**26a**

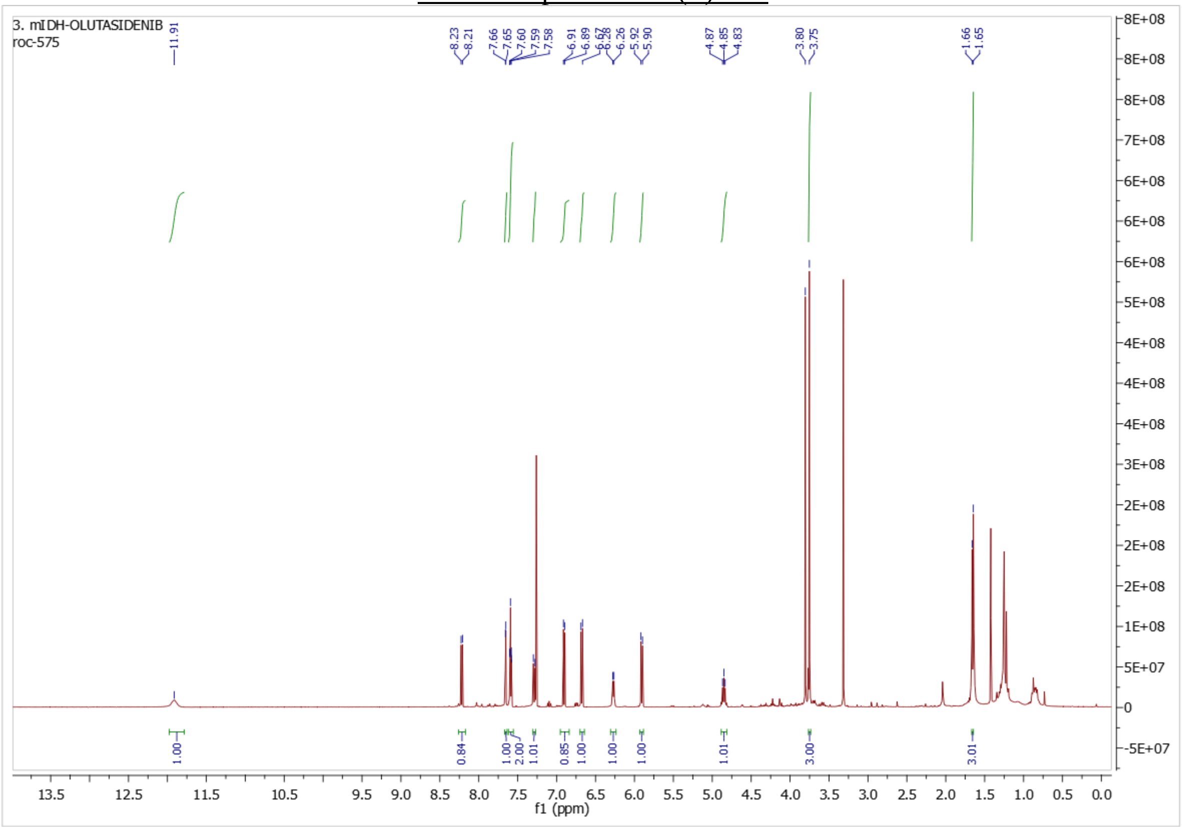

5.41. (*S*)-5-[{1-(2-Oxo-1,2-dihydroquinoline-3-yl)ethyl}amino]-1-methyl-6-oxo-1,6-dihydropyridine-2-carbonitrile [(*S*)-**26c**]

^{1}H NMR spectrum of (*S*)-**26c**

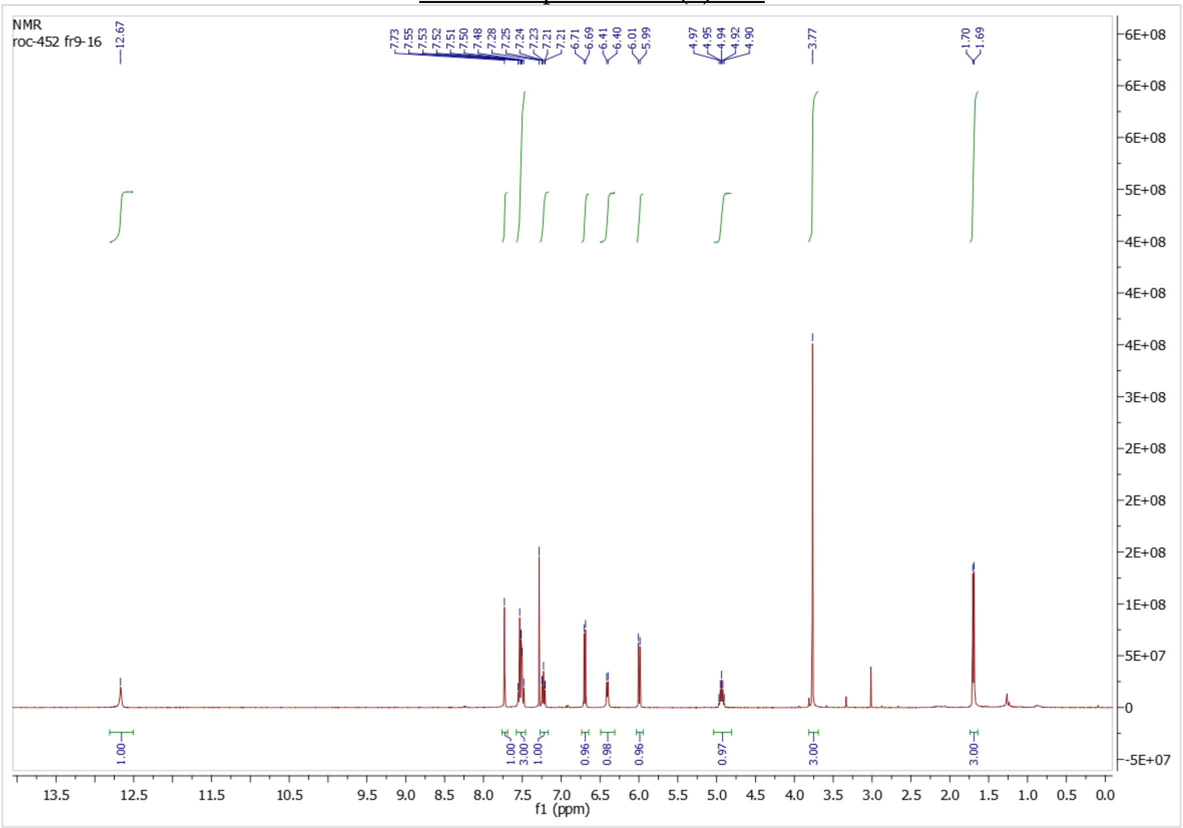

^{13}C NMR spectrum of (*S*)-**26c**

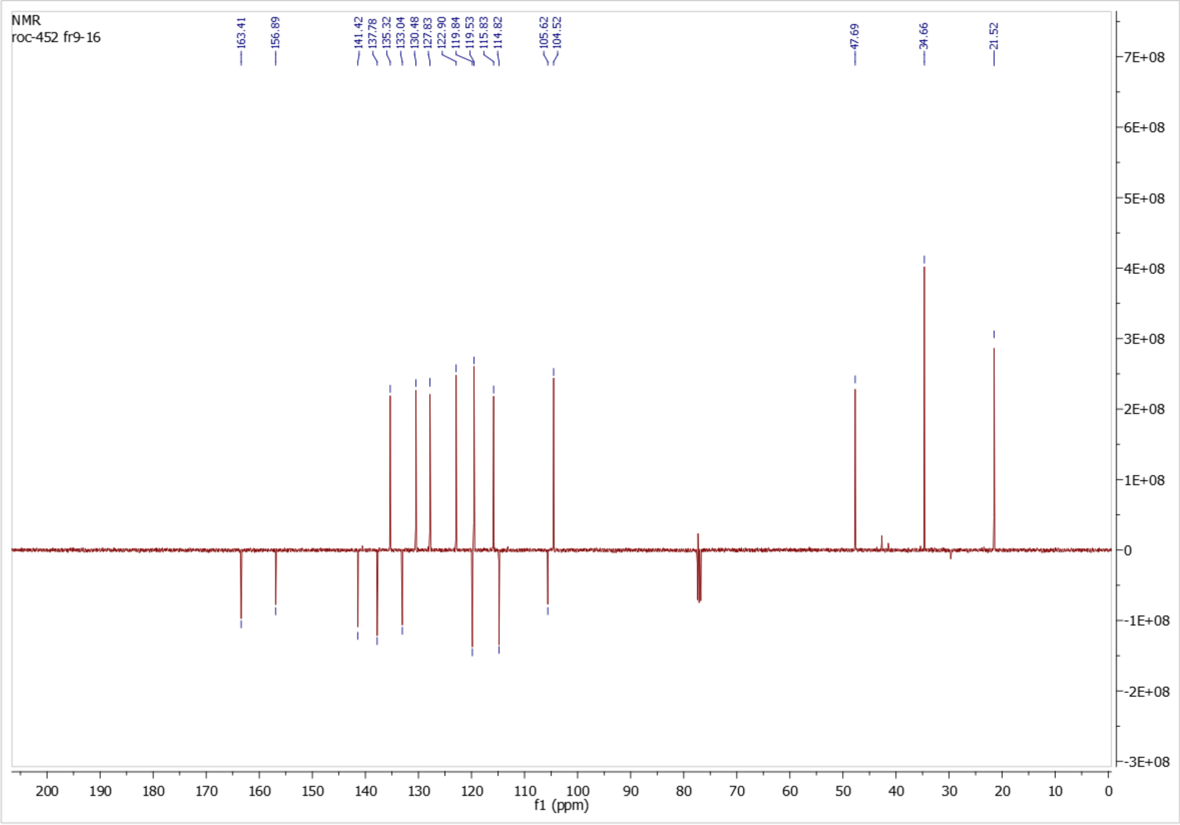

5.42. (*R*)-5-[{1-(2-Oxo-1,2-dihydroquinoline-3-yl)ethyl}amino]-1-methyl-6-oxo-1,6-dihydropyridine-2-carbonitrile [(*R*)-**26c**]

^{1}H NMR spectrum of (*R*)-**26c**

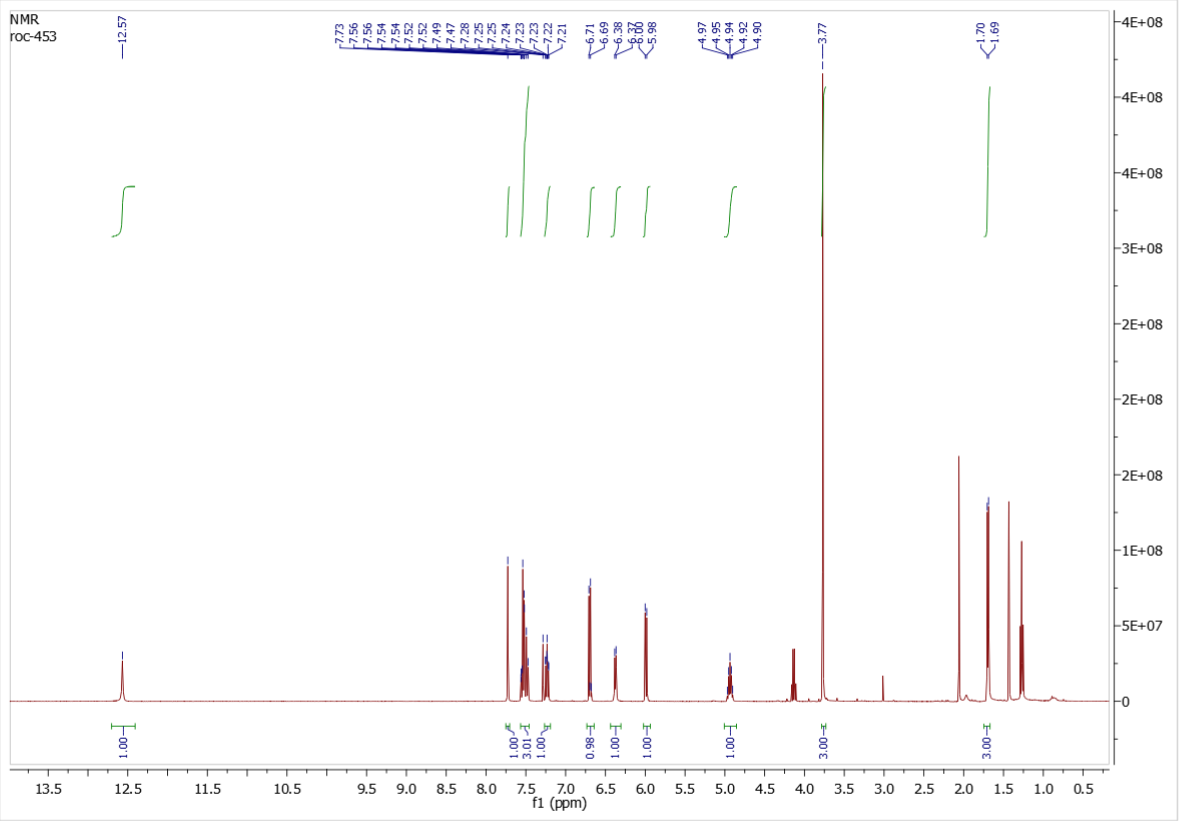

^{13}C NMR spectrum of (*R*)-**26c**

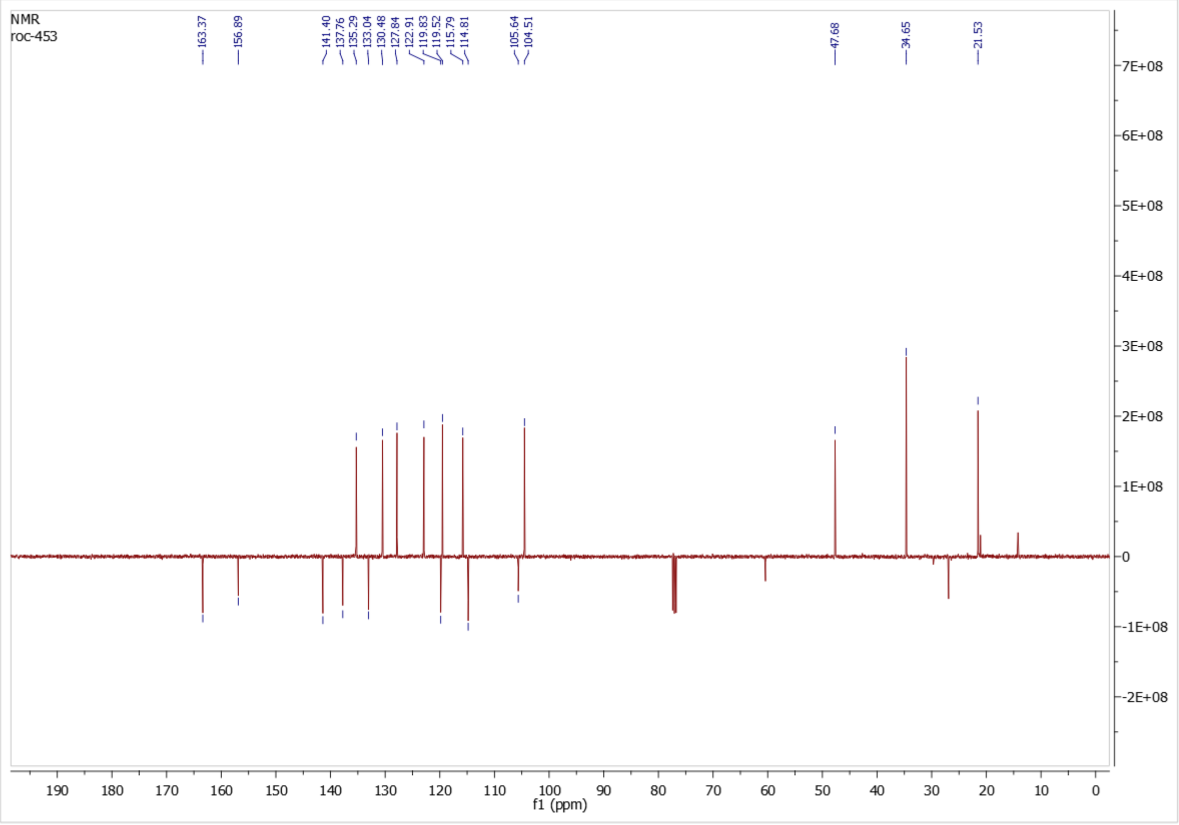

5.43. (*S*)-((6-Bromo-3-(1-((6-cyano-1-methyl-2-oxo-1,2-dihydropyridin-3-yl)amino)ethyl)quinolin-2-yl)oxy)methyl pivalate [(*S*)-**27a**] and (*S*)-(6-bromo-3-(1-((6-cyano-1-methyl-2-oxo-1,2-dihydropyridin-3-yl)amino)ethyl)-2-oxoquinolin-1(2*H*)-yl)methyl pivalate [(*S*)-**28a**]

^{1}H NMR spectrum of the mixture of (*S*)-**27a** and (*S*)-**28a**

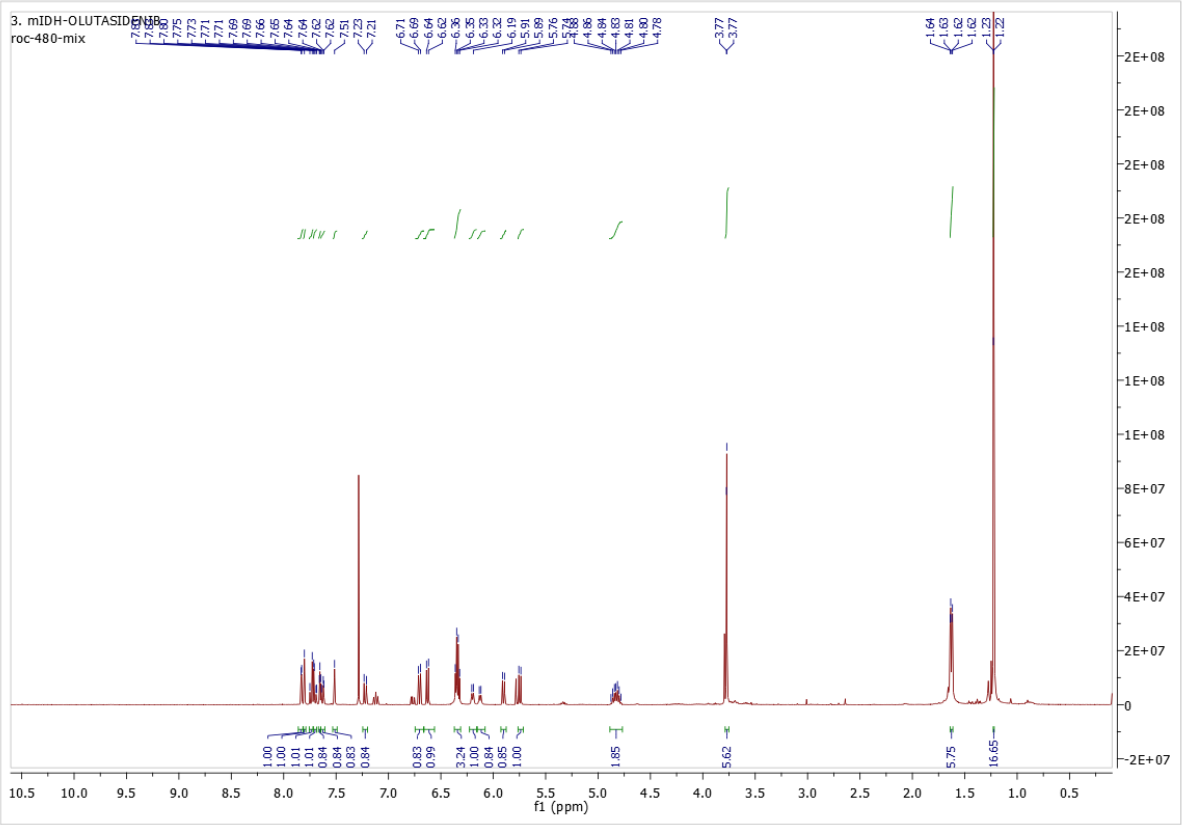

5.44. (*R*)-((6-Bromo-3-(1-((6-cyano-1-methyl-2-oxo-1,2-dihydropyridin-3-yl)amino)ethyl)quinolin-2-yl)oxy)methyl pivalate [(*R*)-**27a**] and (*R*)-(6-bromo-3-(1-((6-cyano-1-methyl-2-oxo-1,2-dihydropyridin-3-yl)amino)ethyl)-2-oxoquinolin-1(2*H*)-yl)methyl pivalate [(*R*)-**28a**]

^{1}H NMR spectrum of the mixture of (*R*)-**27a** and (*R*)-**28a**

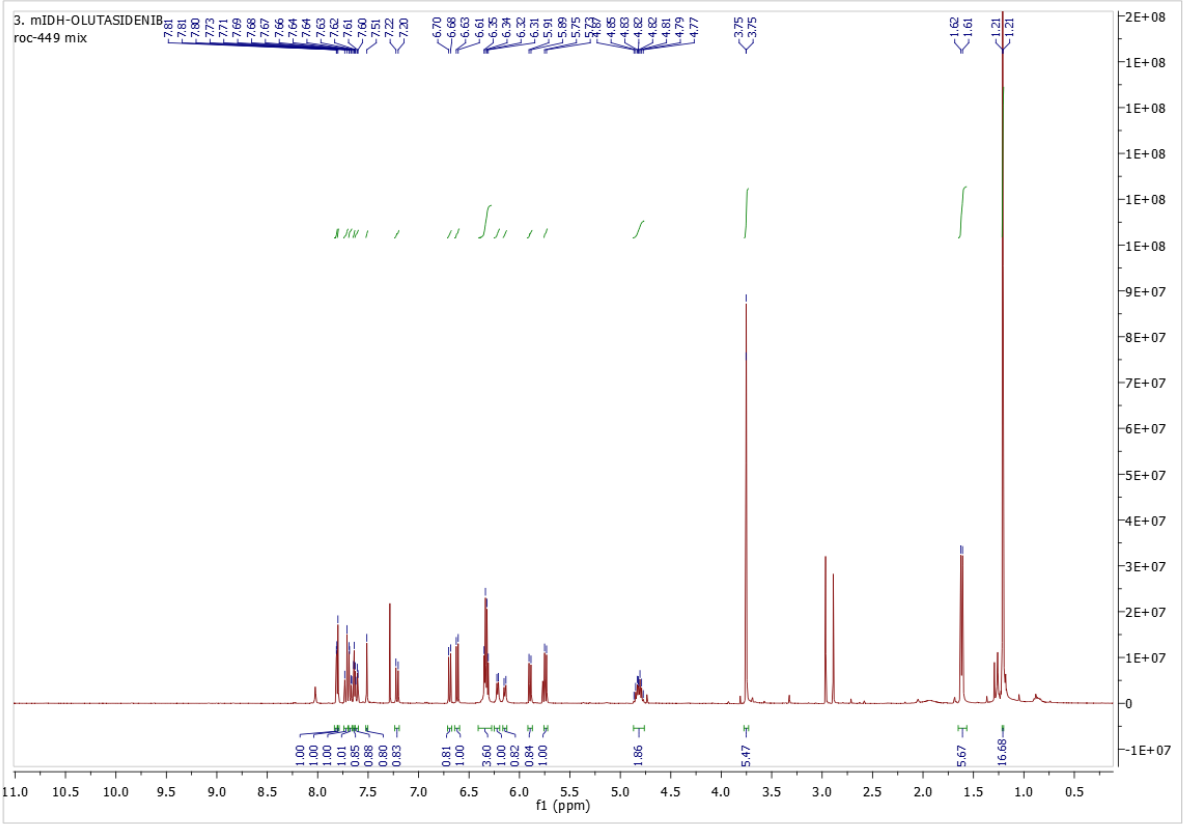

5.45. (*S*)-(3-(1-((6-Cyano-1-methyl-2-oxo-1,2-dihydropyridin-3-yl)amino)ethyl)-2-oxo-6-(4,4,5,5-tetramethyl-1,3,2-dioxaborolan-2-yl)quinolin-1(2*H*)-yl)methyl pivalate [(*S*)-**29**]

^{1}H NMR spectrum of (*S*)-**29**

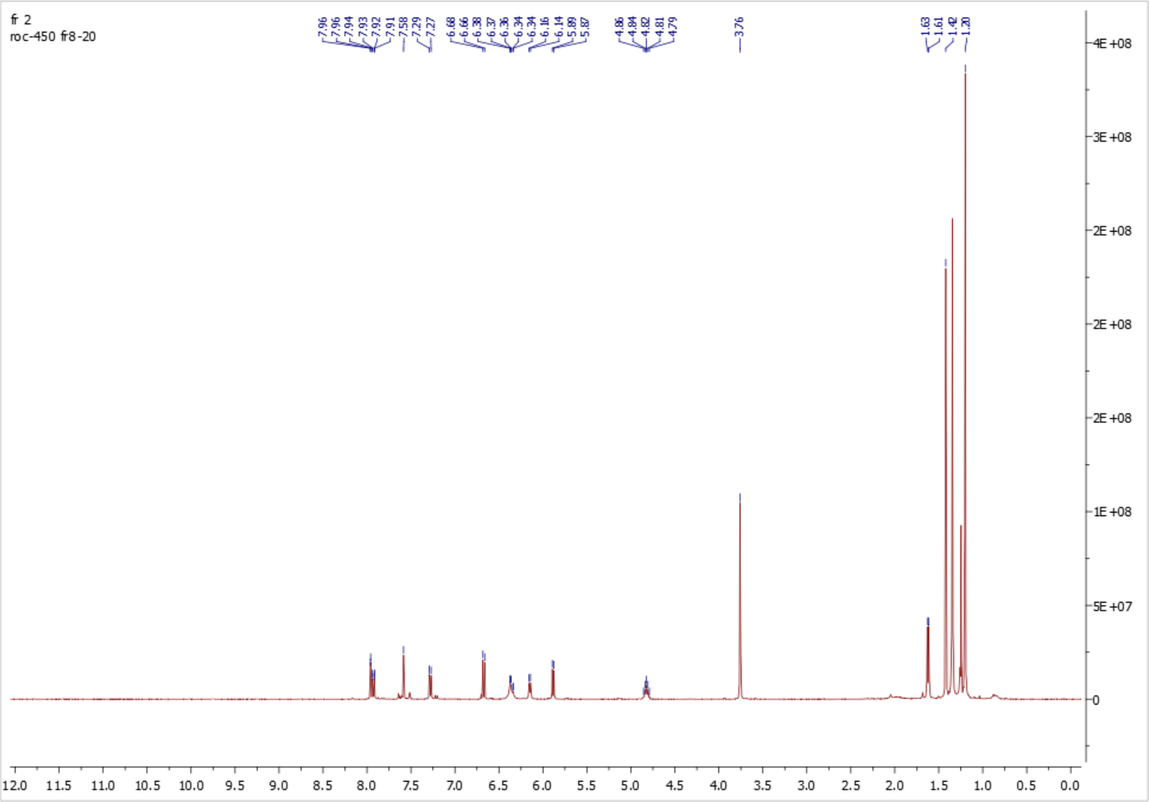

^{13}C NMR spectrum of (*S*)-**29**

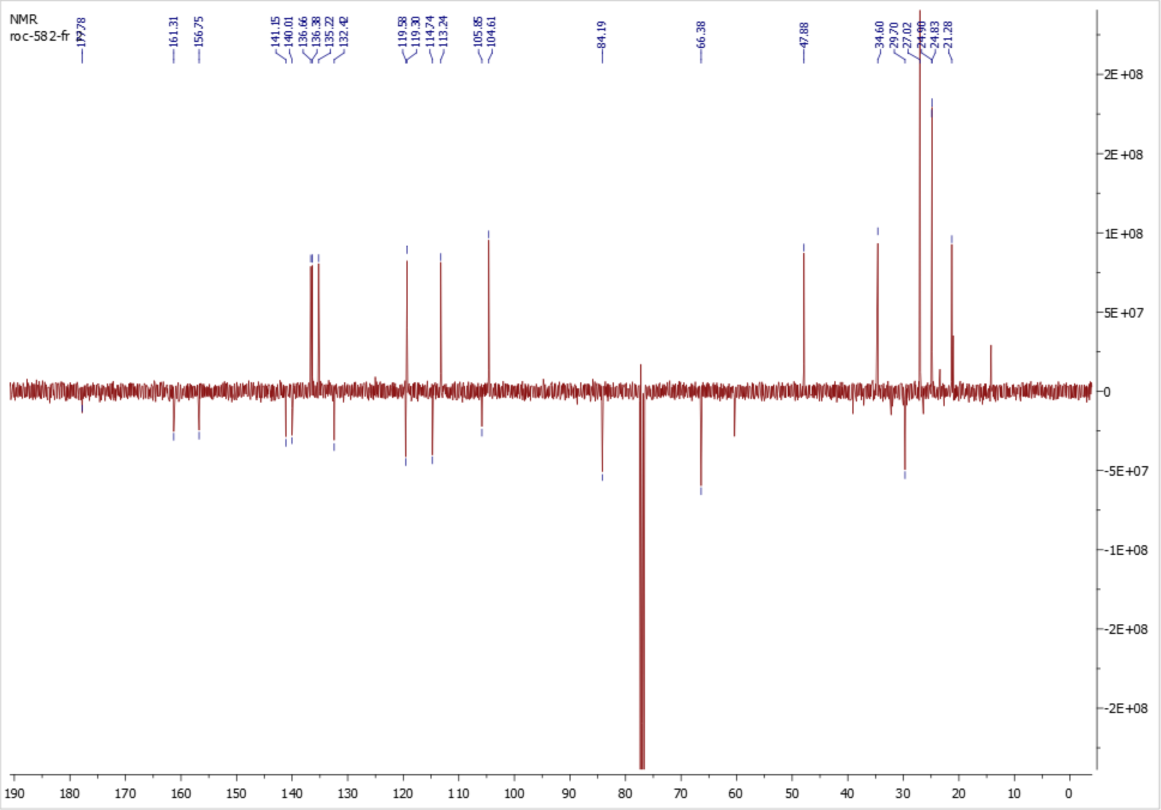

5.46. (*S*)-((3-(1-((6-Cyano-1-methyl-2-oxo-1,2-dihydropyridin-3-yl)amino)ethyl)-6-(4,4,5,5-tetramethyl-1,3,2-dioxaborolan-2-yl)quinolin-2-yl)oxy)methyl pivalate [(*S*)-**30**]

^{1}H NMR spectrum of (*S*)-**30**

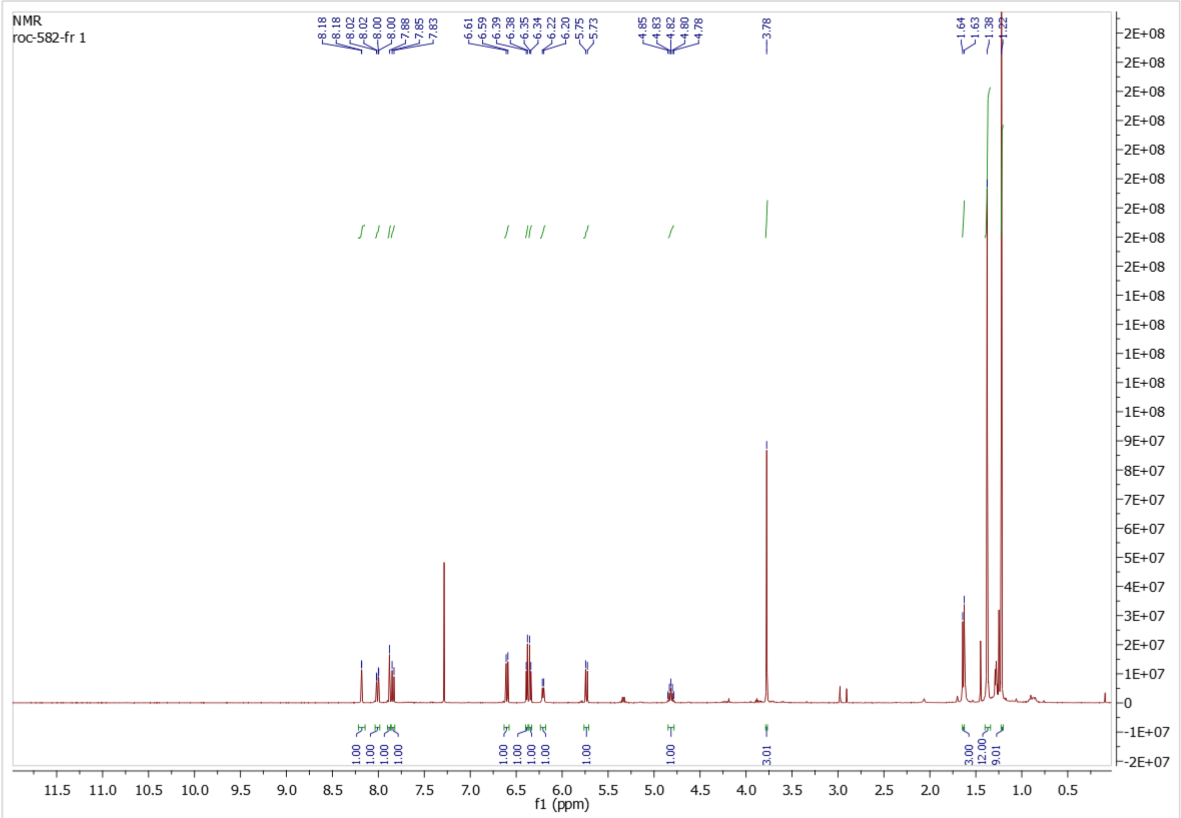

^{13}C NMR spectrum of (*S*)-**30**

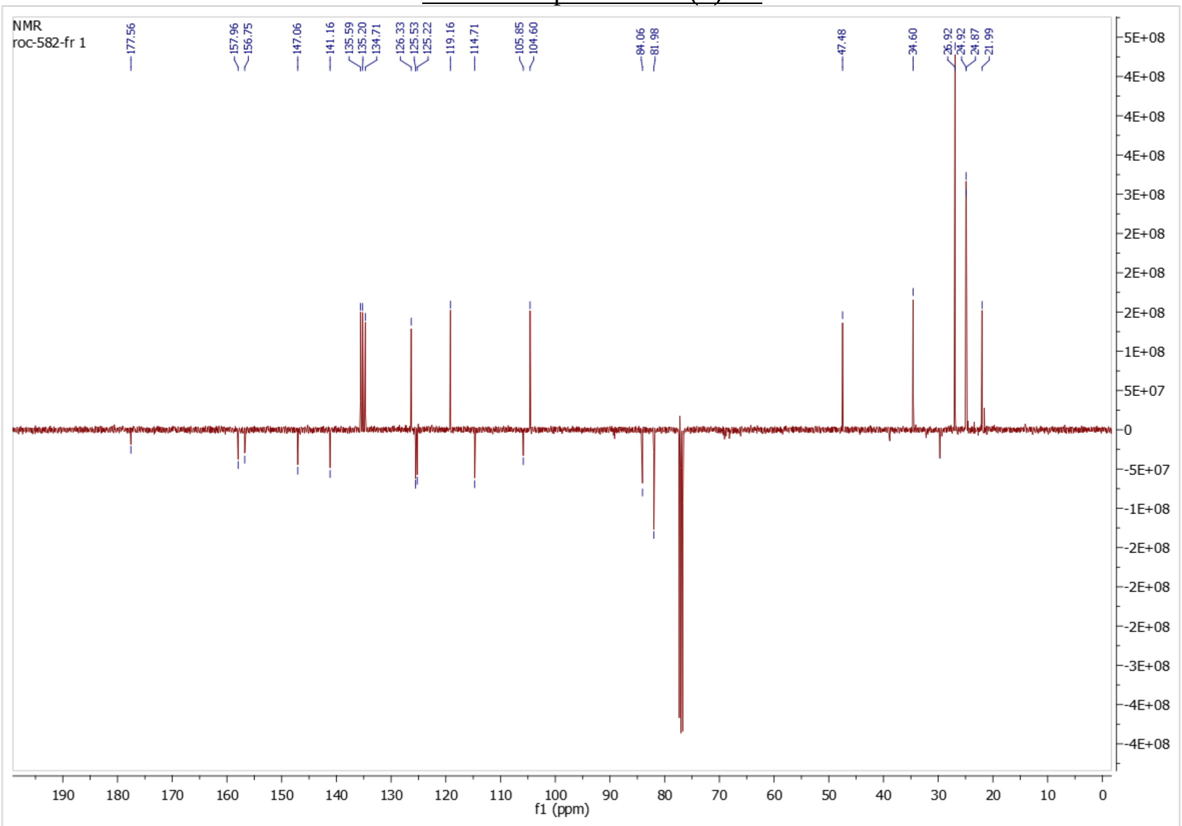

5.47. (*R*)-(3-(1-((6-Cyano-1-methyl-2-oxo-1,2-dihydropyridin-3-yl)amino)ethyl)-2-oxo-6-(4,4,5,5-tetramethyl-1,3,2-dioxaborolan-2-yl)quinolin-1(2*H*)-yl)methyl pivalate [(*R*)-**29**]

^{1}H NMR spectrum of (*R*)-**29**

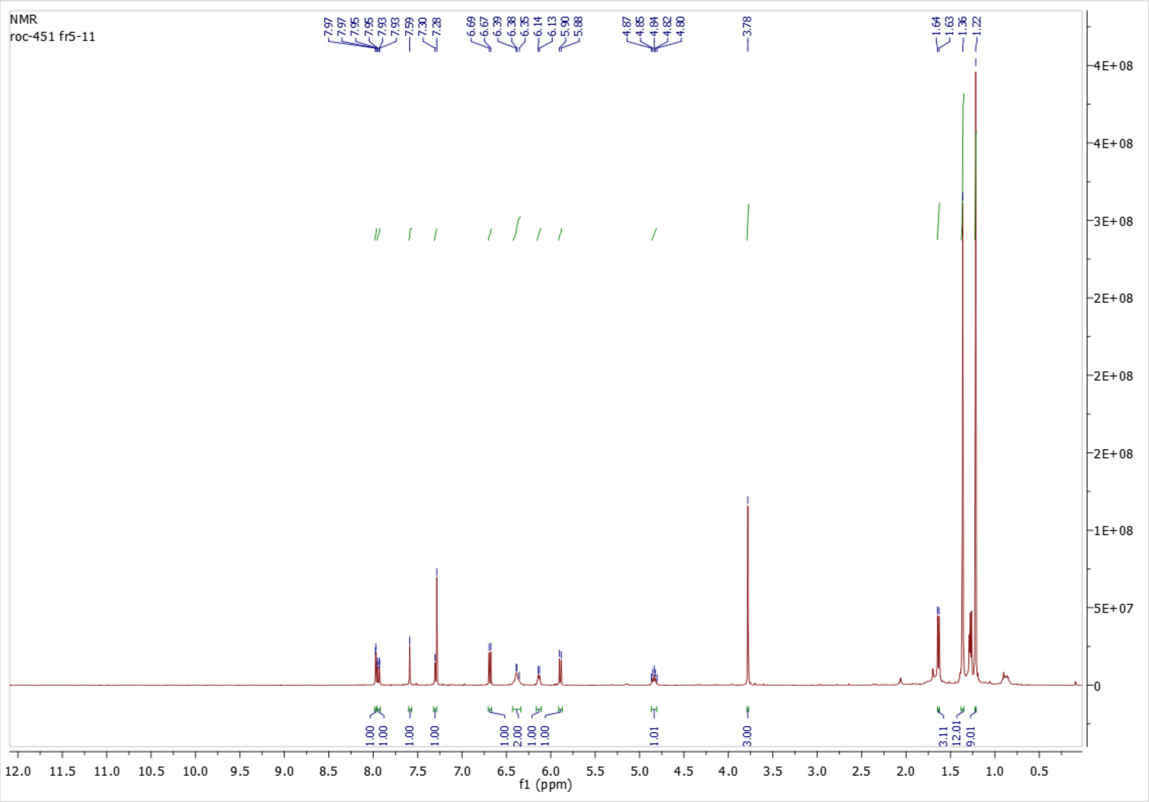

^{13}C NMR spectrum of (*R*)-**29**

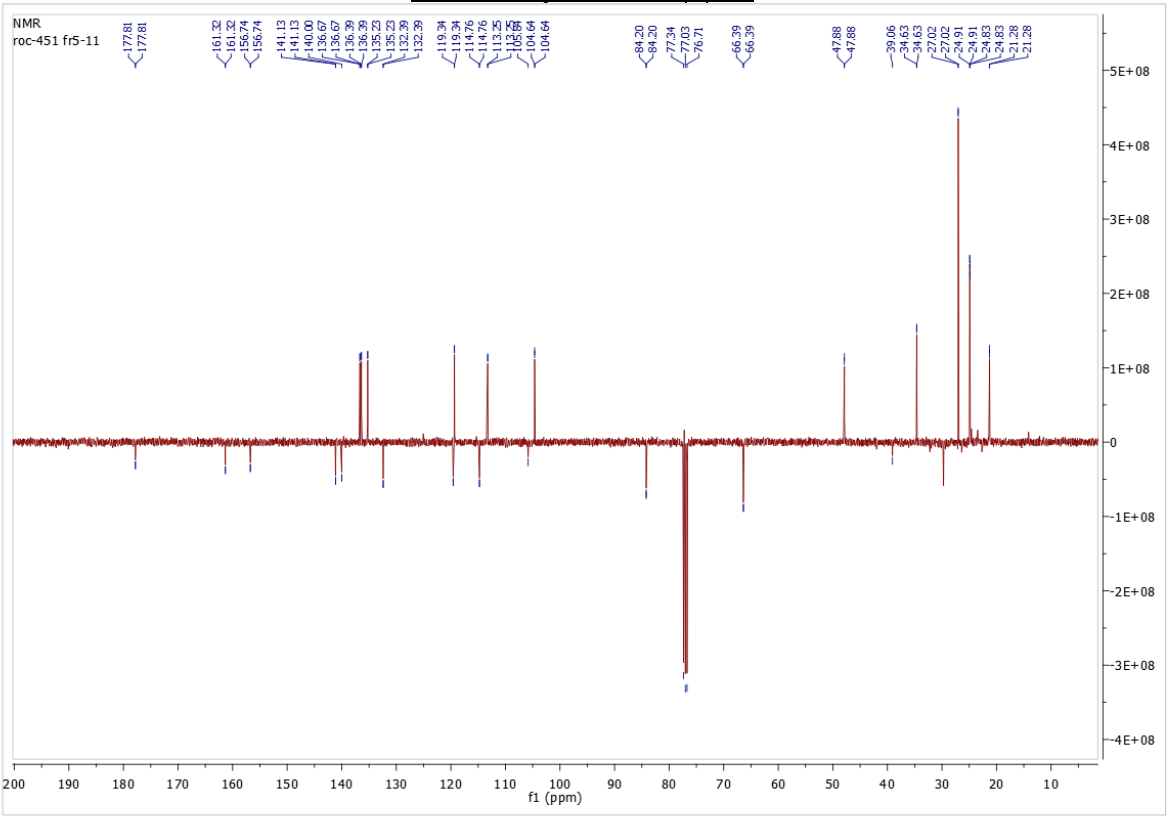

5.48. (*R*)-((3-(1-((6-Cyano-1-methyl-2-oxo-1,2-dihydropyridin-3-yl)amino)ethyl)-6-(4,4,5,5-tetramethyl-1,3,2-dioxaborolan-2-yl)quinolin-2-yl)oxy)methyl pivalate [(*R*)-**30**]

^{1}H NMR spectrum of (*R*)-**30**

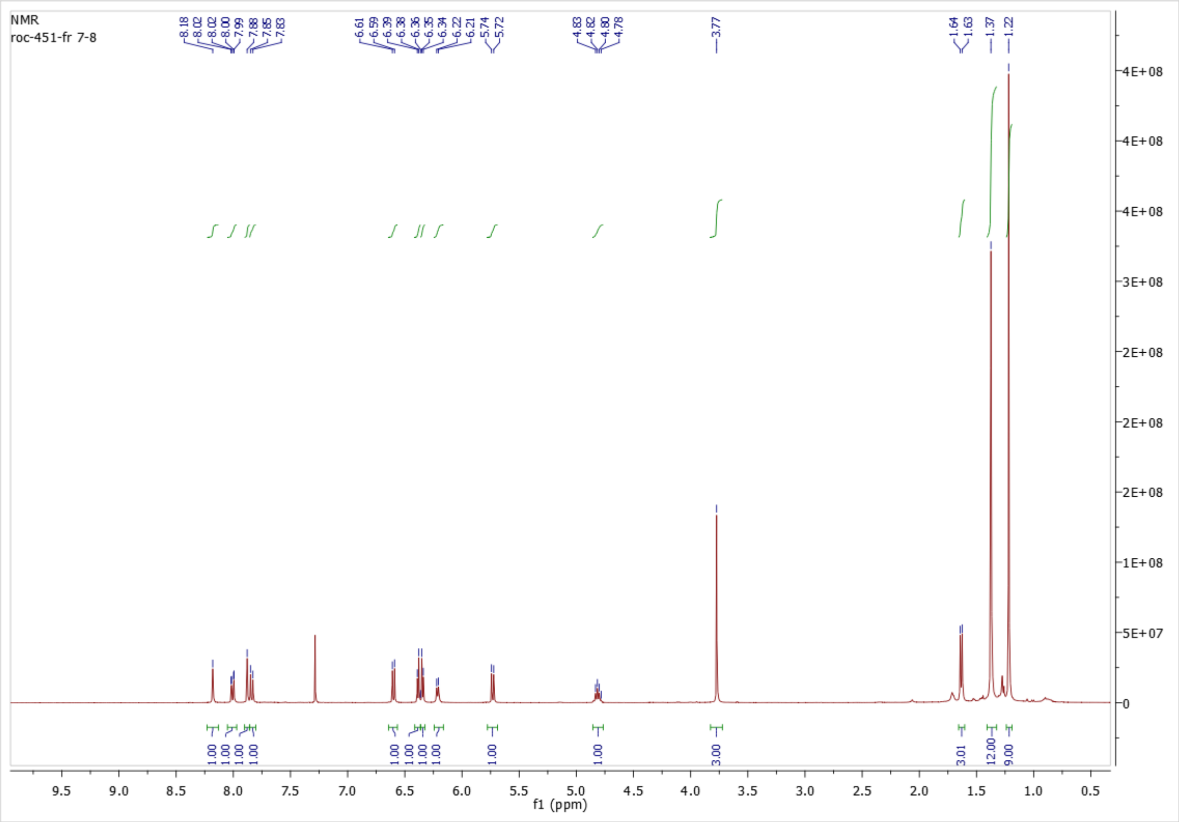

^{13}C NMR spectrum of (*R*)-**30**

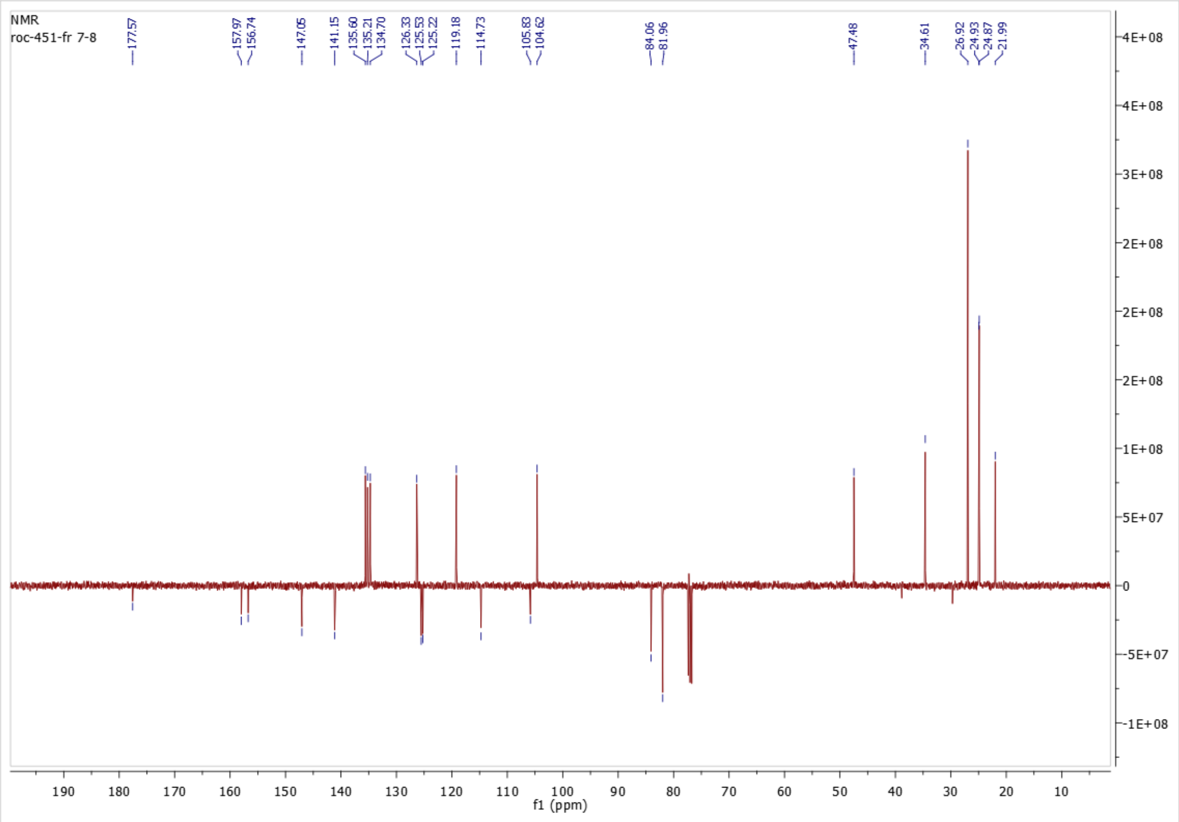

HSQC NMR spectrum of (*R*)-**30**

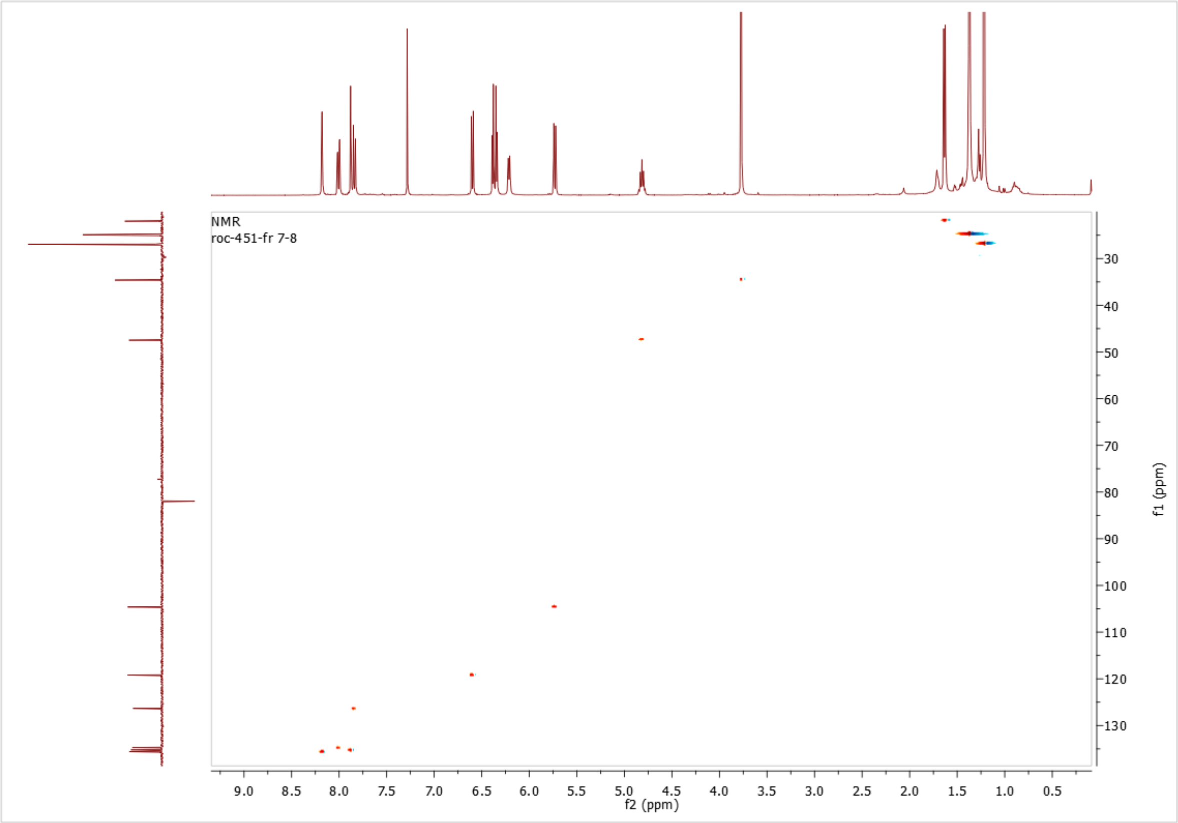

5.49. 2-Cyano-5-fluoropyridine 1-oxide (**S3**)

^{1}H NMR spectrum of **S3**

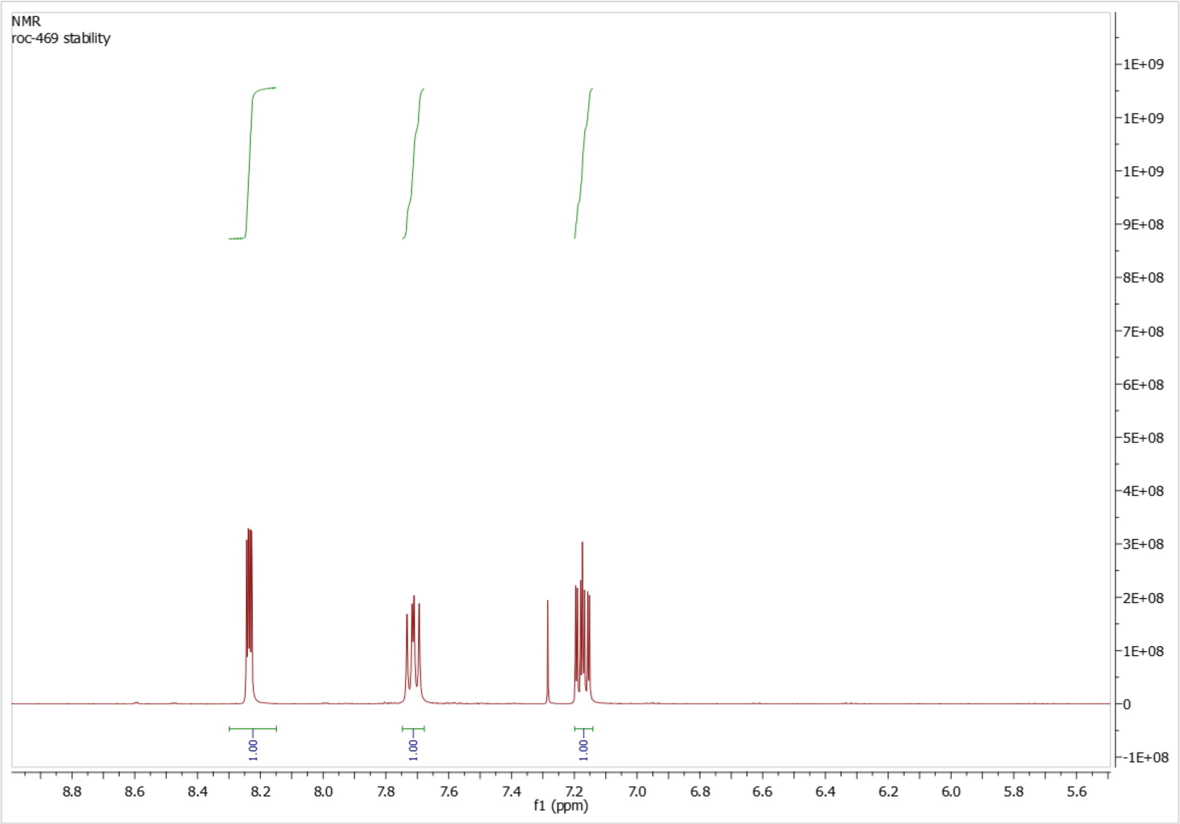

^{13}C NMR spectrum of **S3**

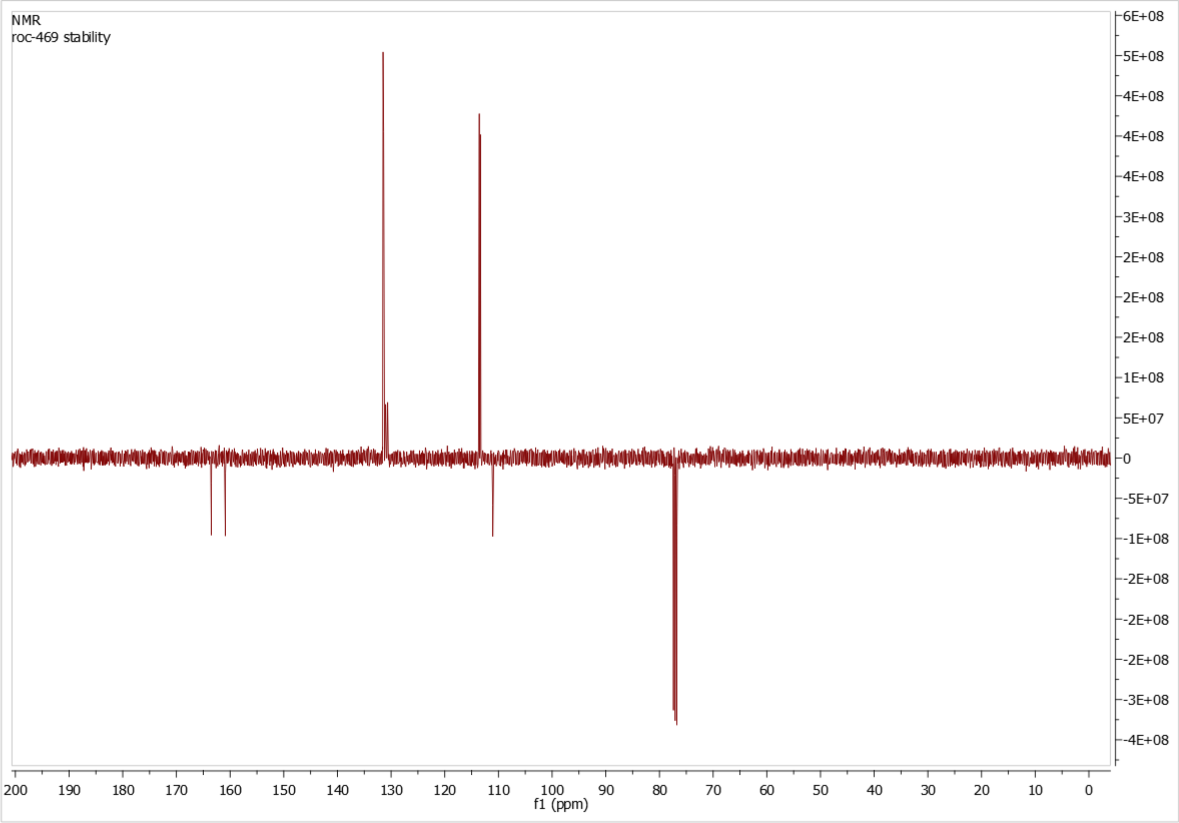

^{19}F NMR spectrum of **S3**

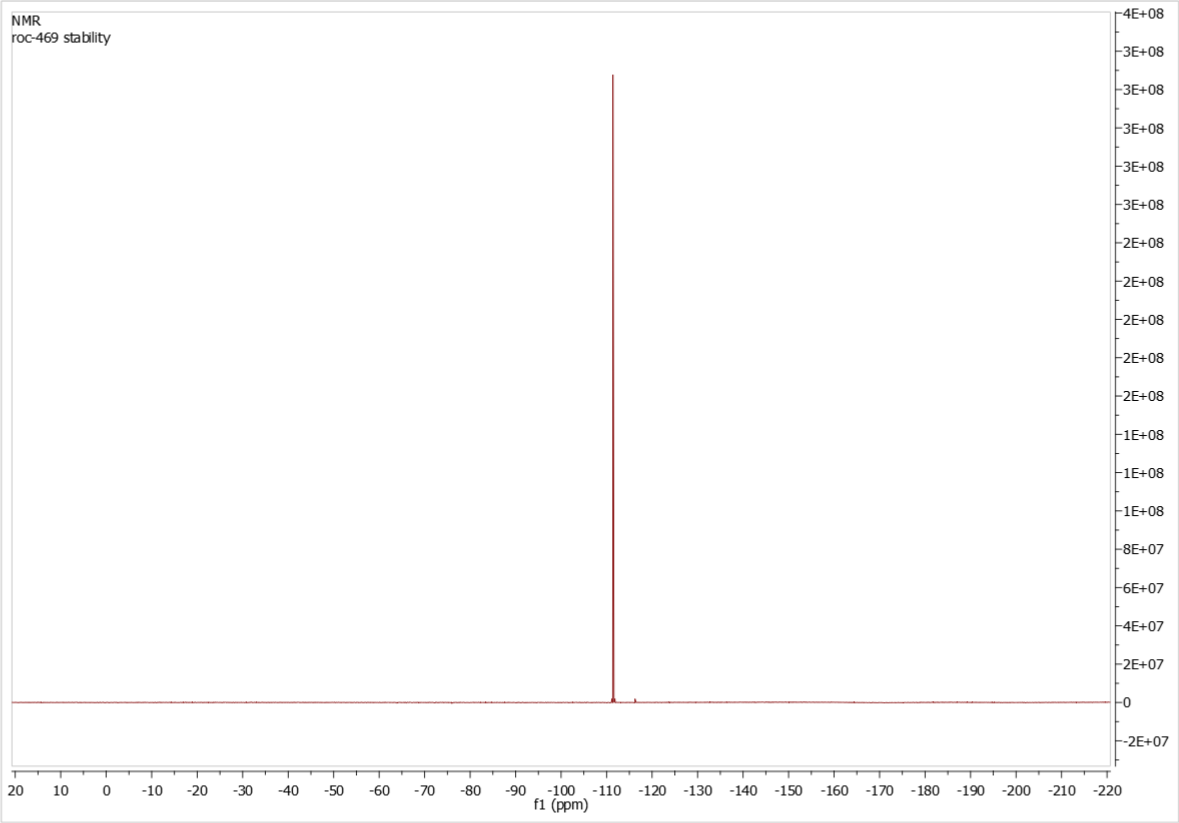

5.50. 6-Cyano-3-fluoropyridin-2-yl acetate (**S4**)

^{1}H NMR spectrum of **S4**

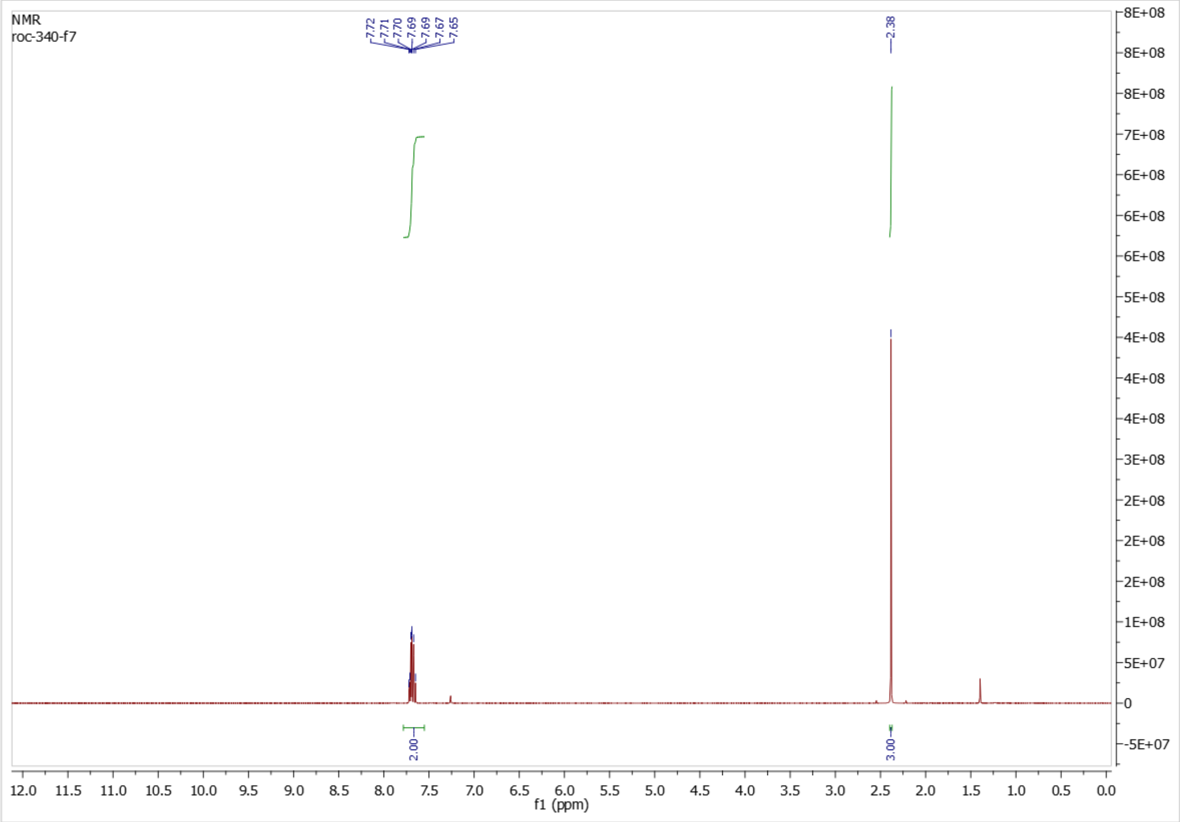

^{13}C NMR spectrum of **S4**

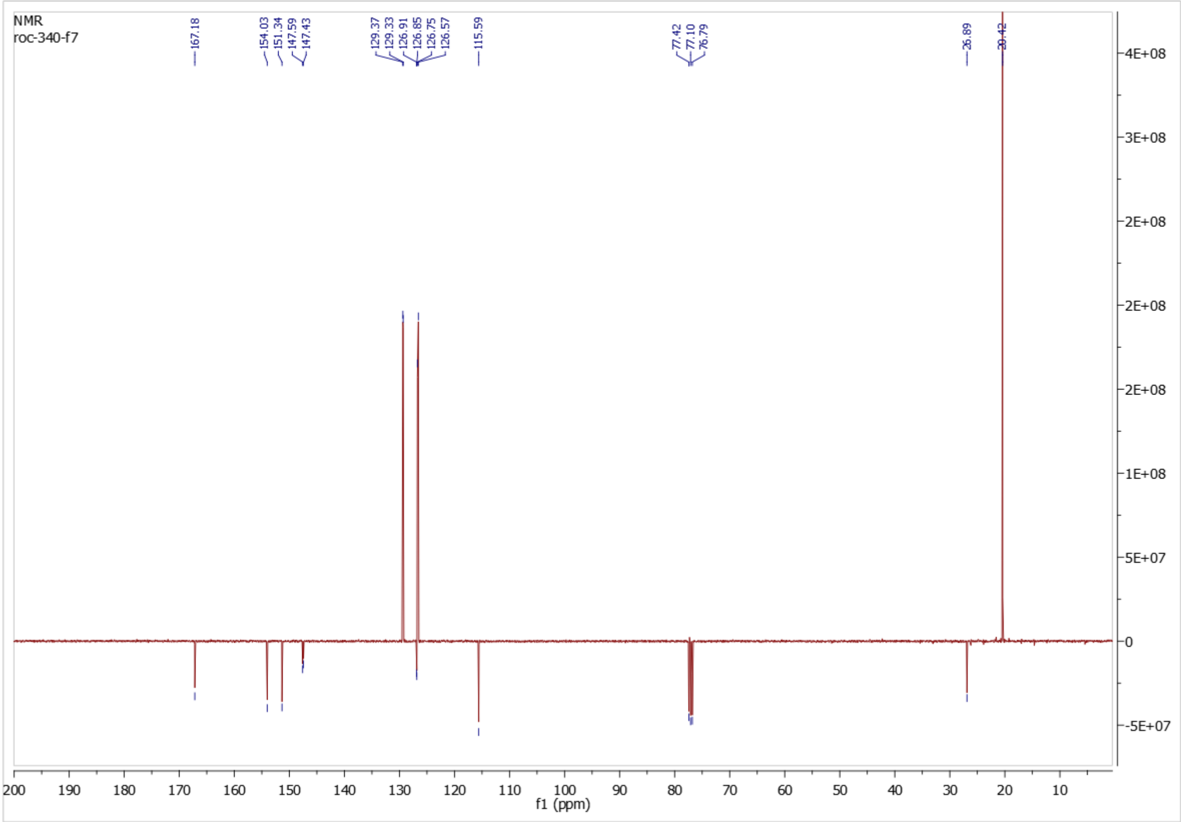

^{19}F NMR spectrum of **S4**

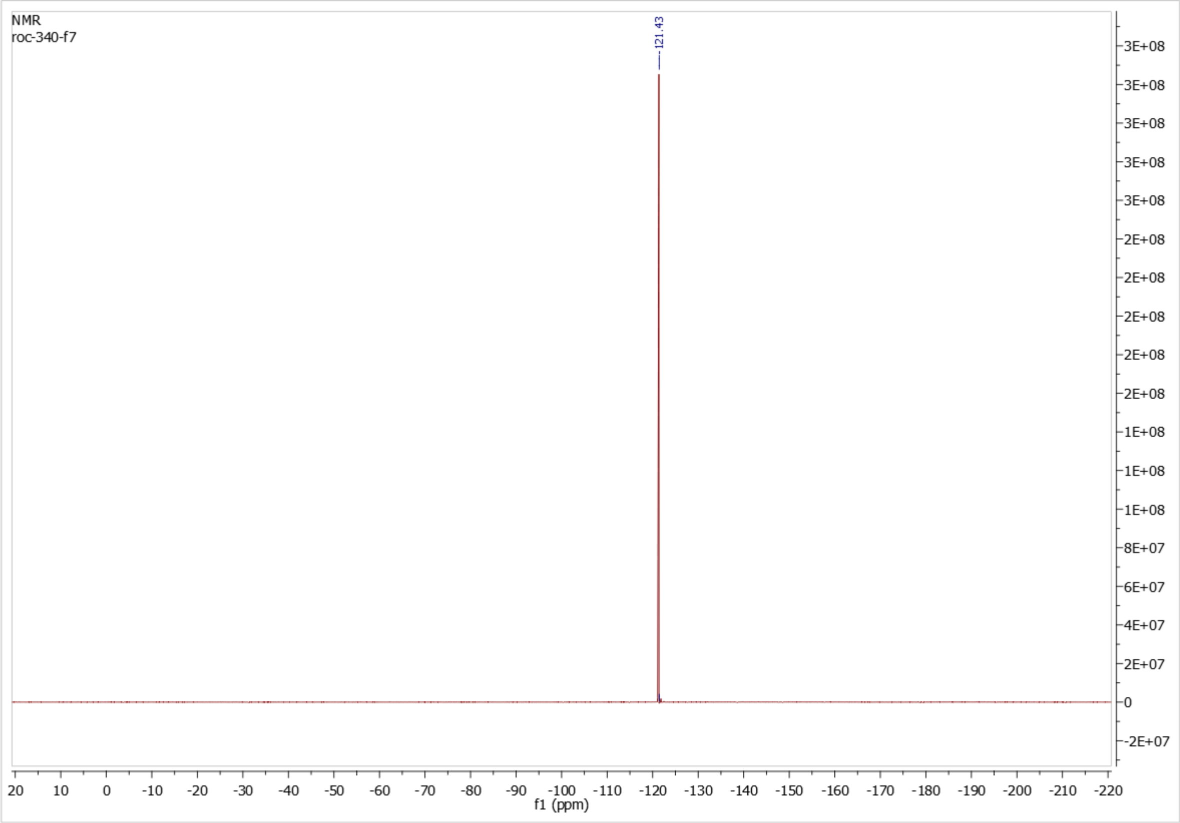

5.51. 6-Cyanopyridine-2,3-diyl diacetate (**S4b**)

^{1}H NMR spectrum of **S4b**

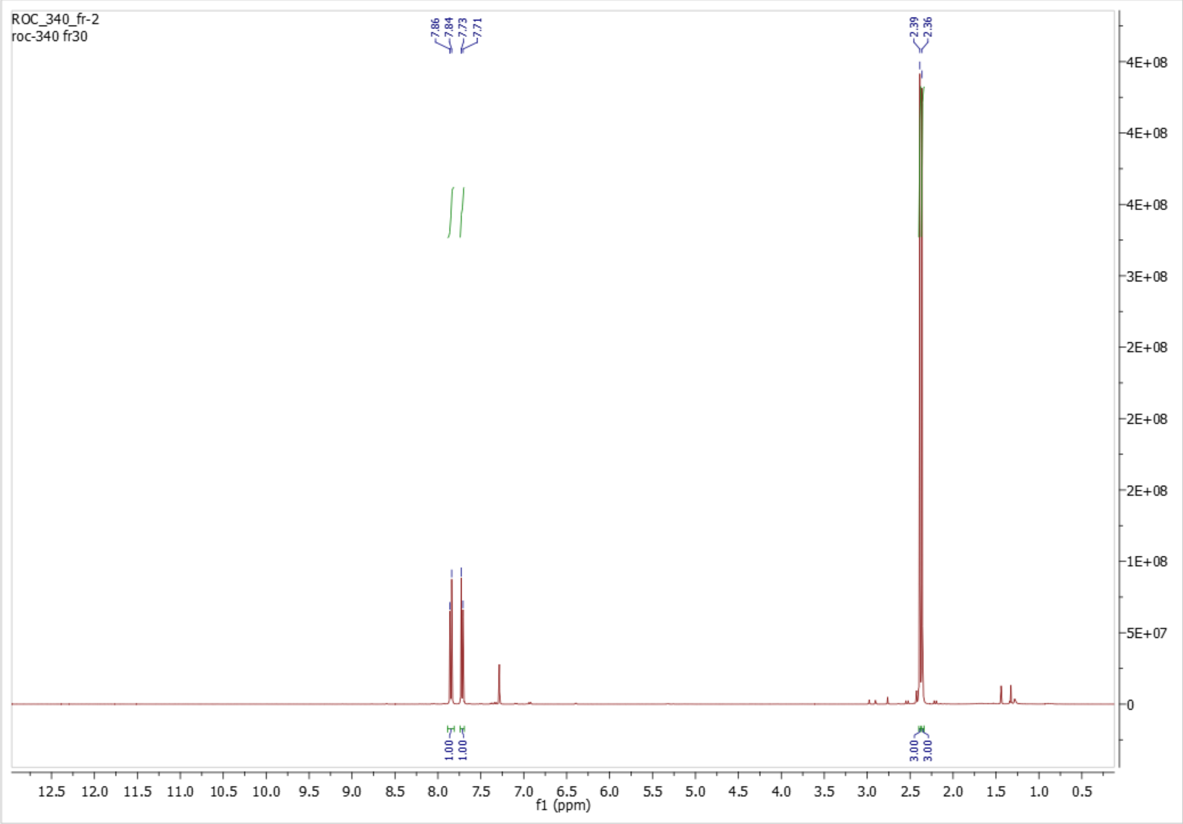

5.52. 5-Fluoro-6-oxo-1,6-dihydropyridine-2-carbonitrile (**S5**)

^{1}H NMR spectrum of **S5**

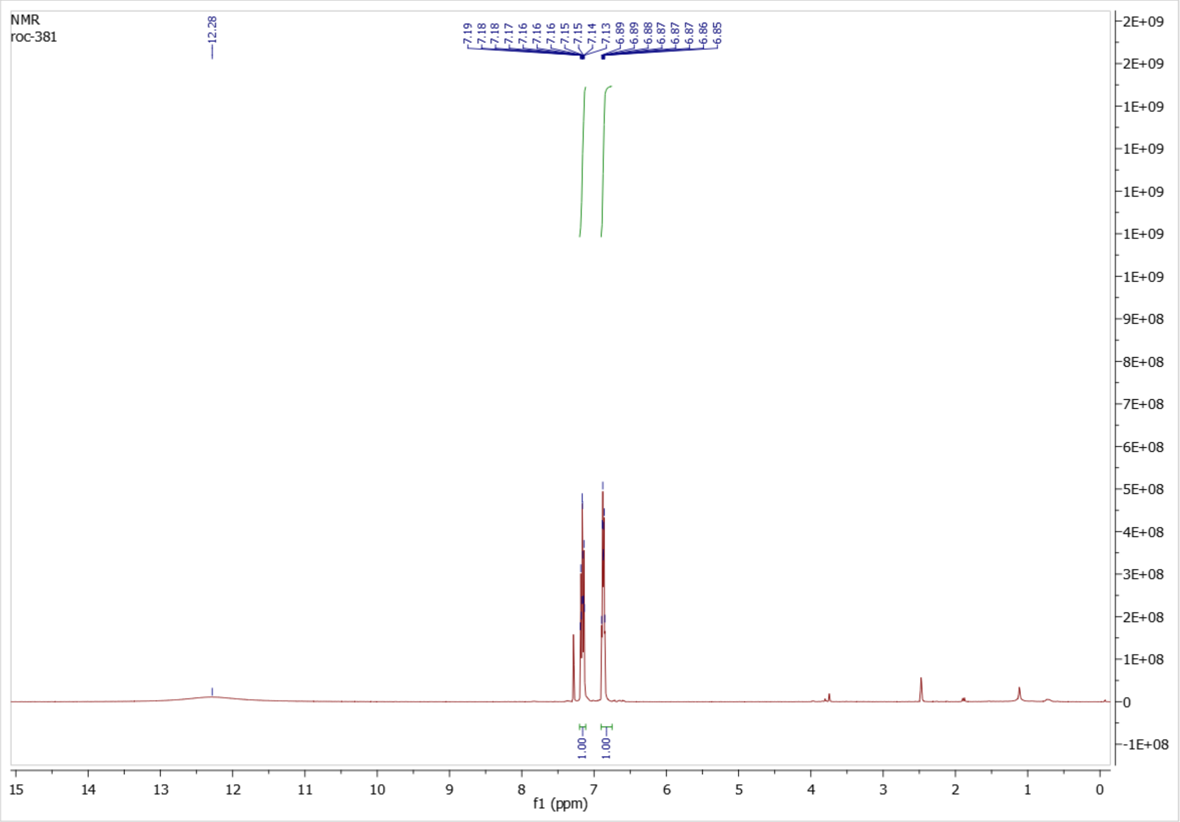

^{13}C NMR spectrum of **S5**

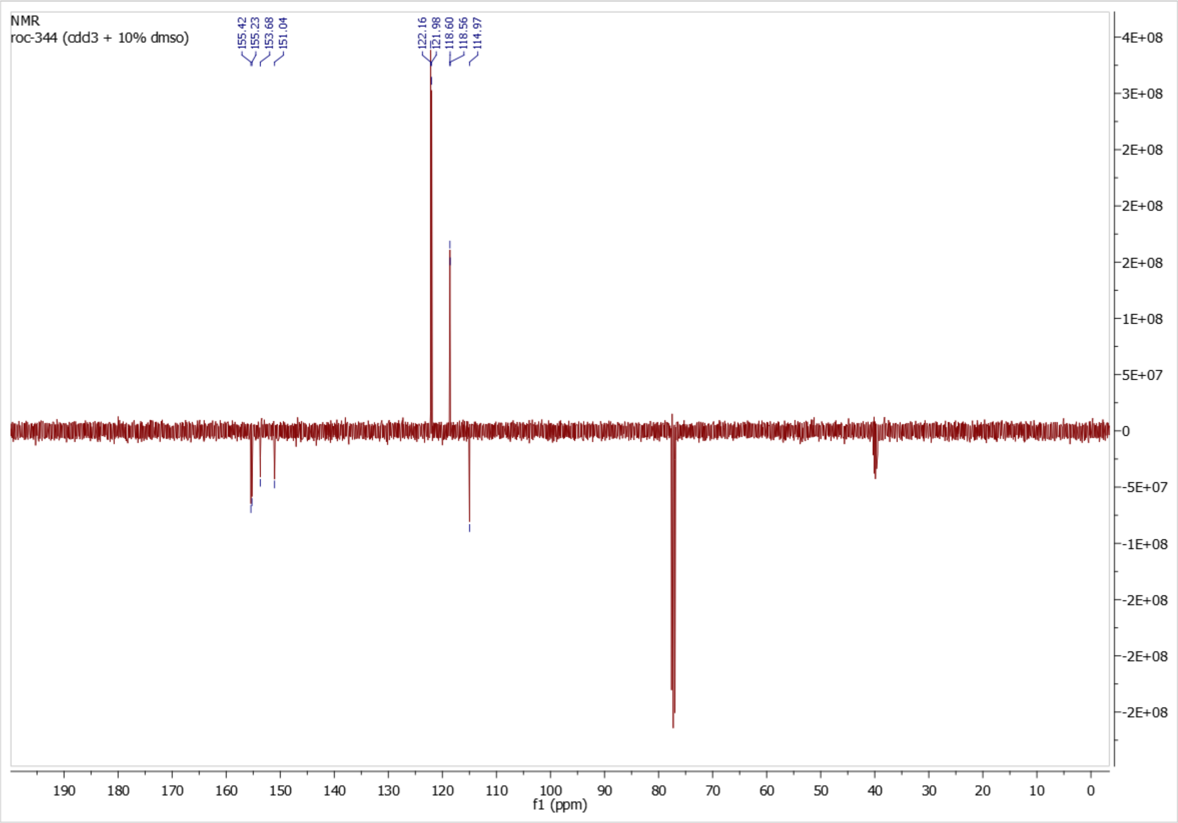

^{19}F NMR spectrum of **S5**

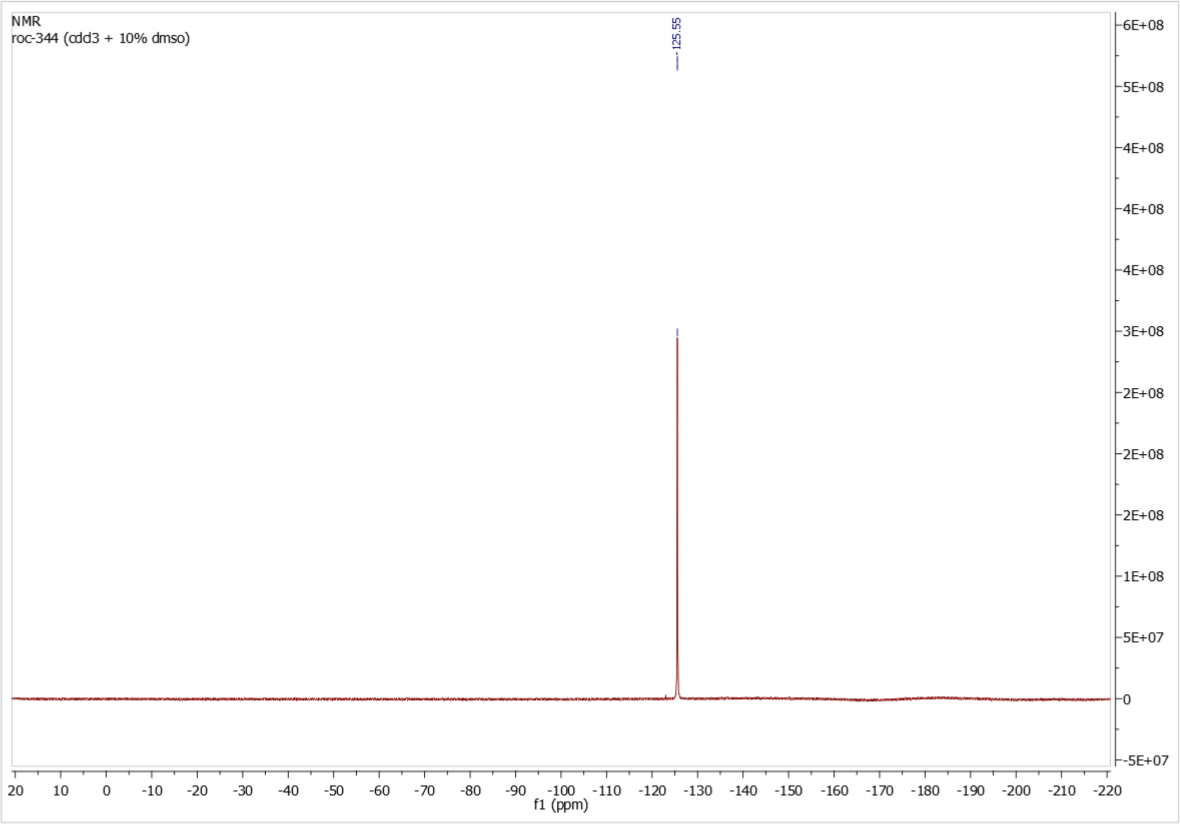

5.53. 5-Fluoro-1-methyl-6-oxo-1,6-dihydropyridine-2-carbonitrile (**31**)

^{1}H NMR spectrum of **31**

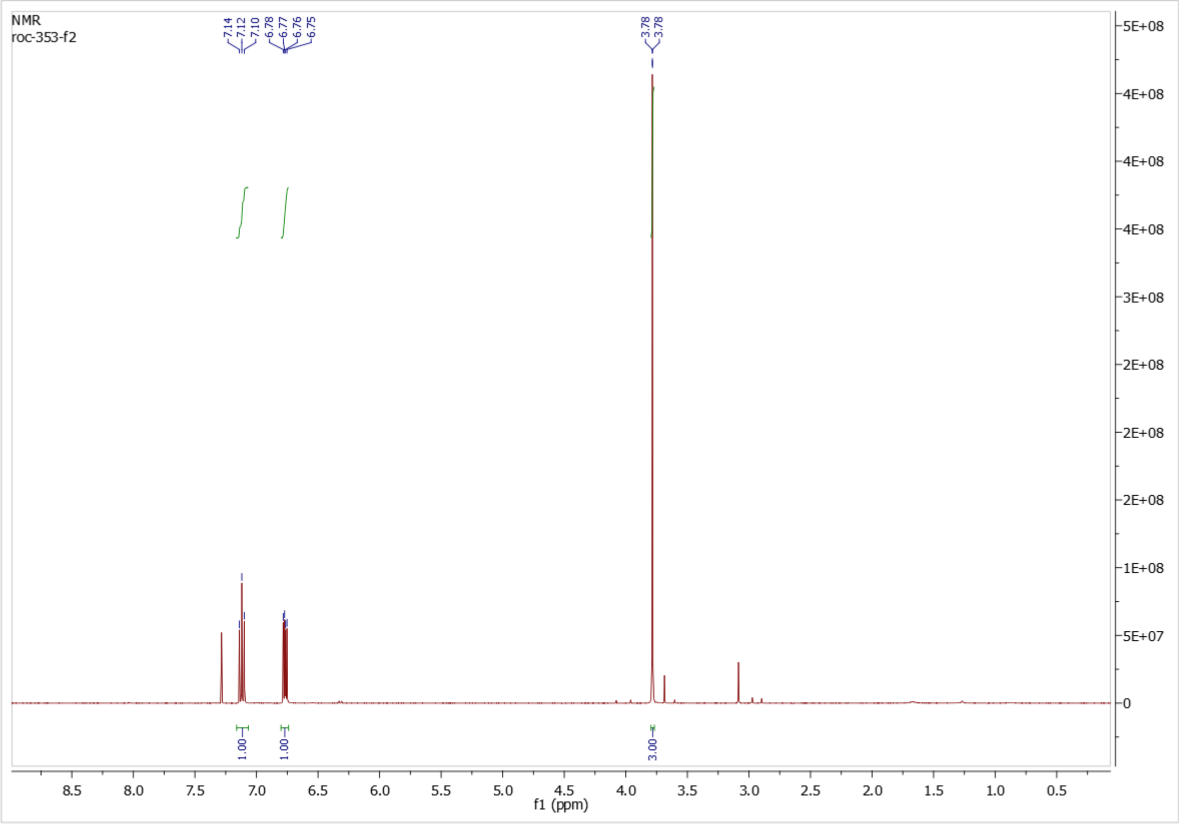

^{13}C NMR spectrum of **31**

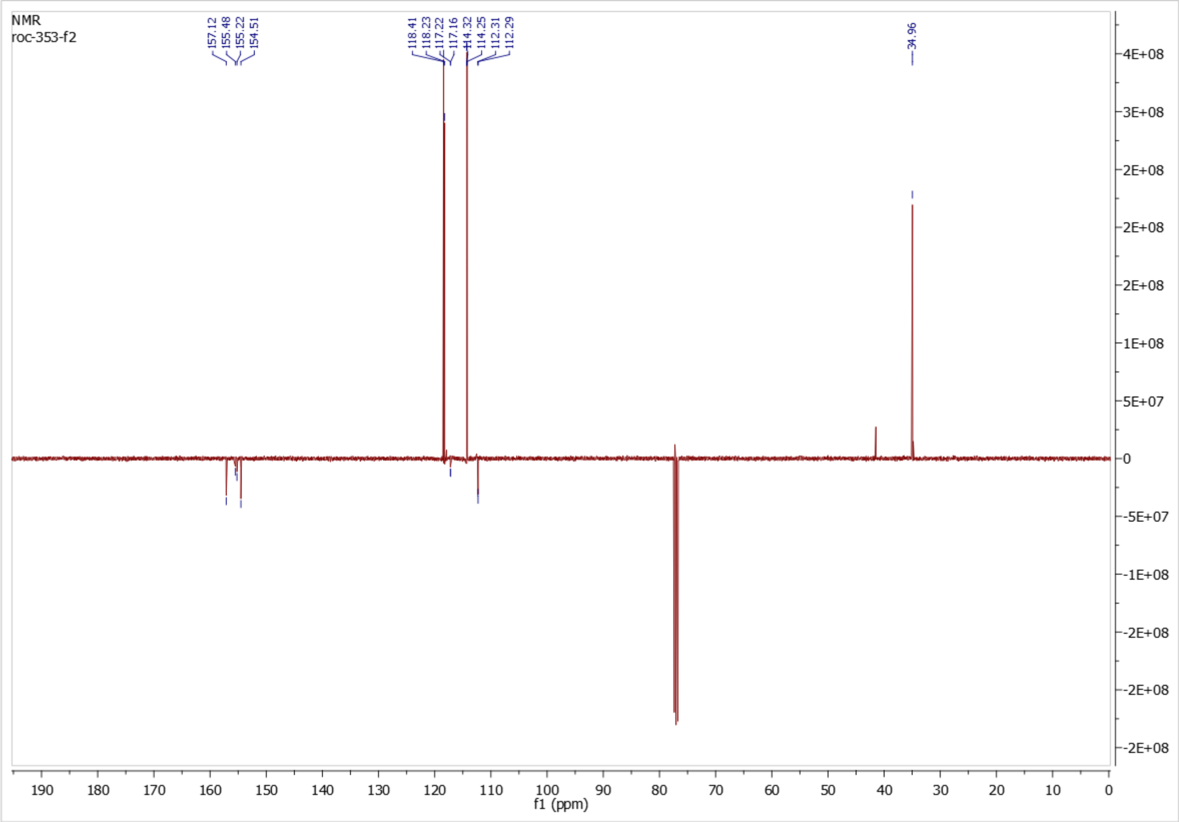

^{19}F NMR spectrum of **31**

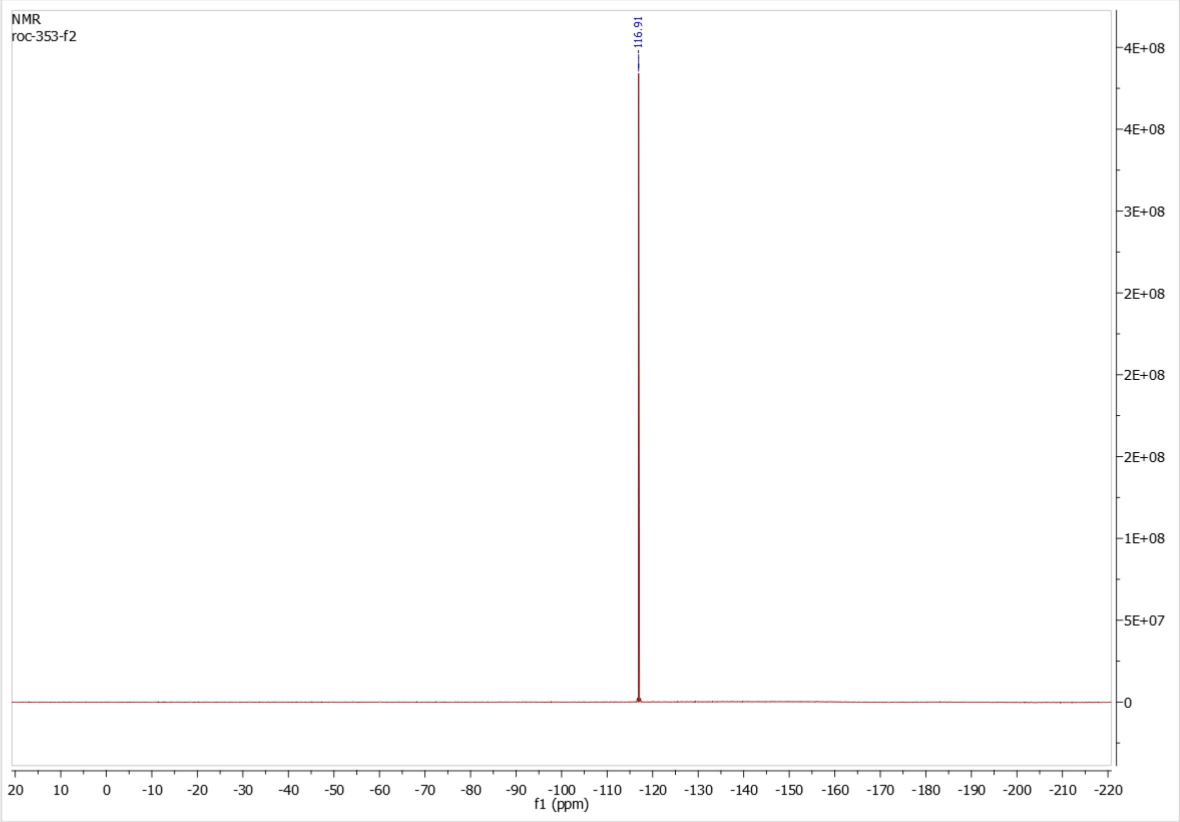

5.54. 5-Fluoro-6-methoxypicolinonitrile (**31b**)

^{1}H NMR spectrum of **31b**

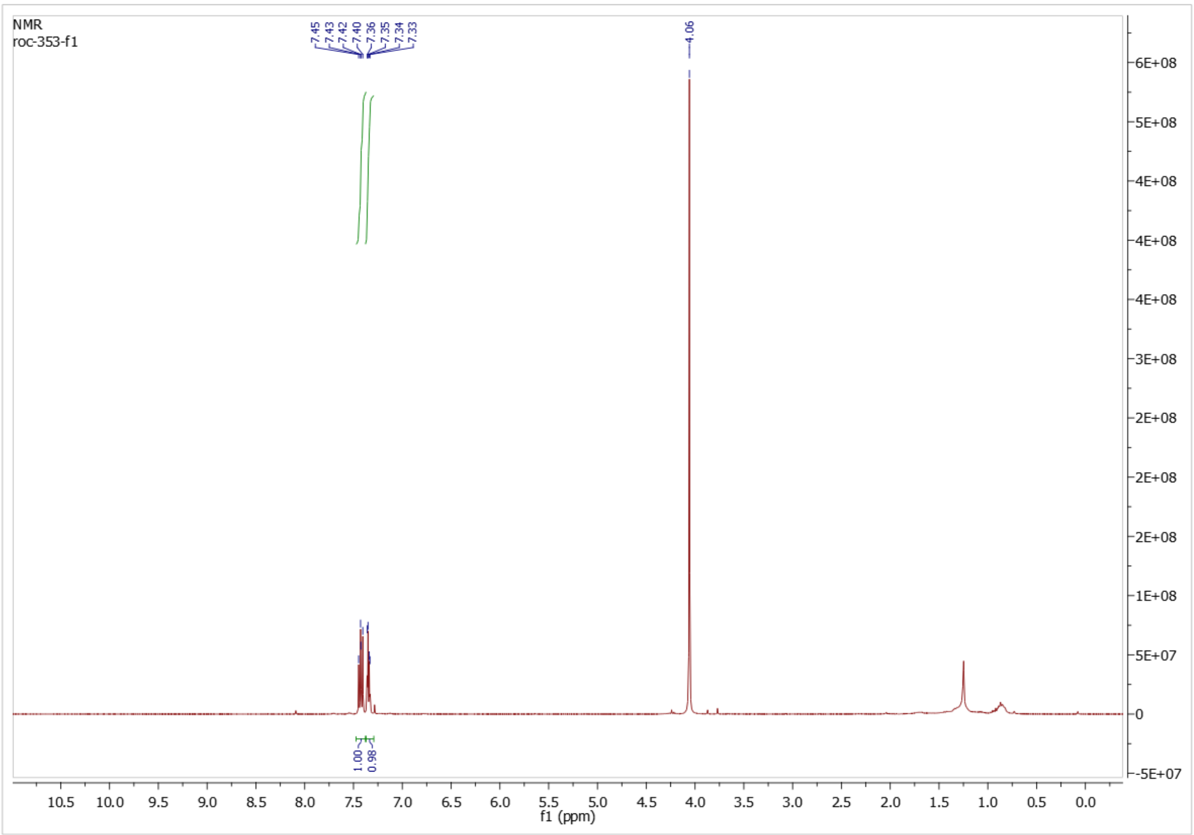

^{13}C NMR spectrum of **31b**

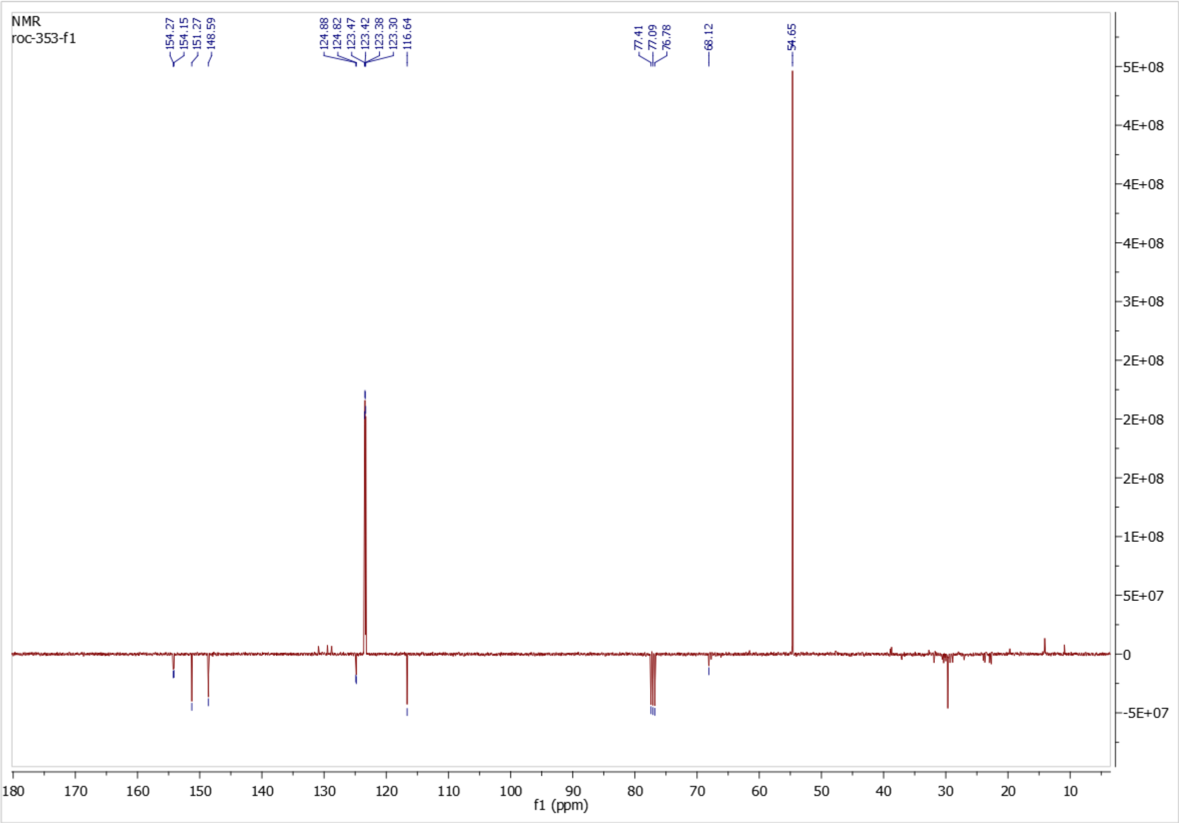

^{19}F NMR spectrum of **31b**

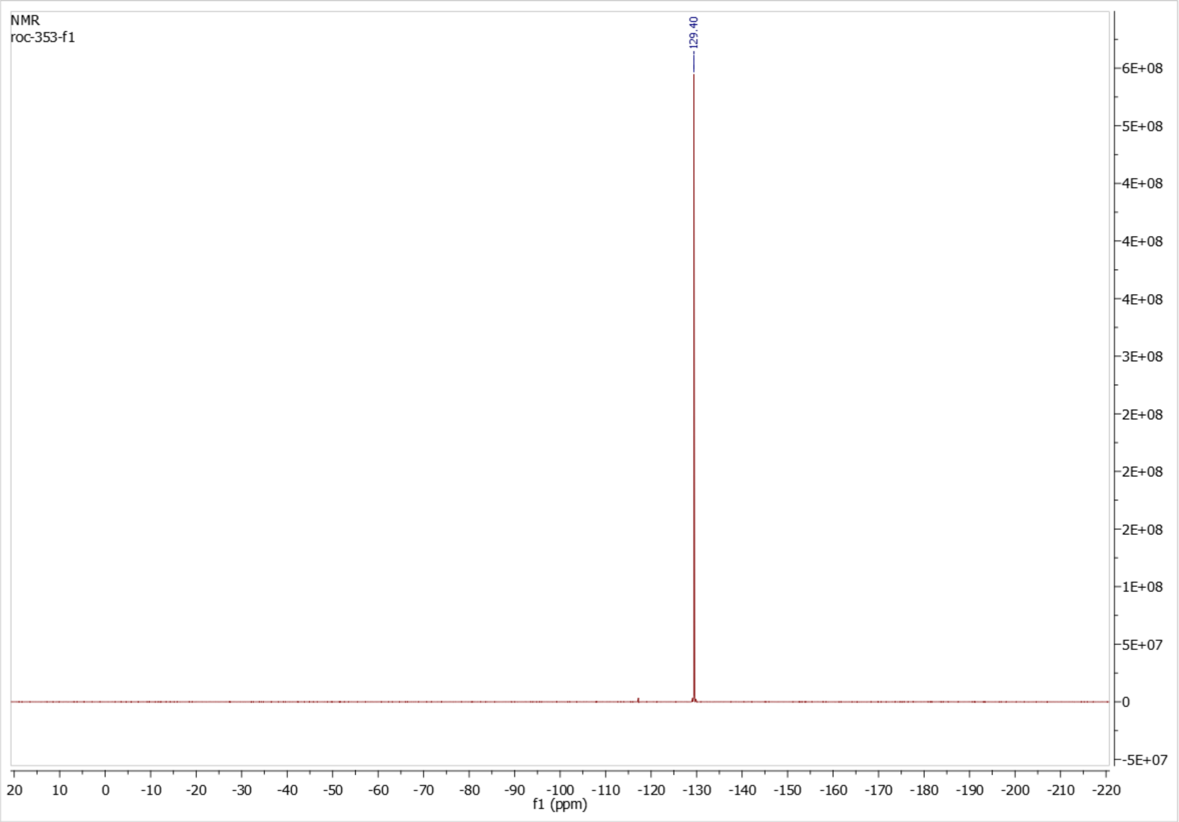

5.55. 2-Fluoroethyl methanesulfonate (**33**)

^{1}H NMR spectrum of **33**

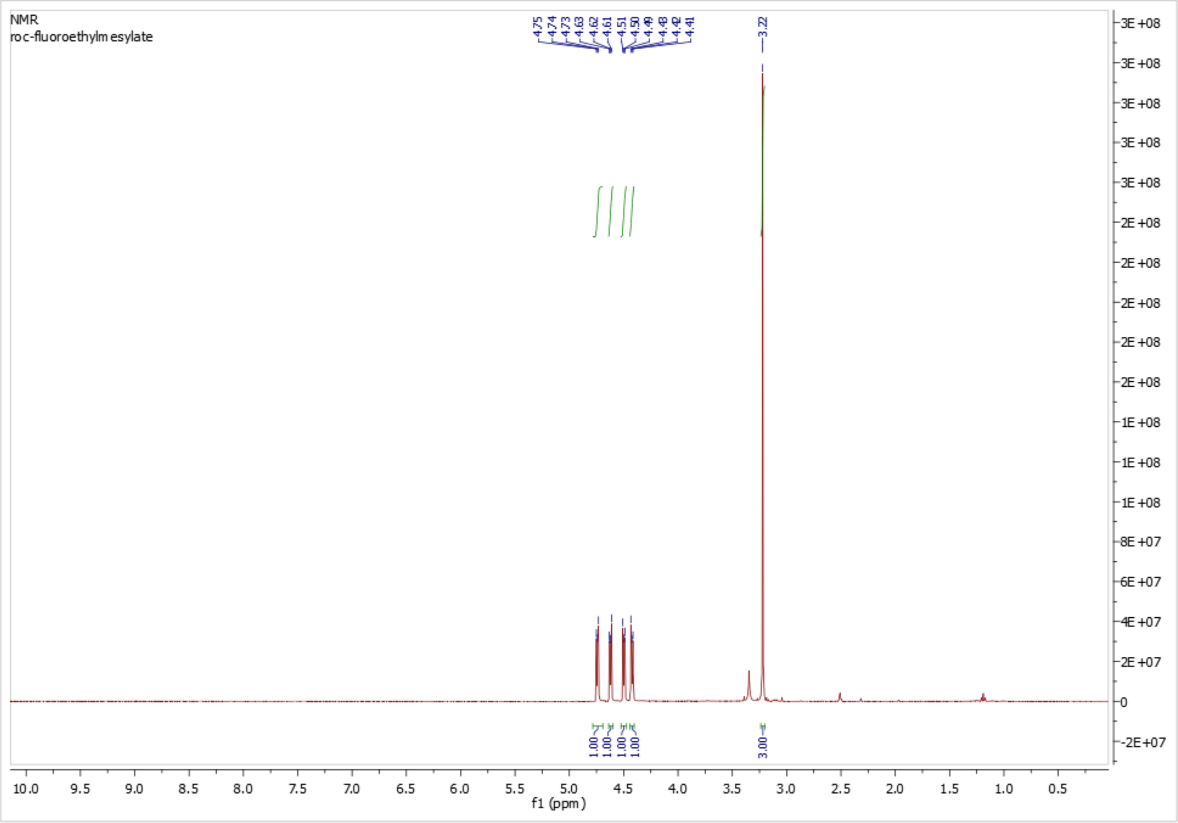

^{19}F NMR spectrum of **33**

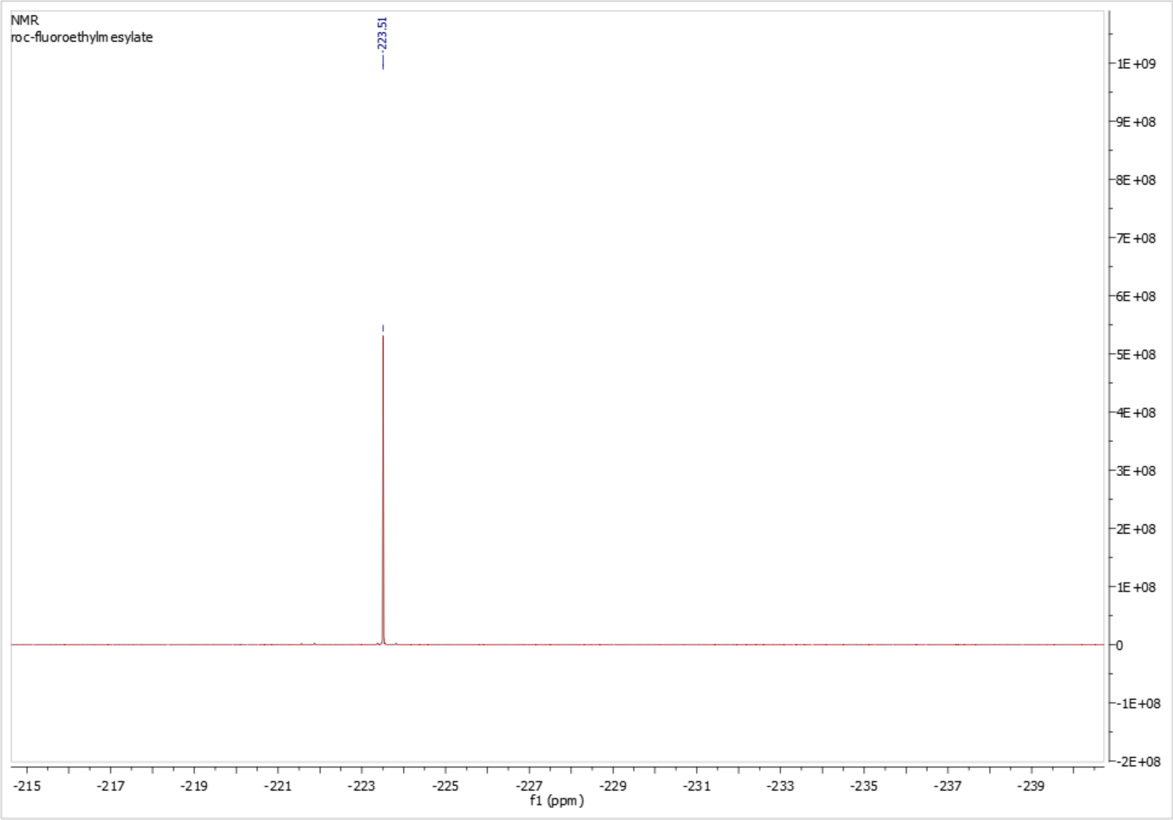

5.56. 2-(2-Fluoroethoxy)-4-nitrobenzonitrile (**35a**)

^{1}H NMR spectrum of **35a**

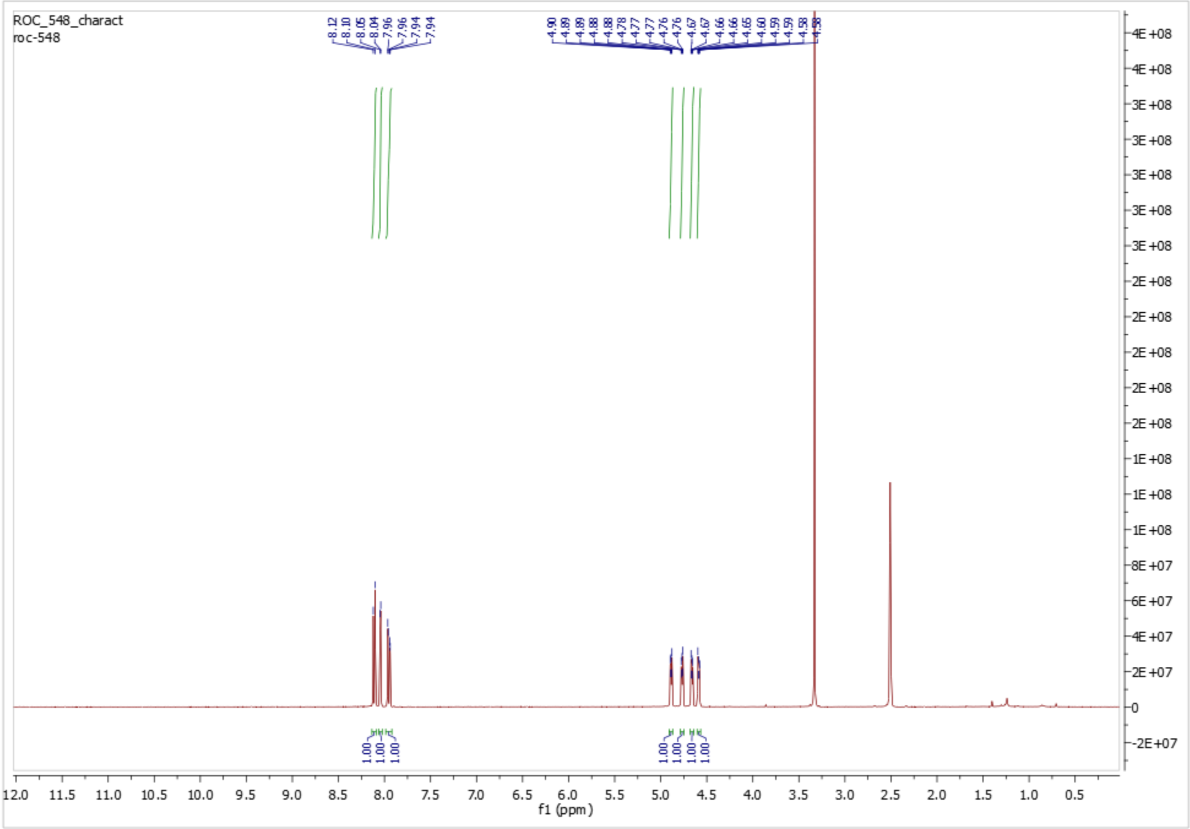

^{13}C NMR spectrum of **35a**

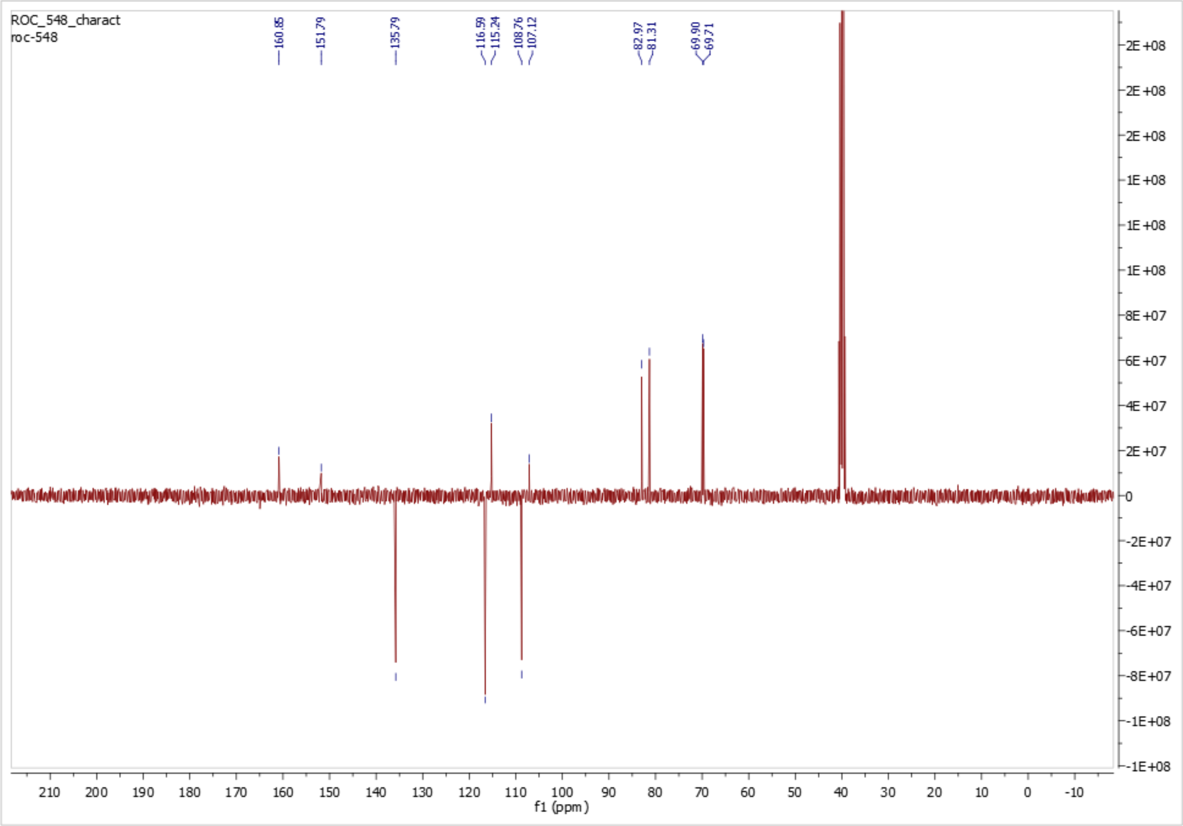

^{19}F NMR spectrum of **35a**

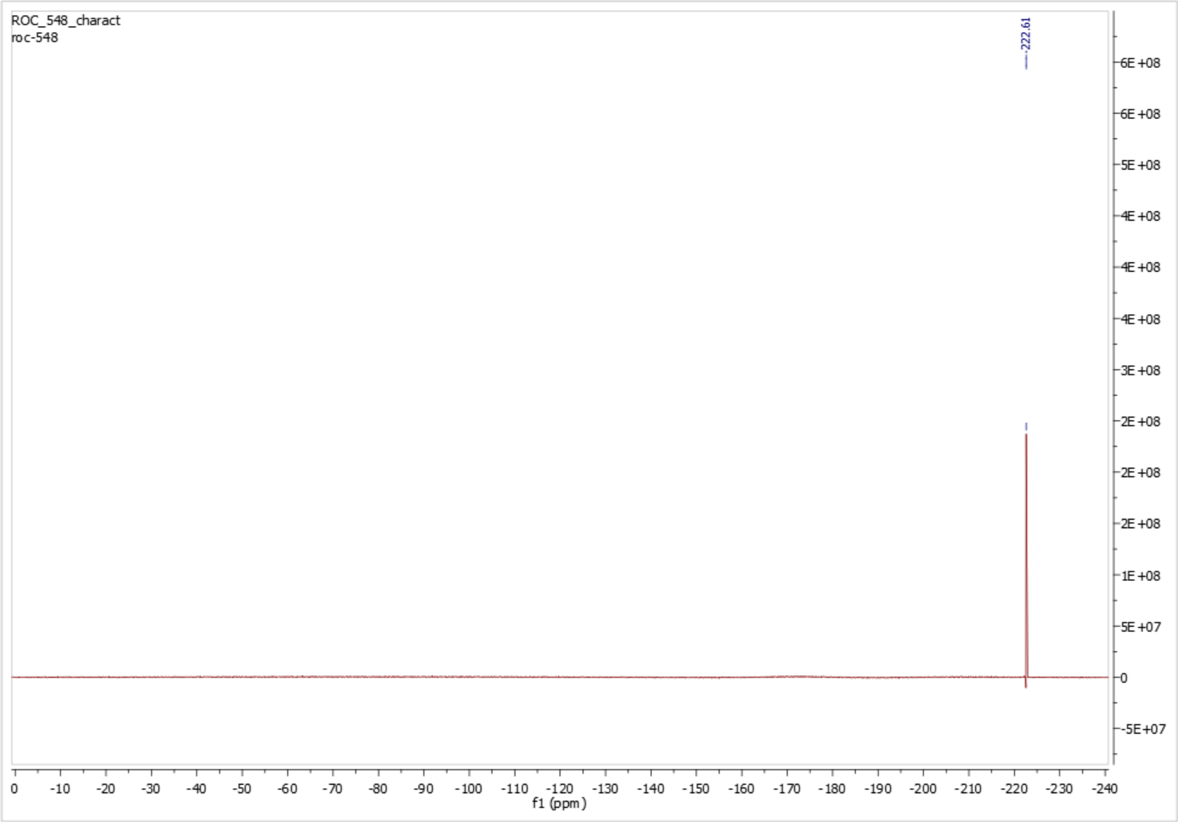

5.57. 2-(2-(Benzyloxy)ethoxy)-4-nitrobenzonitrile (**35b**)

^{1}H NMR spectrum of **35b**

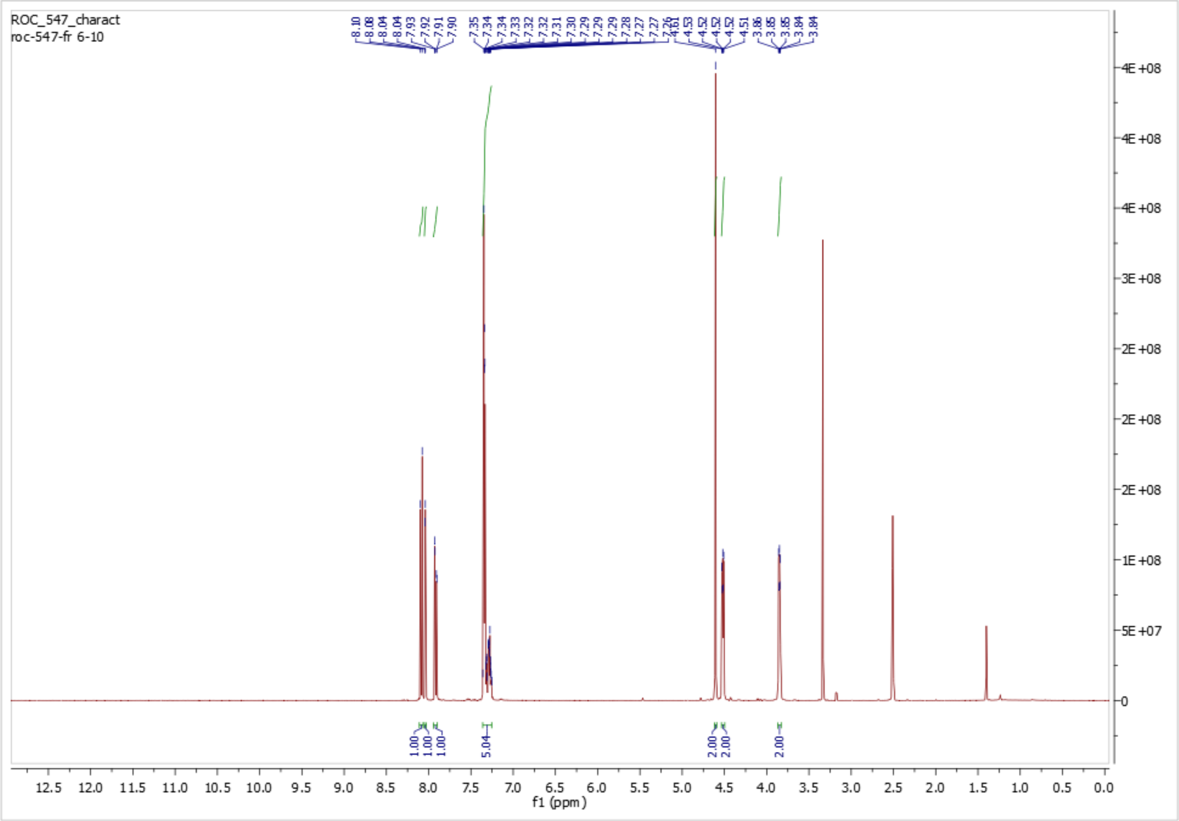

^{13}C NMR spectrum of **35b**

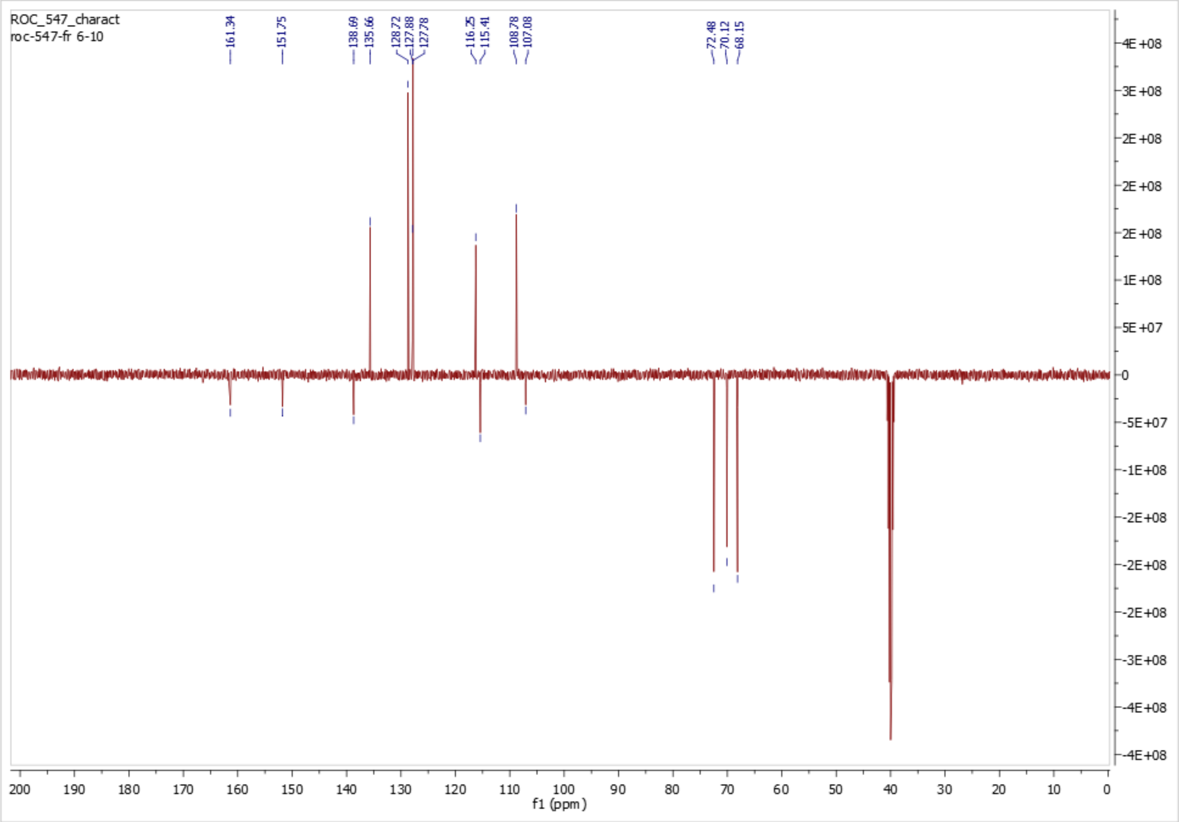

5.58. 4-Amino-2-(2-fluoroethoxy)benzonitrile (**36a**)

<u>^{1}H NMR spectrum of **36a**</u>

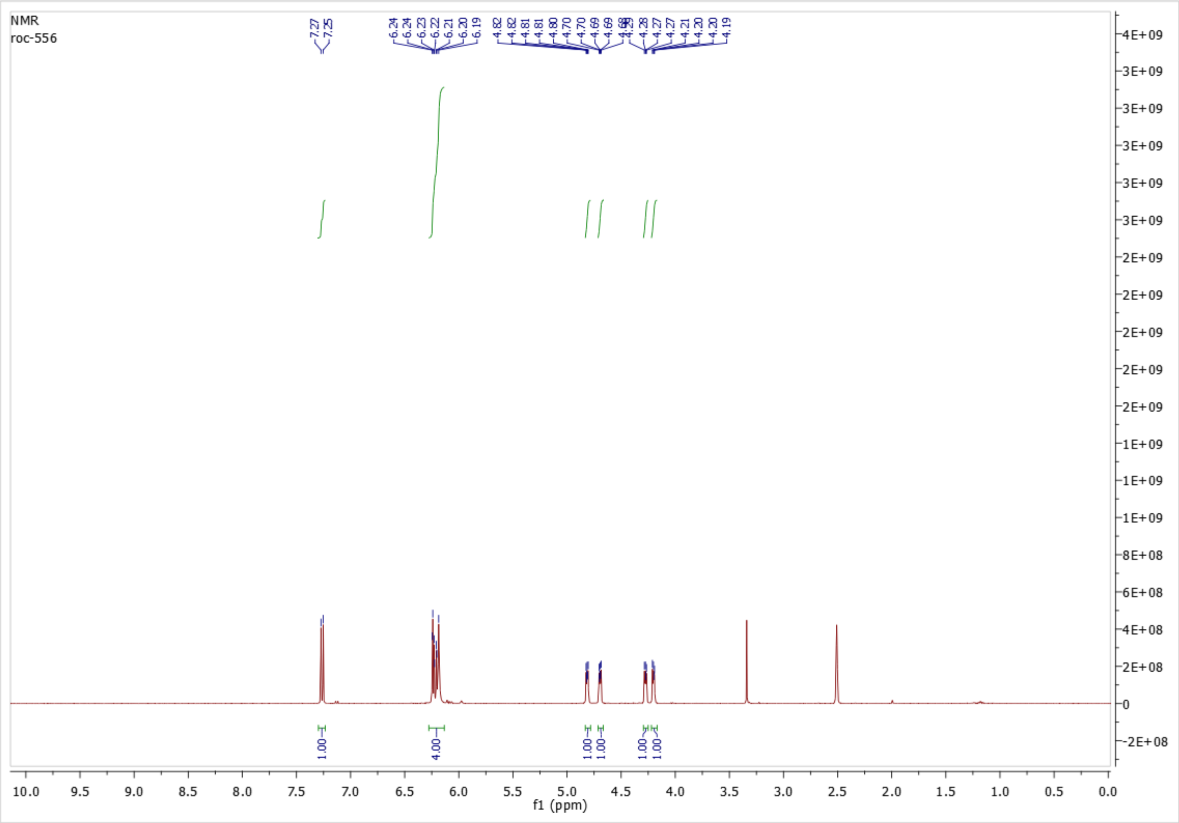

<u>^{13}C NMR spectrum of **36a**</u>

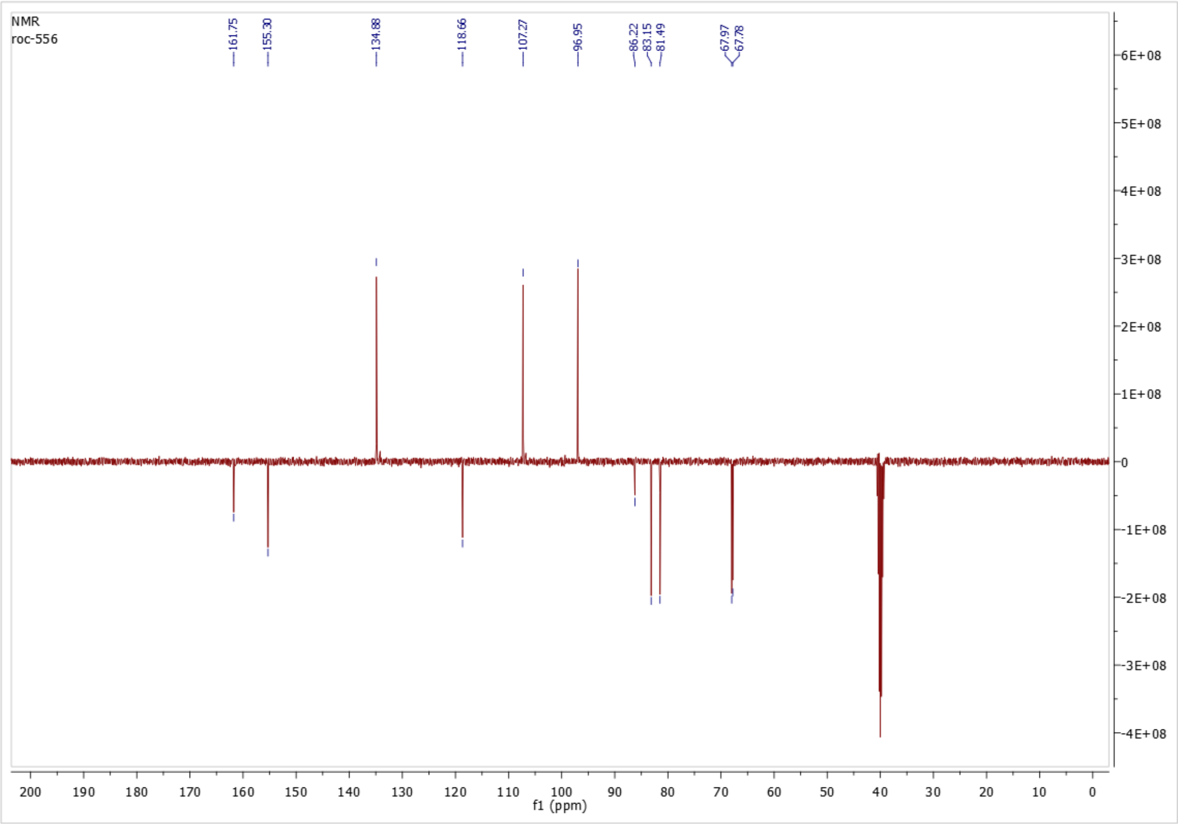

^{19}F NMR spectrum of **36a**

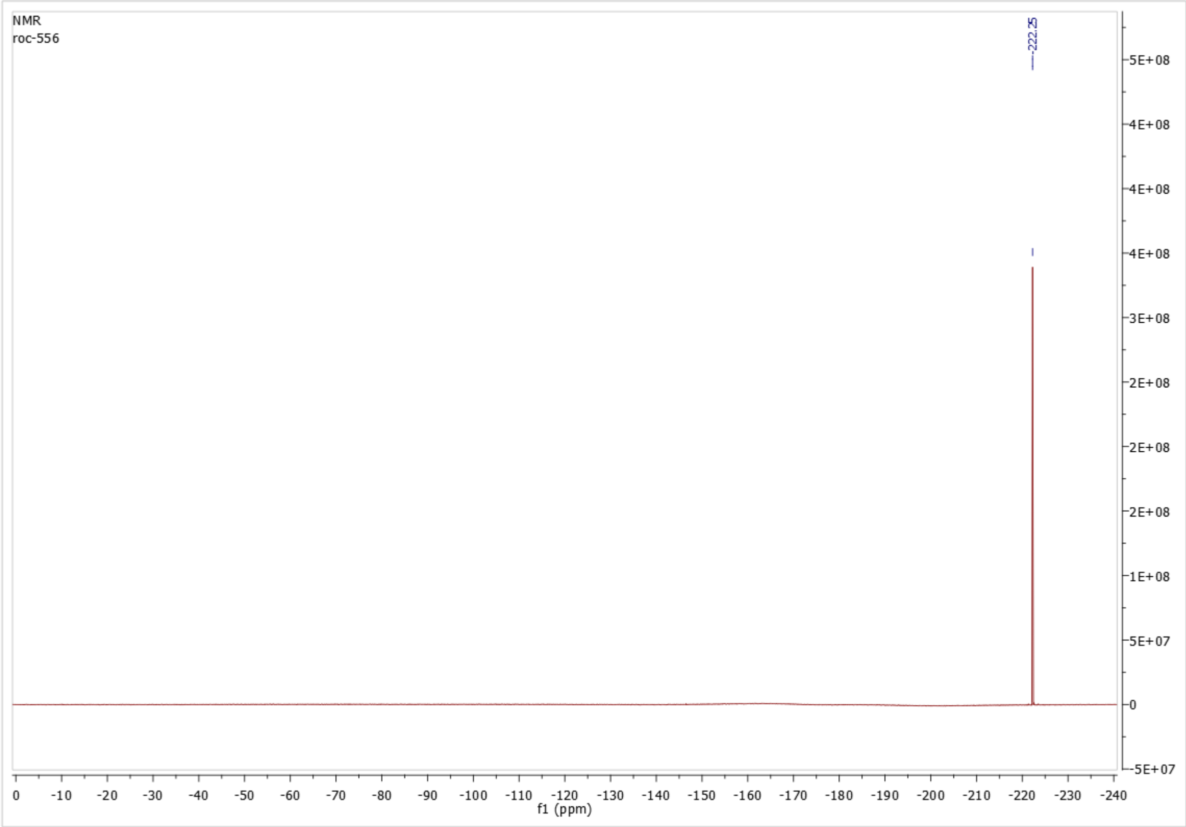

5.59. 4-Amino-2-(2-(benzyloxy)ethoxy)benzonitrile (**36b**)

^{1}H NMR spectrum of **36b**

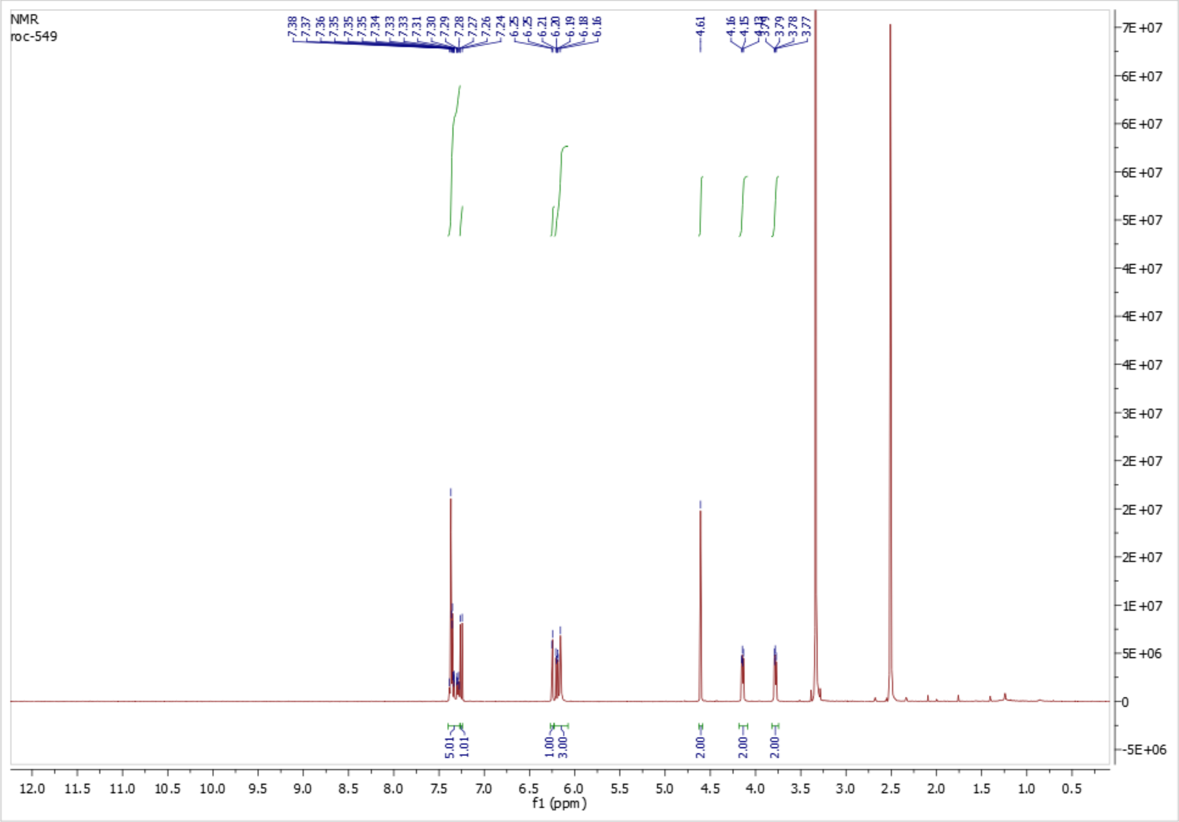

^{13}C NMR spectrum of **36b**

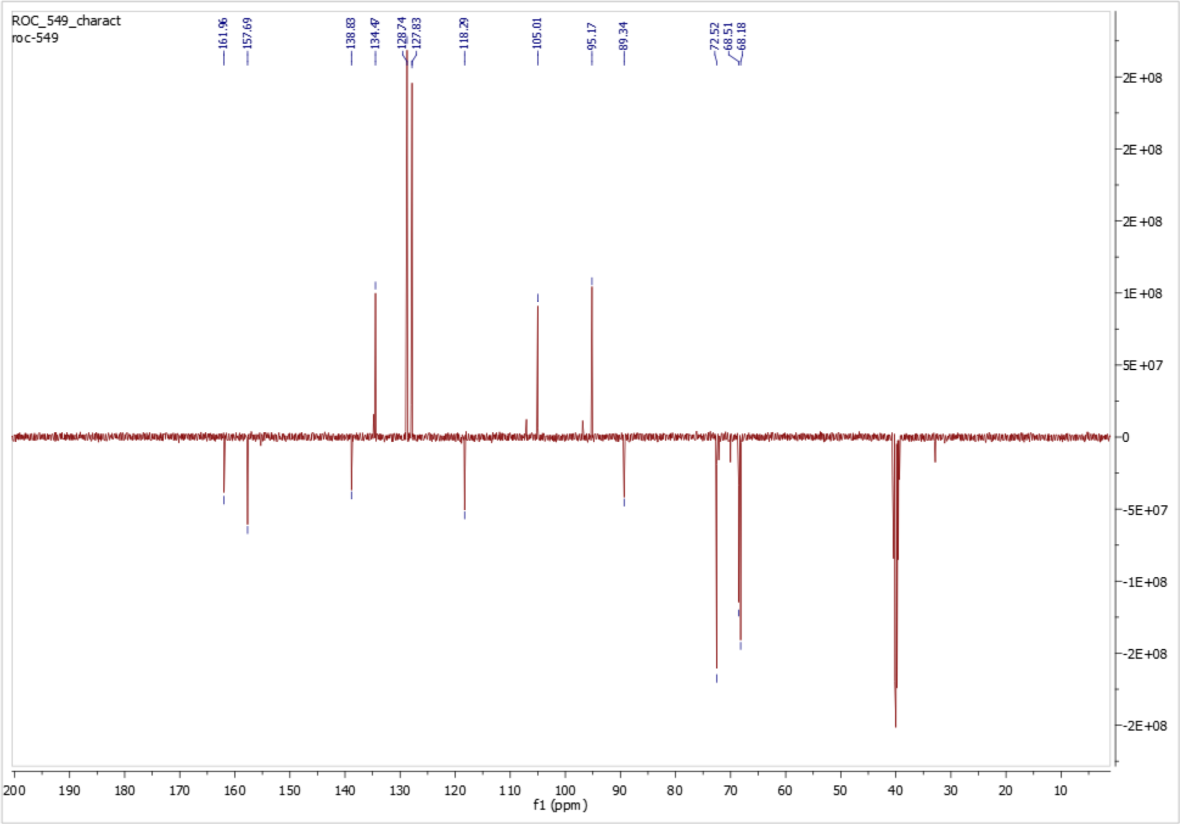

5.60. ((3-(((4-Cyano-3-(2-fluoroethoxy)phenyl)amino)methyl)-6-fluoroquinolin-2-yl)oxy)methyl pivalate (**37a**)

<u>^{1}H NMR spectrum of **37a**</u>

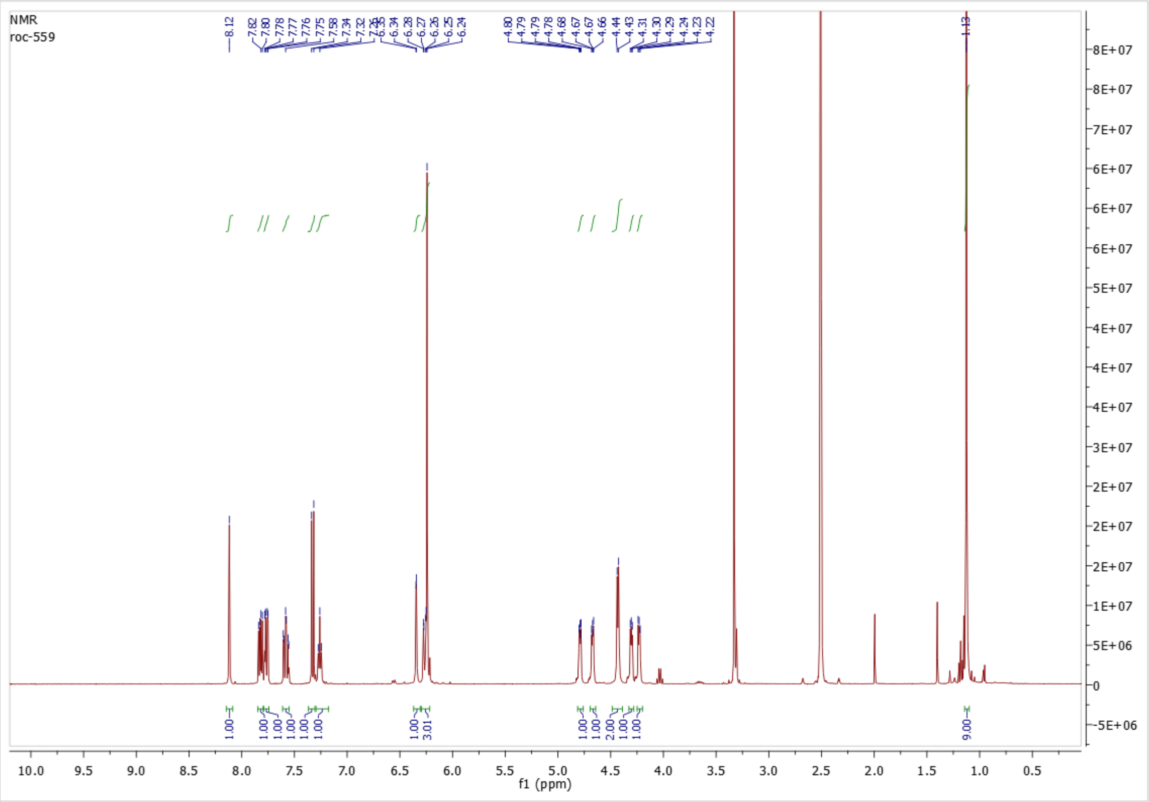

<u>^{13}C NMR spectrum of **37a**</u>

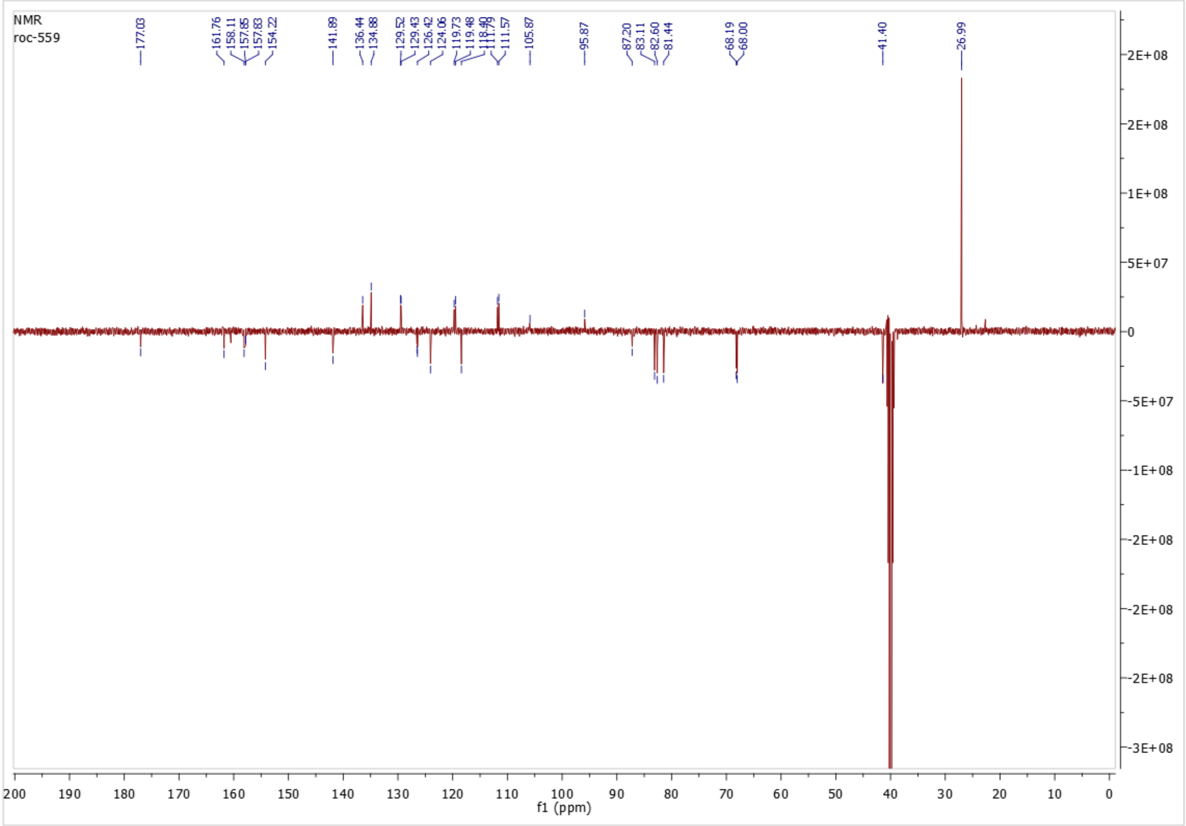

<u>^{19}F NMR spectrum of **37a**</u>

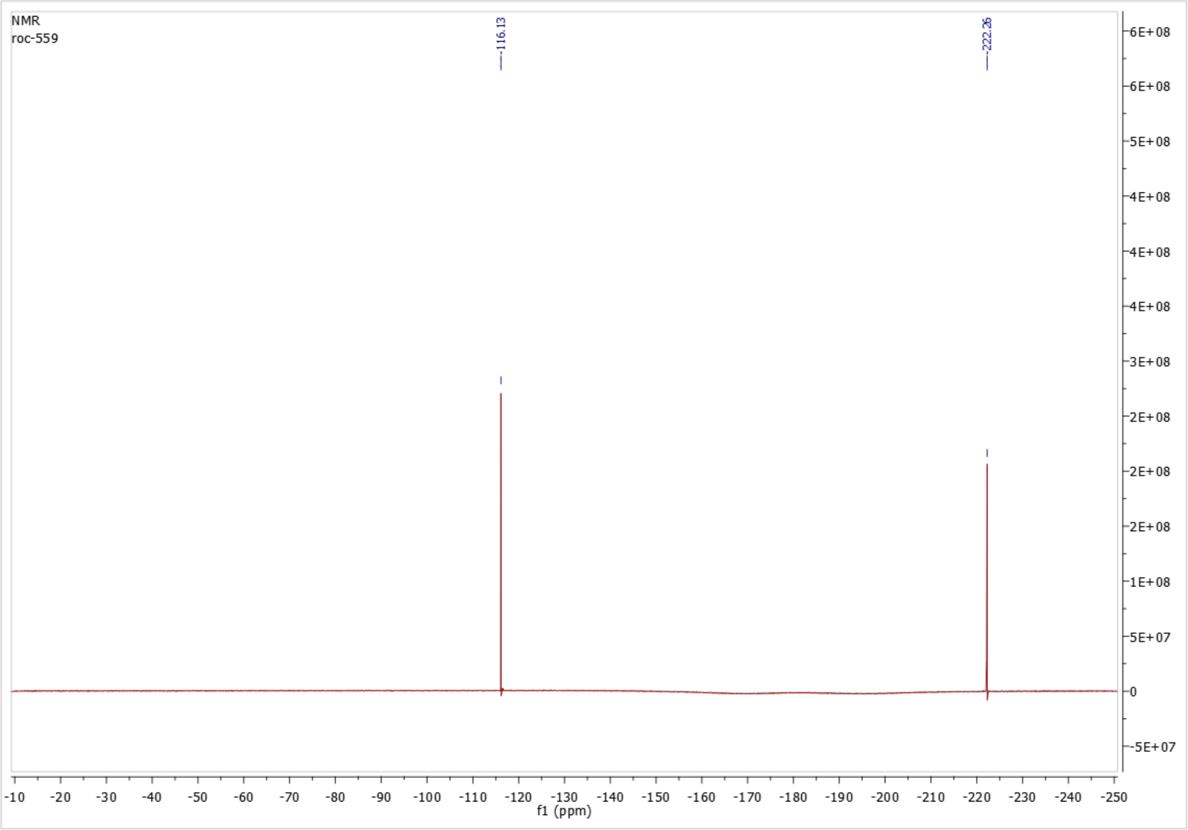

5.61. 2-(2-Cyano-5-(((6-fluoro-2-((pivaloyloxy)methoxy)quinolin-3-yl)methyl)amino)phenoxy)ethyl benzoate (**37b**)

^{1}H NMR spectrum of **37b**

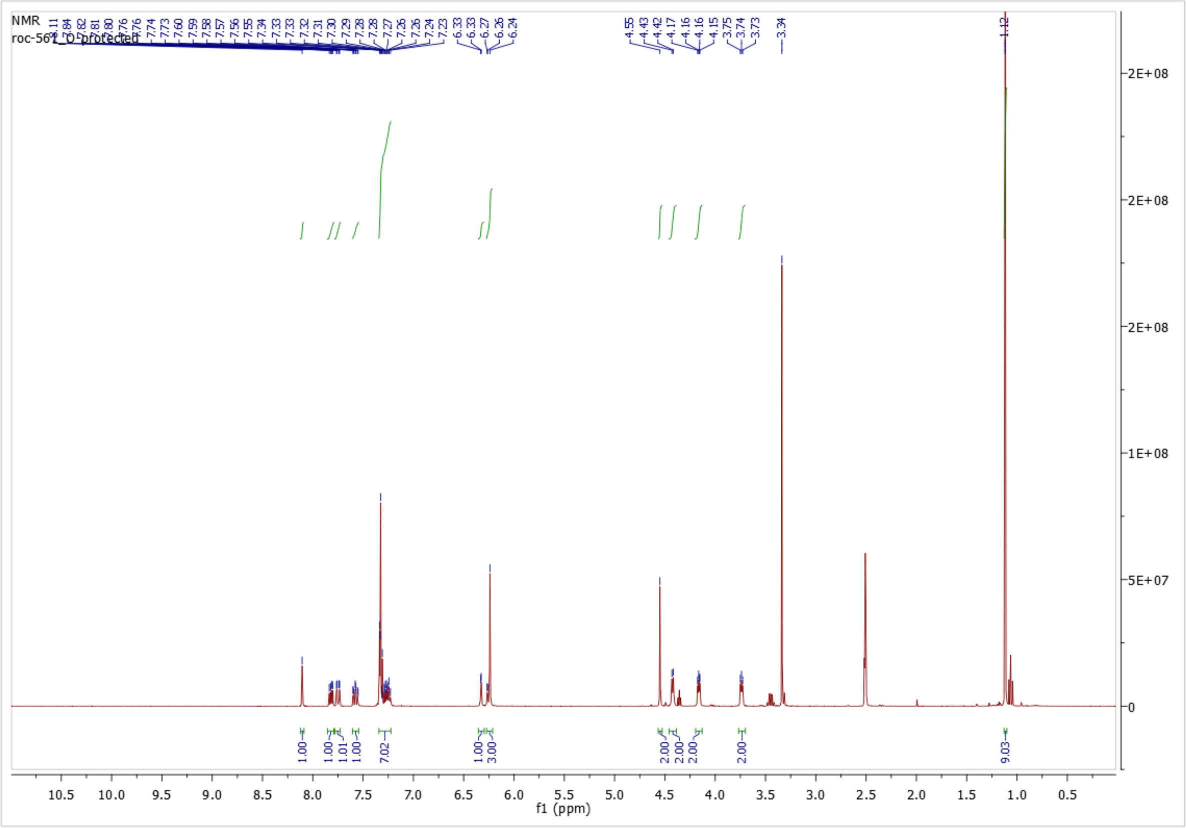

^{13}C NMR spectrum of **37b**

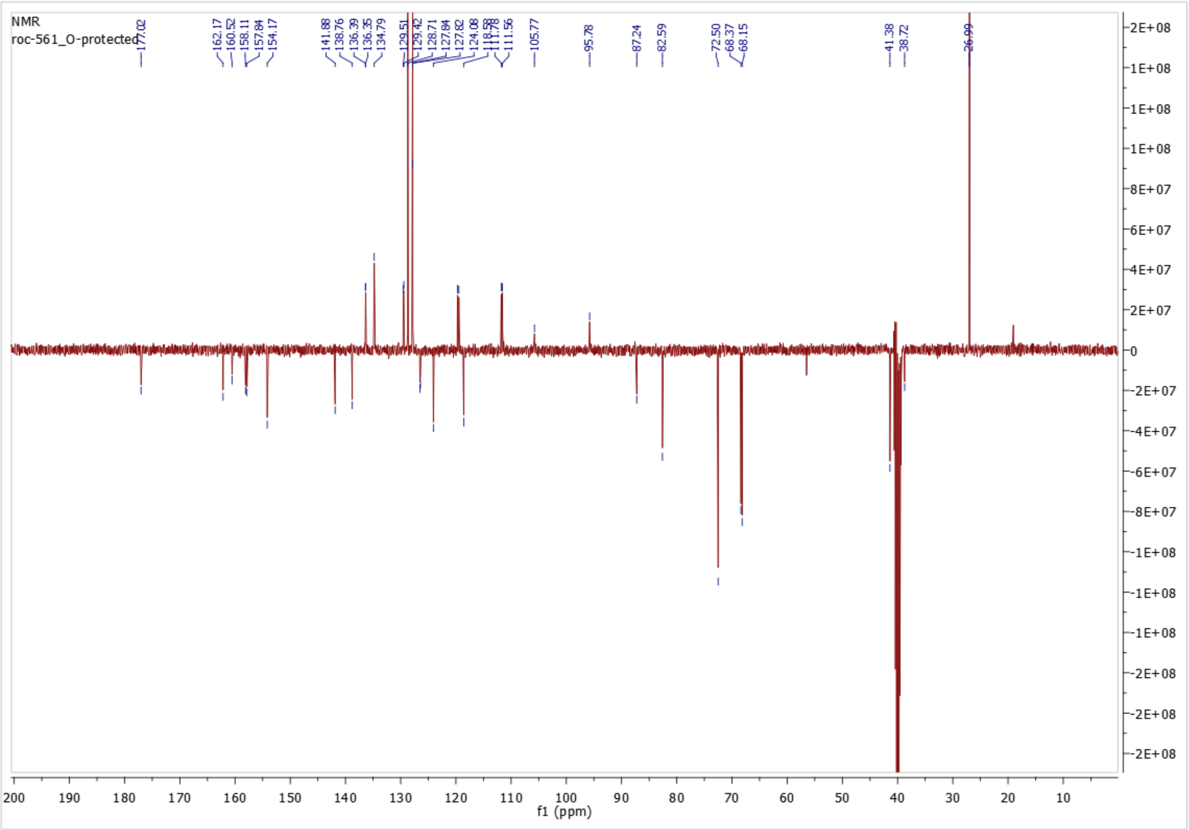

^{19}F NMR spectrum of **37b**

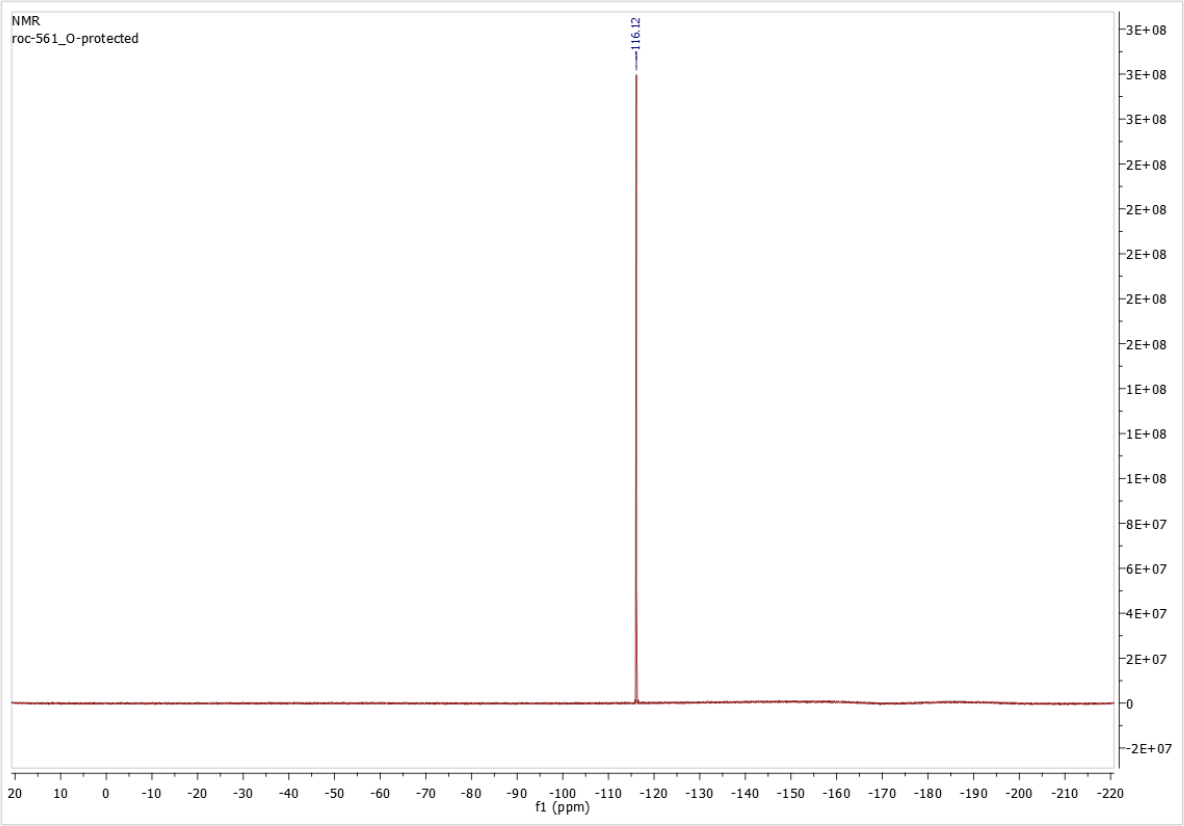

5.62. 2-(2-Cyano-5-(((6-fluoro-2-oxo-1-((pivaloyloxy)methyl)-1,2-dihydroquinolin-3-yl)methyl)amino)phenoxy)ethyl benzoate (**S6**)

^{1}H NMR spectrum of **S6**

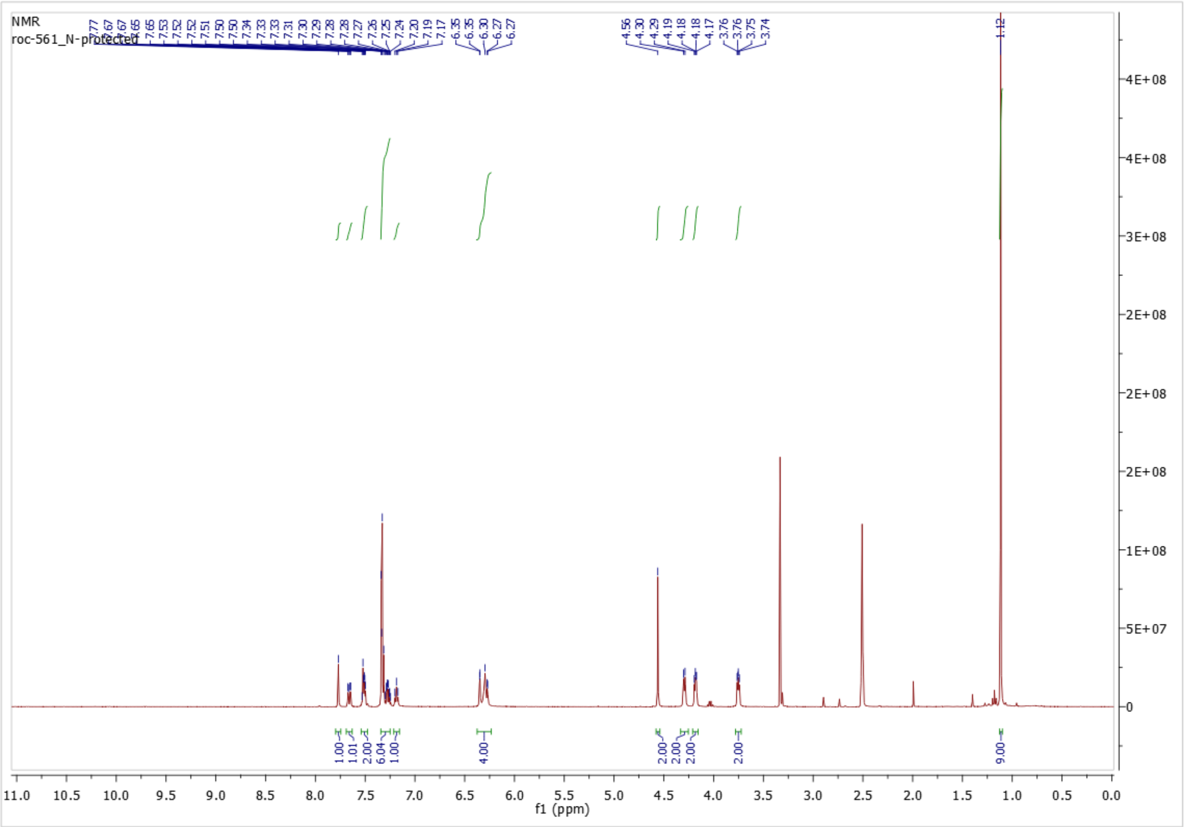

^{13}C NMR spectrum of **S6**

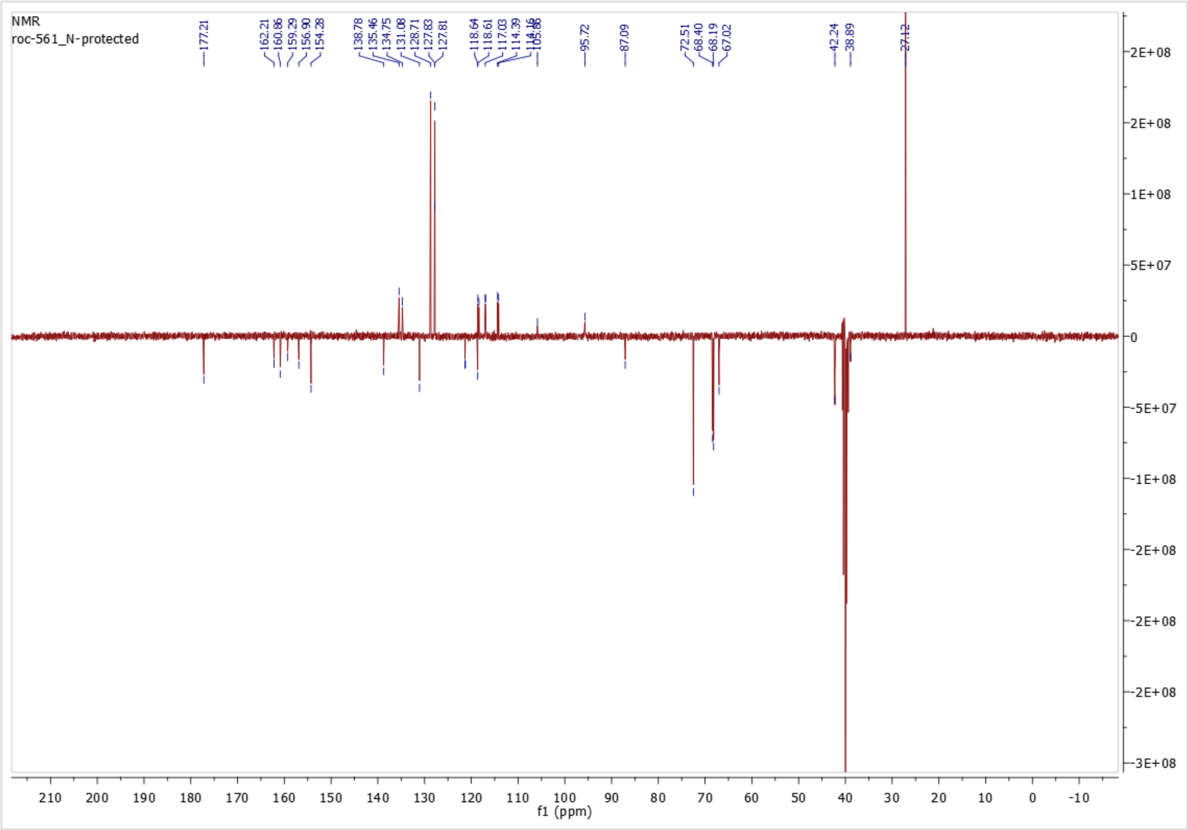

^{19}F NMR spectrum of **S6**

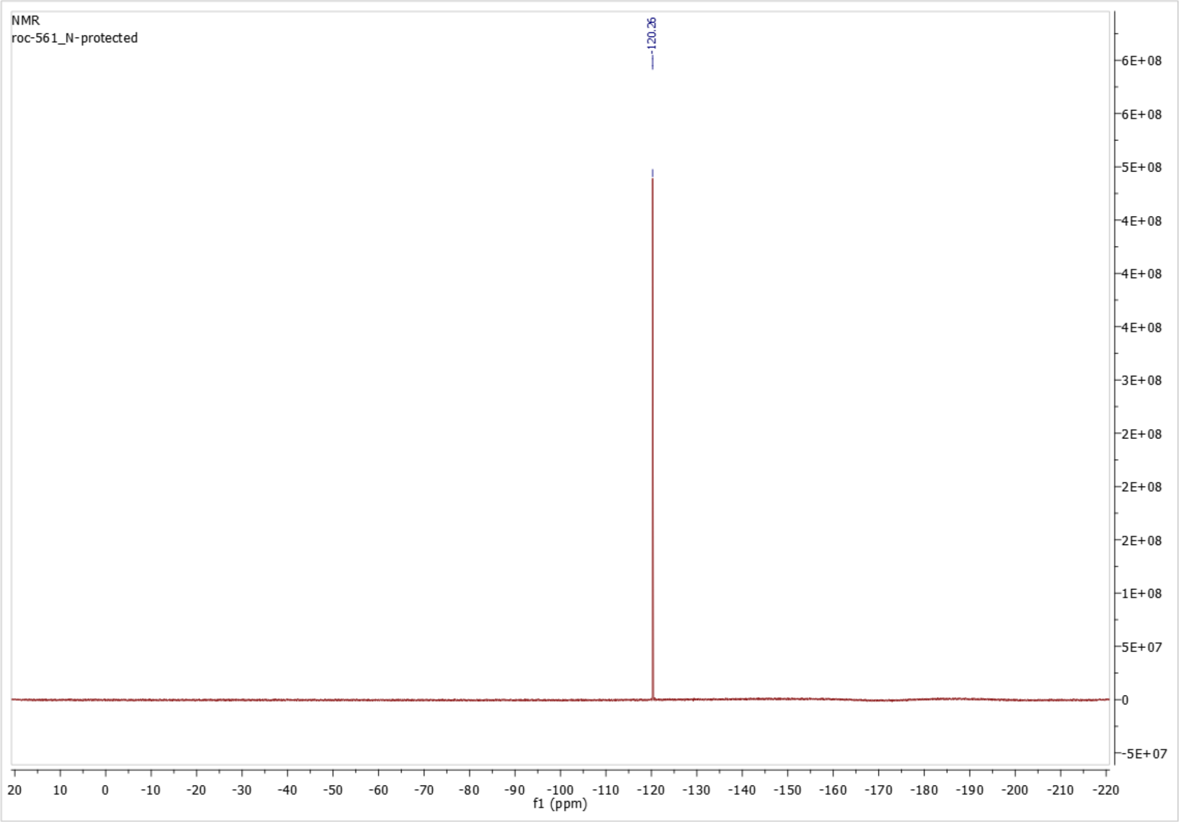

HSQC NMR spectrum of **S6**

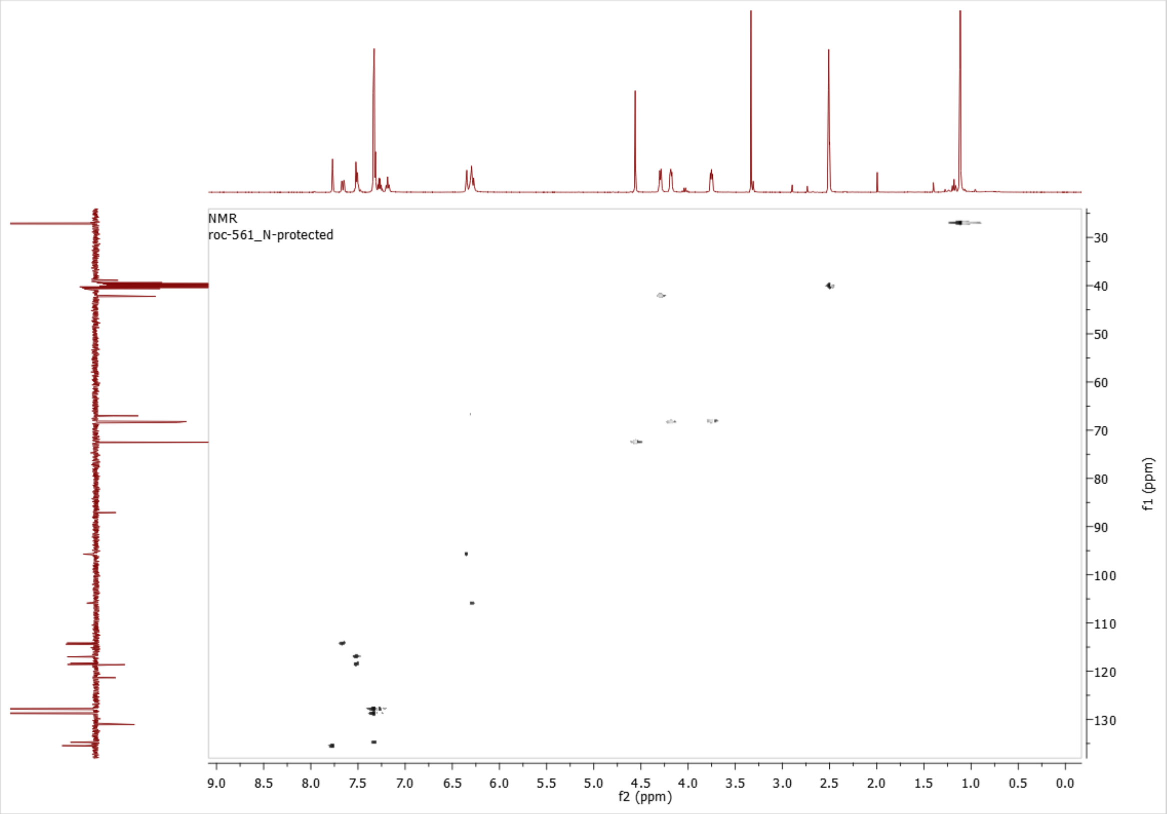

5.63. 4-(((6-Fluoro-2-oxo-1,2-dihydroquinolin-3-yl)methyl)amino)-2-(2-fluoroethoxy)benzonitrile (**5**)

^{1}H NMR spectrum of **5**

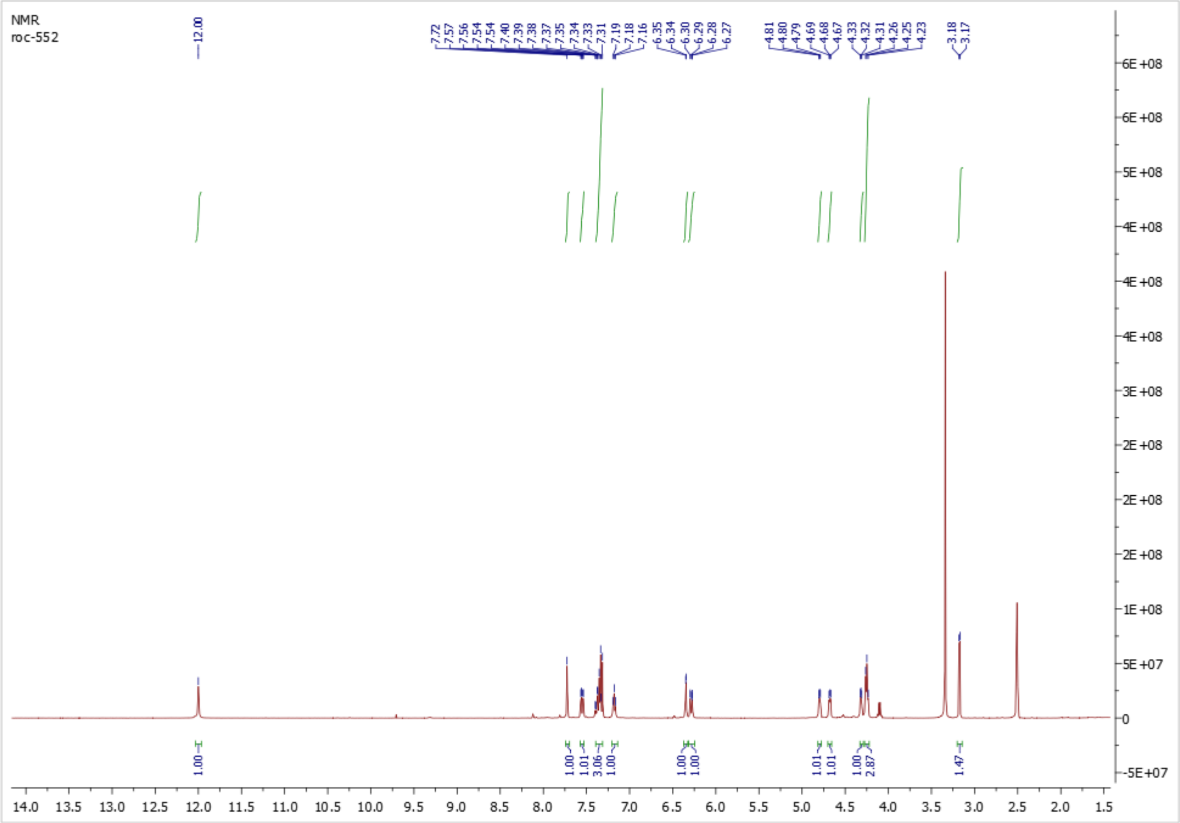

^{13}C NMR spectrum of **5**

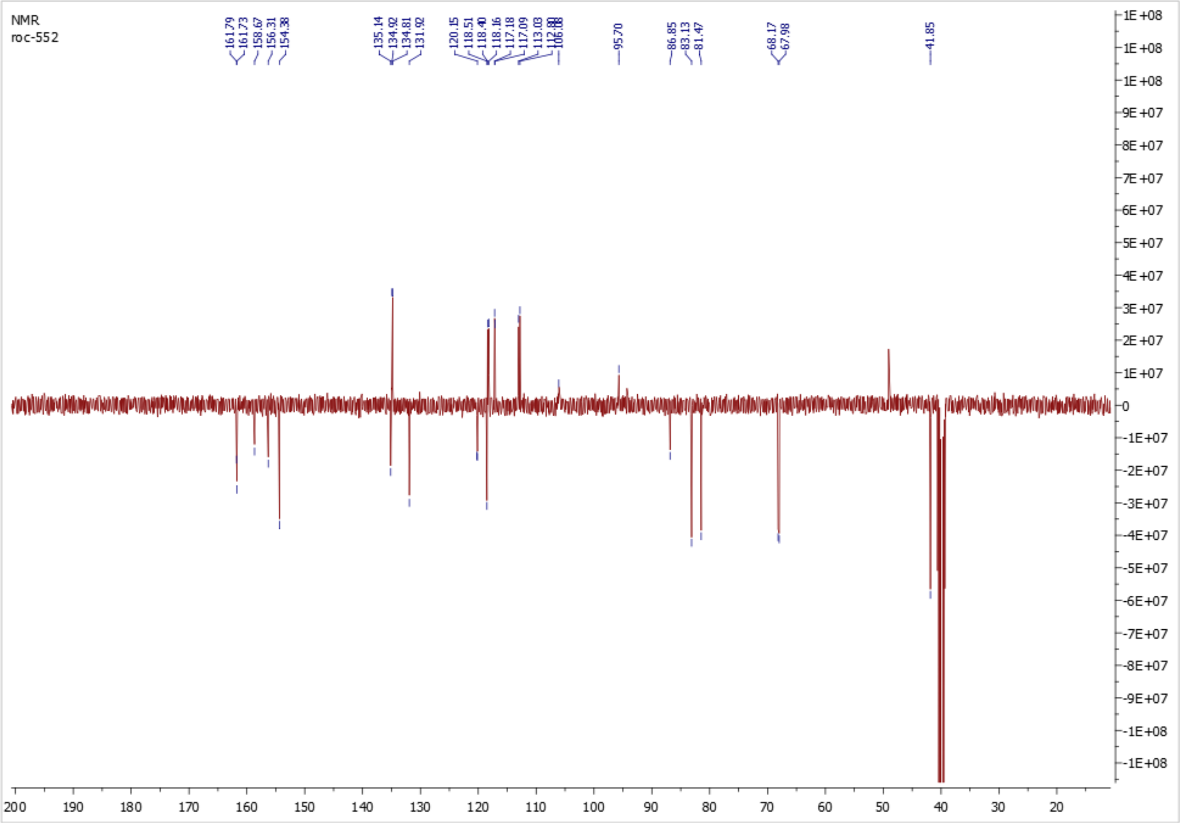

^{19}F NMR spectrum of **5**

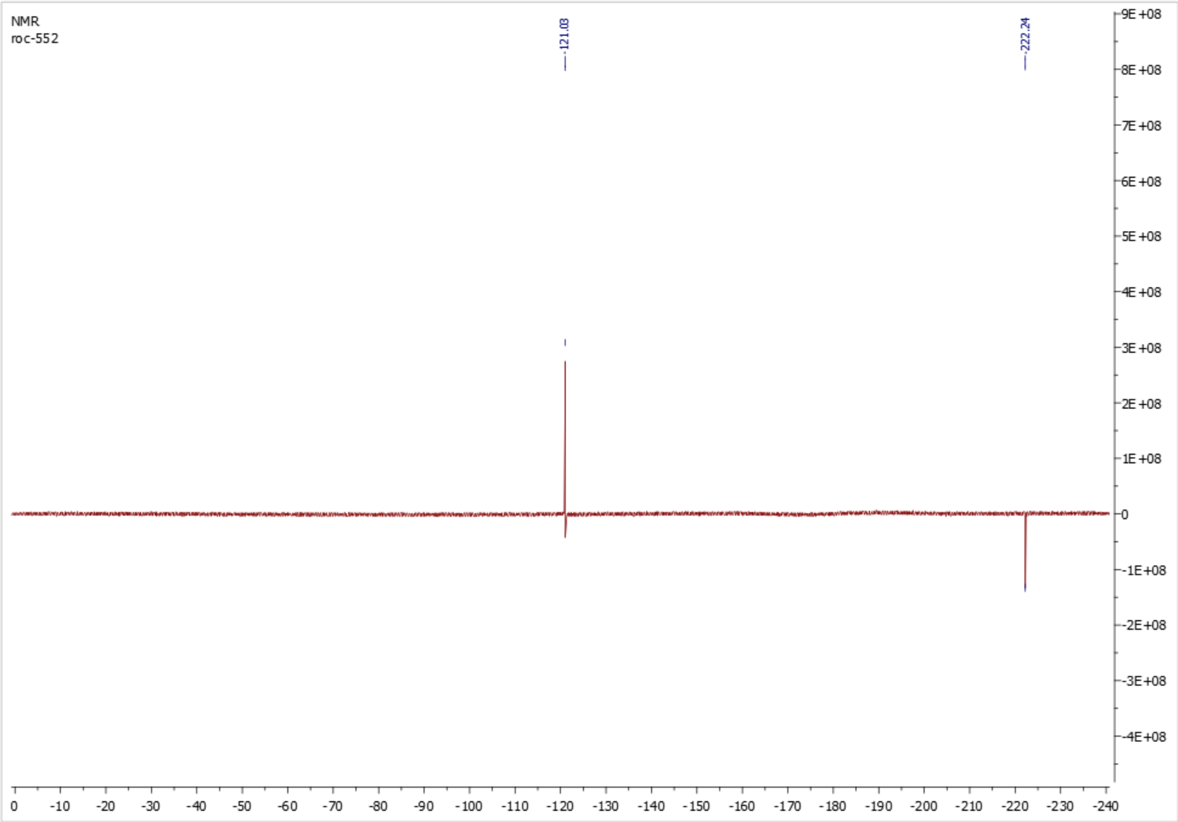

5.64. ((3-(((4-Cyano-3-(2-hydroxyethoxy)phenyl)amino)methyl)-6-fluoroquinolin-2-yl)oxy)methyl pivalate (**38**)

^{1}H NMR spectrum of **38**

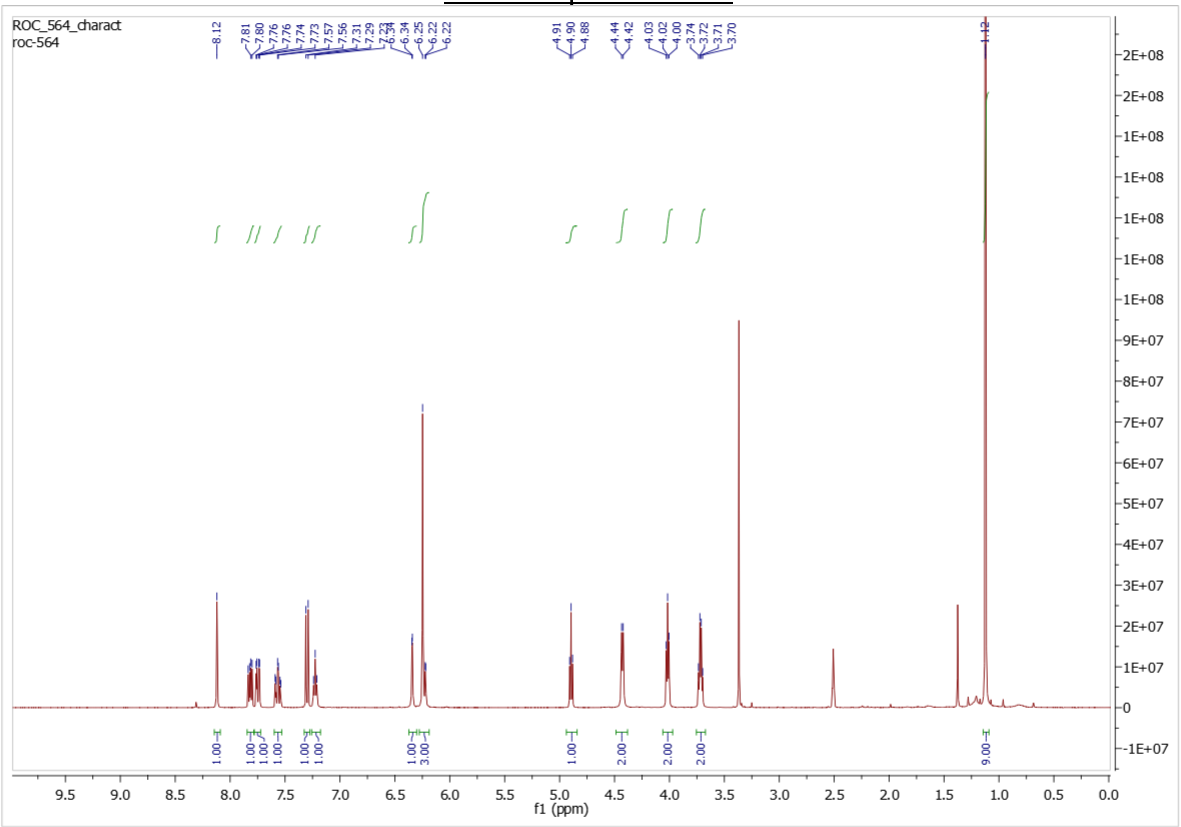

^{13}C NMR spectrum of **38**

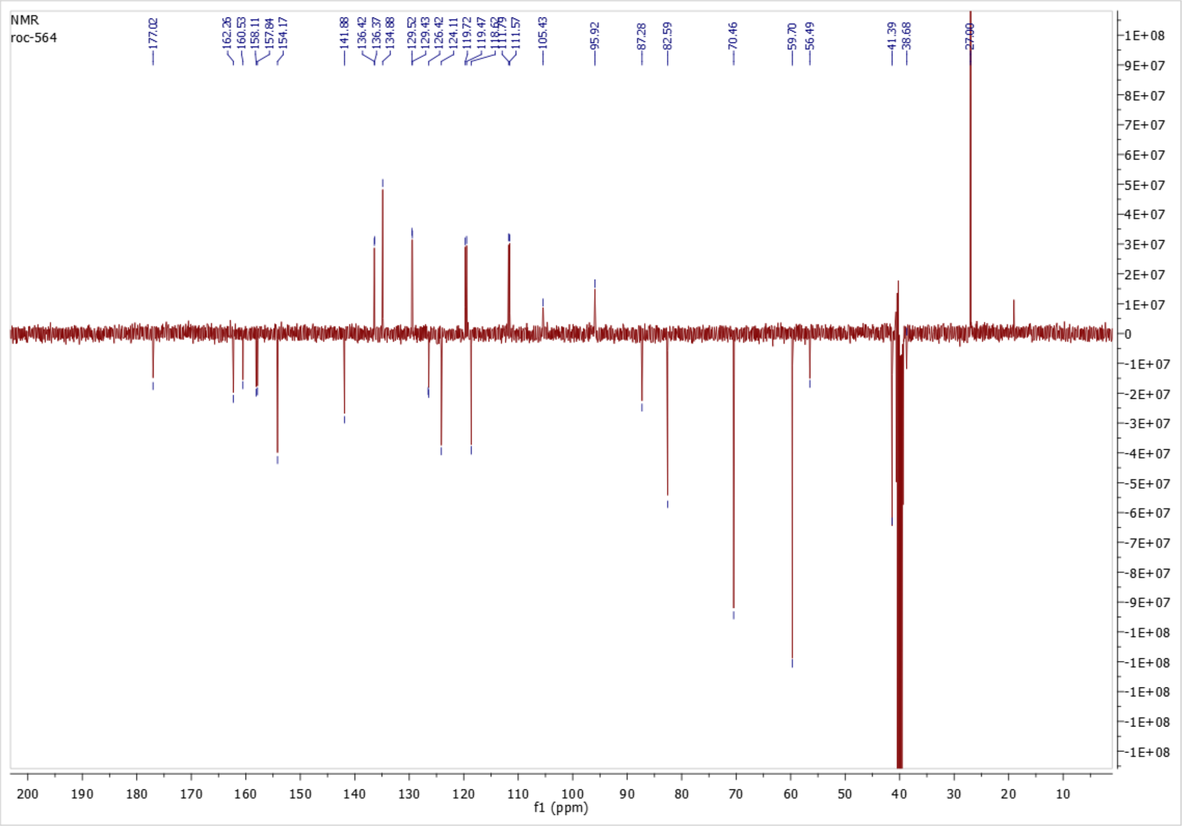

^{19}F NMR spectrum of **38**

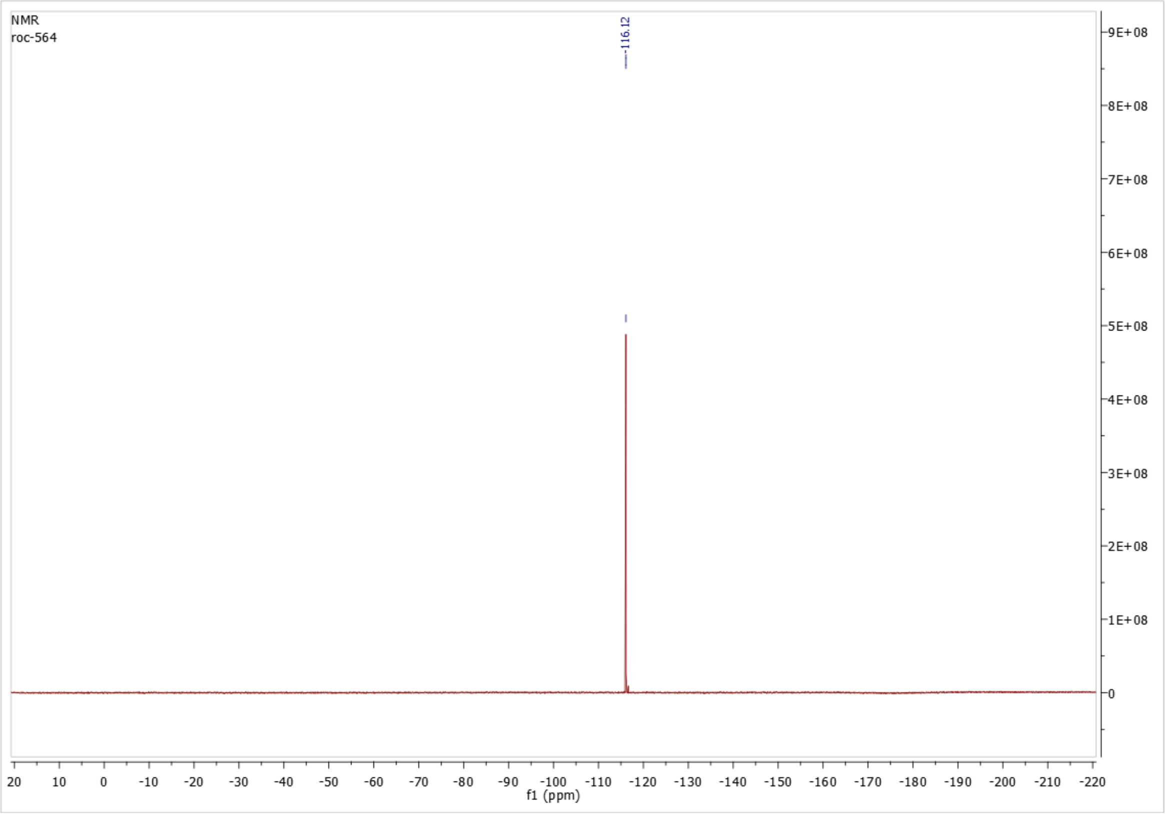

5.65. ((3-(((4-Cyano-3-(2-((methylsulfonyl)oxy)ethoxy)phenyl)amino)methyl)-6-fluoroquinolin-2-yl)oxy)methyl pivalate (**39**)

^{1}H NMR spectrum of **39**

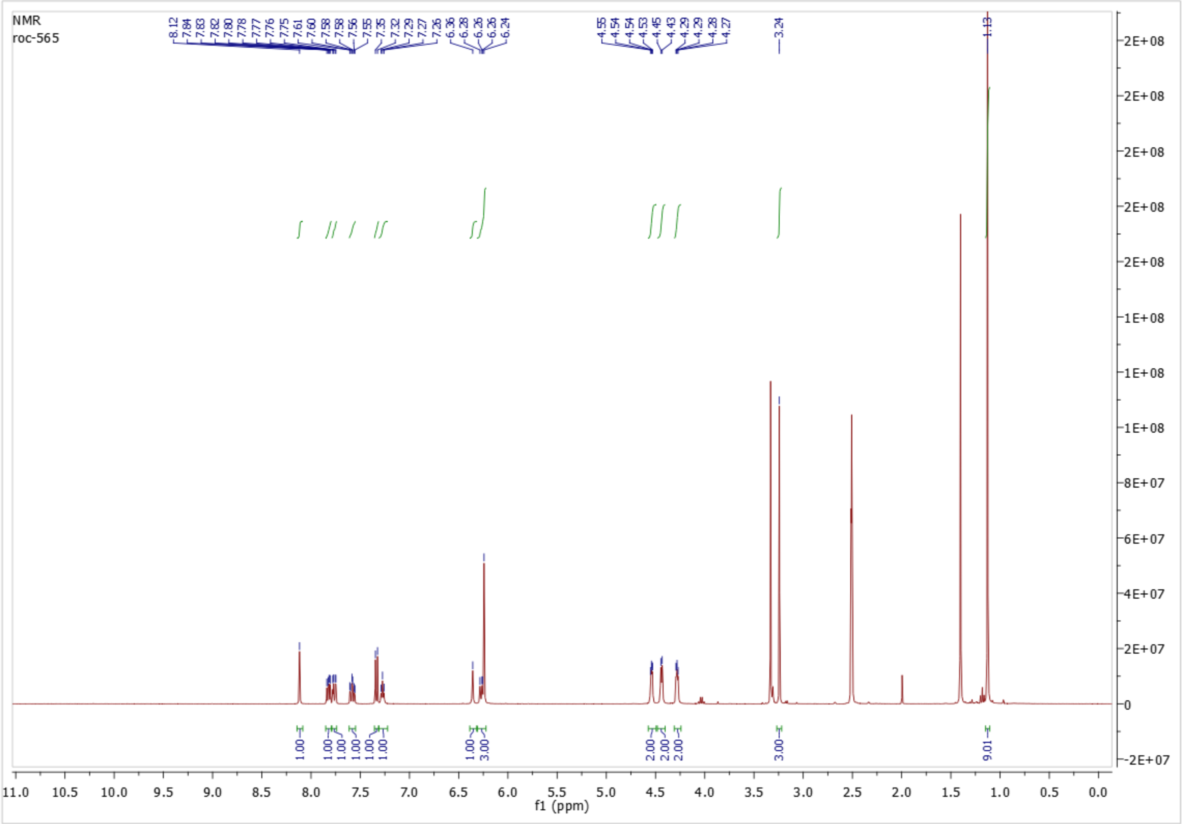

^{13}C NMR spectrum of **39**

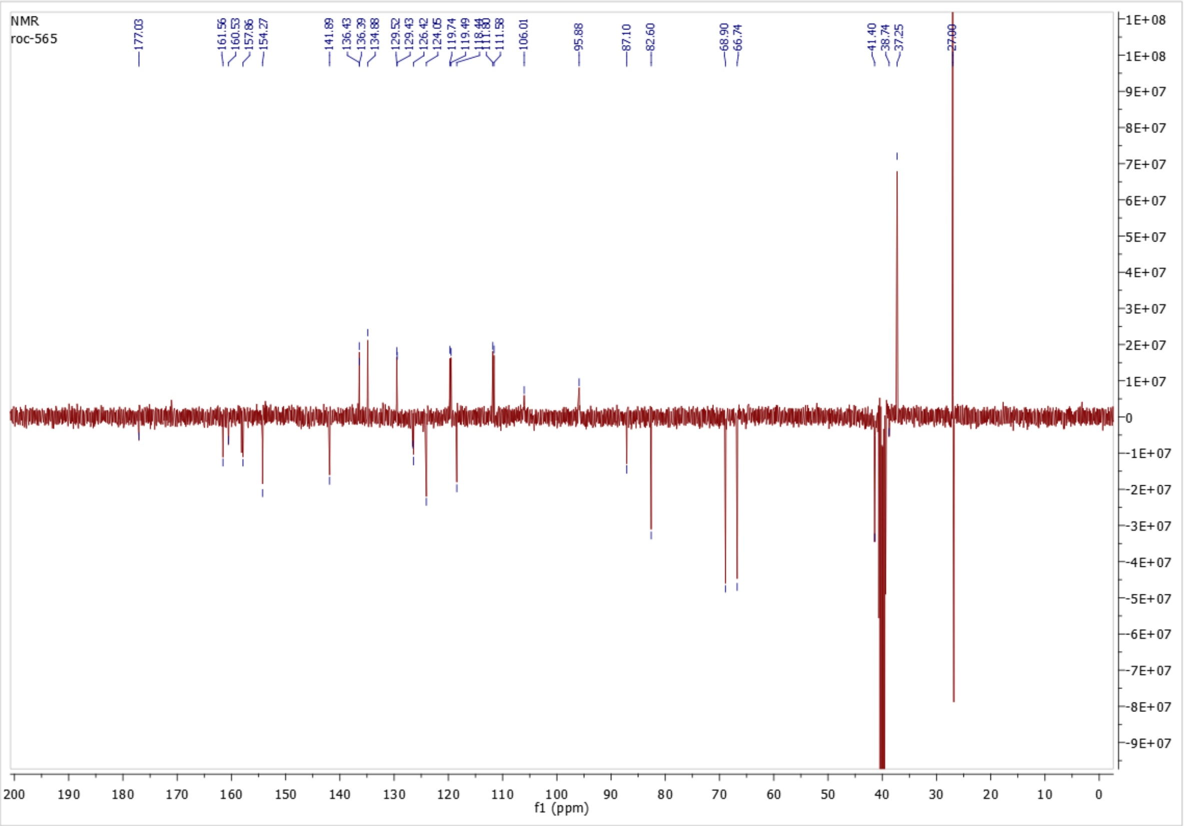

^{19}F NMR spectrum of **39**

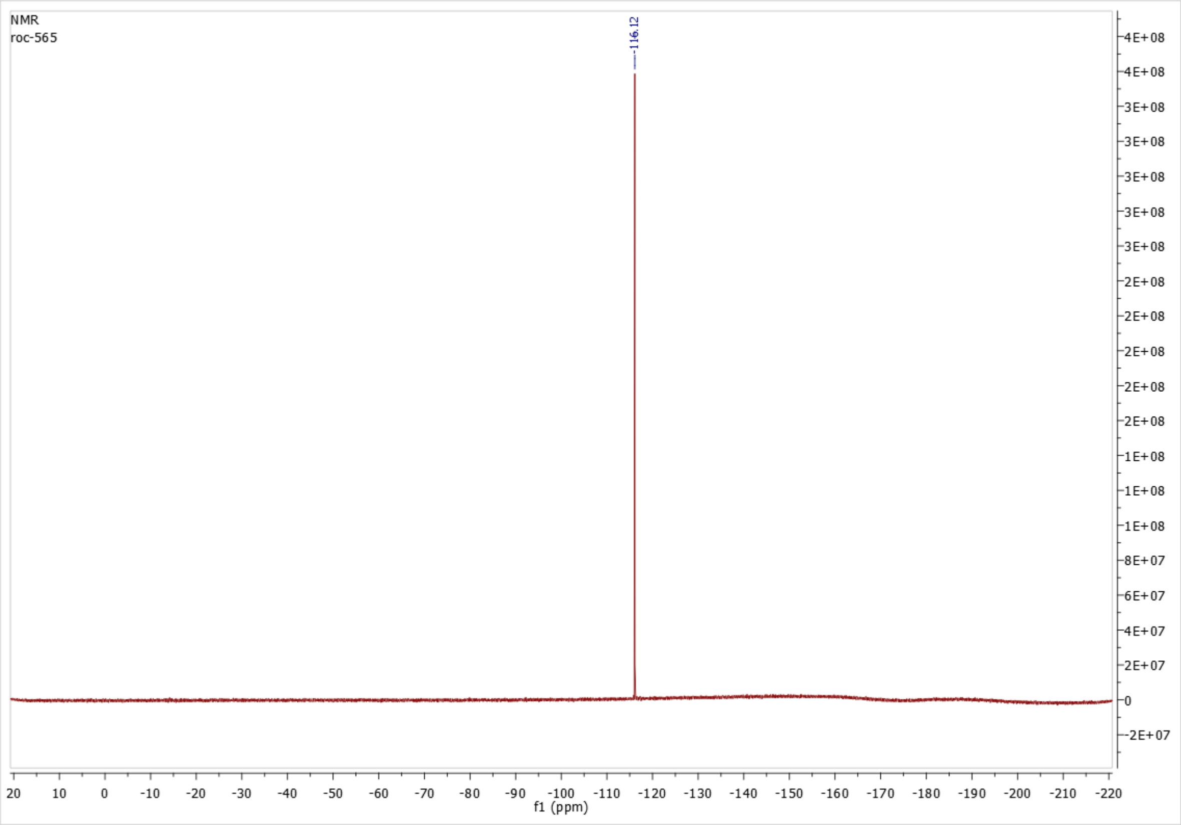

Supplement: Supplementary file 1 [file molecules-29-03939-s001.zip › molecules-3143277-supplementary.pdf]
